# A novel nano-iron supplement versus standard treatment for iron deficiency anaemia in children 6–35 months (IHAT-GUT trial): a double-blind, randomised, placebo-controlled non-inferiority phase II trial in The Gambia

**DOI:** 10.1016/j.eclinm.2023.101853

**Published:** 2023-02-09

**Authors:** Nuredin I. Mohammed, James Wason, Thomas Mendy, Stefan A. Nass, Ogochukwu Ofordile, Famalang Camara, Bakary Baldeh, Chilel Sanyang, Amadou T. Jallow, Ilias Hossain, Nuno Faria, Jonathan J. Powell, Andrew M. Prentice, Dora I.A. Pereira

**Affiliations:** a Medical Research Council Unit The Gambia at the London School of Hygiene & Tropical Medicine, Banjul, Gambia; b MRC Biostatistics Unit, Institute of Public Health, University of Cambridge, Cambridge, CB2 0SR, UK; c Population Health Sciences Institute, Newcastle University, Newcastle, NE2 4BN, UK; d Medical Humanities, Amsterdam-UMC - VUmc Location, Vrije Universiteit, Amsterdam, the Netherlands; e Department of Veterinary Medicine, University of Cambridge, Madingley Road, Cambridge, CB3 0ES, UK; f Department of Pathology, University of Cambridge, Tennis Court Road, Cambridge, CB2 1QP, UK

**Keywords:** Iron deficiency, Anaemia, Children, Iron hydroxide adipate tartrate (IHAT), Iron supplementation, Clinical trial, Phase II

## Abstract

**Background:**

Iron deficiency anaemia (IDA) is the leading cause of years lost to disability in most sub-Saharan African countries and is especially common in young children. The IHAT-GUT trial assessed the efficacy and safety of a novel nano iron supplement, which is a dietary ferritin analogue termed iron hydroxide adipate tartrate (IHAT), for the treatment of IDA in children under 3 years of age.

**Methods:**

In this single-country, randomised, double-blind, parallel, placebo-controlled, non-inferiority Phase II study in The Gambia, children 6–35 months with IDA (7≤Hb < 11 g/dL and ferritin<30 μg/L) were randomly assigned (1:1:1) to receive either IHAT, ferrous sulphate (FeSO_4_) or placebo daily for 3 months (85 days). The daily iron dose was 12.5 mg Fe equivalent for FeSO_4_ and the estimated dose with comparable iron-bioavailability for IHAT (20 mg Fe). The primary efficacy endpoint was the composite of haemoglobin response at day 85 and correction of iron deficiency. The non-inferiority margin was 0.1 absolute difference in response probability. The primary safety endpoint was moderate-severe diarrhoea analysed as incidence density and prevalence over the 3 months intervention. Secondary endpoints reported herein include hospitalisation, acute respiratory infection, malaria, treatment failures, iron handling markers, inflammatory markers, longitudinal prevalence of diarrhoea and incidence density of bloody diarrhoea. Main analyses were per-protocol (PP) and intention-to-treat (ITT) analyses. This trial is registered with clinicaltrials.gov (NCT02941081).

**Findings:**

Between Nov 2017 and Nov 2018, 642 children were randomised into the study (214 per group) and included in the ITT analysis, the PP population included 582 children. A total of 50/177 (28.2%) children in the IHAT group achieved the primary efficacy endpoint, as compared with 42/190 (22.1%) in the FeSO_4_ group (OR 1.39, 80% CI 1.01–1.91, PP population) and with 2/186 (1.1%) in the placebo group. Diarrhoea prevalence was similar between groups, with 40/189 (21.2%) children in the IHAT group developing at least one episode of moderate-severe diarrhoea over the 85 days intervention, compared with 47/198 (23.7%) in the FeSO_4_ group (OR 1.18, 80% CI 0.86–1.62) and 40/195 (20.5%) in the placebo group (OR 0.96, 80% CI 0.7–1.33, PP population). Incidence density of moderate-severe diarrhoea was 2.66 in the IHAT group and 3.42 in the FeSO_4_ group (RR 0.76, 80% CI 0.59–0.99, CC-ITT population).

There were 143/211 (67.8%) children with adverse events (AEs) in the IHAT group, 146/212 (68.9%) in the FeSO_4_ group and 143/214 (66.8%) in the placebo group. There were overall 213 diarrhoea-related AEs; 35 (28.5%) cases reported in the IHAT group compared with 51 (41.5%) cases in the FeSO_4_ group and 37 (30.1%) cases in the placebo group.

**Interpretation:**

In this first Phase II study conducted in young children with IDA, IHAT showed sufficient non-inferiority compared to standard-of-care FeSO_4_, in terms of ID correction and haemoglobin response, to warrant a definitive Phase III trial. In addition, IHAT had lower incidence of moderate-severe diarrhoea than FeSO_4_, with no increased adverse events in comparison with placebo.

**Funding:**

The 10.13039/100000865Bill & Melinda Gates Foundation (OPP1140952).

**Research in context**

**Evidence before this study**

Simple ferrous salts (sulphate, fumarate or gluconate) are widely used as affordable supplements to combat iron deficiency anaemia in low-income settings. With only partial absorption in the small intestine the majority of the iron passes to the large intestine where it is redox active and also acts as a substrate for potentially pathogenic gut flora often causing dysbiosis, gastrointestinal side effects that impair adherence to treatment (cramps, nausea, diarrhoea, constipation, black stools), and occasionally severe diarrhoea. On 11th June 2021 we screened for novel iron supplements that might have a more benign side–effect profile by searching clinical trials registers (clinicaltrials.gov, clinicaltrialsregister. eu, ICTRP) using search terms ‘iron deficiency anaemia’ and ‘iron’ restricted to trials in children. Excluding intravenous agents for specialist conditions (e.g. ferumoxytol, ferric carboxymaltose, iron dextran) the compounds under investigation were lactoferrin (7 trials) and single trials of ferrous acetyl-aspartate casein (ASP), NovaFerrum ®, FerroSanol ®, SantaFerr ®, and iron hydroxide adipate tartrate (IHAT). In general, these specialist products would be prohibitively expensive to use in community-wide intervention programmes in low-income countries.

**Added value of this study**

Iron hydroxide adipate tartrate (IHAT) is a low-cost novel nano-particulate iron supplement that is a plant ferritin analogue and is designed to have a benign side–effect profile by withholding any unabsorbed iron from redox activity and the gut flora. IHAT is the first nano iron approved by the European Commission (EC) as a novel food (2022/1373 5th Aug 2022). In this Phase II randomised trial in iron deficient anaemic rural Gambian children aged 6–35 months IHAT was non-inferior to ferrous sulphate. IHAT was non-inferior to the government standard care (ferrous sulphate) and both were superior to placebo. Resolution of anaemia was more likely with IHAT than with standard care or placebo. IHAT had lower incidence of moderate-severe diarrhoea than ferrous sulphate and adverse events did not differ from placebo. Further studies with higher statistical power are required to confirm these findings.

**Implications of all the available evidence**

IHAT offers an affordable form of safe, bio-available and dietary-relevant supplemental iron for low-income countries and merits consideration in micronutrient intervention strategies. A pivotal study in pregnant women and children is now warranted.

## Introduction

Iron deficiency (ID) and its associated anaemia (IDA) remain the most common form of micronutrient malnutrition in the world today. Globally, IDA is estimated to affect 1.2 billion people, the majority of whom are children and women from resource-poor countries, and it is responsible for an estimated loss of 35 million DALYs (1.5% of total disability-adjusted life years).^1^ IDA is estimated to cause more years lived with disability (YLD) than all other micronutrient deficiencies, haemoglobinopathies and haemolytic anaemias combined, and is one of the top three causes of disease burden in children and adolescents worldwide.^1^^,^^2^ Most sub-Saharan Africa countries have an anaemia prevalence above 40% in young children and pregnant women, classifying it as a severe public health problem according to the World Health Organization (WHO).^3^

The effects of anaemia on child cognition are well recognised with an analysis of 5 separate trials finding a combined 1.73 lower IQ points per 1 g/dL decrease in haemoglobin^4^^,^^5^. Even mild iron deficiency in the absence of anaemia appears to impair intellectual development in young children, whilst overt IDA is associated with increased risk of serious morbidity, poor motor and mental development in children and impaired immunity.^5^, ^6^, ^7^, ^8^

Iron supplementation with simple ferrous salts is cheap and widely available, but can release redox-reactive iron which is non-physiological and has been associated with significant side-effects and adverse events that limit its tolerability and reduce adherence.^9^

Since 2005 we have been developing an engineered analogue of natural food iron as an alternative iron supplement. Iron hydroxide adipate tartrate, or IHAT, differs from other iron compounds currently used in supplementation or home fortification strategies. Its rationale challenges the established dogma that iron absorption requires ionic solubilised iron to be taken up by the duodenal enterocytes and supports the growing evidence that dietary non-haem iron, or its digestion product, is nanoparticulate.^10^ IHAT is a tartrate-modified, nano-dispersed Fe(III) oxo-hydroxide with similar functional properties and small primary particle size (∼2 nm) to the iron form found in the ferritin core (i.e. ferrihydrite).^11^

The main purpose of the IHAT-GUT study was to determine whether supplementation with IHAT safely corrects IDA in young children and to compare this to the present standard-of-care for iron supplementation. We hypothesised that 12-weeks’ supplementation with IHAT would correct iron deficiency and improve haemoglobin levels in young children without causing diarrhoea or inducing intestinal inflammation or detrimental changes in the gut microbiome. Here we report the first clinical study to assess the efficacy and safety of IHAT in anaemic children living in resource-poor rural areas of The Gambia.

## Methods

### Trial design

The IHAT-GUT trial was a three-arm, parallel, individually randomised, placebo-controlled, double-blind study of iron supplementation in young children with mild-to-moderate iron deficiency anaemia. The trial was conducted in The Gambia according to a pre-specified protocol (see Supplementary Data S1 for full protocol and Pereira et al.^12^ for published protocol). This trial is registered with clinicaltrials.gov, NCT02941081.

### Trial governance and ethics

The trial was conducted in accordance with the ethical principles of the Declaration of Helsinki, and that are consistent with the International Conference on Harmonisation (ICH) requirements for Good Clinical Practice (GCP), and the applicable regulatory requirements. The study sponsor was the London School of Hygiene and Tropical Medicine (LSHTM) and the study was conducted at the Medical Research Council (MRC) Unit The Gambia at LSHTM (MRCG). Scientific advice on the study protocol was given by the UK Medicines and Healthcare products Regulatory Agency (MHRA 1400, 21/12/2016). The study protocol and any subsequent amendments were reviewed and approved by The Gambia Government/MRC Joint Ethics Committee (reference SCC1489). Clinical Trials Authorisation was granted by the Medicines Control Agency, The Gambia (HP373/347/16/MJK (80)).

### Data and safety monitoring

An independent local safety monitor (LSM) and an independent Data and Safety Monitoring Board (DSMB) monitored quality control of the data, progress of recruitment and safety aspects of the IHAT-GUT trial, including the regular review of all adverse events. The LSM reviewed adverse events monthly and the DSMB reviewed adverse events quarterly during the study. Serious adverse events were reported in real time to both the LSM and the DSMB. No interim analysis were requested by the DSMB during this study.

### Participants

Further details on Participants, Procedures and Outcomes are provided in Supplementary Data S2.

The study population in IHAT-GUT was children under the age of 3 years inhabiting the north bank rural communities in the Upper River Region (URR) of The Gambia in West Africa.

Inclusion and exclusion criteria were as follows.

Inclusion criteria
  - Age 6–35 months
  - Apparently healthy with no signs of acute infection
  - Free of malaria (RDT negative)
  - IDA defined as 7≤ Hb < 11 g/dL AND ferritin <30 μg/L, as per WHO recommendation on assessing iron status^13^ for children under 5 y that live in regions with high infection burden
  - Resident in the study area (and planning to remain in the study area for the duration of the trial)
  - Ability and willingness to adhere to the study protocol (daily intake of supplement and daily study visits with weekly finger prick)
  - Informed consent given by parent or guardian
Exclusion criteria
  - Severe malnutrition (height-for-age (HAZ), weight-for-age (WAZ) and weight-for-height (WHZ) z-scores < -3 standard deviations (SD).
  - Congenital anomalies (minor external congenital malformation was not an exclusion criteria)
  - Shock syndrome
  - Known chronic conditions (epilepsy, congenital heart disease/defect, nephrotic syndrome & chronic glomerular nephritis, diabetes, HIV/AIDS, psychological/mental retardation, chronic respiratory infections and chronic respiratory conditions, chronic viral hepatitis, cerebral palsy or motor disability, cancer, sickle cell, thalassaemia, tuberculosis, tropical sprue, primary immunodeficiency syndromes)
  - Currently participating in another study
  - Currently taking iron supplements/multiple micronutrient supplements
  - Currently experiencing moderate-severe diarrhoea

### Randomisation and masking

Randomisation was performed by the study statistician (first author), who remained blinded to the treatment codes, using a stratified block design to achieve group balance in terms of age (6–11 months, 12–23 months and 24–35 months) and baseline haemoglobin concentration (above and below median, calculated for each cluster of enrolment separately; i.e. median Hb = 9.8, 9.5 and 9.4 g/dL for cluster 1, 2 and 3, respectively) at the pre-enrolment day (Day 0). Within each of the 6 resulting strata, children were randomly assigned to one of the three study treatment arms (1:1:1) to IHAT, FeSO_4_ or placebo using R version 3.4.3 and a block randomisation approach with fixed block size of six. After randomisation, the allocation list was kept electronically, with access granted to an independent clinician/nurse at the Basse site. A paper copy of the allocation list was kept securely in the Sponsor's office.

### Blinding

All participants and the entire study team, including lab staff, outcome assessors and database analysts were blinded to treatment group. Each treatment dose (iron compounds and placebo) was encapsulated in identical capsules (also containing powders of identical colour) by Capsugel-Lonza (Ploermel, France) and the supply of capsules for each child was packed in one bottle, individually labelled with the randomisation number/study ID for each child. If the DSMB had deemed necessary to perform emergency unblinding, only the particular study subject in question would have been unblinded, since each participant has a unique treatment code.

### Procedures

We screened and enrolled children in 3 clusters that were run sequentially (i.e. starting in Jan, May or August 2018). If the enrolment criteria were met, then a small venous blood sample was collected by the study nurse to send to the laboratory for confirmation of Hb levels and determination of serum ferritin. If 7≤ Hb < 11 g/dL and serum ferritin <30 μg/L, the child was invited to a pre-enrolment day back at the clinic (Day 0), 4–5 weeks after the screening visit, for a finger prick to confirm absence of malaria by RDT and to remeasure haemoglobin concentration. Those confirmed eligible were then randomised and enrolled in the study.

On study Day 1, venous blood and stool samples were collected (baseline samples), demographic and immunisation data were collected and a morbidity questionnaire was completed (full details in Supplementary Data S4). Each arm included an intervention period of 85 days plus an additional active follow-up period of 4 weeks post intervention (until Day 113). Highly trained and experienced field workers visited all children every day during the 85 day supplementation period in order to administer the iron supplements or placebo. Every week, at the study health facilities children's wellbeing was checked by the study nurses and they had a finger prick capillary blood collection to determine their malaria and Hb status. On Days 15 and 85, another stool sample and another venous blood sample were collected.

Any child found to have Hb below 7 g/dL during the follow-up study period stopped the study supplementation and was referred to the health centre for management and provided with standard iron syrup for 3 consecutive months according to national and WHO guidelines. These children continued to be followed up for adverse events by the field team without receiving any of the study treatments.

The final study visit was on day 113 (in the post-intervention follow-up period) when a finger prick blood sample was collected and those children that remained anaemic were given standard iron syrup for 3 consecutive months according to the national and WHO guidelines.

### Interventions

The IHAT powder clinical batch was manufactured under cGMP ICH Q7 rules by Sterling Pharma Solutions (Dudley Cramlington, UK). Pharmaceutical grade ferrous sulphate monohydrate and sucrose (placebo) were sourced by Capsugel-Lonza.

In line with the WHO and Gambian guidelines for iron supplementation in this age group the ferrous sulphate dose was 12.5 mg elemental Fe (38 mg ferrous sulphate monohydrate) once a day. The iron dose for IHAT was the anticipated dose with comparable iron-bioavailability: 20 mg elemental Fe (equivalent to 74 mg IHAT powder), determined taking into account IHAT's relative bioavailability to ferrous sulphate based on data from single-dose iron bioavailability studies in adults (see full protocol in Supplementary Data S1).

### Study objectives and outcomes

The study was a phase II trial designed to investigate for the first time the efficacy and tolerability of daily supplementation with IHAT in young children. This involved investigation of four primary objectives:

1) non-inferiority of IHAT compared to ferrous sulphate in terms of correction of iron deficiency anaemia defined as achievement of either a normal Hb (≥11 g/dL) or an increase of at least 1 g/dL from baseline (day 1) to day 85 of iron supplementation. The primary efficacy endpoint was the composite of iron deficiency and haemoglobin level at day 85;
2) superiority of IHAT compared to ferrous sulphate in terms of incidence density of moderate-severe diarrhoea (i.e. number of new moderate-severe diarrhoea episodes per child over the 85 days intervention);
3) superiority of IHAT compared to ferrous sulphate in terms of prevalence of moderate-severe diarrhoea (i.e. proportion of children with at least one episode of moderate-severe diarrhoea over the 85 days intervention);
4) non-inferiority of IHAT compared to placebo in terms of prevalence of moderate-severe diarrhoea.

Secondary endpoints were faecal microbiome diversity and profile, abundance of enteric pathogens, faecal calprotectin, hospitalisation and morbidity, malaria infection, treatment failures (i.e. the number of children who had to stop the study because their Hb fell below 7 g/dL), the proportion of days a child had diarrhoea over the 85 days intervention period (‘longitudinal prevalence’ of diarrhoea), the proportion of days a child had moderate-severe diarrhoea over the 85 days period (‘longitudinal prevalence’ of moderate-severe diarrhoea), incidence density of bloody diarrhoea (i.e. the number of bloody diarrhoea episodes per child-month of observation), markers of systemic inflammation (serum CRP and AGP), and systemic markers of iron handling (hepcidin, sTfR, transferrin saturation and circulating non-transferrin bound iron - NTBI). Gut microbiome, hepcidin and NTBI will be reported separately.

sTfR and hepcidin were assessed at days 1 and 85 and all other outcome measures were assessed at days 1, 15 and 85.

### Sample size

The study was powered for the first objective: determining whether IHAT was non-inferior to FeSO_4_ on the day 85 response outcome. It was assumed based on prior evidence that the proportion of children who were responders with FeSO_4_ at day 85 would be 0.3.^14^, ^15^, ^16^ The non-inferiority margin was an odds ratio of 0.583 (equivalent to a 0.1 absolute difference in response probability). A Cochrane review of iron supplementation in children in malaria-endemic areas showed that the magnitude of the effect on anaemia prevalence of providing iron supplementation is very heterogeneous with a RR for anaemia at the end of treatment of 0.63 (95% CI 0.49 to 0.82; 15 trials, 3784 children^17^), given this wide variability of response to oral iron supplementation in these settings, we believe the 0.1 absolute difference in response probability is clinically acceptable as non-inferiority margin. As any statistically significant result would be tested in a subsequent pivotal (Phase III) study, a 10% one-sided type I error rate was used. Assuming test statistics for each primary hypothesis are independent and all null hypotheses are true, the family-wise error rate would be approximately 0.35 based on formula 1−(1−0.1)^4^. A sample size of 200 per arm provides 89% power to demonstrate non-inferiority when the two arms have the same response probability.

As described further in the protocol, the sample size of 200 per arm also provides: 1) 90% power (10% one-sided type I error rate) for testing superiority of IHAT over FeSO_4_ for prevalence of diarrhoea when prevalence is 0.15 in IHAT arm and 0.25 in FeSO_4_ arm; 2) 93% power (10% one-sided type I error rate) for testing non-inferiority of IHAT vs placebo for diarrhoea prevalence when it is 0.15 in the IHAT and placebo arms with a 0.1 absolute non-inferiority margin; 3) 90% power (10% one-sided type I error rate) to find a reduction in incidence density of diarrhoea in IHAT vs FeSO_4_ assuming 1.28 episodes per child over the 85 days in the FeSO_4_ arm and rate ratio of 0.8.

To account for an anticipated 15% non-completion rate, based on previous studies in The Gambia, the target sample size was set to 705. As this was a phase II trial aiming to determine whether a phase III trial was warranted, no adjustment for multiple testing was made.

### Statistical analysis

The statistical analysis was fully pre-specified in a statistical analysis plan prior to database lock (see Supplementary Data S3). Two analysis populations were specified. The intention-to-treat (ITT) population was defined as all randomised participants, regardless of when they withdrew from the study, or whether they fully adhered to the treatment or switched to an alternative treatment. ITT participants were analysed according to which group they were randomised. The per-protocol (PP) population was defined as all randomised participants who completed the study protocol without any major protocol deviations, having received their assigned treatment for at least 80% of the planned study period. This PP population only includes the subset of the ITT population who have^1^ not missed more than 20% of the assigned study supplement daily doses from Day 1 to Day 85 (i.e. not missed more than 17 days of supplementation),^2^ have provided study samples for the relevant study timepoints and^3^ have not had any major protocol deviations (i.e. children randomised outside eligibility criteria and those protocol deviations which could have impacted on participant safety or on the scientific credibility of the trial).

We defined iron deficiency as a ferritin concentration below 12 μg/L, with ferritin values adjusted for inflammation for all children with elevated C-reactive protein (CRP >0.1 mg/L) and/or elevated α-1-acid-glycoprotein (AGP >0.59 g/L) using the regression model recommended by the Biomarkers Reflecting Inflammation and Nutritional Determinants of Anemia (BRINDA) group^18^ (full details in Supplementary Data S2).

All comparative analyses for the primary and secondary outcomes were based on generalised linear models (GLMs). Logistic, Poisson and linear regressions were used for binary, count and continuous outcomes respectively. From these models, a suitable between-group difference was estimated together with two-sided 80% confidence interval (CI) and p-value for superiority as appropriate. At the request of the reviewer, p-values are not quoted for the non-inferiority assessments and interpretation is in the context of the 80% CIs. Odds ratio (OR), rate ratio (RR) and mean difference were used for the three GLMs respectively. All models were adjusted for the randomisation strata (Hb and age group).

Analyses were all conducted in ITT and PP populations; the primary analysis population was ITT for the superiority analyses and PP for the non-inferiority analyses. As pre-specified in the Statistical Analysis Plan (Supplementary Data S3) we show in the main analysis both the PP and ITT analysis for the non-inferiority comparisons and the ITT analysis for the superiority comparisons; the remaining analysis are shown in Supplementary Data.

It had been planned to use multiple imputation (MI) if the proportion of children with missing baseline covariate data was above 5% (for the main baseline covariates of Hb and age), this was not the case so we present complete-cases (CC) ITT analysis for the primary analyses and MI-ITT results are available in Supplementary Data.

For the primary endpoints, confidence intervals are two-sided and reported p-values are one-sided, with values below 0.1 treated as statistically significant. For the secondary endpoints, reported p-values are two-sided.

All trial data was entered in the study database established using the Research Electronic Data Capture (REDCap) secure web-based application based in PHP and MySQL, and data was managed as detailed in the published study protocol.^12^

R/RStudio version 4.0.5^19^ and Stata 16^20^ were used to perform all data analyses and management.

### Role of the funding source

The funder of the study had no role in study design, data collection, data analysis, data interpretation, writing of the report, or in the decision to submit the paper for publication.

## Results

### Participants

**Fig. 1:**
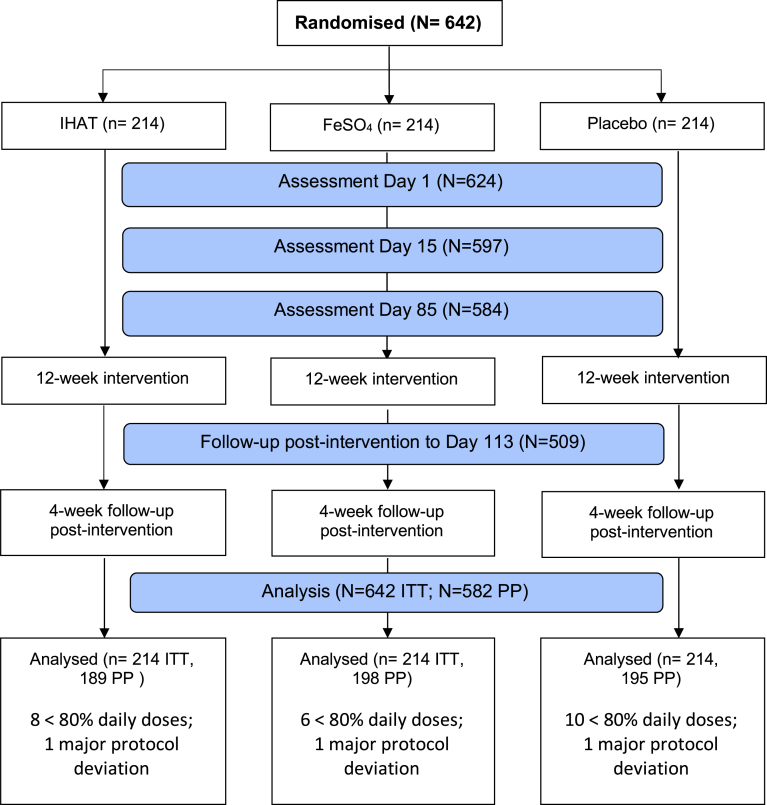
**Trial profile**. ITT = intention-to-treat; PP = per-protocol; IHAT = iron hydroxide adipate tartrate; FeSO_4_ = ferrous sulphate.

The trial was conducted from November 2017 to November 2018. A total of 1494 participants were screened and 642 participants met the eligibility criteria for entry into the trial and were randomised after the day 0 visit (n = 214 per study group) and constituted the intention-to-treat population (Fig. 1). The per-protocol population included 582 children who completed the day 85 follow-up, with >80% study supplementation doses and without major protocol deviations: 189 (88.3%) in the IHAT group, 198 (92.5%) in the FeSO_4_ group and 195 (91.1%) in the placebo group (Fig. 1).

The characteristics of the children at enrolment were balanced between groups, except for gender (Table 1). A total of 624 (97.2%) attended the day 1 baseline visit, and 584 (91.0%) participants attended the day 85 study visit and contributed outcome data, which was well balanced across the three arms. Overall, 37 (5.8%) children withdrew from the study (15 in the IHAT group, 10 in the FeSO_4_ group and 12 in the placebo group); withdrawals were not treatment related. Adherence to allocated treatment was high, with 580 (90.3%) of participants fully adhering to treatment with all daily doses (Supplementary Table S1). A total of 637 of the 642 randomised children had at least one dose of their allocated treatment. The number of participants with missing outcome data per treatment group is presented in Supplementary Table S2.

**Table 1:** Characteristics of randomised study participants.

Variable		ITT Population			PP Population		
		IHAT (N = 214)	FeSO_4_ (N = 214)	Placebo (N = 214)	(N = 189)	FeSO_4_ (N = 198)	Placebo (N = 195)
Age (months), median (IQR)		22 (15, 29)	22 (15, 30)	21.5 (15, 29)	22 (15, 29)	22 (16, 30)	21 (15, 29)
Weight (kg), mean (SD)		10.0 (2.0)	10.0 (1.5)	10.0 (1.7)	10.1 (5.2)	9.5 (2.1)	9.8 (5.5)
Height (cm), mean (SD)		81.6 (6.0)	81.5 (6.2)	81.6 (6.4)	79.8 (8.6)	78.6 (13.4)	76.6 (16.7)
WAZ, mean (SD)		−0.9 (0.8)	−1.0 (0.9)	−1.0 (0.9)	−0.9 (0.9)	−1 (0.9)	−1 (0.9)
HAZ, mean (SD)		−0.8 (0.9)	−0.9 (1.0)	−0.9 (1.0)	−0.8 (0.9)	−0.9 (1.0)	−0.9 (1.0)
WHZ, mean (SD)		−0.7 (0.8)	−0.7 (0.9)	−0.9 (0.8)	−0.7 (0.9)	−0.7 (0.9)	−0.9 (0.9)
Hb (g/dl), mean (SD)		9.4 (1.0)	9.4 (1.0)	9.4 (1.0)	9.4 (0.9)	9.5 (0.9)	9.5 (1.0)
Ferritin (μg/L), mean (SD)		13.0 (11.5)	13.0 (16.1)	14.2 (23.1)	13.1 (11.8)	13.2 (16.6)	13 (15.1)
Age group, n (%)	6–11mo	24 (11.2)	26 (12.1)	24 (11.2)	20 (10.6)	25 (12.6)	23 (11.8)
	12–23mo	96 (44.9)	93 (43.5)	96 (44.9)	85 (45)	83 (41.9)	87 (44.6)
	24–35mo	94 (43.9)	95 (44.4)	94 (43.9)	84 (44.4)	90 (45.5)	85 (43.6)
Gender, n (%)	Female	120 (56.1)	96 (44.9)	120 (56.1)	107 (56.6)	92 (46.5)	86 (44.1)
	Male	94 (43.9)	118 (55.1)	94 (43.9)	82 (43.4)	106 (53.5)	109 (55.9)
Hb group, n (%)	Low	103 (48.1)	101 (47.2)	103(48.1)	91 (48.2)	92 (46.5)	91 (46.7)
	High	111 (51.9)	113 (52.8)	111(51.9)	98 (51.9)	106 (53.5)	104 (53.3)
Health facility allocation, n (%)	Yorrobawol	72 (33.6)	70 (32.7)	72(33.6)	70 (37)	64 (32.3)	59 (30.3)
	Taibatu	60 (28)	63 (29.4)	60(28)	52 (27.5)	61 (30.8)	59 (30.3)
	Darsilami	35 (16.4)	36 (16.8)	35(16.4)	31 (16.4)	34 (17.2)	36 (18.5)
	Kuwonkuba	18 (8.4)	22 (10.3)	18(8.4)	15 (7.9)	20 (10.1)	15 (7.7)
	ChamoiBunda	29 (13.6)	23 (10.7)	29(13.6)	21 (11.1)	19 (9.6)	26 (13.3)

HAZ: height-for-age Z score, WAZ: weight-for-age Z score, WHZ: weight-for-height Z score, Hb: haemoglobin, SD: standard deviation. Hb group defined as: Low (below median Hb), High (equal to/above the median Hb) for each enrolled group.

### Primary outcomes

Both iron supplement formulations were equally effective at correcting IDA in comparison with the placebo group. IDA treatment response was achieved in 28.2% of children in the IHAT group, 22.1% of children in the FeSO_4_ group and 1.1% of children in the placebo group (PP population, Table 2). Diarrhoea incidence was higher in the FeSO_4_ group than in the IHAT group and IHAT was non-inferior to placebo in terms of diarrhoea prevalence (Table 2).

**Table 2:** Summary of primary outcomes.

Outcome	Population N=Complete Case	Parameter	Group		
			IHAT	FeSO_4_	Placebo
**IDA correction/response probability**					
	CC_PP	n (%)	50/177 (28.2)	42/190 (22.1)	2/186 (1.1)
	N = 553	OR (80% CI)- Crude	1.39 (1.02, 1.89)	Reference	NR
		OR (80% CI)- Adjusted^a^	1.39 (1.01, 1.91)	Reference	NR
	CC_ITT	n (%)	52/180 (28.9)	42/192 (21.9)	2/191 (1)
	N = 563	OR (80% CI)- Crude	1.45 (1.07, 1.97)	Reference	NR
		OR (80% CI)- Adjusted^a^	1.46 (1.07, 1.99)	Reference	NR
**Incidence density of moderate-severe diarrhoea**					
	CC_ITT	Episodes/child	2.66	3.42	3.02
	N = 584	RR (80% CI)- Crude	0.78 (0.6, 1.01)	Reference	NR
		RR (80% CI)- Adjusted^a^	0.76 (0.59, 0.99)	Reference	NR
		P-value for Superiority	0.0908		
**Prevalence of moderate-severe diarrhoea**					
	PP	n (%)	40/189 (21.2)	47/198 (23.7)	40/195 (20.5)
	N = 582	OR (80% CI)- Crude	Reference	1.16 (0.85, 1.59)	0.96 (0.70, 1.33)
		OR (80% CI)- Adjusted^a^	Reference	1.18 (0.86, 1.62)	0.96 (0.70, 1.33)
		P-value for Superiority		0.2494	NR
	CC_ITT	n (%)	39/190 (20.5)	46/196 (23.5)	42/198 (21.2)
	N = 584	OR (80% CI)- Crude	Reference	1.19 (0.87, 1.63)	1.04 (0.76, 1.44)
		OR (80% CI)- Adjusted^a^	Reference	1.22 (0.89, 1.68)	1.05 (0.76, 1.45)
		P-value for Superiority		0.2104	NR

P-values for superiority are one-sided for the primary endpoints. P-values for NI are not reported at the request of the reviewer and interpretation is in the context of the 80% CI.

PP – per-protocol, ITT – intention-to-treat; CC – complete case; NI – non-inferiority; NR – not reported as not pre-specified analysis; OR – odds-ratio; RR – rate ratio.

a Adjusted for age and Hb groups.

### Non-inferiority of IHAT vs FeSO_4_ for IDA correction

IHAT was non-inferior compared to FeSO_4_ for IDA correction both in the PP and ITT populations (Table 2 and Supplementary Table S3). The adjusted odds-ratio for IHAT vs FeSO_4_ was 1.39 (80% CI 1.01–1.91) in the PP population, which was consistent in the CC-ITT population (OR 1.46, 80% CI 1.07–1.99).

### Incidence density of moderate-severe diarrhoea

IHAT showed lower incidence of moderate-to-severe diarrhoea than FeSO_4_ in the CC-ITT population (RR_IHAT/FeSO4_ 0.76, 80% CI 0.59–0.99) (Table 2). This result was consistent in the PP population (RR_IHAT/FeSO4_ 0.77, 80% CI 0.6–1.0) (Supplementary Table S3).

### Prevalence of moderate-severe diarrhoea

The difference between the IHAT and FeSO_4_ groups for prevalence of diarrhoea did not reach statistical significance, either in the CC-ITT population (OR_FeSO4/IHAT_ 1.22, 80% CI 0.89–1.68) or the PP population (OR_FeSO4/IHAT_ 1.18, 80% CI 0.86–1.62) (Table 2). Non-inferiority of IHAT vs placebo was shown for the diarrhoea prevalence outcome in both the PP population (OR_placebo/IHAT_ 0.96, 80% CI 0.70–1.33) and the CC-ITT population (OR_placebo/IHAT_ 1.05, 0.76–1.45) (Table 2). MI-ITT results are consistent and presented in Supplementary Table S3.

### Secondary outcomes

There were no statistically significant differences between the IHAT and the FeSO_4_ group for any of the secondary outcome measures but there was a trend towards lower prevalence of ID and IDA in the IHAT group compared to the FeSO_4_ group (P < 0.1). The IHAT group had statistically significant less anaemia, iron deficiency and iron deficiency anaemia after the 3 months of supplementation than the placebo group (P < 0.0001) (Table 3). The rates of hospitalisation, malaria, acute respiratory infection and fever were similar between the 3 treatment groups (Table 3). Inflammation markers, systemic and gut specific, were also similar between the groups. Incidence of diarrhoea, calculated as number of diarrhoea episodes per child-month of follow-up, was similar between the treatment groups (Table 3). Iron status and inflammatory markers descriptive data are presented in Supplementary Data S5 (Table S4).

**Table 3:** Summary of secondary outcomes after 85 days of follow-up.

Outcome	Group			OR^a^ (80% CI), P-value	
	IHAT (n = 214)	FeSO_4_ (n = 214)	Placebo (n = 214)	Placebo vs IHAT	FeSO_4_ vs IHAT
***Iron status (n (%))***					
sTfR-Ft index^b^ sTfR/log_10_ferritin >2	180 (84.1)	193 (90.2)	170 (79.4)	0.56 (0.24,1.26), P = 0.18	2.17 (0.7,6.7), P = 0.19
IDA prevalence as per WHO (Hb < 11 g/dL and Ferritin_unadjusted_ < 30 μg/L)	80 (37.4)	98 (45.8)	150 (70.1)	8.92 (6.28,12.65), P < 0.0001	1.36 (1.04,1.79), P = 0.074
Low MCV (<75 fl)	169 (79.0)	178 (83.2)	182 (85)	2.82 (1.49,5.34), P = 0.019	1.15 (0.7,1.9), P = 0.36
Low MCH (<25 pg)	151 (70.6)	162 (75.7)	177 (82.7)	3.7 (2.3,5.94), P < 0.0001	1.2 (0.84,1.73), P = 0.26
High sTfR (>8.3 mg/L)	15 (7.0)	22 (10.3)	72 (33.6)	10.59 (6.9,16.24), P < 0.0001	1.46 (0.92,2.33), P = 0.15
Anaemia (Hb < 11 g/dL)	138 (64.5)	145 (67.8)	180 (84.1)	8.04 (4.79,13.47), P < 0.0001	1.06 (0.77,1.44), P = 0.41
Iron deficiency prevalence as per WHO (Ferritin_unadjusted_ < 30 μg/L)^c^	104 (48.6)	124 (57.9)	161 (75.2)	8.47 (5.71,12.56), P < 0.0001	1.36 (1.03,1.79), P = 0.078
Iron deficiency prevalence as per inflammation-adjusted ferritin (Ferritin_adjusted_ <12 μg/L)^d^	102 (47.7)	123 (57.5)	162 (75.7)	8.68 (5.85, 12.88), P < 0.0001	1.37 (1.05, 1.8),P = 0.065
***Safety (n (%))***					
Treatment failures (i.e. the number of children who have to stop study supplementation because their Hb fell below 7 g/dL)	1 (0.5)	0 (0)	1 (0.5)	NR^e^	NR^e^
Hospitalisation	1 (0.5)	2 (0.9)	2 (0.9)	1.93 (0.25,14.7), P = 0.59	1.98 (0.26,15.1), P = 0.58
Malaria infection	0 (0)	0 (0)	2 (0.9)	NR^e^	NR^e^
Acute Respiratory Infection (ARI)	89 (41.6)	83 (38.8)	91 (42.5)	1.03 (0.75,1.43), P = 0.87	0.89 (0.64,1.24), P = 0.56
Fever	12 (5.6)	15 (7.0)	15 (7.0)	1.29 (0.77,2.16), P = 0.53	1.29 (0.77,2.16), P = 0.53
**Tolerability (Diarrhoea), Mean (min, max)**					
The proportion of days a child has diarrhoea over the intervention period (‘longitudinal prevalence’ of diarrhoea)^f^	1.36 (0–15.56)	1.68 (0–18.82)	1.44 (0–20)	1.01 (0.76,1.33), P = 0.97	1.16 (0.88,1.53), P = 0.49
The proportion of days a child has moderate-severe diarrhoea over the intervention period (‘longitudinal prevalence’ of moderate-severe diarrhoea)^f^	0.97 (0–15.56)	1.32 (0–16.25)	1.02 (0–15.29)	0.94 (0.69,1.28), P = 0.79	1.08 (0.79,1.46), P = 0.76
Incidence of bloody diarrhoea per 30 child days of follow-up	0.011	0.019	0.014	1.36 (0.67,2.76), P = 0.58	1.73 (0.88,3.41), P = 0.30
**Inflammation, Median(IQR)**					
High faecal calprotectin ≥300 μg/g (n (%))	78 (36.4)	73 (34.1)	64 (29.9)	0.73 (0.49,1.08), P = 0.18	0.89 (0.60,1.31), P = 0.62
				**GMR**^g^**(80% CI), P-value**	

Serum C-reactive protein (CRP, mg/l)	1.54 (0.55–4.82)	1.07 (0.51–3.58)	1.03 (0.45–2.94)	0.79 (0.64,0.96), P = 0.12	0.84 (0.69,1.02), P = 0.25
Serum alpha 1-acid glycoprotein (AGP, g/l)	1.13 (0.89–1.41)	1.11 (0.84–1.42)	1.05 (0.85–1.3)	0.92 (0.88,0.97), P = 0.042	0.96 (0.92,1.01), P = 0.3

Data shown for the ITT population.

a Adjusted for age and Hb groups; P-values are two-sided for the secondary endpoints.

b Normal iron status is defined as sTfR-Ft index ≤2.^21^

c As per WHO recommendations of Hb < 11 g/dL and Ferritin_unadjusted_ < 30 μg/L, for populations with high burden of infection and inflammation.^13^

d Ferritin was adjusted for inflammation using the BRINDA recommendation lnFerritin_adjusted_ = lnFerritin_observed_ − 0.19 (lnCRP_observed_ + 2.26) − 0.74 (lnAGP_observed_ + 0.52). Iron deficiency is defined as Ferritin_adjusted_ < 12 μg/L.^18^

e NR, not reported due to very small number of events, so model was not fitted.

f OR for outcome comparing duration (≥1 vs 0).

g GMR, Geometric Mean Ratio.

### Safety

Of the 722 adverse events, 469 were considered mild, 228 moderate and 25 severe (Table 4). The number of individual subjects with adverse events was 432 (67.8%). The maximum number of AEs per subject was 6 and the median duration for an AE was 5 days. The majority of the AEs (65.8%) were considered as possibly related to the trial procedure by the study clinical team, these events were mainly acute respiratory infection or diarrhoea (Table 5). There were more cases of diarrhoea with no dehydration and infected skin lesions in the FeSO_4_ group in comparison with the placebo and the IHAT groups (Table 5). The rates of other AEs were similar between the 3 groups (Table 5).

**Table 4:** Summary of all adverse events by treatment arm.

Adverse events	Safety population^a^			Overall (N = 637)
	IHAT (n = 211)	FeSO_4_ (n = 212)	Placebo (n = 214)	
**Number of AEs reported**	225	251	246	722
**Number of subjects with AEs, N(%)**^b^	143 (67.8)	146 (68.9)	143 (66.8)	432 (67.8)
**Duration of AEs in days (Median, Min-Max)**	5 (1–12)	5 (1–24)	5 (1–16)	5 (1–24)
**Maximum AEs number per subject**	5	6	5	6
**Number of SAEs reported**	1	2	2	5
**SAEs leading to treatment discontinuation**	0	1	1	2
**Number of subjects with SAEs, N(%)**^b^	1 (0.5)	2 (0.9)	2 (0.9)	5 (0.8)
**Deaths, N(%)**	0	1 (0.5)	0	1
**Number of AEs by severity**^d^**, N (%)**				
Mild	151 (67.1)	163 (64.9)	155 (63.0)	469 (65.0)
Moderate	66 (29.3)	79 (31.5)	83 (33.7)	228 (31.6)
Severe	8 (3.6)	9 (3.6)	8 (3.3)	25 (3.5)
Life-threatening	0	0	0	0
**Subjects with AEs by severity**^c^^,^^e^				
Mild	84 (58.7)	79 (54.1)	78 (54.5)	241 (55.8)
Moderate	52 (36.4)	59 (40.4)	57 (39.9)	168 (38.9)
Severe	7 (4.9)	8 (5.5)	8 (5.6)	23 (5.3)
Life-threatening	0	0	0	0
**CAUSALITY**				
**Number of AEs by possible relatedness to treatment**^d^**, N (%)**				
Definitely unrelated	11 (4.9)	22 (8.8)	15 (6.1)	48 (6.6)
Unlikely	61 (27.1)	78 (31.1)	60 (24.4)	199 (27.6)
Possible	153 (68.0)	151 (60.2)	171 (69.5)	475 (65.8)
Probable	0	0	0	0
Definitely related	0	0	0	0
**Subjects with AEs by possible relatedness to treatment**^c^^,^^e^				
Definitely unrelated	7 (4.9)	9 (6.2)	6 (4.2)	22 (5.1)
Unlikely	27 (18.9)	32 (21.9)	23 (16.1)	82 (19)
Possible	109 (76.2)	105 (71.9)	114 (79.7)	328 (75.9)
Probable	0	0	0	0
Definitely related	0	0	0	0

a The pre-specified safety population in this study included all children that were randomised (Supplementary Table S6); only 3 children randomised to the IHAT group and 2 children randomised to the FeSO_4_ group did not receive at least one dose of their allocated treatment; none of these 5 children had adverse events reported during the study, but they are excluded from the safety population analysis presented here.

b Subjects are counted only once if they experience more than one AE or SAE.

c Subjects are counted only once if they experience more than one AE/SAE within a particular severity grade or relatedness category.

d Percentages are based on total number of AEs reported in each group.

e Percentages are based on total number of subjects with AEs reported in each group.

**Table 5:** Summary of number of adverse events by main diagnosis in each treatment arm.

AE diagnosis	Safety population^a^			Overall
	IHAT n (%)^b^	FeSO_4_ n (%)^b^	Placebo n (%)^b^	
Abdominal pain	2 (33.3)	0 (0)	4 (66.7)	6 (100)
Abscesses/soft tissue infections	7 (26.9)	9 (34.6)	10 (38.5)	26 (100)
Acute ear infection	7 (38.9)	6 (33.3)	5 (27.8)	18 (100)
Acute respiratory infection	117 (33.0)	110 (31.0)	128 (36.1)	355 (100)
Burns	2 (33.3)	1 (16.7)	3 (50.0)	6 (100)
Chicken pox	0 (0)	1 (100)	0 (0)	1 (100)
Conjunctivitis	1 (8.3)	5 (41.7)	6 (50.0)	12 (100)
Diarrhoea with no dehydration	29 (27.9)	44 (42.3)	31 (29.8)	104 (100)
Diarrhoea with some dehydration	1 (33.3)	1 (33.3)	1 (33.3)	3 (100)
Dysentery	5 (31.2)	6 (37.5)	5 (31.2)	16 (100)
Fever	12 (36.4)	13 (39.4)	8 (24.2)	33 (100)
Infected skin lesions	17 (29.3)	26 (44.8)	15 (25.9)	58 (100)
Malaria	0 (0)	1 (25.0)	3 (75.0)	4 (100)
Oral sores/gingivitis	2 (40.0)	1 (20.0)	2 (40.0)	5 (100)
Persistent diarrhoea	0 (0)	0 (0)	3 (100)	3 (100)
Poor appetite	0 (0)	1 (50.0)	1 (50.0)	2 (100)
Skin lesions	10 (31.2)	13 (40.6)	9 (28.1)	32 (100)
Trauma	0 (0)	0 (0)	2 (100)	2 (100)
Urinary Tract Infection (UTI)	1 (20.0)	2 (40.0)	2 (40.0)	5 (100)
Vomiting	7 (50.0)	2 (14.3)	5 (35.7)	14 (100)
Worms	2 (25.0)	5 (62.5)	1 (12.5)	8 (100)
Other	3 (33.3)	4 (44.4)	2 (22.2)	9 (100)

a The pre-specified safety population in this study included all children that were randomised (Supplementary Table S6 and S7); only 3 children randomised to the IHAT group and 2 children randomised to the FeSO_4_ group did not receive at least one dose of their allocated treatment; none of these 5 children had adverse events reported during the study, but they are excluded from the safety population analysis presented here.

b Number of events of a particular diagnosis in each group and percentage in relation to total number of adverse events of that particular diagnostics overall.

There were five SAEs reported during the study (Supplementary Table S5), one on the IHAT arm, two on the FeSO_4_ arm and two on the placebo arm. One of the five SAEs (severe respiratory distress due to peanut powder inhalation) resulted in death and was judged to be definitely unrelated to study arm (FeSO_4_). The other four SAEs resulted in complete recovery.

## Discussion

There are many forms of iron supplements and fortificants available for preventive and therapeutic intervention against iron deficiency and its consequent anaemia in children and pregnant women.

The more complex formulations, designed for improved tolerability and efficacy, are prohibitively expensive for mass administration in low-income countries and, anyway, their effectiveness is generally not known. Hence, national programmes supported by WHO and aid organisations such as UNICEF generally employ cheap ferrous salts (sulphate, gluconate, fumarate). Despite being relatively well absorbed these non-physiological ‘chemical’ forms of iron have limited efficacy in young children,^15^^,^^16^^,^^22^ may cause gut dysbiosis,^23^ are poorly tolerated in pregnancy,^24^ and have been associated with serious side effects in infancy.^5^

IHAT, the compound tested in this trial, is a novel formulation engineered to mimic the iron form of natural plant ferritin. The novel aspects of IHAT are that it is not a soluble compound nor does it require solubilisation in the stomach prior to uptake: instead it is taken up as whole nanoparticles by the duodenal enterocytes via endocytosis,^10^^,^^11^ a natural absorption pathway for both dietary plant ferritin and digested ferric iron.^25^ This means that the unabsorbed fraction of the compound transiting to the lower gut, usually at least 70% of all ingested iron irrespective of the form, will remain nanoparticulate and insoluble, and as such should not be available to promote significant pathogen growth, tissue inflammation or other iron redox associated issues.^26^^,^^27^

A crucial aspect of the IHAT nanostructure is that once it has entered the enterocyte it is sufficiently labile to break down inside the lysosomes/endosomes and deliver its iron because the native iron oxo-hydroxide structure (i.e. ferrihydrite) has been intentionally destabilised by the incorporation of tartaric and adipic acids,^10^^,^^11^^,^^28^ in much the same way as occurs with the ferritin iron core which interacts with phosphate and amino-acid residues of the ferritin protein shell.^29^ Our pre-clinical and early-clinical data indicate that IHAT is effectively absorbed in humans, corrects IDA in animal models, is not redox reactive and does not have a detrimental impact on the gut microbiome.^11^^,^^26^, ^27^, ^28^ Recently published results for the gut microbiome analysis (secondary endpoint) in the IHAT-GUT study have confirmed that IHAT does not impact negatively the microbiome.^30^

The IHAT-GUT study has shown that a 3 month regime of daily IHAT administration is non-inferior to the usual standard of care (ferrous sulphate) at correcting IDA in a population of moderately undernourished, iron deficient young Gambian children. IHAT was also non-inferior to placebo in terms of prevalence of moderate-severe diarrhoea. Importantly, IHAT was superior to FeSO_4_ in incidence density of moderate-severe diarrhoea, but results did not reach statistical significance for diarrhoea prevalence, although further scrutiny is worthwhile given the noticeable higher number of cases of diarrhoea reported as adverse events in the FeSO_4_ group (Table 5). Coupled with an effectiveness of IHAT that was nearly greater than ferrous sulphate (Table 2), further work is warranted.

Our finding of lower than 30% response after 3 months of oral iron supplementation in both iron groups is consistent with the existing evidence for this age group and setting.^14^, ^15^, ^16^ This supports the current WHO recommendation for 6 months or longer treatment to achieve the required improvement of IDA in this population group.^31^

To our knowledge, this is one of a limited number of RCTs conducted in sub-Saharan Africa in children with IDA which included a placebo group. Our study suggested that ‘doing nothing’ is not a suitable strategy and, despite access to basic healthcare, those children that did not receive iron had no improvement in their Hb levels and iron indices with fewer than 1% of children exhibiting haematological improvement or recovery.

Importantly, the presence of the placebo group allowed us to determine ‘background’ diarrhoea and other adverse events. A study in 6-month old Kenyan infants suggested that iron-containing multi-micronutrient powders (MNP) are associated with an increased risk of diarrhoea versus the no iron group; 27.3% of infants receiving 12.5mgFeMNP required treatment for diarrhoea versus 8.3% who received the MNP without added iron, P = 0.092.^32^ A recent systematic review of trials conducted in similar settings^17^ also reported that iron supplementation in young children may increase prevalence of diarrhoea by 15% (rate ratio 1.15, 95% CI 1.06 to 1.26; eight trials, 23,912 child-months) although this effect seems to be driven by the iron-zinc micronutrient combination (rate ratio 1.29, 95% CI 1.15 to 1.44; three trials, 6346 children) rather than by iron supplementation alone (rate ratio 0.99, 95% CI 0.87 to 1.13; seven trials, 17,566 children). In our study the prevalence of diarrhoea in the placebo group did not show statistically significant differences between the two iron supplementation groups, possibly because we provided iron supplementation in isolation and not combined with zinc.

One limitation of this study is that since this is the first Phase II study with IHAT, where primary hypothesis were tested using a statistical significance level of 0.2 (one-sided), it does not permit definite conclusions of non-inferiority or superiority of IHAT in relation to clinical standard-of-care. We considered this study as an initial trial where results could inform the design and be tested in a subsequent pivotal Phase III study, which is currently in development.

Another limitation of this study was that the estimated dose with comparable iron-bioavailability for IHAT was determined based upon data of relative bioavailability to ferrous sulphate collected in adults, rather than in infants and young children, and in a single dose study rather than repeated dosing. Either of these factors could have led to an underestimate of the long term absorption of IHAT in infants and young children, even though we tried to correct for this by considering the upper 75% centile of the median RBV (i.e. 60%) determined in adult women.

A further potential limitation of the study was the use of multiple primary endpoints without adjusting for multiple testing. Since this was the first Phase II study with IHAT, we considered it an initial trial where results could be tested in a subsequent pivotal Phase III study. As such, in the present study, each primary hypothesis was tested at a 10% one-sided type I error rate. Nevertheless, the non-inferiority of IHAT vs ferrous sulphate in IDA correction was sufficiently shown with a statistically significant p-value in both the intention-to-treat and per-protocol populations, and this would have retained statistical significance even after adjusting for multiple testing with the 4 primary hypotheses.

The study strengths include its sample size and trial setting, to our knowledge this is the largest trial conducted with oral iron supplementation in rural Africa with young children which included a placebo arm allowing for testing safety signals due to the iron supplements, particularly in relation to diarrhoea and infection. Further strengths include the stratified randomisation by age and Hb level, double-blinding, placebo control, individual participant treatment allocation masking, high levels of adherence to the study intervention (580/642 children fully adhering to treatment with all 85 daily doses) and low attrition with 584/642 children contributing data for the primary outcomes. The trial population was highly representative of the type of children that should be targeted by public health interventions. Furthermore, we had consistent engagement between the trial staff and study communities throughout the study and any potential barriers and facilitators to conducting this trial were promptly identified and addressed.^33^ There were no differences in the primary outcome results between the per-protocol and the intention-to-treat analyses. There were no differences between the two iron supplementation groups for all the multiple iron status biomarkers analysed or for the different safety and inflammatory markers used. These strengths, along with the fact that this was a trial conducted to the highest international standards of good clinical practice in a resource-poor setting, with defined protocols and documentation of process, support the validity of this Phase II trial results.

The putative advantages of IHAT are that its (endocytic) absorption does not require divalent metal transporter-1 (DMT1) (the activity of which may be impaired by villous atrophy common in children in low income settings), that any unabsorbed iron is not available to potentially pathogenic gut bacteria, and that it is readily manufactured at low cost. The IHAT-GUT study has now shown that IHAT is as good as ferrous sulphate at correcting IDA in young children living in resource-poor settings without any safety signals or concerns in terms of gut inflammation. Moreover, our study suggested there could be lower incidence and prevalence of moderate-severe diarrhoea with IHAT supplementation as compared to ferrous sulphate, although this would need to be tested in a larger trial. We envisage that IHAT could become a novel iron source for use in micronutrient intervention strategies in resource-poor countries, particularly in individuals intolerant to ferrous iron salts, and in this way contribute to the reduction of the global burden of IDA. The next step will be to conduct a pivotal Phase III clinical trial in pregnant women and children. IHAT has been evaluated by the European Food Safety Authority and the European Commission and is approved as a novel form of iron in supplements for both adults and children in the EU market.

In conclusion, in the IHAT-GUT study daily supplementation with IHAT was safe, and effective, showing sufficient non-inferiority to standard-of-care (ferrous sulphate) with respect to treating iron deficiency anaemia in young children and with statistically significant lower incidence of moderate-severe diarrhoea. A definitive Phase III trial is now warranted.

## Contributors

AMP and DIAP acquired the funding for the study. JW, AMP and DIAP conceptualised the manuscript and study design. NIM, JW, AMP, and DIAP interpreted data and wrote the original draft and were responsible for the decision to publish. TM, NIM and DIAP have assessed and verified the data. SAN, OO, FC, BB, CS, ATJ, IH collected data and provided important input for the study protocol. IH and DIAP were project supervisors. NIM and JW analysed data. NF and JJP provided technical input on the IHAT compound and study protocol, and critically revised the manuscript for important intellectual content. All authors approved the final version of the manuscript to be published. All authors are accountable for all aspects of the work in ensuring that questions related to the accuracy or integrity of any part of the work are appropriately investigated and resolved.

## Data sharing statement

Study protocol and Statistical Analysis Plan are enclosed in the Supplementary Data. Research data and other material (eg, informed consent form) will be available to the scientific community, immediately on publication, with as few restrictions as possible. All requests should be submitted to the corresponding author for consideration.

## Declaration of interests

D.I.A.P., N.F. and J.J.P. are inventors of the IHAT iron supplementation technology, for which they could receive future awards to inventors through the MRC Awards to Inventor scheme. They are also scientific advisors to Nemysis Ltd, who now hold the license for IHAT. They have received honoraria or consultancy fees from some or all of: Vifor Pharma UK, Shield Therapeutics, Entia Ltd, Danone Nutricia, UN Food and Agriculture Organisation (FAO) and Nemysis Ltd, for work concerning iron in health. D.I.A.P. has since moved to full employment in the iron and health industry with Vifor Pharma UK but all work pertaining to this publication was conducted whilst at the University of Cambridge and MRC Unit The Gambia at LSHTM. Nemysis Ltd paid consulting fees to J.W.’s Institution. Notwithstanding, the authors declare no potential conflicts of interest with respect to the research, authorship, and/or publication of this article.
