# Supplementary material for: A novel nano-iron supplement versus standard treatment for iron deficiency anaemia in children 6–35 months (IHAT-GUT trial): a double-blind, randomised, placebo-controlled non-inferiority phase II trial in The Gambia
Source: eClinicalMedicine. 2023 Feb 9;56:101853. doi: 10.1016/j.eclinm.2023.101853 (PMC9985047; doi:10.1016/j.eclinm.2023.101853)
Supplement: Supplementary Data S1 [file mmc1.pdf]

CLINICAL TRIAL PROTOCOL

A novel nano-iron supplement (IHAT) to safely combat iron deficiency and anaemia (IDA) in young children: a double-blind randomised controlled trial

---

Protocol No:

**SCC No:**	**1489**
**Brief Title**	IHAT-Gut
**Other Number(s)**	
**Protocol Version – Date**	V5.0, 21 May 2018
**Sponsor**	London School of Hygiene & Tropical Medicine (LSHTM)<br>MRC Unit The Gambia at LSHTM<br>PO Box 273 Banjul,<br>The Gambia, West Africa
**Chief Investigator**	Professor Andrew Prentice
**Local Principal Investigator**	Dr Mohammad Ilias Hossain
**Co PI:**	Dr Dora Pereira (University of Cambridge)

Table of contents

Page
Signature page 2
Protocol amendment(s) 3
Key roles 6
List of abbreviations 8
Protocol summary 11
1 Background information and rationale 14
1.1 Background information 14
1.2 Currently used oral iron and why IHAT is novel and dietary-like 16
1.3 Rationale 19
1.4 Potential risks and benefits 25
1.4.1 Risk mitigation 26
2 Study objectives 29
2.1 Study endpoints 30
3 Study design 32
3.1 Type of study and design 32
3.2 Randomisation and blinding procedures 33
3.2.1 Randomisation 33
3.2.2 Blinding 35
3.3 Sub-studies 36
3.4 Investigational products 36
3.4.1 Description of products 36
3.4.2 Formulation, packaging and labelling 36
3.4.3 Product storage and stability 37
3.4.4 Dosage, preparation and administration of investigational products 37
3.4.5 Concomitant medications/treatments 38
4 Selection and withdrawal of participants 38
4.1 Selection of participants 38
4.2 Eligibility of participants 40
4.2.1 Inclusion criteria 40
4.2.2 Exclusion criteria 41
4.3 Withdrawal of participants 42
5 Study procedures and evaluations 42
5.1 Study schedule 42
5.1.1 Study sensitisation 42
5.1.2 Screening and enrolment (baseline) 43
5.1.3 Follow-up (study visits) 44
5.1.4 Final study visit 46
5.1.5 Early termination visit 47
5.2 Study evaluations 49

5.2.1 Clinical evaluations 49
5.2.2 Laboratory evaluations 49
6 Safety considerations 52
6.1 Methods and timing for assessing, recording, and analysing safety parameters 53
6.1.1 Adverse events 53
6.1.2 Reactogenicity 53
6.1.3 Serious adverse events (SAEs) 53
6.1.4 Assessment of intensity of AEs 54
6.1.5 Assessment of causality 54
6.1.6 Serious Adverse Reaction (SAR) 56
6.2 Reporting procedures 56
6.3 Safety oversight 57
7 Discontinuation criteria 57
8 Statistical considerations 59
8.1 Sample size determination 59
9 Data handling and record keeping 66
9.1 Data management and processing 66
9.2 Source documents and access to source data 67
9.3 Protocol deviations 68
10 Quality control and quality assurance 68
10.1 Study monitoring 69
11 Ethical considerations 69
11.1 General considerations on human subject protection 69
11.1.1 Rationale for participant selection 69
11.1.2 Rationale for use of a placebo group 72
11.1.3 Evaluation of risks and benefits 73
11.2 Informed consent 74
11.3 Participant confidentiality 74
11.4 Future use of stored specimen 75
12 Financing and insurance 75
13 Publication policy 75
14 References 75
Supplements, appendices and other documents 80
Appendix 1: Project Timeline 81
Appendix 2: Target Product Profile (TPP) 83

Key roles

For questions regarding this protocol, contact Dr Dora Pereira, Department of Pathology, University of Cambridge, UK, diap2@cam.ac.uk, 0044-1223764864.

**Author(s):**	Dr Dora Pereira (University of Cambridge, diap2@cam.ac.uk) and Professor Andrew Prentice (MRCG, andrew.prentice@lshtm.ac.uk)
**Sponsor's representative:**	Dr Jonas Lexow (MRCG, jlexow@mrc.gm)
**Chief Investigator:**	Professor Andrew Prentice (MRCG, andrew.prentice@lshtm.ac.uk)
**Principal Investigator(s):**	Local PI: Dr Mohammad Ilias Hossain (Paediatrician, MRCG Basse, mihossain@mrc.gm)<br>Co-PI: Dr Dora Pereira (University of Cambridge, diap2@cam.ac.uk)
**Sub-Investigator(s):**	Ebrima Sise MRCG Keneba (support for iron assays)<br>Thomas Mendy/Golam Sarwar MRCG Basse (study data manager/database developer)<br>Lady Chilel Sanyang MRCG Basse (laboratory assays)<br>Amadou Jallow MRCG Fajara and Keneba (laboratory assays)<br>Dr Davis Nwakanma MRCG (Head of Laboratory Services)<br>Bolarinde Lawal MRCG (Manager of Clinical Laboratories Fajara)<br>Nurudeen Ikumapayi MRCG Basse (laboratory manager, Analytical Project Manager)
**Trial Physicians:**	Dr Ogochukwu Ofordile, Study Research Clinician<br>Dr Mohammad Ilias Hossain, Local PI (Clinical Trial Coordinator)

**Sponsor Medical Expert:**	To be confirmed (we will consult Dr Muhammad Afolabi, Dr Ed Clark and other MRCG clinicians with paediatric expertise)
**Trial monitor(s):**	Sey Gibbi (MRCG Clinical Trials Support Office)
**Safety monitor(s):**	Local safety monitor (Dr Aderonke Odutola, Paediatrician)
**Chair of DSMB:**	Professor James Jay Berkley, Professor of Paediatric Infectious Diseases, KEMRI-Wellcome (Kilifi, Kenya)
**Chair of the TSC:**	Dr Margaret Pinder, MRCG Basse
**Statistician:**	Dr Nuredin Ibrahim Mohammed and Dr David Jeffries MRC Unit The Gambia at LSHTM (statistical support)<br>Dr James Wason MRC Biostatistics Unit, Cambridge (advisor for trial design, power calculation and primary outcome analysis, and member of TSC)
**Clinical Laboratory/ies:**	MRC Unit The Gambia at LSHTM, Keneba<br>MRC Unit The Gambia at LSHTM, Basse<br>MRC Unit The Gambia at LSHTM, Fajara
**Other institutions/ Collaborators:**	National Nutrition Agency [NaNA] (Modou Phall)<br>Wellcome Trust Sanger Institute, Cambridge, UK (Professor Julian Parkhill and Dr Josef Wagner)<br>King's College London, London, UK (Professor Robert Hider)
**Local Ethics Committee**	Gambia Government/MRC Joint Ethics Committee,<br>c/o MRC Unit The Gambia at LSHTM,<br>PO Box 273, Banjul, The Gambia, West Africa

List of abbreviations

ADME	Absorption, Distribution, Metabolism & Excretion
AE	Adverse Event
AGP	Alpha-1 acid Glycoprotein
API	Active Pharmaceutical Ingredient
BMGF	Bill and Melinda Gates Foundation
BSU	Biostatistics Unit
CI	Chief Investigator
CMO	Contract manufacturing Organisation
CRF	Case Report Form
CRP	C-reactive protein
DMT1	Divalent Metal Transporter 1
DSMB	Data & Safety Monitoring Board
EDTA	Ethylenediaminetetraacetic acid
FA	Field Assistant
FDA	Food and Drug Administration
FS	Field Supervisor
$FeSO_4$	Ferrous Sulphate
GCP	Good Clinical Practice
GMP	Good Manufacturing Practice
cGMP	Current Good Manufacturing Practice
GRAS	Generally Recognised as Safe
Hb	Haemoglobin
HNR	Human Nutrition Research (Unit)
ID	Iron Deficiency
IDA	Iron Deficiency Anaemia
ICH	International Conference on Harmonization

IEC	Independent Ethics Committee
IHAT	Iron Hydroxide Adipate Tartrate
IMP	Investigational Medicinal Product
LH	Lithium heparin
LSHTM	London School of Hygiene and Tropical Medicine
MHRA	Medicines and Healthcare products Regulatory Agency
MNP	Multimicronutrient Powder
MRC	Medical Research Council
MRCG	MRC Unit The Gambia at LSHTM
NaNa	National Nutrition Agency
NTA	Nitrilotriacetic acid
NTBI	Non-transferrin bound iron
PI	Principal Investigator
RBV	Relative Bioavailability Value
RCT	Randomised Controlled Trial
RDT	Rapid Diagnostic Test
RHT	Regional Health Team
SAE	Serious Adverse Event
SAR	Setious Adverse Reaction
SD	Standard Deviation
SFA	Senior Field Assistant
SO	Scientific Officer
SOP	Standard Operating Procedure
SSP	Study Specific Procedure
sTfR	Soluble transferrin receptor
STH	Soil-Transmitted Helminths
TSAT	Transferrin saturation
TSC	Trial Steering Committee

TPP	Target Product Profile
XRD	X-Ray Diffraction
WHO	World Health Organization

Protocol summary

**Title:**	A novel nano-iron supplement (IHAT) to safely combat iron deficiency and anaemia (IDA) in young children: a double-blind randomised controlled trial.
**Alias :**	IHAT-Gut
**Phase**:	Phase II
**Population:**	Young Children (6-35 mo.)
**Number of participants:**	705
**Number of Sites:**	1
**Location of Sites (including satellite sites):**	One site, Communities in Upper River Division (North Bank villages within a 25 km distance from Basse)
**Trial Duration:**	
**- Clinical Phase:**	9 months of study supplementation/intervention
**- Whole trial:**	11 months field data collection
**Duration for Participants:**	12 weeks intervention<br>+ 4 weeks AE/SAEs active follow-up post-intervention
**Description of Investigational Products:**	- 1 dose/day single IMP containing IHAT powder bioequivalent to 12.5 mg Fe (i.e. 20 mg Fe taking into account IHAT's relative bioavailability to $FeSO_4$)<br>- 1 dose/day single IMP containing $FeSO_4$ powder equivalent to 12.5 mg Fe<br>- 1 dose/day containing a placebo powder (no-iron, 'sugar' compound)
**Objectives:**	In this trial we will test the hypothesis that supplementation with IHAT eliminates iron deficiency and improves haemoglobin levels in young children without increasing infectious diarrhoea or promoting negative changes in the gut microbiome or inducing gut inflammation.<br>The **primary objective** for this trial is to show non-inferiority of IHAT in relation to ferrous sulphate at correcting

IDA, and in terms of diarrhoea to show superiority in relation to ferrous sulphate and non-inferiority in relation to placebo.

Secondary objectives are to show that IHAT is non-detrimental with respect to enteric pathogen burden, the gut microbiome, and intestinal inflammation.

Endpoints:

Primary endpoints:

There are 4 primary endpoints of the trial:

1. iron deficiency at 12 weeks
2. haemoglobin levels at 12 weeks
3. 'incidence density' of moderate-severe diarrhoea over the 12 weeks (i.e. the number of new moderate-severe diarrhoea episodes per child over the 12 weeks intervention)
4. 'period prevalence' of moderate-severe diarrhoea over the 12 weeks intervention period (i.e. the proportion of children with at least one episode of moderate-severe diarrhoea over the 12 weeks intervention)

To assess iron deficiency we will take into consideration the most up-to-date recommendation from WHO, who are currently conducting a consultation on this matter. The new WHO guidelines will include a recommendation for the best marker of iron deficiency in the context of inflammation and we expect this to be either using ferritin alone or the sTfR/logferritin index, where in both cases ferritin values will be inflammation-adjusted. More details on the possible methods to adjust ferritin values for inflammation are given in Section 2.1.

Iron deficiency and haemoglobin levels at 12 weeks will be used to define the prevalence of IDA which we will use to assess non-inferiority of IHAT relative to $FeSO_4$ in terms of efficacy at correcting IDA. We will determine the proportion of children in each arm which resolve iron deficiency **and** either achieve a normal Hb or an increase in Hb of at least 1 g/dL after the 12 weeks of the intervention.

	‘Incidence density’ and ‘period prevalence’ of moderate-severe diarrhoea will both be used to assess superiority of IHAT relative to $FeSO_4$ for the diarrhoea outcome and non-inferiority relative to placebo for the diarrhoea outcome (i.e. safety).<br>More details about the 4 study hypothesis and evaluations are provided in Sections 2.1 and 8.1.<br><br>Moderate-severe diarrhoea refers to those diarrhoea episodes where (i) the child passes more than 5 loose or liquid stools per day, (ii) there is blood or mucus in the stool (dysentery), or (iii) the child shows signs of clinical dehydration (assessed by the study nurse based on physical signs such as little or no urination, sunken eyes, and skin that lacks its normal elasticity).<br><br>**Secondary endpoints:**<br>Secondary endpoints will be faecal microbiome diversity and profile (particularly in terms of abundance of Enterobacteria), abundance of enteric pathogens, faecal calprotectin (marker of gut inflammation), hospitalisation and morbidity (data collected three times per week using the questionnaire developed in the HIGH study), malaria infection (data collected every week with RDT), treatment failures (i.e. the number of children who have to discontinue the study intervention because their Hb falls below 7 g/dL), the proportion of days a child has diarrhoea over the intervention period (‘longitudinal prevalence’), the proportion of days a child has moderate-severe diarrhoea over the intervention period (‘longitudinal prevalence’ of moderate-severe diarrhoea), ‘incidence density’ of bloody diarrhoea (i.e. the number of bloody diarrhoea episodes per child-month of observation), markers of systemic inflammation (serum CRP and AGP), and systemic markers of iron handling (hepcidin, transferrin saturation and circulating non-transferrin bound iron - NTBI).
**Description of Study Design:**	3-arm (IHAT, $FeSO_4$, placebo), parallel, randomised, double-blind, placebo-controlled clinical trial.

1 Background information and rationale

1.1 Background information

Iron deficiency (ID) and its associated anaemia (IDA) is the largest nutritional deficiency disorder in the world and affects a total of 1.2 billion people, the majority of whom are children and women from resource-poor countries. Iron deficiency is frequently exacerbated by concomitant parasitic and bacterial enteropathogenic infections and contributes to almost 0.8 million deaths a year as well as irreparably limiting the cognitive development of children and leading to poor outcomes in pregnancy (1). Currently available iron compounds are cheap and readily available but constitute an unphysiological approach to providing iron that leads to significant side effects and serious adverse events (2-5). Data and meta-analysis from trials involving nearly ten thousand young children in developing countries have consistently shown that conventional soluble oral iron supplementation used to treat IDA is associated with increased infection including bloody diarrhoea (3, 6-8) and detrimental changes to the gut microbiome and gut inflammation (8, 9), further increasing the burden from enteric infection and environmental enteropathy (i.e. persistent gut damage and inflammation that leads to malabsorption), which is a major cause of growth failure in children in resource-poor environments (10, 11). Consequently, ID and IDA remain without an effective cure and we have so far been unable to decrease the burden of this disease in children, even after considerable effort and investment in the past 20 years (Figure 1).

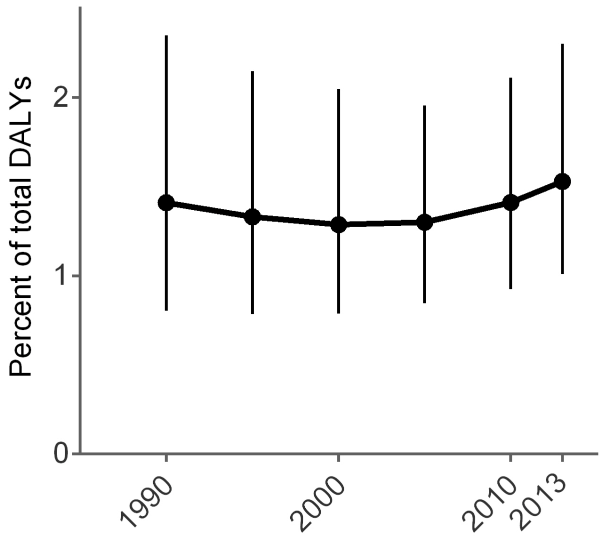

Figure 1. Global burden of iron deficiency anemia as a cause of disease in children under 5 y. Data is expressed as percent of disability adjusted life years (DALYs). Reference: Institute for Health Metrics and Evaluation (IHME). GBD Compare. Seattle, WA: IHME, University of Washington, 2015. Available from http://vizhub.healthdata.org/gbd-compare. (Accessed [13/01/2016])

The team at the MRC Human Nutrition Research Unit, have developed an innovative oral iron supplement (IHAT) for the safe treatment of iron deficiency and anaemia in resource-poor countries. Dr Pereira (who has been working at MRC HNR for the past 11 years) is a co-inventor of IHAT and is leading on its clinical development. As a next step in development we propose to conduct the first Phase II clinical trial with IHAT and obtain clinical data for its safety and efficacy in correcting IDA in young children. As mentioned above, many studies have shown that this is

the population group that suffers the most debilitating effects of current iron supplementation and, therefore, the group most at need of an alternative oral iron supplement that is safer, particularly in the gastrointestinal tract. Please refer to Section 11 (Ethical Considerations) for more details on our rationale for conducting this trial directly in children.

If IHAT is successful in the trial, these data will provide the evidence needed to encourage further investment so that IHAT can be implemented as a novel iron source for use in micronutrient intervention strategies aimed at children and women living in resource-poor countries and, hence, reduce the global burden of IDA.

The specific problem this study seeks to address relates to these serious side effects of oral iron supplements for treating anaemia in young children. Specifically, we will be addressing the fact that current iron supplements increase risk of infectious diarrhoea (3, 6-8) which promotes a pathogen-driven inflammatory response (8, 9, 12, 13) that impacts on hepcidin with reduction of iron absorption (14) and, therefore, offers limited benefit with added risk.

In the 21st century, we simply cannot accept the current view that increased risk of infectious diarrhoea in young children is normal collateral damage of oral iron supplementation in populations at high risk of enteric infection. This is a problem of current iron supplements that are all based on providing large bolus of non-physiological forms of ‘gut-reactive’ iron, that is soluble in the gut lumen, and to which humans have not previously been naturally exposed through diet. These harmful effects are caused by supplements that circumvent the natural chaperone and absorption/exclusion mechanisms of the gut.

We know that growth and virulence of bacterial enteropathogens is stimulated by these soluble iron supplements which are bioavailable to both the gut microbiome and the human host.

Conversely, our nano iron IHAT is effectively absorbed in humans without requiring solubilisation and, because it is not solubilised, it is not accessible as an iron source to bacterial enteropathogens or available to undergo significant redox cycling in the gut lumen (please refer to the summary of our pre-clinical and proof-of-concept data in Section 1.3 for details about IHAT’s absorption, safety and metabolism)**. This means that supplementation with IHAT should correct IDA without increasing the burden from infectious diarrhoea and should result in improved overall response to iron supplementation compared to conventional soluble iron. <u>In essence, our nano iron looks like dietary non-haem iron and should behave like dietary iron.</u>**

1.2 Currently used oral iron and why IHAT is novel and dietary-like

The current paradigm for iron delivery is that only soluble ionic iron can be efficiently absorbed through DMT1 present in the apical membrane of duodenal enterocytes. This paradigm has supported the development, and use, over the past 20 years of interventions that aim to deliver a large bolus of ionic iron to the enterocytes. Soluble ionic iron is not naturally present in the diet and, therefore, our bodies have only recently been exposed to significant amounts of this iron form through supplementation and fortification. Many different forms of iron have been tested, and are currently being used, but these all rely in the solubilisation of the compound in the stomach prior to the delivery of ionic iron, in the ferrous or ferric form, to the enterocyte cell.

Soluble ferrous iron (e.g. Fe(II) fumarate, Fe(II) bisglycinate) is redox reactive in the upper gastrointestinal tract and can redox cycle via Fenton chemistry promoting mucosal inflammation which leads to the acute side-effects normally reported with iron supplementation, such as heartburn, nausea and abdominal pain (15). Soluble ferric iron (e.g. Na Fe(III) EDTA, Fe(III) citrate, Fe(III) trimaltol, Fe(III) trisglycinate) is less redox reactive but soluble Fe(III) must still be reduced at the mucosal surface and may yield some 'sub-symptomatic' free radicals in the process, whilst the unabsorbed iron still delivers soluble iron to the colon where it is available to be utilized by pathogenic bacteria and to induce detrimental changes to the gut microbiome (8), and potentially increases risk of colorectal cancer (16-18). These effects may result in the distal and more chronic side effects of oral iron, namely diarrhoea or constipation. Furthermore, the high cost of ferric chelates limits their use in resource-poor countries. Haem iron supplements, such as heme iron polypeptide, are also expensive and there are marked concerns over haem safety in the colon in terms of catalizing the formation of N-nitroso compounds associated with cancer risk (19, 20). Other protein-bound forms of iron, such as lactoferrin and ferric mannitol ovoalbumin are several order of magnitude higher cost (~100 x), have limited shelf-life or are only useful for niche groups such as newborn babies.

Insoluble forms of iron, such as iron phosphates and elemental iron are not sufficiently bioavailable in humans to merit serious clinical use because they still require solubilisation in the stomach prior to absorption and the efficiency of this process is very low for these forms of iron. Ferric iron polymaltose is an insoluble iron compound made of aggregates of small iron oxo-hydroxide particles encased in a carbohydrate (polymaltose) coating. Even in the case these particles would be taken up whole by the enterocyte, they are too stable to be broken down and deliver enough iron for systemic use, hence their reported low bioavailability (21, 22).

Therefore, ferrous iron salts remain the oral iron compounds of choice because they are cheap and well absorbed and significant commercial effort and investment in the alternatives mentioned above has been unable to convince prescribers and governments that new oral iron preparations merit widespread use. Even in patients severely intolerant of standard oral iron, or in those where

risk outweighs benefit, the increasing second line preference is for intravenous (IV) iron, in Western countries, and for 'no iron', in resource-poor countries.

IHAT is distinct from all these forms of iron, in that it is not soluble nor does it require solubilisation in the stomach to be absorbed since it is taken up by enterocytes as whole nanoparticles (Section 1.3). This means that the unabsorbed fraction of the compound that transits to the lower gut, and inherently this is at least 60% of all ingested oral iron irrespective of the form, will remain nanoparticulate and, therefore not soluble, and as such will not be available to promote pathogen growth and tissue inflammation. An important aspect of IHAT structure is that this is sufficiently labile to break down effectively inside the enterocyte and deliver its iron because the native iron oxo-hydroxide structure (i.e. ferrihydrite) in IHAT has been purposely destabilised with the incorporation of dietary tartaric and adipic acids (23), much in the same way to what occurs in the ferritin iron core due to interactions with the aminoacid residues in the protein shell (24). IHAT is a tartrate-modified, nano-disperse Fe(III) oxo-hydroxide in the ferrihydrite mineral phase, formed in an adipate buffer, with similar functional properties and small primary particle size (~2 nm) as the iron form found in the ferritin core (i.e. ferrihydrite). Full physicochemical characterisation in relation to particle size, morphology, iron phase, XRD pattern, infrared spectra, and dissolution are presented in Powell *et al* (23). All components of IHAT (i.e. iron oxide, tartaric and adipic acids) are naturally present in foods and are approved food additives with a generally recognised as safe (GRAS) status by the US Food and Drug Administration (FDA). Besides composing the ferritin core, iron oxides are naturally present in foods and soils and are approved food additives (E172). Tartaric acid is naturally present in foods, most notably grapes, and is an approved antioxidant and acidifier food additive (E334). Adipic acid is also naturally found in foods and is an approved flavouring and gelling agent (E355).

Each daily dose of IHAT in this study will contain in addition to the 20 mg elemental Fe, 21 mg of tartaric acid and 4.7 mg of adipic acid. These amounts of the organic acids are less than 20% of the estimated ADI (acceptable daily intake) for a 5 kg child and less than 10% of the respective ADI for a 10 kg child (http://ec.europa.eu/food/fs/sfp/addit_flavor/flav15_en.pdf).

A molecular representation of IHAT is presented in Figure 2.

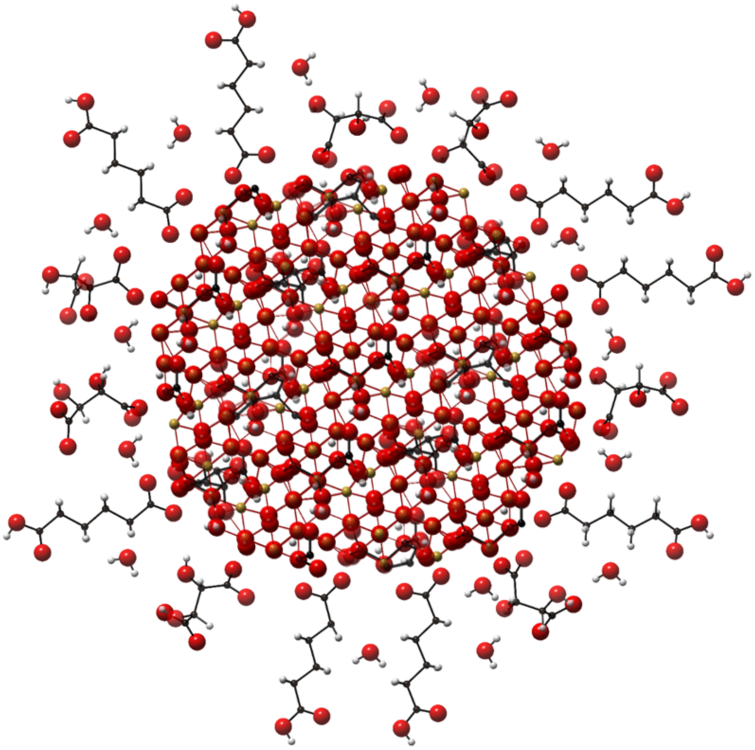

Figure 2. Molecular model of one IHAT 2 nm particle. The colours used to represent the atoms are: O (red), H (white), Fe (brown), C (black). The organic acids (tartaric and adipic) are represented adsorbed to the surface and incorporated into the structure core. We estimate that one IHAT particle would contain 150 Fe atoms (based on the ferrihydrite structure by Michel *et al* (25)). Chemical modelling by Dr Helen Chappell (unpublished).

Ferritin is composed of up to eight nanoparticles of iron oxo-hydroxide, also in the ferrihydrite mineral phase, stored in a protein shell that renders the iron particles both nano-dispersible and sufficiently labile to be utilized biologically. When ingested in either plant or animal based foods, it is well absorbed (26-28). The exact mechanism of absorption is not resolved- either ferritin is gradually broken down in the acidic, gastric lumen at a rate that matches later intestinal iron absorption or the particle resists total gastric degradation and is taken up whole by the enterocytes and gradually broken down intralysosomally to join the common iron pool. Either way, undigested nanoparticulate iron oxo-hydroxide should not be reactive in the same way that soluble iron is and, thus, should be poorly available to colonic bacteria or to participate in redox reactions at the epithelial surface (Section 1.3).

It was with this in mind, and based upon 20+ years of research into the chemistry of dietary minerals in the gastrointestinal lumen, that the co-PI and the team at MRC Human Nutrition Research have developed IHAT as a paradigm-shift from existing or pipeline products. IHAT is well absorbed and due to its nanoparticulate nature should carry very low side-effects as it should *not* undergo luminal redox cycling and it should *not* give up its unabsorbed iron to commensal and pathogenic bacteria (Section 1.3).

We are confident of IHAT's ability to outpace any other form of supplemental iron in use or under development. The anticipated advantages to the end user over competing and pipeline solutions will be (i) lack of acute 'sub-symptomatic', but nonetheless chemically undesirable, redox effects on the colonic mucosa and microbiome, (ii) reduced risk of intestinal infection and diarrhoea, and (iii) lack of symptomatic side-effects, all of which will ultimately result in better efficacy at correcting IDA.

1.3 Rationale

In this clinical trial we will test the hypothesis that supplementation with IHAT eliminates iron deficiency and improves haemoglobin levels in young children without increasing infectious diarrhoea or promoting negative changes in the gut microbiome or inducing gut inflammation. We propose to conduct the trial in the North Bank villages of the URR because these are some of the most deprived communities in the country where risk of infection, particularly enteropathogenic infection, is high. This will allow us to better distinguish IHAT and ferrous sulphate in terms of safety, which is where most of the clinical need for better iron supplementation lies.

Over the past ~10 years we have established sufficient pre-clinical and proof-of-concept data for IHAT to support moving to the Phase II trial proposed here. These data are summarised below and for the most part have been published. We have prepared an Investigator Brochure for IHAT comprising essentially the data summarised below, and have submitted this to the UK Medicines and Healthcare Products Regulatory Agency (MHRA), and asked for their scientific advice in relation to this protocol and the future development plan for IHAT, the MHRA's positive response letter is attached to this proposal. The Investigator Brochure for IHAT was also submitted to the Gambia Medicines Board, when we applied for clinical trial authorisation.

IP protection: IHAT is protected by an MRC-owned patent (WO2008096130) that has been granted in most major territories (e.g. Europe, US and China). The claims of this patent protect the composition-of-matter for the oral iron formulation to be taken into the clinic, as well as methods of manufacture and use for the preparation of a medicament for therapeutic delivery of

IHAT to a subject. The claims also protect the chemistry underlying the generation of oxo-hydroxide metal ion structures modified with organic moieties and thus offers broad protection of the chemical landscape.

Manufacture: A crucial advantage of IHAT, over competitors, is its facile synthesis leading to low manufacture cost compared to other complex iron formulations (namely iron chelates or protein-bound complexes). The cost of IHAT manufacture at scale is estimated as \$0.3-\$1.5 for a 28-day supply for one child (scale range 1-150 ton). This is only ~3x the cost of simple ferrous salts. Manufacture of IHAT involves a simple, but carefully-controlled, aqueous co-precipitation technique and product recovery is either by tray-drying or spray-drying. Based on feedback received from three contract-manufacture organizations (CMO) for the scale-up manufacture of IHAT, we are reassured that there will not be any significant hurdles in accommodating larger scale GMP manufacture.

Importantly, IHAT's manufacture is easily scalable and has a low cost due to the facile synthesis and inexpensive FDA GRAS (generally recognised as safe) raw materials. We anticipate that following this initial trial, we will have high-quality clinical data to support using IHAT instead of soluble iron (e.g. ferrous fumarate or sulphate) as the iron source in the micronutrient supplementation and home fortification (e.g. MNPs) interventions recommended by WHO to improve the iron status of populations. Following this initial pilot award, we would apply to BMGF for a full award that would be used to fund a trial where we would be investigating these different delivery systems with IHAT, different iron dosage regimens, and also assess efficacy in pregnant women. At that stage we would engage with the manufacturers of MNPs (for example DSM) to produce a formulation with IHAT instead of ferrous fumarate for testing.

Regulatory aspects: Scientific advice from the UK MHRA have assured us that the existing non-clinical and clinical data with IHAT would be sufficient to support the proposed trial (please refer to the MHRA letter dated 21st December 2016 attached to this proposal). Furthermore, advice from the MHRA in relation to a similar, but not identical, iron compound developed by our Group for a different indication was that toxicology studies would not be required and that the toxicology section of the CTA application could be comprised of data for the individual dietary components drawn from the literature. This compound has now completed Phase II testing in the USA.

For a Phase II/III adaptive trial in women (pregnant and non-pregnant) we also asked for advice from a regulatory consultancy and their report (commissioned to form part of a Wellcome Trust proposal pack) is also attached to this submission (Alacrita_151001 IHAT Regulatory Gap Analysis).

Commercial strategy: IHAT received the first prize at the 2014 Emerging Technologies Prize from the UK Royal Society of Chemistry (see http://www.rsc.org/chemistryworld/2014/12/solving-iron-solubility-problem-profile-mrc). As part of that prize we have been partnered with GSK who will continue to offer 'in-kind' support and mentorship as needed to help translate IHAT and bring it to those most at need of an alternative oral iron compound. MRC already has significant commercial interest from various commercial partners. The results of this trial will be very beneficial to a company and open up a new market for IHAT in the developing world.

Pre-clinical data: The main findings from the proof-of-concept and pre-clinical studies that support our hypothesis are presented below.

Phase 0 pharmacokinetics human studies: A study with 4 iron deficient women in the UK showed that (i) bioavailability (i.e. red blood cell incorporation) of iron from IHAT was ~ 75% that of iron from ferrous sulphate and (ii) IHAT was successful in reducing the non-physiological post-absorptive iron surge caused by ferrous sulphate that raises transferrin saturation (29). A recent single-dose study in 30 pre-menopausal women in West Kiang, The Gambia (SCC1422 – IHAT) has confirmed that a new recovery-method for IHAT's large-scale manufacture does not affect IHAT's bioavailability (Figure 3A); showed again that IHAT was successful in reducing the non-physiological post-absorptive iron surge caused by ferrous sulphate, that raises transferrin saturation (Figure 3B); and IHAT was successful in reducing *ex vivo* pathogen growth in the serum collected from the women following the single-dose (Figure 3C). We note that the relative bioavailability (RBV) of IHAT in the Gambia study (range 10-69%) was lower than that in the UK study (range 58-89%). However, the UK study had very few numbers, only 4 women, and, therefore, the full range of IHAT bioavailability was not observed.

For the purpose of calculating the bioequivalent dose for IHAT to use in the proposed trial we have considered the upper 75% centile of the median RBV (i.e. 60%), we took into account the data from both studies (n=25) since they were both conducted in the same population group (i.e. pre-menopausal IDA women). Our reasoning for using the upper 75% centile rather than the median value was that in IDA young children (in the absence of infection) we expect to have increased iron absorption due to their increased erythropoietic needs for growth and brain development relative to adult women and, therefore, we did not want to overestimate the dose needed.

Most importantly, IHAT was developed to simulate dietary iron absorption and slowly release the iron into the circulation so as not to cause an abnormal rise in transferrin saturation and this was indeed confirmed with the Gambia study (SCC 1422), in which the vast majority of women had TSAT always below 40% following the 60 mg Fe dose. This feature of IHAT has a number of advantages over ferrous sulphate which induces the un-physiological iron bolus release into the circulation: (i) the lower rise in serum Fe and TSAT after each daily dose of IHAT should not

cause a bolus rise in hepcidin which has been recently shown to inhibit absorption of the next-day dose with ferrous sulphate (30), (ii) less likelihood of formation of 'true' NTBI when TSAT is maintained below 40% (31), (iii) less 'free' iron highly available to sustain growth of systemic extracellular pathogens (32, 33).

A

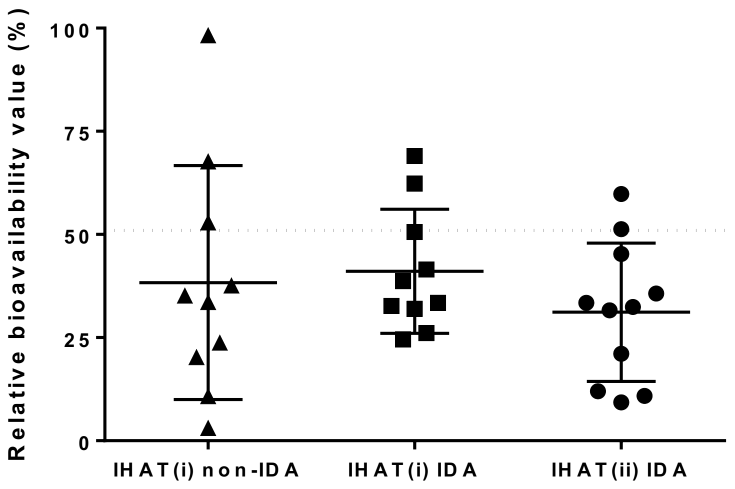

B

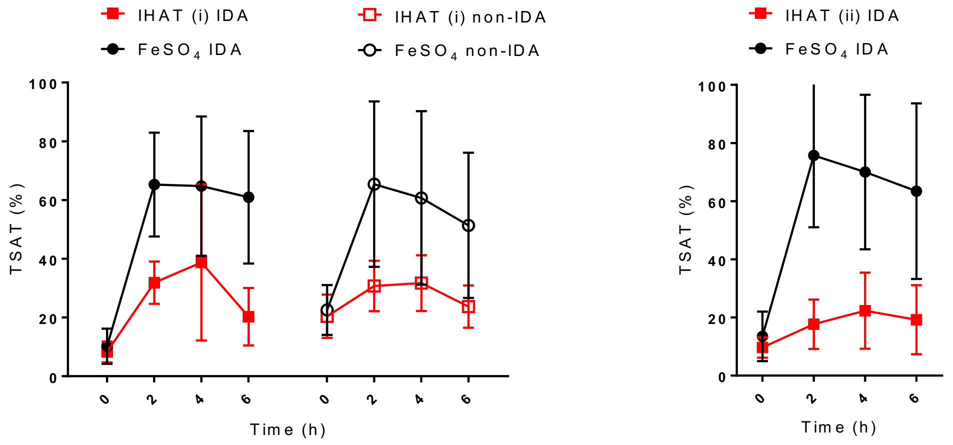

C

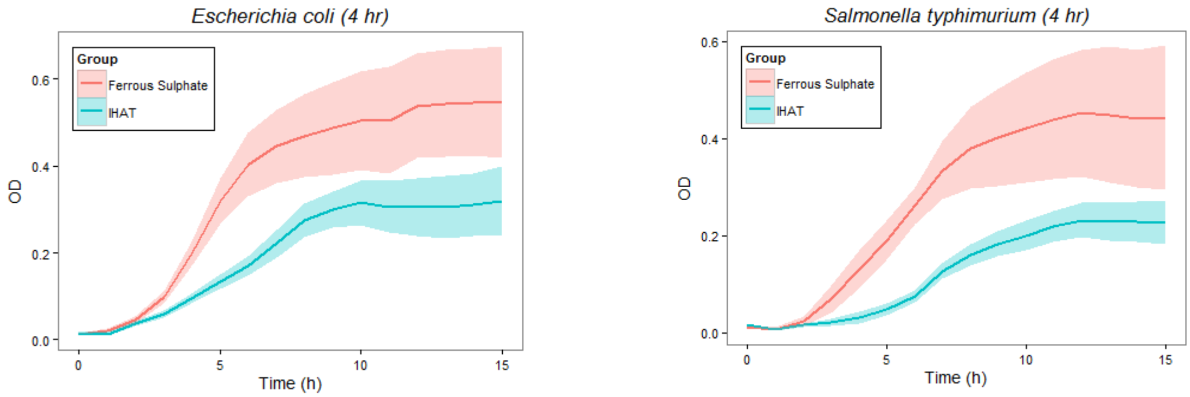

Figure 3. Unpublished data from the recent single-dose absorption study in pre-menopausal women in West Kiang, The Gambia (SCC 1422). A, relative bioavailability value (RBV) to ferrous sulphate (100%) determined from the red blood cell incorporation of the iron isotopes, box and whisker plots show median, minimum and maximum for n = 10 in each group, differences are not statistically significant; **B,** transferrin saturation following a single-dose of IHAT or ferrous sulphate (60 mg elemental Fe equivalent), data shown as mean (± SD, n=10/group), differences between IHAT and FeSO4 are statistically significant at the 2, 4 and 6 hours time-points, $p<0.01$; **C,** *ex vivo* bacterial growth of *E. coli* and *Salmonella typhimurium* in serum collected 4 hours following the single-dose of 60 mg Fe as IHAT or ferrous sulphate. There was strong evidence for the effect of supplementation group on bacterial growth ($p<0.0001$ using non-linear mixed effects modelling).

NOTE: Two identical IHAT powders were tested, these powders were recovered using a different procedure: IHAT (i) – tray-dried at 45 °C, IHAT (ii) – concentrated by solvent recovery and then tray-dried at 45 °C. The *ex vivo* assays were only conducted for IHAT(i).

Efficacy/haemoglobin repletion study in rats (14-days): Two independent studies have shown that IHAT is equivalent to ferrous sulphate at correcting haemoglobin levels in anaemia. No differences in bodyweight were observed between groups and there was no detectable iron deposition in the mucosa of the small intestine with IHAT (23, 29).

Pharmacokinetics mouse study (4 hours, radio-labelled): Absorption of IHAT was significantly increased in iron-deficient mice compared to iron-sufficient mice, showing that systemic absorption of IHAT is normally regulated by body iron levels (34).

ADME mouse studies (7 & 28 days): In two independent studies, IHAT was equivalent to ferrous sulphate at correcting haemoglobin levels. Systemic absorption of IHAT, i.e. basolateral export from the intestinal enterocyte into the blood circulation, was via ferroportin (as with ferrous sulphate) and was under regulation of normal iron homeostasis. Expression of duodenal DMT1, expression of liver hepcidin, liver iron, splenic iron and duodenal iron levels were all similar between the 2 groups (34, 35).

Mechanism of intestinal apical uptake - *in vitro* and *in vivo* studies:

In cellular and animal studies, IHAT was shown to be taken up apically as whole nanoparticles- i.e. a separate mechanism to the DMT1-driven uptake of conventional soluble iron supplements – and therefore did not require luminal or mucosal iron redox activity:

In mice, IHAT's absorption did not require redox activity (i.e. reduction of Fe(III) to Fe(II)) (23).

In Caco-2 differentiated cells (cells that resemble the duodenal enterocytes once differentiated), IHAT was taken up by the cells by endocytosis and this was followed by breakdown of IHAT inside endosomes/lysosomes within the cell to release the Fe (36).

In HuTu 80 cells (duodenal adenocarcinoma cells), IHAT was taken up by a mechanism independent of the divalent metal transporter 1 (DMT1) and of the duodenal cytochrome b (Dcytb) (34).

Correlation with human absorption: Caco2 Fe uptake studies

IHAT absorption in humans correlated with direct *in vitro* cellular uptake, but not with gastric solubility (29).

Importantly, we wish to note that even though IHAT is taken up apically by the enterocyte via a different mechanism to soluble iron, once inside the cell IHAT dissolves rapidly inside late-endosomes and lysosomes due to the combined action of low pH and high ligand-affinity for ferric iron (typically ~10mM citrate). Then, the iron released from IHAT in these cellular vesicles joins the common cellular labile iron pool (LIP) and does not bypass the normal physiological mechanisms that regulate iron absorption. Our data above indicate that IHAT nanoparticles do not translocate intact from the gut epithelium into the blood circulation.

Safety proof-of-concept *in vitro* assays: IHAT had no adverse effect on Caco-2 gut cell viability, even at doses 10-20 fold in excess of those possible in the gut lumen, whereas soluble ferrous iron was markedly toxic at 14-fold lower doses than IHAT (29). IHAT had no adverse effects on epithelial cell monolayer integrity (36).

IHAT had no adverse effects on Caco-2 (colon) and Hutu-80 (duodenum) gut cell viability or on cell growth and proliferation even at doses much higher than those expected in the gut lumen. Unpublished data indicate that ferric maltol is more toxic still.

Safety proof-of-concept *in vivo* studies: In an 'enteric infection' (i.e. microbiome dysbiosis) rodent model, IHAT was much less available to potential enteropathogens than ferrous sulphate and led to a more beneficial gut microbiome, with increasing Lactobacilli after 14 days supplementation. (29).

In a 'healthy colon' rodent model, IHAT did not negatively impact the gut microbiome and appeared to be less available as an iron source to bacteria after 28 days of supplementation (37).

1.4 Potential risks and benefits

Our vision is that our novel iron compound (IHAT) will significantly contribute to the goal of safely eliminating iron deficiency and iron deficiency anaemia in women and children living in developing countries. Our existing evidence strongly supports the contention that IHAT offers very important advantages over any other form of supplemental iron in use or under development.

The anticipated advantages to the end user over competing and pipeline solutions will be (i) lack of acute sub-symptomatic, but nonetheless chemically undesirable, redox effects on the colonic mucosa and microbiome, (ii) reduced risk of intestinal infection and diarrhoea, and (iii) lack of symptomatic side-effects leading to better compliance; all of which will ultimately result in better efficacy at correcting iron deficiency anaemia. Furthermore, as described above, IHAT's absorption into serum follows a slower 'dietary-like' kinetics and it should not result in the non-physiological post-absorptive iron surge caused by ferrous iron salts, therefore, supplementation with IHAT should carry less risk of systemic infection such as tuberculosis and other co-infections normally associated with malaria. IHAT has a low-cost of manufacture due to its facile synthesis and inexpensive raw materials and, therefore, there should be no constraints to its implementation as an iron supplement for populations in resource-poor countries.

If IHAT is successful in this first trial, i.e. safer in terms of diarrhoea and gut effects than ferrous sulphate and not worse at correcting IDA, these data will be used to support a full award application to the BMGF together with a translation/commercial partner so that IHAT can be implemented as a novel iron supplement for use in micronutrient intervention strategies in

developing countries and, hence, help to reduce the global burden of IDA. Ultimately, following the next trial (supposedly in pregnant women and children) we envisage that IHAT would be used instead of ferrous sulphate and ferrous fumarate in micronutrient intervention strategies across the developing world. This fast-track clinical development (i.e. ability to move directly to Phase II and not Phase I studies) is only possible due to (i) the fact that iron supplements already have a WHO recommended daily active dose for the several age groups and (ii) the dietary-like nature of IHAT.

IHAT is an analogue of natural food iron and is made-up of dietary constituents that are approved food ingredients by the US Food and Drug Administration (FDA), namely iron oxide, tartaric acid and adipic acid and have the FDA generally recognised as safe (GRAS) status. More details about the dietary-like molecular structure of IHAT are explained in Section 1.2.

We have prepared a risk mitigation strategy for the proposed trial, in discussion with our program officer at the BMGF, and this is shown below. We are also required by the Sponsor to develop a risk assessment and risk mitigation strategies prior to study starting and we are currently working with MRCG to develop such documentation.

1.4.1 Risk mitigation

- Stability of MRC Unit The Gambia at LSHTM; the Unit has just been approved for renewed funding from the UK Medical Research Council until 2021.
- Regulatory approval for the clinical trial in children; based on scientific advice from the UK MHRA (mentioned above) and our previous experience with an iron compound targeting a different clinical application (phosphate binder for end-stage CKD patients) we are confident that we will obtain authorisation to proceed directly to the trial proposed here. With the MRC Unit The Gambia at LSHTM we have already conducted an early-stage clinical trial with IHAT (single-dose study SCC 1422) and both the Ethics Committee and the Medicines Control Agency in the Gambia have reviewed the pre-clinical data with IHAT for that study.

  We are submitting a clinical trial authorisation (CTA) to both the Gambia and the UK MHRA (for protocol review), even though we are not legally required to apply to the MHRA, since we feel this is best practice and in line with the EU/Horizon 2020 guidelines for best-practice for clinical trials conducted in developing countries (http://ec.europa.eu/research/participants/portal/doc/call/h2020/h2020-msca-if-2015/1645175-h2020_-_guidance_ethics_self_assess_en.pdf).

We have contacted MHRA in relation to our intention to submit a CTA dossier for the trial proposed here and received confirmation that it would be appropriate to seek MHRA approval/review of the trial design and supporting pre-clinical data as part of a scientific advice meeting alongside local Gambia approvals. This MHRA meeting has now taken place and their scientific advice letter is attached to this proposal. The trial will be conducted in accordance with the principles of GCP as laid down in the Consolidated Guideline for Good Clinical Practice published by the International Conference on Harmonization in 1996 (ICH GCP Guideline) and the MRC Unit The Gambia at the London School of Hygiene & Tropical Medicine will Sponsor the research.

- Ethical approval; even though the planned trial is early-stage, given the long experience of the MRC Unit The Gambia at LSHTM in conducting oral iron interventions in children in The Gambia, the relatively low burden on study participants, and the use of children that are generally healthy apart from having IDA, we do not anticipate any ethical issues that could delay approval. The study protocol will be explained to the child mother/guardian orally in the presence of an independent and literate witness following MRCG SOPs in case they are illiterate or in writing and we will not start any study specific procedures before informed consent is obtained. The study investigates oral iron used in doses specifically recommended for this age group and even though IHAT is a novel compound, it is made up entirely of compounds naturally present in the diet and all our pre-clinical data supports that it should not behave any differently from dietary ferritin. In any case, even if IHAT is not absorbed and does not correct anaemia, it would not be worse in terms of safety that the forms of iron already in use in supplementation and home fortification strategies in this age group. We are assured that there should not be any ethical concerns over delaying 4-months the supply of iron treatment to the children enrolled in the placebo group since that it is not standard-of-care to provide routine iron supplementation to children in The Gambia and most children are not regularly screened for anaemia or IDA. All our children will be closely monitored during the study and all children that remain anaemic (Hb<11 g/dL) at the end of the study period (3 months intervention + 1 month AE follow-up), irrespective of their treatment group allocation, will be provided with iron supplementation for 3 consecutive months according to national and WHO guidelines. Severely anaemic children (i.e. Hb<7 g/dL) will not be enrolled in the study and if during the study a child (in any group) is found to become severely anaemic, they will discontinue the study supplementation and will be offered iron supplementation for 3 consecutive months according to national and WHO guidelines. These children will continue to be followed up at the weekly clinics for AEs and Hb levels. These children will not be excluded from data analysis and will be regarded as treatment failures. A full rationale for the inclusion of the placebo group is provided in Section 11.1.1.

- GMP manufacture of the clinical batch (low): as mentioned above, based on the feedback received for the scale-up manufacture of IHAT from CMOs, we are reassured that there won’t be any significant hurdles in accommodating larger scale GMP manufacture. For the trial proposed here, the clinical batch of IHAT will be manufactured under cGMP conditions by Shasun

Pharma Solutions in the UK and the powder-filling of the capsules, packaging and labelling will be provided by Capsugel in Belgium, also under cGMP conditions.

- Recruitment of participants (low); mitigated by highly experienced staff and well engaged communities in The Gambia. Assuming that ~ 50% of children under 3y in the Upper River region would fit the study eligibility criteria (the prevalence of low ferritin in this age group is based on our current data from the HIGH study) then we would need to screen ~1500 children. There are ~1700 children 6-35 mo. of age in the Wuli and Sandu districts in the North Bank within a 20 km distance of the Basse field station from where we would initially recruit into the study. We would start recruitment in Nov 2017 for the 1st cohort and would finish in July 2018 for the 3rd cohort. We will monitor recruitment rates closely and if necessary we can extend the catchment area. The catchment area of the Upper River Division will be sensitised and a team led by the local PI (Clinical Trial Coordinator) and the field coordinator will tour all the villages in this area to sensitise them on the project.

- Increased malaria and co-infections risk during and after oral iron supplementation; there are risks associated with a large intake of iron supplements especially in areas of malaria endemicity. The dose of iron given daily in the reference arm (12.5 mg) is according to WHO guidelines for the age group children in non-malarious areas or malaria-endemic areas where it should be implemented in conjunction with measures to prevent, diagnose and treat malaria and co-infections. We have put in place the following strategies to mitigate the risk of possible interactions between iron supplements and malaria or other co-infections: (1) data from the Gambia over the last 5 years (medical records from the Kiang West region) shows that the peak malaria months are October and November and, therefore, we have timed the study intervention periods to avoid these months so that there will be no intervention/supplementation during Oct and Nov 2017 (2) trained field workers will be visiting all children every day during the 12 weeks supplementation period in order to supervise the administration of the iron supplements or placebo and on these occasions they will check on the children’s health status and actively look for signs of malaria and co-infections, if a child shows signs of these infections the study nurse will perform adequate tests and the child will be offered the appropriate treatment/referral to the next Health Centre. In case of a fever, a malaria rapid test will be performed and if positive the child will be treated according to national guidelines. A sick child will always be visited by a study nurse for further clinical investigations and if needed referred to the nearest Health Centre. These visits will carry on for 4 weeks after the end of the study intervention and, during both the intervention and this follow-up period, morbidity data will be captured every other day. This is similar to what was done in the HIGH study and we do not anticipate any difficulties of implementation. Every week, the investigators will check whether children are RDT positive. Note: according to national guidelines, all children at birth are provided with bed nets and children in the URR receive intermittent preventive treatment (IPT) of malaria and so we have not anticipated we would need to provide insecticide-treated bed nets to the study population, but we could do this is deemed necessary.

- Analytical (low); mitigated by high level expertise of the laboratory technicians at the MRC Unit The Gambia at LSHTM and the internationally-recognised excellence of the Sanger Institute Group in microbiome and pathogen analysis and the expertise of the group at Kings College London in NTBI analysis.

2 Study objectives

In this trial we will test the hypothesis that supplementation with IHAT eliminates iron deficiency and improves haemoglobin levels in young children without increasing infectious diarrhoea or promoting negative changes in the gut microbiome or inducing gut inflammation.

There are four null hypotheses in the study:

1) non-inferiority of IHAT compared to ferrous sulphate for efficacy (in terms of Hb and iron deficiency correction: i.e. IDA) response probability (or prevalence). In this case we are testing the null hypothesis that: response probability in the IHAT arm minus the response probability in the ferrous sulphate arm is less than or equal to – 0.1. **We define 'response' for IDA as correction of iron deficiency and either achieving a normal Hb or an increase of at least 1 g/dL after 12 weeks of iron supplementation.**

2) superiority of IHAT compared to ferrous sulphate in terms of incidence density of diarrhoea. Here the null hypothesis is that the mean number of new episodes in the IHAT arm is greater than or equal to the mean number in the $FeSO_4$ arm.

3) superiority of IHAT compared to ferrous sulphate in terms of prevalence of diarrhoea. Here the null hypothesis is that the prevalence of diarrhoea in the IHAT arm is greater than or equal to the prevalence in the $FeSO_4$ arm.

4) non-inferiority of IHAT compared to placebo in terms of prevalence of diarrhoea. Here the null hypothesis is that the prevalence of diarrhoea in the placebo arm minus the prevalence in the IHAT arm is less than or equal – 0.1.

The primary objective for this trial is to show non-inferiority of IHAT in relation to ferrous sulphate at correcting IDA, and in terms of diarrhoea to show superiority in relation to ferrous sulphate and non-inferiority in relation to placebo (as defined above). Secondary objectives are to show that IHAT is non-detrimental with respect to enteric pathogen burden, the gut microbiome, and intestinal inflammation.

2.1 Study endpoints

Primary endpoints:

There are 4 primary endpoints of the trial:

1. iron deficiency at 12 weeks

2. haemoglobin levels at 12 weeks

3. 'incidence density' of moderate-severe diarrhoea over the 12 weeks (i.e. the number of new moderate-severe diarrhoea episodes per child over the 12 weeks intervention)

4. 'period prevalence' of moderate-severe diarrhoea over the 12 weeks intervention period (i.e. the proportion of children with at least one episode of moderate-severe diarrhoea over the 12 weeks intervention)

Efficacy primary endpoints:

To assess iron deficiency we will take into consideration the most up-to-date recommendation from WHO who are currently conducting a consultation on this matter. The new WHO guidelines will include a recommendation for the best marker of iron deficiency in the context of inflammation and we expect this to be either using ferritin alone or the sTfR/logferritin index, where in both cases ferritin values will be inflammation-adjusted. There are several models we can use to adjust ferritin for inflammation, for example use unadjusted ferritin with the cut-off with optimal sensitivity and specificity from the Malawi study (<18 µg/L), use the CRP/AGP regression model being investigated by Parmi Suchdev (BRINDA) to adjust ferritin values, use the Thurnham et al (38) stratified adjustment or the continuous adjustment used by Engle-Stone et al (39). The choice of the method for adjusting ferritin concentrations and of which marker to use to define ID (ferritin alone or the sTfR/logferritin index) will be made at the time of locking the data analysis plan, in consultation with an expert on this matter (Dr Sant-Rayn Pasricha), our BMGF Program Officer (Dr Ken Brown) and taking into consideration the results from Brinda and the results from the ongoing WHO consultation on this matter.

Iron deficiency and haemoglobin levels at 12 weeks will be used to define the prevalence of IDA which we will use to assess non-inferiority of IHAT relative to $FeSO_4$ in terms of efficacy. **We will determine the proportion of children who resolve iron deficiency <u>and</u> either achieve a**

normal Hb or an increase in Hb of at least 1 g/dL after 12 weeks of iron supplementation.

Safety primary endpoints:

Incidence density and period prevalence of moderate-severe diarrhoea will be used to show superiority of IHAT in relation to ferrous sulphate and non-inferiority of IHAT in relation to placebo.

Diarrhoea is defined as 3 or more loose or watery stools per day and **moderate-severe diarrhoea** refers to those diarrhoea episodes where (i) the child passes more than 5 loose or watery stools per day, (ii) there is blood in the stool (dysentery), or (iii) the child shows signs of clinical dehydration (assessed by the study nurse based on physical signs such as little or no urination, sunken eyes, and skin that lacks its normal elasticity). These episodes will require treatment (including ORS) and will be recorded as adverse-events.

'Incidence density' is defined as the number of new moderate-severe diarrhoea episodes per child over the 12 weeks intervention and 'period prevalence' is defined as the proportion of children with at least one episode of moderate-severe diarrhoea over the 12 weeks intervention.

Secondary endpoints:

Secondary endpoints will be faecal microbiome diversity and profile (particularly in terms of abundance of Enterobacteria), abundance of enteric pathogens, faecal calprotectin (marker of gut inflammation), hospitalisation and morbidity (data collected three times per week using the questionnaire developed in the HIGH study), malaria infection, treatment failures (i.e. the number of children who have to discontinue the study intervention because their Hb falls below 7 g/dL), the proportion of days a child has diarrhoea over the 12 weeks intervention period ('longitudinal prevalence' of diarrhoea), the proportion of days a child has moderate-severe diarrhoea over the 12 weeks period ('longitudinal prevalence' of moderate-severe diarrhoea), 'incidence density' of bloody diarrhoea (i.e. the number of bloody diarrhoea episodes per child-month of observation), markers of systemic inflammation (serum CRP and AGP), and systemic markers of iron handling (hepcidin, sTfR, transferrin saturation and circulating non-transferrin bound iron - NTBI).

All outcome measures will be assessed at 0, 2 and 12 weeks in all the children.

The 2 weeks time-point will provide an indication of acute compound-related effects and the 12 weeks time-point of chronic (i.e. longer term/chronic) effects.

3 Study design

3.1 Type of study and design

3-arm, parallel, randomised, placebo-controlled, double blind.

Children will be randomised (1:1:1) to IHAT, ferrous sulphate or placebo, each arm will include an intervention period of 12 weeks, faecal and blood samples will be collected at the time-points indicated below.

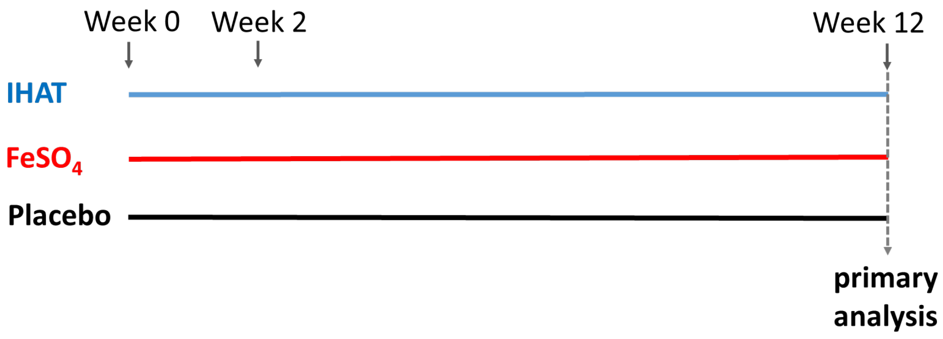

Participants will be iron deficient anaemic young children (n=705, accounting for 15% loss to follow-up) living in rural communities in the Upper River Division in The Gambia. Children will be recruited and randomised into the 3 study arms (n=235 per arm). Inclusion criteria will be: apparently healthy as judged by a study nurse on the day of recruitment, age 6-35 months, no malaria (RDT negative) and IDA defined as 7≤Hb<11 g/dl (for recruitment we will use Medonic Hb values rather than HemoCue) with serum ferritin<30 µg/L.

Each arm will include an intervention period of 12 weeks. As suggested by our BMGF proposal reviewers, we have included a placebo arm to allow us to fully evaluate IHAT effects, particularly to rule-out negative impact on diarrhoea episodes, the gut microbiome and compound-related

bacterial infection, and to determine treatment effect sizes with either IHAT and ferrous sulphate that will be necessary to power future iron supplementation studies (please refer to Section 11.1.1.).

3.2 Randomisation and blinding procedures

3.2.1 Randomisation

Recruited children (N=705) will be randomly assigned (computer generated) to receive one of the 3 treatment arms (N=235 in each arm) stratified by the Hb concentration prior to enrolment and age. This will assure that Hb concentration at baseline (as a proxy for erythropoietic demand, which is the main driver for iron absorption) will not differ between the 3 arms.

At recruitment, each child will be categorised into two Hb classes (below or equal to/above the median Hb for that recruited cohort) and also according to age into 3 classes (6-11 months, 12-23 months and 24-35 months). This will divide children into 6 different strata and in each strata the children will be randomly assigned to one of the 3 treatment arms using a computer program and a block randomisation approach with fixed block size by age and Hb levels as defined above.

Ideally subjects will be recruited so that there will be balanced numbers in each of the 3 age classes; each of the age classes are further categorised into two groups based on the Hb level. Thus, in the balanced case, a total of 117-118 subjects each will belong to one of the six strata, created by Hb level (Low, High) and age group (Young, Middle, Old) combinations:

a. Low Hb, young age group
b. Low Hb, middle age group
c. Low Hb, old age group
d. High Hb, young age group
e. High Hb, middle age group
f. High Hb, old age group

However, 204 random treatment allocations will be generated for each strata in order to allow flexibility with respect to the numbers recruited in each of the six groups (a)-(f). That is, the list of treatment sequences in the balanced case will be extended by 86-87 to account for inbalance in the ages of children enrolled in the study as it may not be possible due to the age demographics of the study communities to enrol equal numbers in each of the age classes above. In any case, we will ensure that equal numbers within each strata are assigned to one of the treatment arms. This will be based on a pre-specified list of IDs ranging from 001-204, 205-408, 409-612, 613-816, 817-1020 and 1021-1224 for groups (a)-(f) above, respectively.

The study supplements will be provided in 750 individual bottles from the manufacturer. These bottles will be labelled using a random bottle ID ranging from 1 to 750 with a check digit character. The check digit character will be augmented to each of these numbers to make up the full subject IDs which will be generated using the Damm algorithm (https://en.wikipedia.org/wiki/Damm_algorithm; date accessed 08 November 2016). Here the check digit numbers 0-9 will be converted to letters A-H and J-K. Thus, there will be 250 bottles with randomly assigned bottle IDs for each of the three treatments (A, B or C). An electronic copy of the list will be maintained to allow electronic randomisation. The key code to which IMP (IHAT, $FeSO_4$ or placebo) corresponds to A, B or C will not be known to the statistician running the randomisation nor to anyone in the study team.

After children in each cohort are classified into one of the six strata based on their age and Hb level, a single bottle will be assigned to the child by selecting a bottle from one of the three groups (A, B or C) according to the treatment allocation, as determined above. The procedure will be repeated until the target number of subjects (n=705) are randomised. This will be performed using a computer programme. A complimentary programme will also be developed with a Microsoft Access application that will generate the appropriate bottle ID for a given screening ID which can be used on site. The bottle IDs will be used as subject/randomisation IDs and written on all relevant forms.

The randomisation code and the application to generate subject IDs on site will be written by Nuredin Mohammed (the trial statistician based at MRCG) and reviewed by David Jeffries (the Head of Statistics MRCG). The final code will be run using a random seed number by a member of the Statistics department who is independent of the trial. The generated randomisation list and the seed number will be recorded in a randomisation database (MS access database). The database and the application will be password protected and stored in a secure server.

After randomisation, the allocation list will be kept electronically and access will be granted to an independent clinician/nurse at the Basse site. A paper copy of the allocation list will be kept securely in the Sponsor’s office (by an independent statistician). The DSMB will have access to grouped data but we would provide unblinded data to the DSMB for their closed meetings if requested. If emergency unblinding is required at the request of the DSMB, only the particular study subject in question will be unblinded, since we will have an individual supplement code for each participant. Following investigation by the DSMB of an emergency unblinding case, and at the request of the DSMB, we may also unblind a whole treatment arm or the entire study. In all cases, someone independent from the trial team will perform the unblinding.

3.2.2 Blinding

Participants, field workers (Senior Field Assistants, Field Assistants, Field Supervisor and Field Coordinator), study nurses, scientific officers, research clinician, local PI (Clinical Trial Coordinator), CI, Clinical Trial Monitor and the entire study team will be blinded as to which treatment group participants belong to. Each treatment dose (iron compounds and placebo) will be encapsulated in identical capsules (also containing powders of identical colour) by Capsugel in France and the supply of capsules for each child will be packed in one bottle that will be individually labelled with a random bottle ID ranging 001-750 with a check digit character. This means that each child will have a unique treatment code that will also be their study ID/randomisation number as specified in 3.2.1. This individual labelling has the advantage of allowing to unblind only one child, if it becomes necessary, without compromising the blinding of the study.

The manufacturer will provide 750 individual bottles (allowing for some spares) comprising 250 bottles with randomly assigned bottle IDs for each of the three treatments.

MRCG will pass on the randomisation list to Capsugel with the treatment code key (A, B, or C) corresponding to each of the bottle IDs and Capsugel will make the decision of which compound is A, B or C. A Sponsor representative will keep the allocation list safe and since Dr Pereira (co-PI) will also remain blinded, we suggest an independent statistician acts as the key holder for the treatment arm codes and allocation list. This list will be kept in electronic format (password protected) and in paper copy (locked cabinet).

The pre-packed weekly supplies of the capsules will be prepared following a study-specific procedure (SSP) by the research clinician, field coordinator and nurse coordinator and the process will be supervised by the clinical trial coordinator (local PI) in Basse. Supplies will be labelled with each participant's ID, they will then be handed over to the Field Coordinator who is responsible for distribution to the field workers who will supervise consumption according to the pre-defined protocol for administration of the oral doses. Staff will be fully trained in all aspects of this activity and quality-control measures will be put in place to ensure that the SSP is followed exactly.

The blinding for a particular study participant may be broken if safety issues arise and we are advised by the DSMB to do so. A study-specific procedure describing the unblinding process will be developed.

3.3 Sub-studies

None.

3.4 Investigational products

3.4.1 Description of products

The trial has 3 arms:

IHAT- iron hydroxide adipate tartrate: an analogue of natural food ferritin iron (as detailed in Section 1.2); **Ferrous sulphate**- the gold standard for iron supplementation and **Placebo** (sucrose).

To avoid the need to use any tablet compression excipients or encapsulation materials, and to investigate the effects of just the active IMP compounds, the iron preparations, i.e. IHAT and ferrous sulphate, and placebo comparator, will be supplied as powders with each dose contained in a hard-gelatine powder-filled easy-open capsule.

3.4.2 Formulation, packaging and labelling

Each daily dose will be contained in a hard-gelatine powder-filled easy-open capsule. Each capsule will contain the single active IMP compound (IHAT or ferrous sulphate) or the placebo compound mixed with a small amount of a food colorant to colour-match the IMP powders. Capsules for each treatment will be packed in medicine bottles, each bottle containing enough capsules for one child for the entire study duration (allowing for some spares in case of accidental loss), and these bottles will be individually labelled with a unique code per participant, which will also be the subject randomisation code as described above. The key to which treatment corresponds to which code will be unknown to the study team or study PIs. The weekly supplies of each treatment for each child will be packed in Basse by the senior trial team and supervised by the clinical trial coordinator (local PI) and labelled with each participant's ID, which will be the same as the bottle ID as described in the randomisation procedure above. The bags with the weekly supplies per child will then be handed over to the Field Coordinator who is responsible for distribution to the field workers who will supervise consumption according to the pre-defined protocol for administration of the oral doses.

3.4.3 Product storage and stability

The capsules will be stored in dry conditions (the capsules will be packed in bottles containing desiccator sachets incorporated in the lids) and away from direct sunlight at MRCG Basse (this will be in an air-conditioning room in MRCG Basse clinical services below 25°C). The products are stable for 24 months if kept below 25°C. A full long-term storage stability assessment is going to be performed by Capsugel, the formulation company preparing the capsules (following recommended ICH protocols for the Gambia climate region: 25°C/60%Relative Humidity (representing normal storage conditions) and 30°C/65%Relative Humidity (representing transport conditions)) for both IHAT and comparators and the capsules will be packed and stored accordingly to these findings (these data will be available prior to study starting when the cGMP capsules are released by Capsugel to be shipped to the Gambia).

The weekly supplies taken out to the field do not require any special short-term storage conditions and will be kept by the field workers in their study bags in sealed plastic bags.

3.4.4 Dosage, preparation and administration of investigational products

The very recent WHO recommendation for the iron supplementation daily dose is 10-12.5 mg elemental Fe for infants 6-23 mo. and 30 mg elemental iron for children 24-59 mo. (40). The iron dose for ferrous sulphate will be 12.5 mg elemental Fe (~38 mg ferrous sulphate monohydrate) once a day for our entire study population (6-35 mo.), rather than having 2 different dosages according to the age groups, as this is still in line with the Gambian national guidelines. The iron dose for IHAT will be the iron-bioavailability equivalent (bioequivalent) in relation to ferrous sulphate as defined above in Section 1.2, i.e. assuming RBV of 60%. The bioequivalent iron dose for IHAT will be 20 mg elemental Fe (equivalent to 80 mg IHAT powder).

Each daily dose will be contained in a hard-gelatine powder-filled easy-open capsule. On the day of administration, the field assistant will open the respective capsule (corresponding to the child study ID according to the randomisation) and add the entire powder content of the capsule to 10 ml of a sugar juice drink contained in a disposable plastic cup. The drink will be used to mask the metallic taste of the iron powders, so that the supplementation is more acceptable to the child. The dose will be administered directly into the child's mouth using a disposable Pasteur pipette (in the younger children), a big spoon or drunk directly from the plastic cup (in the older children). There will be a Study Specific Procedure for this dose-administration protocol, which will be followed by each field worker and specific training will be provided before the start of the study. Whenever possible, each dose will be ingested after a feed or within 1 hour of the last meal, since iron supplements are generally better tolerated when ingested after meals rather

than on an empty stomach. If, exceptionally, the child has not been fed before supplementation, the mother will be encouraged to feed the child immediately after supplementation.

In order to be able to do this protocol for supplement administration and not compromise the double-blind design of the trial we have been working with the formulation company (i.e. Capsugel) producing the capsules to ensure that all powders (IHAT, ferrous sulphate and placebo) have the same colour and a small amount of a food colorant will be added to all the powders prior to filling the capsules). The IHAT powder will be produced by Shasun Pharma Solutions in the UK (now called Sterling Pharma) and sent to Capsugel who will formulate the IHAT and source and formulate the ferrous sulphate and placebo (sucrose) in the gelatine capsules.

3.4.5 Concomitant medications/treatments

Participants will remain under the care of the Wuli and Sandu RHT teams.

Any subject whose Hb falls below 7g/dL will be referred to the closest health centre to receive iron supplementation for 3 consecutive months according to national and WHO guidelines. We will support the RHT systems by ensuring a constant supply of malaria diagnostics, anti-helminth treatment, basic drugs and iron syrup (for all young children – not just study participants) for the duration of this trial. All children participating in the study who remain anaemic after the study intervention and follow-up period (i.e. after the 4 months) will be offered the iron syrup for 3 consecutive months according to national and WHO guidelines.

No specific medication is prohibited during the study, with the exception that participants in the study are not allowed to take any other form of iron supplementation, including multimicronutrient powders containing iron and iron syrups for the entire duration of the study, i.e. during the 12 weeks intervention and 4 weeks AEs follow-up.

4 Selection and withdrawal of participants

4.1 Selection of participants

Since the welfare card/immunisation cards in the North Bank of the URR are not available for all children and records are unreliable, children between the age of 6-35 months will be identified through the enumeration data collected by the study field team within the study catchment area in the Wuli and Sandu districts (distance of ~ 25 km North of Basse, Figure 4). The compounds within the study catchment area are identified from the mapping exercise conducted by our field team. These mapping and enumeration/census data are captured in an electronic database and will be linked with the eCRFs.

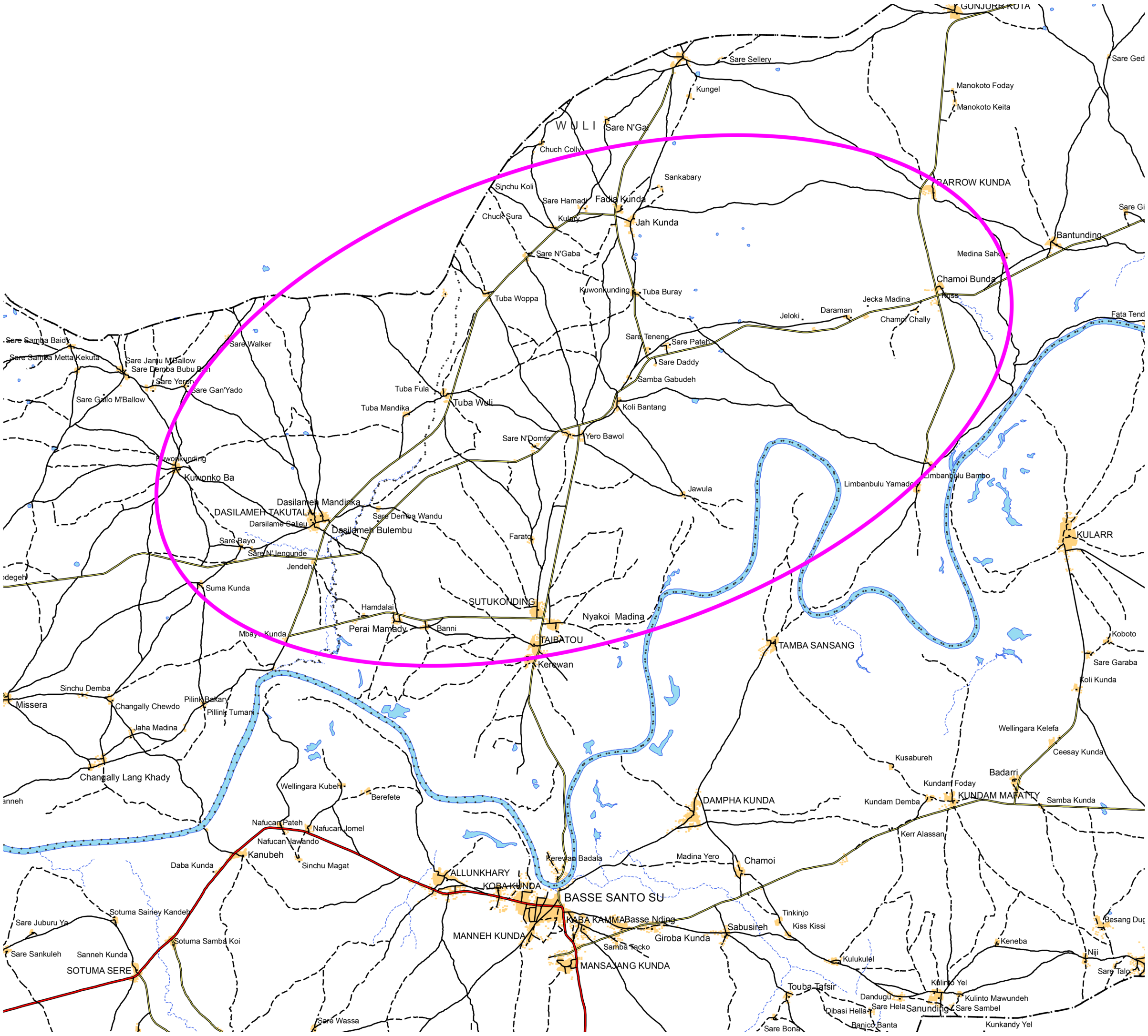

Figure 4. Study Catchment area. The area demarked represents an area of ~ 20 Km radius (at a maximum distance of 25 km by straight line from Basse), and corresponds to an area perimeter of ~ 60 km. Study nurses will be stationed at the following health facilities in the Nortn

Bank of the URR: Yorrobawol Health Center, Darsilami Community Health Post, Konkuba Community Health Post, Taibatu Health Post and Chamoi Health Center.

Assuming that ~ 50% of children under 3 y. in the Upper River Region would fit the study eligibility criteria (the prevalence of low ferritin in this age group is based on our current data from the HIGH study) then we would need to screen ~1500 children. From our initial community census exercise, we estimate that there are ~1700 children 6-35 mo. of age in the Wuli and Sandu districts in the north Bank within a 20 km distance of the Basse field station from where we would initially recruit into the study. We would start recruitment in Nov 2017 for the 1st cohort and would finish in July 2018 for the 3rd cohort. We will monitor recruitment rates closely and if necessary we can extend the catchment area.

The catchment area of the Upper River Division will be sensitised and a team led by the local PI (Clinical Trial Coordinator) will tour all the villages in this area to sensitise them on the project. For the sensitisation we will use the Study Communication Plan developed together with the MRCG Communications Team.
The Regional Health Team (RHT) and MRCG Basse will advise on all studies taking place in the area. All mothers/guardians of eligible children will be asked if their child participates in another study and children will only recruited if this is not the case. They will also be asked if they intend to remain in the study catchment area for the entire duration of the study period (i.e. 4 months).

4.2 Eligibility of participants

Participants must meet all of the inclusion criteria and none of the exclusion criteria to be eligible to participate in the trial.

4.2.1 Inclusion criteria

Apparently healthy as judged by a study nurse at day of screening and recruitment
Age 6-35 mo.
Free of malaria (RDT negative)
HAZ, WAZ, WHZ >-3 SD
IDA defined as 7≤ Hb <11 g/dl AND ferritin<30 µg/L, as per WHO recommendation for children under 5y that live in regions with high infection burden (14)
Resident in the study area (and planning to remain in the study area for the duration of the trial)
Ability and willingness to comply with the study protocol (daily intake of supplement and daily study visits with weekly finger prick)
Informed consent given by parent

4.2.2 Exclusion criteria

Participants with will be excluded, and treated or referred as necessary, if they have any of the following conditions:

- **Congenital anomalies/birth defects** (minor external congenital malformation is not an exclusion criteria)
- **Shock syndrome**
- **Currently experiencing moderate-severe diarrhoea** (defined as those diarrhoea episodes where (i) the child passes more than 5 loose or watery stools per day, (ii) there is blood or mucus in the stool (dysentery), or (iii) the child shows signs of clinical dehydration (assessed by the study nurse based on physical signs such as little or no urination, sunken eyes, and skin that lacks its normal elasticity), this diarrhoea will usually require referral to the study nurse and treatment (including ORS))
- **Chronic conditions:**

  Epilepsy
  Congenital heart disease/defect
  Nephrotic Syndrome & Chronic Glomerular Nephritis
  Diabetes
  HIV/AIDS
  Psychological/ mental retardation
  Chronic respiratory infections:
    - ✓ Chronic Bronchial Asthma/bronchopneumonia/aspiration pneumonia
    - ✓ Chronic suppurative otitis media (resulting from upper respiratory tract infection)
    - ✓ Bronchiectasis

    (children with lung abscess, pleurisy with effusion, and empyema can be enrolled if after treatment the condition resolves)

  Chronic viral hepatitis
  Cerebral palsy or motor disability
  Cancer
  Sickle cell/Thalassaemia
  Tuberculosis (including intestinal tuberculosis)
  Tropical sprue
  Primary immunodeficiency syndromes

Participants **currently participating in another study or currently taking iron supplements/multiple micronutrient supplements** will also be excluded.

Severely malnourished children (HAZ, WAZ, WHZ z-score <-3) will also be excluded and will be referred as necessary.

4.3 Withdrawal of participants

A study participant will be discontinued from the study intervention if:

- Hb concentration falls < 7 g/dL
- Any clinically-significant adverse event (SAE), laboratory abnormality, intercurrent illness, or other medical condition or situation occurs such that continued participation in the study would not be in the best interest of the participant
- Development of a chronic disease
- Participation in another study

Note that there is no formal withdrawal criteria for participants that develop moderate-severe diarhoea episodes since this is a primary outcome of the trial. However, children that show clear signs of infectious diarrhea, such as blood or mucus in the stool, will be provided with appropriate treatment and the causative agent for the infection will be determined using stool microbiology and the TaqMan Array Card (TAC) system (41).

Participants are free to withdraw from the study at any time without giving a reason.

Participants who discontinue study intervention due to severe anaemia (Hb<7 d/dL) will be offered standard-of-care treatment according to the national guidelines and will continue to be followed up by the study clinical team at the weekly clinics. These children will not be excluded from the data analysis and will be treated as treatment failures.

5 Study procedures and evaluations

5.1 Study schedule

5.1.1 Study sensitisation

The Management and Field team of MRCG Basse have already been engaged with the proposed trial and have offered their support. An initial mapping of the catchment area has also been carried out by the Field Coordinator and visits to some of the health clinics in the area were conducted. The Regional Health Team (RHT) and staff of the health facilities responsible for the catchment areas of Wuli and Sandu have been sensitised and approval from the Director of Health Services to proceed with the study in the North Bank of the URR has also been granted. The

study nurses will be based at the following health facilities in the Nortn Bank of the URR: Yorrobawol Health Center, Darsilami Community Health Post, Konkuba Community Health Post, Taibatu Health Post and Chamoi Health Center. A team lead by the local PI (Clinical Trial Coordinator), the Research Clinician and Field Coordinator will tour all the communities to sensitise them on the study during from April 2017 until recruitment for the last cohort starts. The community sensitisation will involve village to village sensitisation and a sentisation program in Yorrobawol, Darsilami and Chamoi where satellite village elders, Alkalos and the chiefs will be invited. The attached information sheet will be used as the basis for the sensitisations.

5.1.2 Screening and enrolment (baseline)

Young children in the participating communities will be identified using the enumeration/census data currently being collected by the study field team within the study catchment area (Figure 4). At screening, once mothers/guardians of the child have signed the informed consent form, the child will be physically examined by a study nurse and, if the child is considered as generally healthy (e.g. no fever, not severely malnourished), their height and weight will be measured and a finger prick blood sample will be collected for Hb and RDT testing at one of the health facilities supervised by the study nurses (Figure 5). If z-scores are >-3, 7 ≤Hb< 11 g/dL and the RDT is negative, then a small venous blood sample (1 mL in total divided into 0.5 ml EDTA and 0.5 ml serum blood collection tubes) will be collected to confirm the Hb levels (Medonic analyser) and determine serum ferritin (Cobas analyser). If 7 ≤Hb< 11 g/dL and serum ferritin < 30 ng/ml, the child will be eligible to enrol in the study. Children with Hb < 7 g/dL will not be enrolled and will be referred to the regional health centre for treatment according to national guidelines. Children with Hb ≥ 11 will not be enrolled as they don't need iron. Malaria positive children (positive RDT and confirmation by blood film) will not be enrolled and will be treated according to national guidelines. Severely malnourished children will also be excluded.

Screening will be divided into 3 cohorts, each recruitment period will be of approximately 1 month prior to enrolment into each of the sequential cohorts and we expect to screen ~ 500 children in each cohort or 50 children/day for 2 weeks, with 2 weeks left to get all screening results back from the lab.

Prior to enrolment (Day 0), which will be a maximum 4 weeks after the screening visit, eligible children will be taken back to the clinic for a finger prick to confirm absence of malaria by RDT and haemoglobin concentration by hemocue. Children will be called back in clusters of 58-60 on a Monday, Tuesday, Wednesday and Thursday. This is necessary before they can be enrolled and randomised to the study because of the time it takes to get all the screening results back from the labs for all children in that cohort (i.e. around 4 weeks). On the Friday of the same week, the list of all the children that remain RDT negative and with 7 ≤Hb< 11 g/dL will be sent to the

study statistician who will run the randomisation to one of the three study arms, stratified by Hb and age as described in 3.2.1., and send the list with the study IDs to the PIs that same day.

The week after, children will be taken back to the clinic again in the same cluster (i.e. either on the Mond, Tue, Wed or Thur as per their Day 0 cluster) and this will be their study Day 1 (see below).

We will enrol children in 3 cohorts (n=235 children each) that will run sequentially.

Each study cohort will be organised as follows. Each cohort will have 4 clusters (58-60 children per cluster), children in each cluster will be allocated to one of the study health facilities according to the child's compound proximity to each of the 4 study health facilities. Children in the first cluster will all have their study visits on a Monday, children in the second cluster will all have their study visits on the Tuesday, and the third cluster on the Wednesday and fourth cluster on the Thursday. Children in each cluster will also have their weekly check-ups at the study health facility on the same day of the week and would have had their Day 0 visit on the same day of the week.

5.1.3 Follow-up (study visits)

Each arm will include an intervention period (follow-up) of 12 weeks plus an additional active follow-up period of 4 weeks post intervention. As suggested by our grant proposal reviewers, we have included a placebo arm to allow us to fully evaluate IHAT effects, particularly to rule-out negative impact on diarrhoea episodes, the gut microbiome and compound-related bacterial infection, and to determine true treatment effect sizes with both IHAT and ferrous sulphate (for more details refer to Section 11.1.1.).

On study Day 1, we will take a photo of the child (with consent) and print and laminate a study ID card that we will ask the mother to keep safe during the study. This ID card will contain the photo of the child and their randomisation/study ID number. We will also provide the child with a wrist band showing the same study ID number so that there is no confusion regarding the identity of the study participants. This was considered necessary in the context of the North Bank of the URR since there is a poor health record system in place and most children do not have welfare cards. Then, we will collect a venous blood (total of 5 ml divided into 1 ml EDTA, 1 ml LH and 3 ml serum collection tubes) and a stool sample from the children (baseline samples). Blood sample collection will be done either before the first meal of the day or, in cases where this is impossible, at least 1 hour after the last meal (so that the more dynamic iron parameters, such as hepcidin and NTBI, are not influenced by the iron absorption from the previous meal). Stool samples may be collected by the mother at home on the morning of the study visit using the

toilet pots and disposable liners supplied by the study team for each child. The field worker will then aliquot the stool sample into the stool sample pots to be brought to the study clinic as soon as feasible after collection of the sample and will transport them to the clinic, when it is not possible to collect the sample from the child at the clinic. Stool samples can be collected within 7 days of the study time-point when it becomes difficult to collect the sample on the exact time-point day, i.e. if the child travels or is not able to pass stool.

On Day 1, we will also collect demographic and immunisation data and the morbidity questionnaire will be completed.

At the end of this visit, the mother will be encouraged to feed the child and once the child is settled, they will be given the iron supplementation or placebo. The treatment arm intervention will be administered directly into the child's mouth homogenised in 10 ml of a sugar juice drink. The iron or placebo powder doses will be added to the small amount of juice in a disposable plastic cup immediately before administration, this will be done by the field worker. This first administration of the study supplement/placebo to a child will be supervised by the study nurse and/or the study clinician to offer extra reassurance to the mother.

Highly trained and experienced field workers will be visiting all children every day during the 12 weeks supplementation period in order to administer the iron supplements or placebo and on these occasions they will check on the children's general health and actively look for signs of malaria and co-infections. If a child shows signs of these infections, the field worker will refer to the study nurse who will perform adequate tests and the child will be offered the appropriate treatment/referral to the next health center. Prior to study start, all field workers will receive appropriate training, provided by the study nurses and supervised by the study clinicians, on which AEs and SAEs to look out for and on when to refer the children to the study nurses for follow-up. Three times per week, morbidity data (including questions regarding fever, diarrhoea, vomiting, cough, malaria symptoms, any other illness, appetite and any mediation taken and assessment of body temperature) will be captured (as part of the CRF). Every week children will be taken back to the health facility in their cluster for a check-up and a finger prick to determine their malaria and Hb status. Hb will be determined with HemoCue. RDT testing will be used for malaria and children found with a positive RDT during the study will be further tested with a blood film and treated according to national guidelines. These check-ups will continue 4 weeks post intervention to follow-up on AE/SAEs.

Each field assistant (FA) will be responsible for 10-15 children within each cohort of 235 children, and will be supported by senior field assistants (SFA) and field supervisors (FS). The study will have a field team comprising of 21 FA, 4 SFA and 2 FS. All will be managed by the Field Coordinator (FC). Each SFA will be responsible for supervising the FA working in the study cluster

comprising 58-60 children. The FS will each be responsible for the communities in the Wuli and Sandu districts, respectively.

Every day during the intervention period of 12 weeks, the treatment arm interventions will be administered directly into the child's mouth homogenised in 10 ml of a sugar juice drink. The iron or placebo powder doses will be added to the small amount of juice in a disposable plastic cup immediately before administration, this will be done by the field worker. Before supplementation, the mother will be encouraged to feed the child so that the supplements are not ingested on an empty stomach (as detailed in 3.4.4).

On Days 15 and 85, the child will visit the clinic and another stool sample and another venous blood sample (total of 5 ml divided into 1 ml EDTA, 1 ml LH and 3 ml serum collection tubes) will be collected. Height and weight will be measured at day 85.

Any child where Hb falls below 7 g/dL during the follow-up study period will discontinue the study supplementation and will be referred to the next health centre for management and will be provided with iron supplements for 3 consecutive months according to national and WHO guidelines. These children will be treated in the data analysis as treatment failures. These children will continue to be followed up for adverse events by the field team but not receive any of the study treatments.

At each study visit (i.e. day 1, 15, 85) we will check the child's welfare card or ask the mother and note in the CRF if there was a vaccine in the past week, since some of the acute inflammatory markers we are measuring, particularly CRP, can transiently increase after immunisation (42, 43).

At the end of the follow-up period (12+4 weeks), the children in any arm who still have anaemia (Hb<11 g/dL) will be provided with iron supplementation for 3 consecutive months as per national and WHO guidelines.

5.1.4 Final study visit

The final study visit will be on day 113 (in the post-intervention follow-up period) when a finger prick blood sample will be collected and those children that remain anaemic will be offered iron supplementation for 3 consecutive months according to the national and WHO guidelines.

5.1.5 Early termination visit

An early termination visit may occur in this study because of a participant's voluntary withdrawal, trial team decision or at the discretion of the Data Safety Monitoring Board as described in Section 7. Apart from the safety evaluations, no other evaluations required for the final study visit will be done.

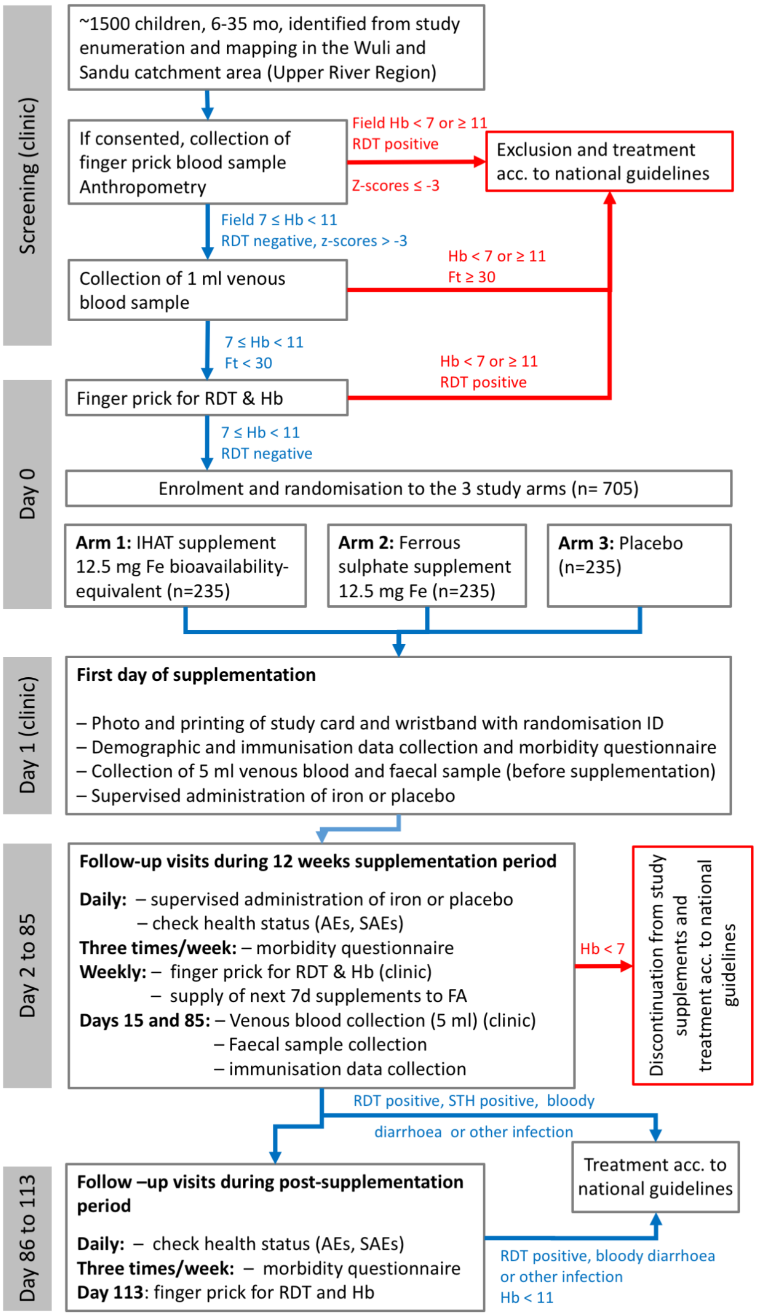

Figure 5. Study Flow Chart. Abbreviations: Hb, haemoglobin; Ft, ferritin; RDT, rapid diagnostics test; STH, soil-transmitted helminths; AE, adverse events; SAEs, serious adverse events; FA, field assistant; Fe, iron.

5.2 Study evaluations

This trial is powered to test non-inferiority of IHAT in relation to ferrous sulphate at correcting IDA, and in terms of diarrhoea to show superiority in relation to ferrous sulphate and non-inferiority in relation to placebo.

Non-inferiority of IHAT in relation to $FeSO_4$ at correcting IDA will be based on the response probability in the IHAT and $FeSO_4$ arms at the Day 85 time-point, i.e. the proportion of children in each arm that resolve iron deficiency and achieve a normal Hb or an increase of at least 1 g/dL in Hb after 12 weeks of the intervention.

Superiority of IHAT in relation to $FeSO_4$ in terms of diarrhoea will be based in both 'incidence density', i.e. the mean number of new moderate-severe diarrhoea episodes per child over the 12 weeks intervention period, and 'period prevalence', i.e. the proportion of children with at least one episode of moderate-severe diarrhoea over the 12 weeks intervention period, of moderate-severe diarrhoea.

Secondary objectives are to show that IHAT is non-detrimental with respect to enteric pathogen burden, the gut microbiome, and intestinal inflammation.

Please refer to Section 8 for more details on this analysis and power of the trial.

5.2.1 Clinical evaluations

Health status of the children at enrolment will be assessed through a physical examination and blood screening results by a study nurse in consultation with the study clinicians. During the study period, diarrhoea, hospitalisation and morbidity data will be collected three times per week in the field using a morbidity questionnaire.

5.2.2 Laboratory evaluations

Blood samples:

The analysis bellow will be performed in the ***venous blood samples*** collected on study days 1 (baseline), 15 and 85 and blood will be taken at the health facilities in the catchment area.

Sample collection will be either before the first meal of the day or, in cases where this is impossible, at least 1 hour after the last meal (so that the more dynamic iron parameters, such as hepcidin and NTBI, are not influenced by the iron absorption from the previous meal). Blood will be transported to the MRCG Basse laboratory for processing/analysis as soon as possible after collection.

Haemoglobin will be determined in EDTA anticoagulated blood (~500 µl) as part of a full haematology panel using the Medonic Haematology Analyser at MRCG Basse (lab was GCLP certified in October 2017).

Four serum aliquots will be frozen (-20°C) at MRCG Basse on the day of blood collection for the following analysis. One aliquot (500 µl) where serum ferritin, sTfR (soluble transferrin receptor), serum iron & total iron binding capacity (for calculation of transferrin saturation), CRP (C-reactive protein) and AGP (alpha-1-acid glycoprotein) will be determined using the fully automated biochemistry analyser (Cobas Integra 400 plus) at MRCG Keneba (lab was GCLP certified in October 2017).

Serum hepcidin will be measured in another aliquot (200 µl) using the DRG® Hepcidin 25 (bioactive) HS ELISA test kit and the Thermo Multiskan FC Microplate Photometer at MRCG Keneba.

Serum NTBI (aliquot of 300 µl) will be measured at King's College London (UK) by the new fluorescent beads method (44) that correlates closely with the traditional NTA method at lower TSAT but offers a crucial advantage over the NTA method because this can overestimate NTBI since NTA can remove some Fe bound to transferrin, particularly at TSAT above 70% (45, 46).

One serum aliquot (500 µl) and one plasma aliquot (500 µl) will be kept stored at -70°C for future analysis, particularly as new biomarkers of adverse effects due to iron emerge. This was a request from the funders. This may include DNA analysis and export of samples. We will obtain informed consent from the mothers/guardians for this to be the case within the study informed consent. Any future use would require PI, MRCG SCC and EC approval. This medium-long term storage will most likely be at MRCG Fajara.

In the ***finger prick blood samples***, Hb will be determined using a HemoCue 301 (5 µl) and an RDT (5 µl) will be performed every week at one of the study health facilities. In case of a positive RDT, a blood film (20 µl) will be prepared and read at the MRCG laboratory in Basse.

Stool samples:

Stool samples (~20 g) will be collected in toilet pots provided by the study team and lined with a disposable plastic liner at study days 1, 15 and 85 either at the participant's home on the morning of the study visit or at the health facilities where the venous blood sample will be collected. Stool samples can be collected within 7 days of the study time-point when it is not

possible to collect the sample on the exact time-point day. The samples will be kept in the toilet pots covered with the plastic liner in a cool place until the field worker process them. The field worker will aliquot the sample into the two labelled sterile stool sample tubes with scoop and screw lid as soon as possible after the child passes the stool. Approximately 15g of stool will be placed into a sterile Sarstedt stool collection tube and ~0.5g of stool into an OMNIgene GUT sample collection kit tube containing a DNA preservative that ensures that samples for microbiome analysis can be kept at room temperature. The field workers will avoid soil contamination and cross-contamination of the samples at all times. The samples will then be transported to the MRCG Basse laboratory. Once samples arrive at the MRCG Basse, the Sarstedt sample will be further divided into three aliquots: one aliquot will be used for helminth egg count and will be kept refrigerated until processing (~ 5 g) and the remaining two aliquots (~5 g each) will be frozen at -70°C, within 24 hours of collection. The OMNIgene sample will be kept at room temperature in the lab until DNA extraction, which should be done within 8 weeks for all samples at MRCG Fajara.

The national policy for anti-helminth treatment in The Gambia is that every child should receive de-worming tablets every 6 months, however, we still expect a proportion of the study population to be infected with soil-transmitted helminths (STH). Since STH infection can affect iron absorption and impact body iron status in young children (47, 48), we will perform a microscopic examination of a small sample of stool (sample kept refrigerated, not frozen) to determine the presence and number of STH eggs using the current WHO recommended Kato-Katz method, based on duplicate slides (49). Sample aliquots for this analysis will not be frozen but will be kept refrigerated at 4°C for up to 3 weeks as this has been shown to not cause significant changes in the egg counts and the morphology of eggs. Those children who test positive for STH infection (assessed on study days 1, 15 and 85) will be provided with anti-helminth treatment according to national guidelines but will not be excluded from the study.

The second Sarstedt sample aliquot (5 g) will be used for calprotectin analysis using the Calprotectin ELISA (EK-CAL, Bühlmann Laboratories). This analysis will be performed at the Basse Lab.

An additional aliquot (5 g) will remain frozen at -70°C for future analysis. This medium-long term storage will most likely be at MRCG Fajara.

The DNA will be extracted from the OMNIgene aliquot sample using the MoBio Soil extraction kit at the MRCG Fajara laboratory. Faecal DNA will be kept frozen at -70°C until it is used to prepare the PCR libraries before sending to the Sanger Institute in Cambridge (UK) for microbiome sequencing analysis. Microbiome analysis will be carried out by 16S rRNA sequencing (Illumina MiSeq). Minimum entropy decomposition (50) and targeted qPCR may be used to probe

specifically the enteric pathogens. A trained study scientific officer will be preparing the PCR libraries at MRCG Fajara following the Sanger protocols.

Finally, for those children presenting with bloody/mucus diarrhoea episodes, an additional stool sample will be collected at that point and we will use stool microbiology, at the Basse laboratory, and the TaqMan Array Card (TAC) system (41) (same used in the GEMS study), at the Fajara laboratory, to identify the causative infectious agent.

6 Safety considerations

A trial steering committee (TSC) has been setup in discussion with the Sponsor. Membership of this committee includes the co-PI (Dr Pereira), the MRC BSU biostatistician advisor (Dr James Wason), an independent Chair (Dr Margaret Pinder, MRCG Basse), a sponsor representative (Mr Pa Cheboh, Head of Operations at MRCG Basse), a community representative (local teacher), and the local PI (Dr Mohammad Ilias Hossain, MRCG Basse).

This trial will be overseen by a Data Safety Monitoring Board (DSMB) (the chair of this committee is Professor James Jay Berkley of the KEMRI-Wellcome Kilifi Institute)). The DSMB will be responsible for reviewing:

- the trial protocol (before the trial is started)
- safety data, including diarrhoea, other adverse events (AEs) and serious adverse events (SAEs), treatment failures due to Hb<7 g/dL, and the protection of the rights and well-being of the participants
- the overall progress of the study (recruitment rates, drop-out rates)
- protocol deviations/violations and non-compliance
- data analysis plan (at the last meeting of the DSMB)

In addition to the DSMB, an independent Local Safety Monitor will regularly review all AEs and SAEs. This review will focus particularly on AEs causality and reasons for losses to follow up, raising any concerns or issues that present immediate safety concern with the PIs for reporting to the DSMB, while protecting the confidentiality of the trial data and the results of monitoring.

6.1 Methods and timing for assessing, recording, and analysing safety parameters

The trial will be conducted according to Good Clinical Practice (GCP) principles. The DSMB will determine how they will monitor the data and safety interest of the participants. The DSMB will also determine how and the frequency of its meetings but we propose that the DSMB meets at least 3 times during the study intervention period to review AE/SAE rates in each arm and to have a concluding meeting towards the end of the study period. SAEs will be sent in real-time to the DSMB. The DSMB's responsibilities will be clearly defined in the DSMB charter, which will include how real-time SAE submissions are to be handled, i.e. mechanisims that would trigger ad-hoc meetings, etc. The DSMB charter has been prepared by the co-PI with Dr Jonas Lexow and has been reviewed by all DSMB members.

6.1.1 Adverse events

An adverse event (AE) is defined as any untoward or unfavourable medical occurrence in a human subject, including signs and symptoms which are temporally associated with the subject's participation in the research, whether or not considered related to the subject's participation in the research. Participants will be monitored for AEs on each scheduled follow up day. All symptoms or signs reported or observed will be assessed by the study Field Assistant and will be recorded as an AE after evaluation by the study nurse. Persistently low Hb will be considered as an AE and will be followed up.

Diarrhoea, defined as 3 or more loose or watery stools per day and no dehydration will NOT be considered as an AE (but will be recorded as part of the morbidity questionnaires), unless it requires observation by the nurse and treatment. However, moderate-severe diarrhoea, referring to those diarrhoea episodes where (i) the child passes 5 or more loose or watery stools per day, (ii) there is blood or mucus in the stool (dysentery), OR (iii) the child shows signs of clinical dehydration (assessed by the study nurse based on physical signs such as little or no urination, sunken eyes, and skin that lacks its normal elasticity), will usually require treatment (including ORS) and will be recorded as an AE.

6.1.2 Reactogenicity

N/A

6.1.3 Serious adverse events (SAEs)

A SAE is any AE that is life-threatening or results in death or requires hospitalisation as an inpatient or prolongation of inpatient hospitalisation or is a persistent or significant disability/incapacity. All SAEs will be investigated by the study clinicians.

6.1.4 Assessment of intensity of AEs

The study clinicians, i.e. the research clinician and the local PI (paediatric clinician and Clinical Trial Coordinator) with support from the clinical team in Basse, will assess the severity and intensity of the AEs and laboratory changes as defined below and record it into the AE form.
The local safety monitor is also a paediatric clinician independent from the research team and her primary responsibility will be to review individual and cumulative AEs and report to the local PI (Clinical Trial Coordinator).

**Grade**		**Description**
1	Mild	Awareness of sign or symptom, but easily tolerated
2	Moderate	Enough discomfort to cause interference with usual activity
3	Severe	Incapacitating with inability to work or do usual activity
4	Life-threatening	This grade will be considered as SAE

The term "severe" is often used to describe the intensity (severity) of a specific event (as in mild, moderate, or severe myocardial infarction); the event itself, however, may be of relatively minor medical significance (such as severe headache). This is not the same as "serious", which is based on the outcome or criteria defined under the SAE definition. An event can be considered serious without being severe if it conforms to the seriousness criteria; similarly severe events that do not conform to the criteria are not necessarily serious. Seriousness (not severity) serves as a guide for defining regulatory reporting obligations.

6.1.5 Assessment of causality

Every effort will be made by the study clinicians (the research clinician and the local PI), in discussion with the clinical team in Basse, and the co-PI (Dr Pereira), to explain each AE and assess its causal relationship to administration of the investigational product. This explanation will be based on the type of event, the relationship of the event to the time of trial intervention,

and the natural history of the underlying diseases, concomitant therapy, etc. The results will be documented on the AE form. The relationship of an AE to the investigational product will be assessed according to the definitions in the MRCG Unit's SOP:

Unrelated

No temporal association with the study supplementation/drug; related to other aetiologies such as concomitant medications or conditions, or participant's known clinical state.

Unlikely

Temporal association with the study supplementation is improbable, but not impossible; other aetiologies such as concomitant medications or conditions, or participant's known clinical state provide plausible explanations.

Possible

Less clear temporal association; event could also be explained by alternate aetiology (clinical state, environmental or other interventions).

Probable

Clear-cut temporal association, with improvement upon drug withdrawal and not reasonably explained by alternate aetiologies (patient's known clinical state, environmental, or other interventions).

Definite

Clear-cut temporal association, with a positive re-challenge test or laboratory confirmation.

The mothers/guardians of participating children will be instructed to contact the field assistant or a member of the study team, should the child manifest any signs or symptoms they perceive as severe during the period extending from performance of the first trial procedure to the end of the study.

All findings observed or reported from the day of the first administration of the investigational product will be recorded on an AE Form electronically by the team. Whenever possible, AEs will be documented in terms of a diagnosis or syndrome rather than multiple symptoms that are clear manifestations of the same diagnosis/syndrome. In case signs and symptoms are reported by

the participants, a medical diagnosis will be obtained by the study clinicians. If a diagnosis cannot be obtained then each sign or symptom will be recorded as separate events.

The action taken (e.g. discontinuation of investigational product, withdrawal of the participant from the trial, requirement of concomitant medication or treatment, others) will be recorded on the AE Form. If hospitalisation or its prolongation is required this will be reported as a SAE.
All AEs will be followed until resolution of the event and/or the end of the trial. The outcome will be assessed as follows:

- Resolved
- Resolved with sequelae
- Ongoing
- Death
- Lost to follow up

Treatment of any AE and SAE will be recorded in the appropriate section of the CRF.

6.1.6 Serious Adverse Reaction (SAR)

SAR is any SAE deemed to be **probably related or definitely related** to the study supplementation (as defined above).

Trial recruitment will be stopped with immediate effect if any child has a SAR to any of the iron supplements (or placebo) until review by the DSMB. Follow-up of children already enrolled in the study will continue as planned pending the review of the DSMB, as indicated in the DSMB Charter.

6.2 Reporting procedures

The Local Safety Monitor (LSM) will prepare regular reports concerning AEs and SAEs for submission to the local PI. The local PI (Clinical Trial Coordinator), shall report all SAEs (defined as in 6.1.3) without filtration, whether or not related to the trial intervention, within 24 hours of becoming aware of the event to the Sponsor, through the MRCG CTSO (dct@mrc.gm), and the LSM. The complete SAE report form will be sent to the DSMB members as soon as the full detailed report is available, usually within 7 days of the study team becoming aware of the event.
If the SAE is related to the trial intervention, the Ethics Committee will be notified within 7 calendar days if fatal or life-threatening, and all others within 15 calendar days.
All SAE will be reported to the Medicines Control Agency in accordance with the applicable requirement.

The minimum information required for this initial SAE report is:

- Trial number and (short) title
- Participant's ID
- Date and time of onset
- Description of the event (clinical history, associated signs and symptoms)
- Reporter's name

The information about which intervention was administered (unblinding) will only be provided at the request of the DSMB.

The local PI will not wait for additional information to fully document the event before notifying the Sponsor and the LSM. The report is then to be followed by submission of a completed SAE Report Form as soon as possible, detailing relevant aspects of the SAE in question. All actions taken by the local PI and the outcome of the event must also be reported immediately.

For documentation of the SAE, any actions taken, outcome and follow-up, the SAE Report Forms will be used. All follow-up activities have to be reported, if necessary on one or more consecutive SAE report forms in a timely manner. All fields with additional or changed information must be completed. Hospital case records and autopsy reports, including verbal autopsy, will be obtained where applicable.
At the request of the DSMB, only final and complete SAE Report Forms, including follow-up actions, will be sent to notify them of an SAE. This notification will occur usually within 7 days of completion of the final report.

6.3 Safety oversight

Safety oversight shall be provided by a DSMB who will provide independent advice as stipulated in Section 6 above. An independent Local Safety Monitor will review all adverse events more regularly and report regularly to the PIs and DSMB.

7 Discontinuation criteria

7.1 Participant's premature termination

Mothers/guardians of participating children have the right to stop their children's participation in the study at any time without giving a reason and this will not affect the medical care that would normally be received. The trial team may also withdraw a participant from the study if deemed necessary at any time taking in to consideration the reasons mentioned below. The reason for a participant's premature termination will be documented on the appropriate page of the CRF and specified which of the following possible reasons were responsible for the premature termination:

- Serious Adverse Event
- Adverse Event
- Participant's consent withdrawal
- Development of withdrawal criterion
- Protocol deviation
- Migrated/moved from the study area
- Lost to follow-up

A 'lost to follow-up' is any participant who completed all protocol specific procedures up to the administration of the investigational product or intervention, but was then lost during the study period to any further follow-up, with no safety information and no efficacy endpoint data ever became available.

In case the participant decides to withdraw participation or consent during the study, we will not work on participant's samples without permission, but any information already generated from the samples will be kept and used. The study clinician may also ask for tests for the participant's safety. The PI will inquire about the reason for any withdrawal and follow-up with the participant regarding any unresolved AEs.

For withdrawn participants no specific data will be collected. Our sample size takes into account a dropout rate of 15% but this will be monitored closely and if the observed dropout rate increases above this level we will replace subjects to ensure we reach our target sample size of n=600 completed.

7.2 Study discontinuation

The rules for study termination will be set by the DSMB at their first meeting.

8 Statistical considerations

8.1 Sample size determination

This section was prepared with the expert input of Dr James Wason, MRC Biostatistics Unit, Cambridge. Dr James Wason is a Programme Leader Track senior statistician who primarily works on statistical methodology for novel clinical trial designs and is one of the few UK experts in design and analysis of adaptive clinical trials. Dr Wason is a long term collaborator of Dr Pereira for the design of the IHAT trials and is a collaborator in the proposed trial. He has provided advice on the trial design and conducted the trial power modelling and sample size calculations presented in this Section. Dr Wason will continue to provide advice on the trial design to the PIs and will supervise an MRCG statistician in the data analysis corresponding to the four trial hypothesis (below). Dr Wason will be a member of the TSC and will review and approve the data analysis plan for the trial, but it will be the responsibility of the MRCG statistician (Dr Nuredin Mohammed supervised by Dr David Jeffries) to develop the initial drafts of the data analysis plan together with the study PIs.

There are four null hypotheses in the study:

1) non-inferiority of IHAT compared to ferrous sulphate for efficacy (in terms of Hb and iron deficiency correction: i.e. IDA) response probability (or prevalence). In this case we are testing the null hypothesis that: response in the IHAT arm minus the response probability in the ferrous sulphate arm is less than or equal to – 0.1. We define 'response' for IDA as correction of iron deficiency and either achieving a normal Hb or an increase of at least 1 g/dL after 12 weeks of iron supplementation.

2) superiority of IHAT compared to ferrous sulphate in terms of incidence density of diarrhoea. Here the null hypothesis is that the mean number of new episodes in the IHAT arm is greater than or equal to the mean number in the $FeSO_4$ arm.

3) superiority of IHAT compared to ferrous sulphate in terms of prevalence of diarrhoea. Here the null hypothesis is that the prevalence of diarrhoea in the IHAT arm is greater than or equal to the prevalence in the $FeSO_4$ arm.

4) non-inferiority of IHAT compared to placebo in terms of prevalence of diarrhoea. Here the null hypothesis is that the prevalence of diarrhoea in the placebo arm minus the prevalence in the IHAT arm is less than or equal – 0.1.

Because this is a pilot study we do not formally adjust for multiple testing due to the four hypotheses. This is because any significant results will be tested again in a pivotal study.

The trial is powered to show non-inferiority of IHAT relative to ferrous sulphate for the 12 weeks efficacy response probability. At the 12 weeks time-point (i.e. Day 85), the estimated odds ratio between the IHAT and $FeSO_4$ arms will be calculated, together with a 90% one-sided confidence interval. If the lower limit of the confidence interval for this difference is above 0.583 (equivalent to a 10% absolute difference if $FeSO_4$ response rate is 0.3), then non-inferiority of IHAT in terms of efficacy will be declared.

Because many trials conducted in Africa and other developing countries with the gold standard ferrous sulphate or ferrous fumarate supplementation still fail to provide anaemia resolution in interventions shorter than 6 months, we have considered here that an increase of 1 g/dl in Hb after 3 months is an indication of efficacy, even though we accept that this is not clinical efficacy. We have based our assay sensitivity from data published with relevant iron supplement studies in children available in the literature (3, 7, 52). We plan, however, to use the data collected in the proposed study to conduct an exploratory analysis to test for assay sensitivity. These data will be very useful so that adequate treatment effect sizes can be used to power any future studies, in particular a pivotal trial, both for ferrous sulphate and IHAT supplementation effects.

Due to this trial being a pilot study, any significant results will be re-tested in the pivotal 'full' trial (BMGF full award). Thus, we considered a larger than traditional type-I error rate of 10%. Assuming that 30% (i.e. a response probability of 0.3) of the children in both arms will resolve iron deficiency and either achieve a normal Hb or an increase of at least 1 g/dL after 12 weeks of iron supplementation (3, 7, 52), then a sample size of 200 in the IHAT arm and 200 in the ferrous sulphate arm will provide 89% power to show non-inferiority with a non-inferiority margin of 0.1 (0.583 on the odds-ratio scale). In other words, the trial will have 89% power (at a 10% one-sided significance level) to show that the lower boundary of the confidence interval of the odds ratio between IHAT and ferrous sulphate is more than 0.583 at 12 weeks.

We have based our calculation above on assay sensitivity data taken from other studies with iron supplements reported in the literature, but we will also perform an exploratory analysis to test for assay sensitivity, and this information will be used to power the potential future pivotal study.

We have considered two options for modelling the diarrhoea outcome: 1) 'period prevalence', i.e. the proportion of children with at least one episode of moderate-severe diarrhoea over the 12 weeks intervention period; 2) 'incidence density'. i.e. the number of new diarrhoea episodes per child over the 12 weeks intervention period. The modelling of how the power of the trial varies according to assumptions for each of these outcomes is detailed below.

The trial sample size will also provide 90% power to show superiority of IHAT in terms of moderate-severe diarrhoea 'incidence density', assuming that IHAT provides a 20% reduction in mean moderate-severe diarrhoea 'incidence density' relative to ferrous sulphate; and 90% power to show superiority of IHAT in terms of 'period prevalence' of moderate-severe diarrhoea, assuming that 25% of children will develop moderate-severe diarrhoea episodes with ferrous

sulphate and 15% will have moderate-severe diarrhoea with IHAT over the 12 weeks intervention. For the comparison between the IHAT and placebo arms, the trial is powered to show non-inferiority of IHAT relative to placebo, for example, the trial will have 93% power for the moderate-severe diarrhoea 'period prevalence' outcome. All the assumptions for these calculations were based on data from studies conducted in The Gambia for the same age group, please refer to the extra information below for further details.

As part of the rationale for the study is to provide information for a pivotal study, we also examined the precision that the sample size would provide on the confidence interval width of the outcomes considered. Since the width of the confidence interval is highly dependent on the proportion in the control group as well as the true odds ratio, we consider several different values in the table below.

Binomial proportion in control group	True odds-ratio	95% confidence interval width
0.1	0.5	0.85
	1	1.38
	1.5	1.91
0.3	0.5	0.49
	1	0.88
	1.5	1.27
0.5	0.5	0.42
	1	0.80
	1.5	1.21

The power calculations for some of the secondary outcomes are also presented in more detail below. Briefly, the trial (n=200 per arm) will have over 85% power to detect significant differences between all the arms in terms of Enterobacteria, NTBI and calprotectin.

Taking into consideration a non-completion rate of 15% (this is based on data from previous studies in The Gambia), we expect to **enrol 705 children in the study**.

Detailed calculations of power for the diarrhoea and secondary outcomes:

I. POWER OF THE TRIAL FOR THE DIARRHEAL OUTCOME.

1. Period Prevalence (i.e. proportion of children with >= 1 episode of moderate-severe diarrhoea over the 12 weeks intervention period)

Based on previous data from the case-control GEMS (Global Enteric Multicentre Study) for the Basse (Upper River BHDSS) region in the Gambia, the background point prevalence of moderate-severe diarrhoea in children under 3 years of age can be as high as 14%, whereas data from the same region in a previous survey HUAS (Health Care Utilization and Attitudes Survey) showed that this number can be as high as 20%.

For our calculations, we chose to consider 15% as the proportion of children with at least one episode of diarrhoea in the IHAT group over the 12 wk. intervention period, which we hypothesise would be similar to the background period prevalence. This prevalence is similar to that found in other iron intervention studies for the no-iron groups (7, 8).

The graph below shows the power of the trial (200 patients per arm at a 10% one-sided type I error rate) to detect a significant difference between IHAT and ferrous sulphate (FS) as the ferrous sulphate diarrhoea period-prevalence varies:

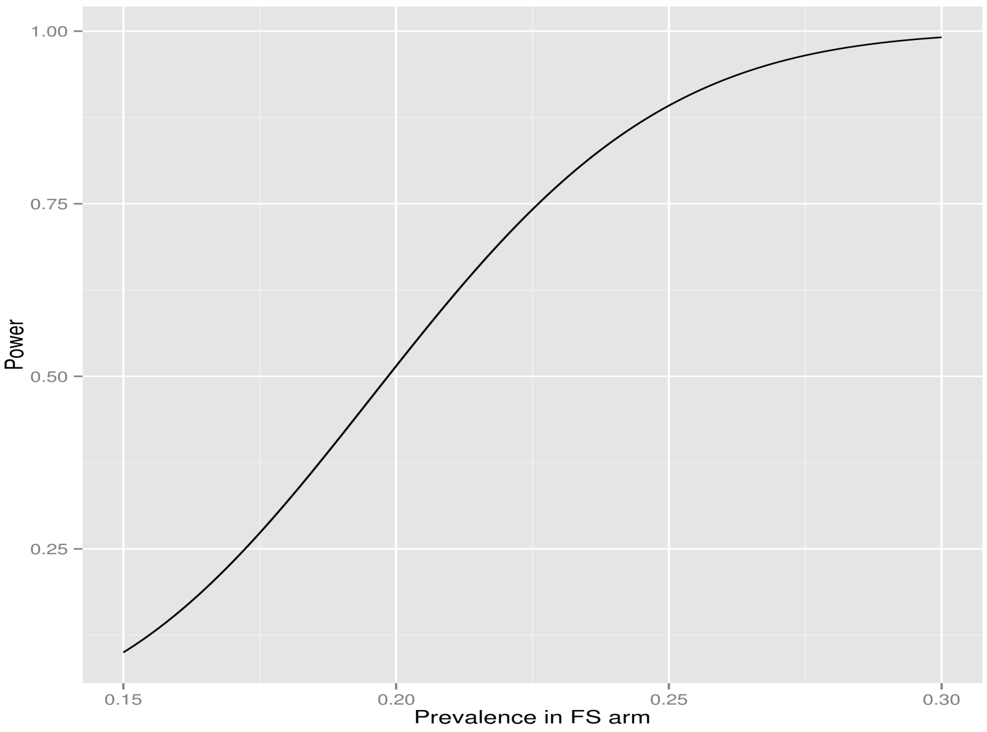

For a period prevalence of 0.15 in the IHAT arm and 0.25 in the ferrous sulphate arm, the power of the trial is around 90%. We note that previous data for ferrous sulphate suggest that 25% is a reasonable figure for the period prevalence: for example Zlotkin et al 2013 reported this to be 16.8% in the iron group and Jaeggi et al 2014 reported 27.3%. Furthermore, for the Gambia specifically, preliminary data from the 84 days period of the current HIGH study in the Soma region shows that, during the 84-days of the intervention, moderate-severe diarrhoea was recorded at least once as an adverse event in 36% of the children.

For comparing placebo and IHAT, we used a non-inferiority approach. With 200 patients per arm, we would have 93% power to show non-inferiority of IHAT with a 10% non-inferiority margin (at a 10% one-sided type I error rate). This means that, if the true diarrhoea period prevalence in the IHAT and placebo arms is 0.15, this sample size has 93% power to show that the upper confidence interval of the difference in prevalence between these two arms is less than 10%.

2. Incidence density (i.e. number of moderate-severe diarrhoea episodes per child over the 12 weeks intervention)

Preliminary data from the 84 days data collected in the HIGH study in the Soma region in the Gambia suggest that the incidence density of moderate-severe diarrhoea is 1.28 episodes per child over the 84-days study period. Using the same type I error rate and sample size as above, the graph below shows the power of the trial to detect a significant difference between arms as the rate ratio varies.

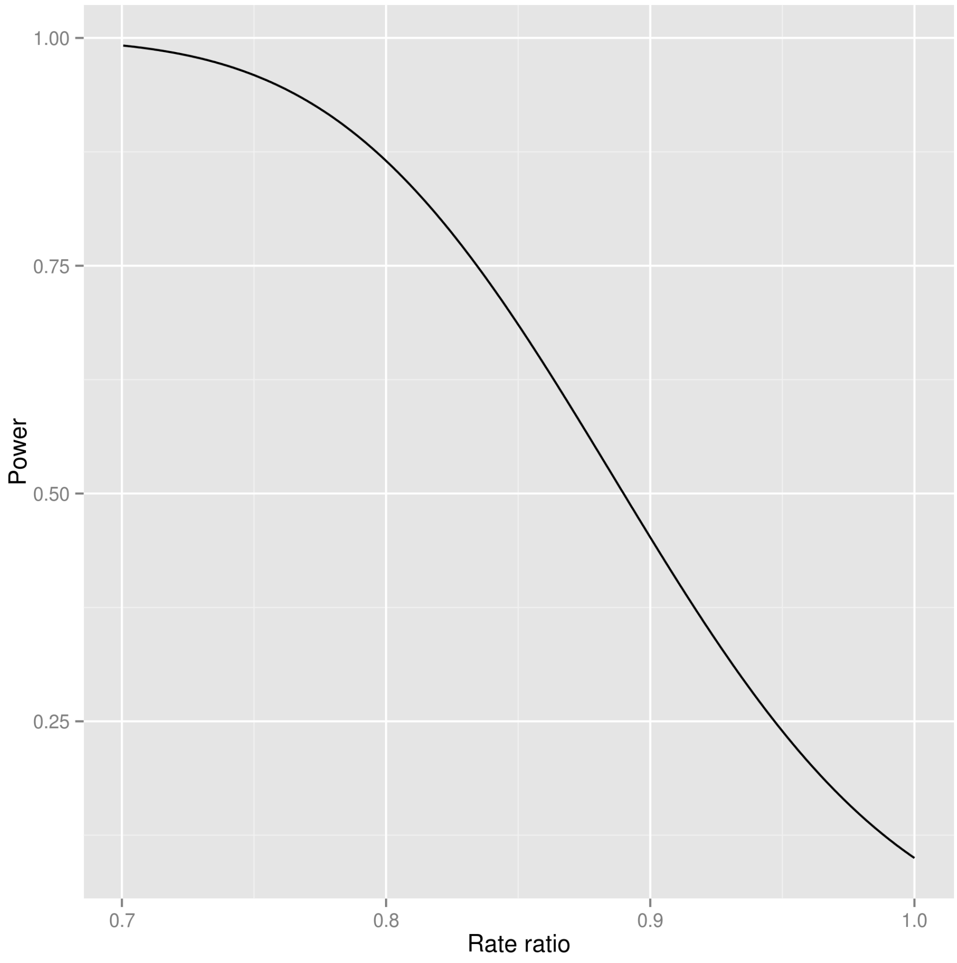

For a rate ratio of 0.8 (i.e. IHAT provides a 20% reduction in mean incidence density of moderate-severe diarrhoea relative to ferrous sulphate), the power is almost 90%.

II. POWER of the trial for the secondary endpoints

1) Microbiome (Enterobacteria log10 counts):

We based our calculation on the data for Enterobacteria (the genus that contains most enteropathogens including *E. coli*) reported in Zimmermann 2014 study conducted with 4 months supplementation (8): with iron 8.9 (0.3) and without iron 8.0 (0.4). Taking into consideration that in this study the analysis combined the groups with 2.5 mg Fe and 12.5 mg Fe MNP, then the combined sample size implies an individual standard deviation of 3 for the Enterobacteria and assuming a 0.9 difference between the groups and a superiority margin of 0.2 (i.e. an absolute difference in Enterobacteria between the IHAT and ferrous sulphate arms of - 0.2), the sample size of 200 children per group would give us 85% (at 10% one-sided significance level).

2) Calprotectin

Again, we based this calculation in the Zimmermann 2014 study (8). Calprotectin concentration with iron: 248.9 +/- 2.2 μg/g and without iron: 102.5 +/- 2.2 μg/g in the 12.5 mg MNP. Here the individual SD should be around 11.8 considering the study sample size in the 12.5mg Fe MNP group, which would mean that with 200 per arm we could detect an absolute mean difference of 3 with 90% power (10% one-sided sig level) and with 50 per arm we would be able to detect a mean difference of 6.1.

3) NTBI

For this calculation we have used the 'normal serum' data provided by Robert Hider with the same method we intend to use in the study (44). Taking the values reported in the paper for 'normal serum' of 0.24 +/- 0.15 (mean +/- SD), n=9 range 0.04 – 0.41, the individual standard deviation would be 0.45. There is no published data with this method for NTBI after an iron dose so we have assumed we would like to detect a mean difference of 0.5 between the IHAT and the ferrous sulphate arm, which would mean that with 50 per arm the power would be more than 90%. To be able to detect smaller differences with this method we would need to increase sample size.

8.2 Statistical analysis

The data analysis plan will be fully developed prior to database lock and will be done with the expert input from Dr James Wason of the MRC Biostatistics Unit in Cambridge. Briefly, for the primary analysis, we will fit a logistic regression to the efficacy outcome data, adjusting for the strata created by age and Hb level groups prior to enrolment. The odds ratio for effect of IHAT relative to ferrous sulphate will be estimated, and if the lower 90% one-sided confidence interval is above 0.583 (equivalent to the 10% non-inferiority margin above), non-inferiority will be declared for IHAT. The diarrhoea endpoint will be analysed in a similar way, but tested for superiority (i.e. if the one-sided p-value for the Wald test of the effect of IHAT is less than 0.1) for the comparison between IHAT and ferrous sulphate. For the primary non-inferiority hypothesis we will conduct per-protocol and intention-to-treat analyses and for the superiority hypotheses we will conduct intention-to-treat analyses.

The secondary endpoints (calprotectin, NTBI etc) will be tested using linear regression with the same covariates. Morbidity data will be analysed using multiple regression analysis controlling for possible confounders.

In relation to the microbiome data analysis we will work together with our collaborators in the Sanger Institute and use the most up to date bioinformatics methodology available at the time of data analysis to investigate the impact of treatment arm and time in the composition of the gut microbiome. Since this analysis will only occur in late 2018/early 2019, it is premature to decide the strategy for data analysis now as this is a fast moving field with new and better bioinformatics tools becoming available every couple of months. The Sanger Institute works very closely with the European Bioinformatics Institute (EBI) (housed in the same campus) to ensure they use the very latest tools in their microbiome analysis.

9 Data handling and record keeping

9.1 Data management and processing

All protocol data will be captured in Case Report Forms (CRF) that will be completed for each included participant using electronic data capture. On the CRF, a reference to the source document will be provided. Instructions for completing all forms, including the CRF, used in the study will be developed.

The following data will be recorded: date of informed consent, personal data (ID, initials, date of birth), socioeconomic data, height and weight, information on health status and regarding participation in other studies, date and time of all venous and finger prick blood collections, date and time of all faecal sample collections, date and time of supplement administration, data on morbidity, lab results.

All trial data will be stored and managed within a clinical database built on the REDCap platform, an application specifically designed to collect and store clinical trial data and customised for Electronic Data Capture (EDC) in the field. Where appropriate, data entry fields will incorporate appropriate range checks. At the point of data collection, data will be entered directly into tablet computers as already done in the MRCG studies, and uploaded to the main server either immediately, or at least daily when network signal is weak. Data export options from the clinical database include CDISC ODM XML, a vendor neutral, platform independent format for interchange and archive of data collected in clinical trials. This should ensure sharing and long term validity of data. This data management system is a fully supported, externally validated (GCP compliant to 21 CFR part 11) clinical database.

Prior to the first participant enrolment a detailed trial specific data management plan (t_DMP) will be created, reviewed and signed off by the Data Manager and the TSC. This t-DMP will outline in detail the specific procedures that will be used to ensure high quality data will be delivered for statistical analysis and future use. The t-DMP will complement the trial risk assessment and statistical analysis plan. Review of data collection tools against the protocol will occur prior to database build by the Data Manager to ensure all data is being captured, at the correct time points, and in a format conducive to the proposed statistical analysis. Site based staff will be fully trained in conducting any study assessments and subsequent completion of the CRF and a Site delegation log will be maintained. Data entry staff will be trained in the use of OpenClinica. Principles of good clinical practice will be adhered to and all training will be documented. To ensure standardisation of processes, standard operating procedures with respect to trial management, quality assurance, data management, IT & security, and statistics will be adhered to.

9.2 Source documents and access to source data

The local PI will maintain appropriate medical and research records for this study in compliance with the principles of good clinical practice and regulatory and institutional requirements for the protection of confidentiality of participants. The study team members will have access to records.

The authorised representatives of the sponsor, the ethics committee(s) or regulatory bodies may inspect all documents and records required to be maintained by the investigator, including but not limited to, medical records (office, clinic, or hospital) for the participants in this study. The clinical study site will permit access to such records.

9.3 Protocol deviations

A protocol deviation (PD) is any noncompliance with the clinical trial protocol, good clinical practice (GCP), or other applicable regulatory requirements. The noncompliance may be either on the part of the participant or the investigator including the study team members, and may result in significant added risk to the study participant. As a result of a deviation, corrective actions will be developed and implemented promptly.

If a deviation from, or a change of, the protocol is implemented to eliminate an immediate hazard(s) to trial participant without prior ethics approval, the PI or designee will submit the implemented deviation or change, the reasons for it, and, if appropriate, the proposed protocol amendment(s) as soon as possible to the sponsor for agreement and the relevant independent ethics committee (IEC) for review and approval.

The local PI or designee will document and explain any deviation from the approved protocol on the CRF, where appropriate, and record and explain any deviation according to the Unit's SOP.

10 QUALITY CONTROL AND QUALITY ASSURANCE

Quality control will be applied to each stage of the study. We will work together with the Sponsor, through their Quality Management team (Mrs *Yai Louise Ndure-Bensouda*) and Research Governance teams (led by Dr Jonas Lexow) to finalise a plan for the trial quality monitoring.

It will be the responsibility of the local PI or designated trial team member to ensure that all source documents and CRFs are reviewed for accuracy and completeness. Any correction will be accurately accounted for.

Finger prick and venous blood collection will be done by the trial nurses. Collection of all samples will be recorded on the CRF.

All Field assistants and their supervisors including trial nurses will be fully trained. Selected participants from the RCH teams will also be trained. Further refresher training will be conducted as the trial progresses. Weekly field team meetings and monthly meetings of the entire trial team will be convened in order to discuss all problems and lessons from the trial.

We will also work with the Quality and Laboratory Management teams to write a detailed Analytical Plan for the trial and appoint a study team member (likely one SO) as the Analytical Project Manager who will be responsible for overseeing and ensuring the quality of the laboratory activities for the trial.

10.1 Study monitoring

A risk-based monitoring plan will developed with the MRCG Clinical Trial Support Office prior to study starting. This will describe monitoring frequencies and content of Site Initiation, Interim Montoring and Close-out Visits.

11 Ethical considerations

This study is conducted in accordance with the principles set forth in the ICH Harmonised Tripartite Guideline for Good Clinical Practice and the Declaration of Helsinki in its current version (see appendix), whichever affords the greater protection to the participants.

11.1 General considerations on human subject protection

Study participants will be young children and the study protocol will be explained to their mothers/guardians orally in the presence of an independent witness in case they are illiterate or in writing. No children will start any study specific procedure before informed consent is obtained. The study investigates iron supplementation in doses specifically recommended for this age group by the WHO. Participants will not get any remuneration but will have free basic medical care for the duration of the study.

11.1.1 Rationale for participant selection

Young children, together with pregnant women, are the two most affected population groups in relation to anaemia and iron deficiency anaemia, and the groups for which better iron supplementation strategies are required.

In pregnant women the main problems of current iron supplements are the gastrointestinal side-effects such as nausea, abdominal cramps, heartburn and constipation (15), which affect compliance with treatment. Our future aim is to be able to also conduct a trial in pregnancy to test the hypothesis that IHAT would not be associated with these side-effects in the pregnant women. We are actively seeking funding for this.

However, in our opinion the most pressing need in relation to iron supplementation in developing countries is to find an alternative iron supplement for use in young children living in areas at risk of enteric infection. As mentioned in the Background Section, RCTs with current iron supplementation in nearly ten thousand young children living in developing countries have consistently shown that these are associated with increased infection including bloody diarrhoea (3, 6-8) and detrimental changes to the gut microbiome and gut inflammation (8, 9), further increasing the burden from enteric infection and environmental enteropathy (i.e. persistent gut damage and inflammation that leads to malabsorption), which is a major cause of growth failure in children in resource-poor environments (10, 11). Furthermore, it appears that these effects are much more relevant in those resource-poor countries where enteric infection risk is higher and in the pre-school age group since in South-African 6-11 years old children with a low enteropathogen burden, iron supplementation did not significantly affect the dominant bacterial groups in the gut or gut inflammation (53). The specific problem this proposal seeks to address relates to these effects of oral iron supplements for treating anaemia in young children. Hence, why we propose to conduct the trial in young children living in the most deprived areas of The Gambia where the risk for enteric infection is higher.

More than 70% of all children under 5 y. in The Gambia will be anaemic and in most cases this will be due to iron deficiency. The consequences of anaemia and iron deficiency include impaired neurocognitive development and immunocompetence leading to substantial loss of human potential.
Combating anaemia due to iron deficiency is a challenge due to the potential negative side-effects of iron when given to people with infections, as is extremely common in young children in The Gambia.

Specifically, we will be addressing the fact that current iron supplements increase risk of moderate-severe diarrhoea in these children, particularly in settings where risk for enteric infection is high, as we mentioned above. In the 21st century, we simply cannot accept the current view that increased risk of infectious diarrhoea in young children is normal collateral damage of oral iron supplementation in populations at high risk of enteric infection. This is a

problem of current iron supplements and we believe that IHAT will work differently for all the reasons explained in the Rationale Section of this protocol. IHAT may also offer other advantages in relation to conventional iron supplements in relation to efficiency of absorption of consecutive daily doses and systemic infection risk as explained in the Rationale Section of this protocol.

Finally, we wish to note that we believe that IHAT's main target population group, where IHAT's benefit will really outweight its added cost over current iron supplements, are young children from resource-poor countries. This is because the main advantage of IHAT over other iron supplements relates to its intestinal safety and, as we mentioned above, young children living in areas of enteric infection risk are the main population group affected by the negative effect of current iron supplements on intestinal infection (including bloody diarrhoea) and inflammation (contributing to environmental enteropathy). Essentially, if IHAT works in the proposed trial, where it will be tested in the population group that is more responsive to the adverse effects of iron supplementation (i.e. young children at risk of enteric infection), then it will work in any other population group. But the reverse is not true, i.e. if IHAT works in adults it does not mean it would work in children, and similarly if IHAT works in older children it does not mean it would work in the young children group, since these are the subjects with the most immature gut where the microbiome is not stable and where the mucosa is more susceptible.

This is why it is crucial to conduct the trial in the young children living in some of the most deprived and infectious communities in the Gambia. Conducting the study in adults would not provide us with clinically-meaningful information about the impact of IHAT on enteric infection and diarrhoea risk, since this effect is not commonly observed in adults (possibly due to the immature gut microbiome and mucosa in young children) with iron supplementation, and would just delay further investment and the authorisation of IHAT for use in children, which would be unethical in our view. As such, we follow the recommendation of the European Medicines Agency to study medicines intended to be used in children in a paediatric population rather than inferring the information and dosages from adult trials (Regulation (EC) No 1901/2006). We appreciate that children are a more vulnerable group for the first Phase II trial of IHAT or indeed any other form of iron supplementation, but they are also the group that will most benefit from a better iron supplement. The health of all children in our study will be closely monitored as will all adverse events including all diarrhoea episodes, malaria and other co-infections. Therefore, ethically we will not be subjecting children to any unnecessary risk or, in fact, any more risk that when current iron supplements are used as per national guidelines. In the worst case scenario, IHAT will be the same as currently used ferrous suphate or fumarate in terms of diarrhoea and other adverse events.

This study will provide the first Phase II trial data with IHAT and will enable us to obtain high-quality clinical data for its safety and efficacy in correcting IDA in children, the population group most in need of an alternative oral iron supplement. If IHAT is successful in the trial, these data will provide the evidence needed to encourage further investment so that IHAT can be implemented as a novel iron source for use in micronutrient intervention strategies aimed at children and women living in resource-poor countries and, hence, reduce the global burden of IDA.

11.1.2 Rationale for use of a placebo group

We have thought very carefully about the lower threshold of Hb inclusion for all study arms and we wish to note that we are not contravening the current national guidelines, since there is no mandatory iron supplementation for this age group in the Gambia. All our prior iron intervention trials in The Gambia, Kenya and Tanzania, have used the Hb 7-11 g/dl inclusion criteria. The rationale being that Hb below 7 g/dl is the current WHO cut-off for severe anaemia in children under 59 months of age (WHO/NMH/NHD/MNM/11.1), and these children would be the ones presenting with more visible symptoms of anaemia and most likely to be automatically given oral iron treatment.

Since recent trials have demonstrated a possible detrimental effect of iron supplements in pre-school age children, there is now a strong ethical rationale for a 'no-iron' control group (i.e. placebo). It is crucial to have this placebo control group in our study, not only for determining background 'side-effects' but also, and importantly, for assessing the true treatment effect size of current iron supplementation (i.e. ferrous sulphate) in Hb levels. There have been numerous iron supplementation studies in young children in resource-poor countries, including in the Gambia, that have failed to show any decrease in anaemia prevalence with iron supplementation, and we feel it is necessary that we determine in this study if iron supplements are preventing Hb from falling further in relation to a 'no iron' (or placebo) group, which we consider to be an important positive effect, even when anaemia prevalence may not decrease for this age group due to the high demands for iron during fast growth.
It is also important to have a placebo group in this study so that we can perform an exploratory analysis and test for assay sensitivity and to determine treatment effect size with IHAT so that these data can be used to power future studies.

In the proposed study we will include only children with mild-moderate anaemia, and we will exclude any children with severe anaemia, who would be most likely identified and referred for iron treatment according to the national policy in The Gambia. The children with Hb between 7 and 11 g/dl would probably not be identified as anaemic had they not been offered screening through our study and would not have been provided with oral iron supplements. Within this

group, the children with Hb between 7 and 8 g/dl are the ones most in need of an alternative oral iron treatment, one that is both effective and safe. In any case, as we mentioned above, we will monitor very closely the Hb levels in all study arms and discontinue the study intervention and treat according to the national guidelines any children where Hb falls below 7 g/dl at any point during the study. These children will continue to be followed-up by the study clinical team at the weekly clinics. Furthermore, at the end of the study, any child who remains anaemic will be offered the standard iron supplementation according to national policy. This means that we will only be delaying treatment for the children in the placebo group by 4 months (unless they become severely anaemic during the study, in which case they receive treatment immediately).

11.1.3 Evaluation of risks and benefits

There are risks associated with a large intake of iron supplements especially in areas of malaria endemicity. The dose of iron given daily in the reference arm (12.5 mg) is according to WHO guidelines for the age group children in non-malarious areas or malaria-endemic areas where it should be implemented in conjunction with measures to prevent, diagnose and treat malaria and co-infections. The iron dose in the IHAT arm (20 mg) is the bioequivalent dose, i.e. the same absolute amount of iron should be absorbed as in the ferrous sulphate arm, and because IHAT should be safer to the gut, the unabsorbed fraction should not cause detrimental effects, such as infectious diarrhoea. This dose (20 mg Fe) is still less than the new WHO recommendation for children in our 24-35 mo. age group (i.e. 30 mg, (40)). In any case, in all arms of the trial these adverse events will be closely monitored.

Additionally, we have put in place the following strategies to mitigate the risk of possible interactions between iron supplements and malaria or other co-infections:

(1) data from the Gambia over the last 5 years (medical records from the Kiang West region) shows that the peak malaria months are Oct and Nov and, therefore, we have timed the intervention period to avoid these months, (2) trained field workers will be visiting all children every day during the 12 weeks supplementation period in order to supervise the administration of the iron supplements or placebo and on these occasions they will check on the children's health status and actively look for signs of malaria and co-infections, if a child shows signs of these infections the study nurse will perform adequate tests and the child will be offered the appropriate treatment/referral to the next Health Centre. In case of a fever, a malaria rapid test will be performed and if positive the child will be treated according to national guidelines. A sick child will always be visited by a study nurse for further clinical investigations and if needed referred to the nearest Health Centre. These visits will carry on for 4 weeks after the end of the study

intervention and, during both the intervention and this follow-up period, morbidity data will be captured every other day. This is similar to what was done in the HIGH study and we do not anticipate any difficulties of implementation. Every week, the child will visit one of the study health facilities for a haemoglobin check-up and malaria RDT testing, and this will ensure that children that develop asymptomatic malaria are identified and treated according to national guidelines.

Participants will experience some transient pain during blood sampling, which will be minimised by recruiting well-trained nurses for the study.

Children will benefit from daily monitoring of their health and from immediate care in case of illness. Although the mechanism of combating iron deficiency is problematic, it is clear that iron is a key micronutrient for the development of the immune system and cognitive function in these young children.

11.2 Informed consent

All field workers taking part in the recruitment of participants will be trained on translating the contents of the information sheet and the details of it will be explained to illiterate mothers in a language they understand in the presence of an independent literate witness. The literate mothers/parental guardian will be allowed to read the information sheet in their own time. They will be given enough time to ask questions and decide if they want their child to participate. Informed consent will be recorded by a signature or thumbprint on the consent form. The consent form together with the trial protocol and information sheet is attached to this submission.

11.3 Participant confidentiality

Each participant will be allocated an individual identification (ID) number and these will be used to label all samples collected for the study and on the CRF during the course of the study. All data will be linked-anonymised and the linkage to the ID will not be possible without a lookup table, which will be held only by the data manager and designated data staff during the course of the study. Once data collection is complete, analysis will be performed on an anonymised copy of the data. At all stages, staff/collaborators responsible for sample analysis will be blinded as to

the subject’s identification. Together, these processes will ensure complete confidentiality of the data gathered and impartiality of data analysis.

11.4 Future use of stored specimen

Some of the samples will be transferred to Kings College London (serum) and the Wellcome Trust Sanger Institute (faecal DNA) for analysis of outcomes not available in the Gambia.

Aliquots of blood and stool samples will be kept frozen at -70°C for future analysis. This may include DNA analysis and export of samples. This was a request from the funders. We will obtain informed consent from the mothers/guardians for this to be the case within the study informed consent. Any future use would require PI, MRCG SCC and EC approval.

12 Financing and insurance

The research related costs of the proposed trial will be paid by a grant from the Bill & Melinda Gates Foundation.

The London School of Hygiene and Tropical Medicine (LSHTM) will sponsor this research and as such research participants will be protected in accordance with the LSHTM Clinical Trial/Non Negligent Harm Insurance and Medical Malpractice Insurance.

13 Publication policy

Our planned dissemination avenues include: at least three publications in high impact peer-reviewed open-access scientific journals with a wide readership (e.g. Lancet, JAMA, NEJM), presentation at international conferences (e.g. the Micronutrient Forum) and dissemination of the trial findings to organizations such as the WHO, UNICEF, UN World Food Programme and to the National Nutrition Agency and the Ministry of Health in The Gambia.

14 References

1. WHO. The global burden of disease: 2004 update. Geneva: WHO, 2008.

2. Sazawal S, Black RE, Ramsan M, Chwaya HM, Stoltzfus RJ, Dutta A, et al. Effects of routine prophylactic supplementation with iron and folic acid on admission to hospital and mortality in preschool children in a high malaria transmission setting: community-based, randomised, placebo-controlled trial. Lancet. 2006;367(9505):133-43.
3. Soofi S, Cousens S, Iqbal SP, Akhund T, Khan J, Ahmed I, et al. Effect of provision of daily zinc and iron with several micronutrients on growth and morbidity among young children in Pakistan: a cluster-randomised trial. Lancet. 2013;382(9886):29-40.
4. Prentice AM, Verhoef H, Cerami C. Iron fortification and malaria risk in children. Jama. 2013;310(9):914-5.
5. Prentice AM. Iron metabolism, malaria, and other infections: what is all the fuss about? J Nutr. 2008;138(12):2537-41.
6. Mayo-Wilson E, Imdad A, Junior J, Dean S, Bhutta ZA. Preventive zinc supplementation for children, and the effect of additional iron: a systematic review and meta-analysis. BMJ Open. 2014;4(6):e004647.
7. Zlotkin S, Newton S, Aimone AM, et al. Effect of iron fortification on malaria incidence in infants and young children in ghana: A randomized trial. Jama. 2013;310(9):938-47.
8. Jaeggi T, Kortman GA, Moretti D, Chassard C, Holding P, Dostal A, et al. Iron fortification adversely affects the gut microbiome, increases pathogen abundance and induces intestinal inflammation in Kenyan infants. Gut. 2014.
9. Zimmermann MB, Chassard C, Rohner F, N'Goran E K, Nindjin C, Dostal A, et al. The effects of iron fortification on the gut microbiota in African children: a randomized controlled trial in Cote d'Ivoire. Am J Clin Nutr. 2010;92(6):1406-15.
10. Naylor C, Lu M, Haque R, Mondal D, Buonomo E, Nayak U, et al. Environmental Enteropathy, Oral Vaccine Failure and Growth Faltering in Infants in Bangladesh. EBioMedicine. 2015;2(11):1759-66.
11. Lin A, Arnold BF, Afreen S, Goto R, Huda TM, Haque R, et al. Household environmental conditions are associated with enteropathy and impaired growth in rural Bangladesh. Am J Trop Med Hyg. 2013;89(1):130-7.
12. Werner T, Wagner SJ, Martinez I, Walter J, Chang JS, Clavel T, et al. Depletion of luminal iron alters the gut microbiota and prevents Crohn's disease-like ileitis. Gut. 2011;60(3):325-33.
13. Dogan B, Suzuki H, Herlekar D, Sartor RB, Campbell BJ, Roberts CL, et al. Inflammation-associated Adherent-invasive Escherichia coli Are Enriched in Pathways for Use of Propanediol and Iron and M-cell Translocation. Inflamm Bowel Dis. 2014;20(11):1919-32.
14. Prentice AM, Doherty CP, Abrams SA, Cox SE, Atkinson SH, Verhoef H, et al. Hepcidin is the major predictor of erythrocyte iron incorporation in anemic African children. Blood. 2012;119(8):1922-8.
15. Tolkien Z, Stecher L, Mander AP, Pereira DI, Powell JJ. Ferrous sulfate supplementation causes significant gastrointestinal side-effects in adults: a systematic review and meta-analysis. PLoS One. 2015;10(2):e0117383.

16. Radulescu S, Brookes MJ, Salgueiro P, Ridgway RA, McGhee E, Anderson K, et al. Luminal iron levels govern intestinal tumorigenesis after apc loss in vivo. Cell Rep. 2012;2(2):270-82.
17. Seril DN, Liao J, Ho KL, Warsi A, Yang CS, Yang GY. Dietary iron supplementation enhances DSS-induced colitis and associated colorectal carcinoma development in mice. Dig Dis Sci. 2002;47(6):1266-78.
18. Seril DN, Liao J, Yang CS, Yang GY. Systemic iron supplementation replenishes iron stores without enhancing colon carcinogenesis in murine models of ulcerative colitis: comparison with iron-enriched diet. Dig Dis Sci. 2005;50(4):696-707.
19. Loh YH, Jakszyn P, Luben RN, Mulligan AA, Mitrou PN, Khaw KT. N-nitroso compounds and cancer incidence: the European Prospective Investigation into Cancer and Nutrition (EPIC)-Norfolk Study. American Journal of Clinical Nutrition. 2011;93(5):1053-61.
20. Lunn JC, Kuhnle G, Mai V, Frankenfeld C, Shuker DE, Glen RC, et al. The effect of haem in red and processed meat on the endogenous formation of N-nitroso compounds in the upper gastrointestinal tract. Carcinogenesis. 2007;28(3):685-90.
21. Santiago P. Ferrous versus ferric oral iron formulations for the treatment of iron deficiency: a clinical overview. Scientific World Journal. 2012;2012:846824.
22. Ruiz-Arguelles GJ, Diaz-Hernandez A, Manzano C, Ruiz-Delgado GJ. Ineffectiveness of oral iron hydroxide polymaltose in iron-deficiency anemia. Hematology. 2007;12(3):255-6.
23. Powell JJ, Bruggraber SFA, Faria N, Poots LK, Hondow N, Pennycook TJ, et al. A nano-disperse ferritin-core mimetic that efficiently corrects anemia without luminal iron redox activity. Nanomedicine: Nanotechnology, Biology and Medicine. 2014;10(7):1529-38.
24. Pan YH, Sader K, Powell JJ, Bleloch A, Gass M, Trinick J, et al. 3D morphology of the human hepatic ferritin mineral core: new evidence for a subunit structure revealed by single particle analysis of HAADF-STEM images. J Struct Biol. 2009;166(1):22-31.
25. Michel FM, Ehm L, Antao SM, Lee PL, Chupas PJ, Liu G, et al. The structure of ferrihydrite, a nanocrystalline material. Science. 2007;316(5832):1726-9.
26. Theil EC, Chen H, Miranda C, Janser H, Elsenhans B, Nunez MT, et al. Absorption of iron from ferritin is independent of heme iron and ferrous salts in women and rat intestinal segments. J Nutr. 2012;142(3):478-83.
27. Bejjani S, Pullakhandam R, Punjal R, Nair KM. Gastric digestion of pea ferritin and modulation of its iron bioavailability by ascorbic and phytic acids in caco-2 cells. World J Gastroenterol. 2007;13(14):2083-8.
28. Lonnerdal B, Bryant A, Liu X, Theil EC. Iron absorption from soybean ferritin in nonanemic women. Am J Clin Nutr. 2006;83(1):103-7.
29. Pereira DIA, Bruggraber SFA, Faria N, Poots LK, Tagmount MA, Aslam MF, et al. Nanoparticulate iron(III) oxo-hydroxide delivers safe iron that is well absorbed and utilised in humans. Nanomedicine: Nanotechnology, Biology and Medicine. 2014;10(8):1877-86.
30. Moretti D, Goede JS, Zeder C, Jiskra M, Chatzinakou V, Tjalsma H, et al. Oral iron supplements increase hepcidin and decrease iron absorption from daily or twice-daily doses in iron-depleted young women. Blood. 2015;126(17):1981-9.

31. Hutchinson C, Al-Ashgar W, Liu DY, Hider RC, Powell JJ, Geissler CA. Oral ferrous sulphate leads to a marked increase in pro-oxidant nontransferrin-bound iron. Eur J Clin Invest. 2004;34(11):782-4.
32. Barton Pai A, Pai MP, Depczynski J, McQuade CR, Mercier RC. Non-transferrin-bound iron is associated with enhanced Staphylococcus aureus growth in hemodialysis patients receiving intravenous iron sucrose. Am J Nephrol. 2006;26(3):304-9.
33. Cross JH, Bradbury RS, Fulford AJ, Jallow AT, Wegmuller R, Prentice AM, et al. Oral iron acutely elevates bacterial growth in human serum. Sci Rep. 2015;5:16670.
34. Latunde-Dada GO, Pereira DI, Tempest B, Ilyas H, Flynn AC, Aslam MF, et al. A Nanoparticulate Ferritin-Core Mimetic Is Well Taken Up by HuTu 80 Duodenal Cells and Its Absorption in Mice Is Regulated by Body Iron. The Journal of Nutrition. 2014;144(12):1896-902.
35. Aslam MF, Frazer DM, Faria N, Bruggraber SF, Wilkins SJ, Mirciov C, et al. Ferroportin mediates the intestinal absorption of iron from a nanoparticulate ferritin core mimetic in mice. Faseb J. 2014;28(8):3671-8.
36. Pereira DI, Mergler BI, Faria N, Bruggraber SF, Aslam MF, Poots LK, et al. Caco-2 Cell Acquisition of Dietary Iron(III) Invokes a Nanoparticulate Endocytic Pathway. PLoS One. 2013;8(11):e81250.
37. Pereira DIA, Aslam MF, Frazer DM, Schmidt A, Walton GE, McCartney AL, et al. Dietary iron depletion at weaning imprints low microbiome diversity and this is not recovered with oral nano Fe(III). MicrobiologyOpen. 2015;4(1):12-27.
38. Thurnham DI, McCabe LD, Haldar S, Wieringa FT, Northrop-Clewes CA, McCabe GP. Adjusting plasma ferritin concentrations to remove the effects of subclinical inflammation in the assessment of iron deficiency: a meta-analysis. Am J Clin Nutr. 2010;92(3):546-55.
39. Engle-Stone R, Nankap M, Ndjebayi AO, Erhardt JG, Brown KH. Plasma ferritin and soluble transferrin receptor concentrations and body iron stores identify similar risk factors for iron deficiency but result in different estimates of the national prevalence of iron deficiency and iron-deficiency anemia among women and children in Cameroon. J Nutr. 2013;143(3):369-77.
40. WHO. Guideline: Daily iron supplementation in infants and children. Geneva, Switzerland: World Health Organization, 2016.
41. Liu J, Gratz J, Amour C, Kibiki G, Becker S, Janaki L, et al. A laboratory-developed TaqMan Array Card for simultaneous detection of 19 enteropathogens. Journal of clinical microbiology. 2013;51(2):472-80.
42. Christian LM, Iams JD, Porter K, Glaser R. Inflammatory responses to trivalent influenza virus vaccine among pregnant women. Vaccine. 2011;29(48):8982-7.
43. Paine NJ, Ring C, Bosch JA, Drayson MT, Veldhuijzen van Zanten JJ. The time course of the inflammatory response to the Salmonella typhi vaccination. Brain Behav Immun. 2013;30:73-9.
44. Ma Y, Podinovskaia M, Evans PJ, Emma G, Schaible UE, Porter J, et al. A novel method for non-transferrin-bound iron quantification by chelatable fluorescent beads based on flow cytometry. Biochem J. 2014;463(3):351-62.

45. Singh S, Hider RC, Porter JB. A direct method for quantification of non-transferrin-bound iron. Anal Biochem. 1990;186(2):320-3.
46. Sebastiani G, Pantopoulos K. NTBI unveiled by chelatable fluorescent beads. Biochem J. 2014;463(3):e7-9.
47. Suchdev PS, Davis SM, Bartoces M, Ruth LJ, Worrell CM, Kanyi H, et al. Soil-transmitted helminth infection and nutritional status among urban slum children in Kenya. Am J Trop Med Hyg. 2014;90(2):299-305.
48. Ahmed A, Al-Mekhlafi HM, Al-Adhroey AH, Ithoi I, Abdulsalam AM, Surin J. The nutritional impacts of soil-transmitted helminths infections among Orang Asli schoolchildren in rural Malaysia. Parasit Vectors. 2012;5:119.
49. Committee WE. Prevention and control of schistosomiasis and soil-transmitted helminthiasis. World Health Organization technical report series. 2002;912:i-vi, 1-57.
50. Eren AM, Morrison HG, Lescault PJ, Reveillaud J, Vineis JH, Sogin ML. Minimum entropy decomposition: unsupervised oligotyping for sensitive partitioning of high-throughput marker gene sequences. Isme J. 2015;9(4):968-79.
51. Stallard N. Optimal sample sizes for phase II clinical trials and pilot studies. Statistics in medicine. 2012;31(11-12):1031-42.
52. De-Regil LM, Suchdev PS, Vist GE, Walleser S, Pena-Rosas JP. Home fortification of foods with multiple micronutrient powders for health and nutrition in children under two years of age. Cochrane Database Syst Rev. 2011(9):CD008959.
53. Dostal A, Baumgartner J, Riesen N, Chassard C, Smuts CM, Zimmermann MB, et al. Effects of iron supplementation on dominant bacterial groups in the gut, faecal SCFA and gut inflammation: a randomised, placebo-controlled intervention trial in South African children. The British journal of nutrition. 2014;112(4):547-56.

Supplements, appendices and other documents

Appendix 1: Project Timeline

Task Name	Start	Finish
**Project**	**Fri 01/04/16**	**Fri 01/03/19**
**Regulatory and Ethical approvals**	**Fri 01/04/16**	**Wed 30/09/16**
Protocol written	Fri 01/04/16	Fri 29/04/16
Sponsor review	Mon 02/05/16	Mon 20/06/16
CTA application	Tue 21/06/16	Wed 09/11/16
Ethics application	Tue 21/06/16	Fri 09/09/16
GMP clinical batch IHAT ordered	Wed 01/06/16	Fri 10/03/17
Interim report submitted (M 1)	Mon 01/08/16	Wed 30/09/16
Milestone 1	Wed 30/09/16	Wed 30/09/16
**Trial Setup and Recruitment**	**Tue 02/08/16**	**Thur 12/07/18**
GMP encapsulation of IHAT and comparators ordered	Thu 01/09/16	Wed 27/12/17
Protocol amendments	Mon 27/02/17	Mon 21/05/18
Trial protocol submitted	Mon 01/05/17	Fri 28/07/17
Trial supplies ordered	Mon 01/08/16	Fri 31/03/17
Trial documentation prepared	Mon 01/08/16	Fri 31/03/17
Trial database built	Mon 01/08/17	Fri 29/12/17
Setup of trial committees	Thu 01/09/16	Fri 29/09/17
Staff training at MRCG Basse	Mon 01/08/16	Thur 12/10/17
Upper river communities sensitization	Mon 12/12/16	Fri 08/06/18
Interim report submitted	Fri 05/05/17	Fri 05/05/17

Protocol #: 1489

Task Name	Start	Finish
Participant recruitment cohort 1	Mon 30/10/17	Thur 04/01/18
Participant recruitment cohort 2	Mon 05/03/18	Thur 05/04/18
Participant recruitment cohort 3	Mon 18/06/18	Thur 02/08/18
Local analysis of screening samples	Mon 30/10/17	Fri 30/11/18
Interim report submitted (M 2)	Fri 31/08/18	Fri 31/08/18
Milestone 2	Fri 31/08/18	Fri 31/08/18
**Trial Field Data Collection**	**Mon 08/01/18**	**Thur 29/11/18**
Cohort 1 intervention	Mon 08/01/18	Thur 05/04/18
Cohort 2 intervention	Mon 23/04/18	Thur 19/07/18
Cohort 3 intervention	Mon 06/08/18	Thur 01/11/18
AEs follow-up	Mon 08/01/18	Thur 29/11/18
Local analysis of trial samples	Mon 08/01/18	Fri 30/11/18
Interim report submitted (M 3)	Fri 14/12/18	Fri 14/12/18
Milestone 3	Fri 14/12/18	Fri 14/12/18
**Analysis and Reporting**	**Mon 17/12/18**	**Fri 29/03/19**
External analysis of trial samples (microbiome and NTBI)	Mon 17/09/18	Fri 21/12/18
Data queries resolved	Mon 30/10/17	Fri 30/11/18
Data analysis	Mon 17/12/18	Thur 28/02/19
Data monitoring committee report	Fri 01/03/19	Fri 29/03/19
Final project report	Fri 01/03/19	Fri 29/03/19
Publication of findings submitted	Mon 04/03/19	Fri 29/03/19
**Milestone 4 and Project End**	**Sun 31/03/19**	**Sun 31/03/19**

Appendix 2: Target Product Profile (TPP)

Proposed Targeted Product Profile (TPP) for IHAT for the treatment of IDA in children (i.e. following the proposed trial and once market authorisation is secured). For the current proposed trial we are working towards meeting the minimum acceptable results rather than the target, which would be for the next stage trial.

Product Properties	Minimum Acceptable Result	Target Result
**Dosing regimen**	Oral, at least four days/ week	Oral, once a day
**Formulation**	Single-API dose containing 20 mg Fe (bioequivalent to 12.5 mg Fe as $FeSO_4$) for children.	Multi-micronutrient powder formulations with taste masking for paediatrics, containing 20 mg Fe.
**Cost of treatment**	≤\$0.05 per child dose	≤\$0.01 per child dose
**Shelf-life of formulated product**	2 yr at ≤ 25°C	2 yr at ≤ 40°C
**Nutrient-nutrient interactions**	No unmanageable risk in terms of solid state or pharmacokinetic interactions	No risks in terms of solid state or pharmacokinetic interactions
**Clinical efficacy (reduction in anaemia after 3 months)**	30% of patients with Hb increase ≥ 1 g/dL	30% of patients with anaemia resolved (HB>11 g/dL)
**Clinical efficacy (reduction in iron deficiency after 6 months)**	30% of patients with ID resolved (sTfR/log10 ferritin (sTfR-F) index ≤2)	50% of patients with ID resolved (sTfR-F index ≤2)
**Safety**	Few drug-related SAEs, including diarrhoea	No drug-related SAEs<br>Minimal drug-related AEs, including diarrhoea
**Intestinal infection**	Drug-related bacterial infection in ≤ 15% of subjects	No drug-related bacterial infection
**Gut microbiome changes**	No significant increase in Enterobacteria or Enterobacteria/(Bifidobacteria +Lactobacillus	Less Enterobacteria relative to Bifidobacteria and Lactobacillus.

Appendix 3:

WORLD MEDICAL ASSOCIATION DECLARATION OF HELSINKI
Ethical Principles for Medical Research Involving Human Subjects

Adopted by the 18th WMA General Assembly, Helsinki, Finland, June 1964
and amended by the:
29th WMA General Assembly, Tokyo, Japan, October 1975
35th WMA General Assembly, Venice, Italy, October 1983
41st WMA General Assembly, Hong Kong, September 1989
48th WMA General Assembly, Somerset West, Republic of South Africa, October 1996
52nd WMA General Assembly, Edinburgh, Scotland, October 2000
53rd WMA General Assembly, Washington DC, USA, October 2002 (Note of Clarification added)
55th WMA General Assembly, Tokyo, Japan, October 2004 (Note of Clarification added)
59th WMA General Assembly, Seoul, Republic of Korea, October 2008
64th WMA General Assembly, Fortaleza, Brazil, October 2013

Preamble

1. The World Medical Association (WMA) has developed the Declaration of Helsinki as a statement of ethical principles for medical research involving human subjects, including research on identifiable human material and data.

The Declaration is intended to be read as a whole and each of its constituent paragraphs should be applied with consideration of all other relevant paragraphs.

2. Consistent with the mandate of the WMA, the Declaration is addressed primarily to physicians. The WMA encourages others who are involved in medical research involving human subjects to adopt these principles.

General Principles

3. The Declaration of Geneva of the WMA binds the physician with the words, "The health of my patient will be my first consideration," and the International Code of Medical Ethics declares that, "A physician shall act in the patient's best interest when providing medical care."

4. It is the duty of the physician to promote and safeguard the health, well-being and rights of patients, including those who are involved in medical research. The physician's knowledge and conscience are dedicated to the fulfilment of this duty.

5. Medical progress is based on research that ultimately must include studies involving human subjects.

6. The primary purpose of medical research involving human subjects is to understand the causes, development and effects of diseases and improve preventive, diagnostic and therapeutic interventions (methods, procedures and treatments). Even the best proven interventions must be evaluated continually through research for their safety, effectiveness, efficiency, accessibility and quality.

7. Medical research is subject to ethical standards that promote and ensure respect for all human subjects and protect their health and rights.

8. While the primary purpose of medical research is to generate new knowledge, this goal can never take precedence over the rights and interests of individual research subjects.

9. It is the duty of physicians who are involved in medical research to protect the life, health, dignity, integrity, right to self-determination, privacy, and confidentiality of personal information of research subjects. The responsibility for the protection of research subjects must always rest with the physician or other health care professionals and never with the research subjects, even though they have given consent.

10. Physicians must consider the ethical, legal and regulatory norms and standards for research involving human subjects in their own countries as well as applicable international norms and standards. No national or international ethical, legal or regulatory requirement should reduce or eliminate any of the protections for research subjects set forth in this Declaration.

11. Medical research should be conducted in a manner that minimises possible harm to the environment.

12. Medical research involving human subjects must be conducted only by individuals with the appropriate ethics and scientific education, training and qualifications. Research on patients or healthy volunteers requires the supervision of a competent and appropriately qualified physician or other health care professional.

13. Groups that are underrepresented in medical research should be provided appropriate access to participation in research.

14. Physicians who combine medical research with medical care should involve their patients in research only to the extent that this is justified by its potential preventive, diagnostic or therapeutic value and if the physician has good reason to believe that participation in the research study will not adversely affect the health of the patients who serve as research subjects.

15. Appropriate compensation and treatment for subjects who are harmed as a result of participating in research must be ensured.

Risks, Burdens and Benefits

16. In medical practice and in medical research, most interventions involve risks and burdens.

Medical research involving human subjects may only be conducted if the importance of the objective outweighs the risks and burdens to the research subjects.

17. All medical research involving human subjects must be preceded by careful assessment of predictable risks and burdens to the individuals and groups involved in the research in comparison with foreseeable benefits to them and to other individuals or groups affected by the condition under investigation.

Measures to minimise the risks must be implemented. The risks must be continuously monitored, assessed and documented by the researcher.

18. Physicians may not be involved in a research study involving human subjects unless they are confident that the risks have been adequately assessed and can be satisfactorily managed.

When the risks are found to outweigh the potential benefits or when there is conclusive proof of definitive outcomes, physicians must assess whether to continue, modify or immediately stop the study.

Vulnerable Groups and Individuals

19. Some groups and individuals are particularly vulnerable and may have an increased likelihood of being wronged or of incurring additional harm.

All vulnerable groups and individuals should receive specifically considered protection.

20. Medical research with a vulnerable group is only justified if the research is responsive to the health needs or priorities of this group and the research cannot be carried out in a non-vulnerable group. In addition, this group should stand to benefit from the knowledge, practices or interventions that result from the research.

Scientific Requirements and Research Protocols

21. Medical research involving human subjects must conform to generally accepted scientific principles, be based on a thorough knowledge of the scientific literature, other relevant sources of information, and adequate laboratory and, as appropriate, animal experimentation. The welfare of animals used for research must be respected.

22. The design and performance of each research study involving human subjects must be clearly described and justified in a research protocol.

The protocol should contain a statement of the ethical considerations involved and should indicate how the principles in this Declaration have been addressed. The protocol should include information regarding funding, sponsors, institutional affiliations, potential conflicts of interest, incentives for subjects and information regarding provisions for treating and/or

compensating subjects who are harmed as a consequence of participation in the research study.

In clinical trials, the protocol must also describe appropriate arrangements for post-trial provisions.

Research Ethics Committees

23. The research protocol must be submitted for consideration, comment, guidance and approval to the concerned research ethics committee before the study begins. This committee must be transparent in its functioning, must be independent of the researcher, the sponsor and any other undue influence and must be duly qualified. It must take into consideration the laws and regulations of the country or countries in which the research is to be performed as well as applicable international norms and standards but these must not be allowed to reduce or eliminate any of the protections for research subjects set forth in this Declaration.

The committee must have the right to monitor ongoing studies. The researcher must provide monitoring information to the committee, especially information about any serious adverse events. No amendment to the protocol may be made without consideration and approval by the committee. After the end of the study, the researchers must submit a final report to the committee containing a summary of the study's findings and conclusions.

Privacy and Confidentiality

24. Every precaution must be taken to protect the privacy of research subjects and the confidentiality of their personal information.

Informed Consent

25. Participation by individuals capable of giving informed consent as subjects in medical research must be voluntary. Although it may be appropriate to consult family members or community leaders, no individual capable of giving informed consent may be enrolled in a research study unless he or she freely agrees.

26. In medical research involving human subjects capable of giving informed consent, each potential subject must be adequately informed of the aims, methods, sources of funding, any possible conflicts of interest, institutional affiliations of the researcher, the anticipated benefits and potential risks of the study and the discomfort it may entail, post-study provisions and any other relevant aspects of the study. The potential subject must be informed of the right to refuse to participate in the study or to withdraw consent to participate at any time without reprisal. Special attention should be given to the specific information needs of individual potential subjects as well as to the methods used to deliver the information.

After ensuring that the potential subject has understood the information, the physician or another appropriately qualified individual must then seek the potential subject's freely-given informed consent, preferably in writing. If the consent cannot be expressed in writing, the non-written consent must be formally documented and witnessed.

All medical research subjects should be given the option of being informed about the general outcome and results of the study.

27. When seeking informed consent for participation in a research study the physician must be particularly cautious if the potential subject is in a dependent relationship with the physician or may consent under duress. In such situations the informed consent must be sought by an appropriately qualified individual who is completely independent of this relationship.

28. For a potential research subject who is incapable of giving informed consent, the physician must seek informed consent from the legally authorised representative. These individuals must not be included in a research study that has no likelihood of benefit for them unless it is intended to promote the health of the group represented by the potential subject, the research cannot instead be performed with persons capable of providing informed consent, and the research entails only minimal risk and minimal burden.

29. When a potential research subject who is deemed incapable of giving informed consent is able to give assent to decisions about participation in research, the physician must seek that assent in addition to the consent of the legally authorised representative. The potential subject's dissent should be respected.

30. Research involving subjects who are physically or mentally incapable of giving consent, for example, unconscious patients, may be done only if the physical or mental condition that prevents giving informed consent is a necessary characteristic of the research group. In such circumstances the physician must seek informed consent from the legally authorised representative. If no such representative is available and if the research cannot be delayed, the study may proceed without informed consent provided that the specific reasons for involving subjects with a condition that renders them unable to give informed consent have been stated in the research protocol and the study has been approved by a research ethics committee. Consent to remain in the research must be obtained as soon as possible from the subject or a legally authorised representative.

31. The physician must fully inform the patient which aspects of their care are related to the research. The refusal of a patient to participate in a study or the patient's decision to withdraw from the study must never adversely affect the patient-physician relationship.

32. For medical research using identifiable human material or data, such as research on material or data contained in biobanks or similar repositories, physicians must seek informed consent for its collection, storage and/or reuse. There may be exceptional situations where consent would be impossible or impracticable to obtain for such research. In such situations the research may be done only after consideration and approval of a research ethics committee.

Use of Placebo

33. The benefits, risks, burdens and effectiveness of a new intervention must be tested against those of the best proven intervention(s), except in the following circumstances:

Where no proven intervention exists, the use of placebo, or no intervention, is acceptable; or

Where for compelling and scientifically sound methodological reasons the use of any intervention less effective than the best proven one, the use of placebo, or no intervention is necessary to determine the efficacy or safety of an intervention.

and the patients who receive any intervention less effective than the best proven one, placebo, or no intervention will not be subject to additional risks of serious or irreversible harm as a result of not receiving the best proven intervention.

Extreme care must be taken to avoid abuse of this option.

Post-Trial Provisions

34. In advance of a clinical trial, sponsors, researchers and host country governments should make provisions for post-trial access for all participants who still need an intervention identified as beneficial in the trial. This information must also be disclosed to participants during the informed consent process.

Research Registration and Publication and Dissemination of Results

35. Every research study involving human subjects must be registered in a publicly accessible database before recruitment of the first subject. We will register this trial with the www.isrctn.com.

36. Researchers, authors, sponsors, editors and publishers all have ethical obligations with regard to the publication and dissemination of the results of research. Researchers have a duty to make publicly available the results of their research on human subjects and are accountable for the completeness and accuracy of their reports. All parties should adhere to accepted guidelines for ethical reporting. Negative and inconclusive as well as positive results must be published or otherwise made publicly available. Sources of funding, institutional affiliations and conflicts of interest must be declared in the publication. Reports of research not in accordance with the principles of this Declaration should not be accepted for publication.

Unproven Interventions in Clinical Practice

37. In the treatment of an individual patient, where proven interventions do not exist or other known interventions have been ineffective, the physician, after seeking expert advice, with informed consent from the patient or a legally authorised representative, may use an unproven intervention if in the physician's judgement it offers hope of saving life, re-establishing health or alleviating suffering. This intervention should subsequently be made the object of research, designed to evaluate its safety and efficacy. In all cases, new information must be recorded and, where appropriate, made publicly available.

CLINICAL TRIAL PROTOCOL

A novel nano-iron supplement (IHAT) to safely combat iron deficiency and anaemia (IDA) in young children: a double-blind randomised controlled trial

Protocol No:

**SCC No:**	**1489**
**Brief Title**	IHAT-Gut
**Other Number(s)**	
**Protocol Version – Date**	V4.0, 16 February 2018
**Sponsor**	London School of Hygiene & Tropical Medicine (LSHTM)
	MRC Unit The Gambia at LSHTM<br>PO Box 273 Banjul,<br>The Gambia, West Africa
**Chief Investigator**	Professor Andrew Prentice
**Local Principal Investigator**	Dr Mohammad Ilias Hossain
**Co PI:**	Dr Dora Pereira (University of Cambridge)

Table of contents

Page
Signature page 2
Protocol amendment(s) 3
Key roles 6
List of abbreviations 8
Protocol summary 11
1 Background information and rationale 15
1.1 Background information 15
1.2 Currently used oral iron and why IHAT is novel and dietary-like 17
1.3 Rationale 20
1.4 Potential risks and benefits 26
1.4.1 Risk mitigation 27
2 Study objectives 30
2.1 Study endpoints 32
3 Study design 34
3.1 Type of study and design 34
3.2 Randomisation and blinding procedures 35
3.2.1 Randomisation 35
3.2.2 Blinding 37
3.3 Sub-studies 38
3.4 Investigational products 38
3.4.1 Description of products 38
3.4.2 Formulation, packaging and labelling 38
3.4.3 Product storage and stability 39
3.4.4 Dosage, preparation and administration of investigational products 39
3.4.5 Concomitant medications/treatments 40
4 Selection and withdrawal of participants 40
4.1 Selection of participants 40
4.2 Eligibility of participants 42
4.2.1 Inclusion criteria 42
4.2.2 Exclusion criteria 42
4.3 Withdrawal of participants 43
5 Study procedures and evaluations 43
5.1 Study schedule 43
5.1.1 Study sensitisation 43
5.1.2 Screening and enrolment (baseline) 44
5.1.3 Follow-up (study visits) 45
5.1.4 Final study visit 47
5.1.5 Early termination visit 48
5.2 Study evaluations 50

5.2.1 Clinical evaluations ... 50
5.2.2 Laboratory evaluations ... 51
6 Safety considerations 53
6.1 Methods and timing for assessing, recording, and analysing safety parameters ... 54
6.1.1 Adverse events ... 54
6.1.2 Reactogenicity ... 54
6.1.3 Serious adverse events (SAEs) ... 55
6.1.4 Assessment of intensity of AEs ... 55
6.1.5 Assessment of causality ... 55
6.1.6 Serious Adverse Reaction (SAR) ... 57
6.2 Reporting procedures ... 57
6.3 Safety oversight ... 58
7 Discontinuation criteria 58
8 Statistical considerations 60
8.1 Sample size determination ... 60
9 Data handling and record keeping 67
9.1 Data management and processing ... 67
9.2 Source documents and access to source data ... 68
9.3 Protocol deviations ... 69
10 Quality control and quality assurance 69
10.1 Study monitoring ... 70
11 Ethical considerations 70
11.1 General considerations on human subject protection ... 70
11.1.1 Rationale for participant selection ... 71
11.1.2 Rationale for use of a placebo group ... 73
11.1.3 Evaluation of risks and benefits ... 74
11.2 Informed consent ... 75
11.3 Participant confidentiality ... 75
11.4 Future use of stored specimen ... 76
12 Financing and insurance 76
13 Publication policy 76
14 References 76
Supplements, appendices and other documents 81
Appendix 1: Project Timeline 82
Appendix 2: Target Product Profile (TPP) 84

Key roles

For questions regarding this protocol, contact Dr Dora Pereira, Department of Pathology, University of Cambridge, UK, diap2@cam.ac.uk, 0044-1223764864.

**Author(s):**	Dr Dora Pereira (University of Cambridge, diap2@cam.ac.uk) and Professor Andrew Prentice (MRCG, andrew.prentice@lshtm.ac.uk)
**Sponsor's representative:**	Dr Jonas Lexow (MRCG, jlexow@mrc.gm)
**Chief Investigator:**	Professor Andrew Prentice (MRCG, andrew.prentice@lshtm.ac.uk)
**Principal Investigator(s):**	Local PI: Dr Mohammad Ilias Hossain (Paediatrician, MRCG Basse, mihossain@mrc.gm)<br>Co-PI: Dr Dora Pereira (University of Cambridge, diap2@cam.ac.uk)
**Sub-Investigator(s):**	Ebrima Sise MRCG Keneba (support for iron assays)<br>Golam Sarwar MRCG Basse (study data manager/developer)<br>Lady Chilel Sanyang MRCG Basse (laboratory assays)<br>Amadou Jallow MRCG Keneba (laboratory assays)<br>Dr Davis Nwakanma MRC Unit The Gambia (Head of Laboratory Services)<br>Bolarinde Lawal MRC Unit The Gambia (Manager of Clinical Laboratories Fajara)<br>Tolulope Osunnuyi MRC Unit The Gambia, Keneba (Keneba laboratory manager, Analytical Project Manager)
**Trial Physicians:**	Dr Ogochukwu Ofordile, Study Research Clinician<br>Dr Mohammad Ilias Hossain, Local PI (Clinical Trial Coordinator)

**Sponsor Medical Expert:**	To be confirmed (we will consult Dr Muhammad Afolabi, Dr Ed Clark and other MRCG clinicians with paediatric expertise)
**Trial monitor(s):**	Sey Gibbi (MRCG Clinical Trials Support Office)
**Safety monitor(s):**	Local safety monitor (Dr Aderonke Odutola, Paediatrician)
**Chair of DSMB:**	Professor James Jay Berkley, Professor of Paediatric Infectious Diseases, KEMRI-Wellcome (Kilifi, Kenya)
**Chair of the TSC:**	Dr Margaret Pinder, MRCG Basse
**Statistician:**	Dr Nuredin Ibrahim Mohammed and Dr David Jeffries MRC Unit The Gambia at LSHTM (statistical support)<br>Dr James Wason MRC Biostatistics Unit, Cambridge (advisor for trial design, power calculation and primary outcome analysis, and member of TSC)
**Clinical Laboratory/ies:**	MRC Unit The Gambia at LSHTM, Keneba<br>MRC Unit The Gambia at LSHTM, Basse<br>MRC Unit The Gambia at LSHTM, Fajara
**Other institutions/ Collaborators:**	National Nutrition Agency [NaNA] (Modou Phall)<br>Wellcome Trust Sanger Institute, Cambridge, UK (Professor Julian Parkhill and Dr Josef Wagner)<br>King's College London, London, UK (Professor Robert Hider)
**Local Ethics Committee**	Gambia Government/MRC Joint Ethics Committee,<br>c/o MRC Unit The Gambia at LSHTM,<br>PO Box 273, Banjul, The Gambia, West Africa

List of abbreviations

ADME	Absorption, Distribution, Metabolism & Excretion
AE	Adverse Event
AGP	Alpha-1 acid Glycoprotein
API	Active Pharmaceutical Ingredient
BMGF	Bill and Melinda Gates Foundation
BSU	Biostatistics Unit
CI	Chief Investigator
CMO	Contract manufacturing Organisation
CRF	Case Report Form
CRP	C-reactive protein
DMT1	Divalent Metal Transporter 1
DSMB	Data & Safety Monitoring Board
EDTA	Ethylenediaminetetraacetic acid
FA	Field Assistant
FDA	Food and Drug Administration
FS	Field Supervisor
$FeSO_4$	Ferrous Sulphate
GCP	Good Clinical Practice
GMP	Good Manufacturing Practice
cGMP	Current Good Manufacturing Practice
GRAS	Generally Recognised as Safe
Hb	Haemoglobin
HNR	Human Nutrition Research (Unit)
ID	Iron Deficiency
IDA	Iron Deficiency Anaemia
ICH	International Conference on Harmonization

IEC	Independent Ethics Committee
IHAT	Iron Hydroxide Adipate Tartrate
IMP	Investigational Medicinal Product
LH	Lithium heparin
LSHTM	London School of Hygiene and Tropical Medicine
MHRA	Medicines and Healthcare products Regulatory Agency
MNP	Multimicronutrient Powder
MRC	Medical Research Council
MRCG	MRC Unit The Gambia at LSHTM
NaNa	National Nutrition Agency
NTA	Nitrilotriacetic acid
NTBI	Non-transferrin bound iron
PI	Principal Investigator
RBV	Relative Bioavailability Value
RCT	Randomised Controlled Trial
RDT	Rapid Diagnostic Test
RHT	Regional Health Team
SAE	Serious Adverse Event
SD	Standard Deviation
SFA	Senior Field Assistant
SO	Scientific Officer
SOP	Standard Operating Procedure
SSP	Study Specific Procedure
sTfR	Soluble transferrin receptor
STH	Soil-Transmitted Helminths
TSAT	Transferrin saturation
TSC	Trial Steering Committee
TPP	Target Product Profile

XRD	X-Ray Diffraction
WHO	World Health Organization

Protocol summary

**Title:**	A novel nano-iron supplement (IHAT) to safely combat iron deficiency and anaemia (IDA) in young children: a double-blind randomised controlled trial.
**Alias :**	IHAT-Gut
**Phase**:	Phase II
**Population:**	Young Children (6-35 mo.)
**Number of participants:**	705
**Number of Sites:**	1
**Location of Sites (including satellite sites):**	One site, Communities in Upper River Division (North Bank villages within a 20 km distance from Basse)
**Trial Duration:**	
**- Clinical Phase:**	9 months of study supplementation/intervention
**- Whole trial:**	11 months field data collection
**Duration for Participants:**	12 weeks intervention<br>+ 4 weeks AE/SAEs active follow-up post-intervention
**Description of Investigational Products:**	- 1 dose/day single IMP containing IHAT powder bioequivalent to 12.5 mg Fe (i.e. 20 mg Fe taking into account IHAT's relative bioavailability to $FeSO_4$)<br>- 1 dose/day single IMP containing $FeSO_4$ powder equivalent to 12.5 mg Fe<br>- 1 dose/day containing a placebo powder (no-iron, 'sugar' compound)
**Objectives:**	In this trial we will test the hypothesis that supplementation with IHAT eliminates iron deficiency and improves haemoglobin levels in young children without increasing infectious diarrhoea or promoting negative changes in the gut microbiome or inducing gut inflammation.<br>The **primary objective** for this trial is to show non-inferiority of IHAT in relation to ferrous sulphate at correcting

IDA, and in terms of diarrhoea to show superiority in relation to ferrous sulphate and non-inferiority in relation to placebo.

Secondary objectives are to show that IHAT is non-detrimental with respect to enteric pathogen burden, the gut microbiome, and intestinal inflammation.

Endpoints:

Primary endpoints:

There are 4 primary endpoints of the trial:

1. iron deficiency at 12 weeks
2. haemoglobin levels at 12 weeks
3. 'incidence density' of moderate-severe diarrhoea over the 12 weeks (i.e. the number of new moderate-severe diarrhoea episodes per child over the 12 weeks intervention)
4. 'period prevalence' of moderate-severe diarrhoea over the 12 weeks intervention period (i.e. the proportion of children with at least one episode of moderate-severe diarrhoea over the 12 weeks intervention)

To assess iron deficiency we will take into consideration the most up-to-date recommendation from WHO, who are currently conducting a consultation on this matter. The new WHO guidelines will include a recommendation for the best marker of iron deficiency in the context of inflammation and we expect this to be either using ferritin alone or the sTfR/logferritin index, where in both cases ferritin values will be inflammation-adjusted. More details on the possible methods to adjust ferritin values for inflammation are given in Section 2.1.

Iron deficiency and haemoglobin levels at 12 weeks will be used to define the prevalence of IDA which we will use to assess non-inferiority of IHAT relative to $FeSO_4$ in terms of efficacy at correcting IDA. We will determine the proportion of children in each arm which resolve iron deficiency **and** either achieve a normal Hb or an increase in Hb of at least 1 g/dL after the 12 weeks of the intervention.

	'Incidence density' and 'period prevalence' of moderate-severe diarrhoea will both be used to assess superiority of IHAT relative to $FeSO_4$ for the diarrhoea outcome and non-inferiority relative to placebo for the diarrhoea outcome (i.e. safety).<br><br>More details about the 4 study hypothesis and evaluations are provided in Sections 2.1 and 8.1.<br><br>Moderate-severe diarrhoea refers to those diarrhoea episodes where (i) the child passes more than 5 loose or liquid stools per day, (ii) there is blood in the stool (dysentery), or (iii) the child shows signs of clinical dehydration (assessed by the study nurse based on physical signs such as little or no urination, sunken eyes, and skin that lacks its normal elasticity).<br><br>**Secondary endpoints:**<br>Secondary endpoints will be faecal microbiome diversity and profile (particularly in terms of abundance of Enterobacteria), abundance of enteric pathogens, faecal calprotectin (marker of gut inflammation), hospitalisation and morbidity (data collected three times per week using the questionnaire developed in the HIGH study), malaria infection (data collected every week with RDT), treatment failures (i.e. the number of children who have to stop the study because their Hb falls below 7 g/dL), the proportion of days a child has diarrhoea over the intervention period ('longitudinal prevalence'), the proportion of days a child has moderate-severe diarrhoea over the intervention period ('longitudinal prevalence' of moderate-severe diarrhoea), 'incidence density' of bloody diarrhoea (i.e. the number of bloody diarrhoea episodes per child-month of observation), markers of systemic inflammation (serum CRP and AGP), and systemic markers of iron handling (hepcidin, transferrin saturation and circulating non-transferrin bound iron - NTBI).
**Description of Study Design:**	3-arm (IHAT, $FeSO_4$, placebo), parallel, randomised, double-blind, placebo-controlled clinical trial.

1 Background information and rationale

1.1 Background information

Iron deficiency (ID) and its associated anaemia (IDA) is the largest nutritional deficiency disorder in the world and affects a total of 1.2 billion people, the majority of whom are children and women from resource-poor countries. Iron deficiency is frequently exacerbated by concomitant parasitic and bacterial enteropathogenic infections and contributes to almost 0.8 million deaths a year as well as irreparably limiting the cognitive development of children and leading to poor outcomes in pregnancy (1). Currently available iron compounds are cheap and readily available but constitute an unphysiological approach to providing iron that leads to significant side effects and serious adverse events (2-5). Data and meta-analysis from trials involving nearly ten thousand young children in developing countries have consistently shown that conventional soluble oral iron supplementation used to treat IDA is associated with increased infection including bloody diarrhoea (3, 6-8) and detrimental changes to the gut microbiome and gut inflammation (8, 9), further increasing the burden from enteric infection and environmental enteropathy (i.e. persistent gut damage and inflammation that leads to malabsorption), which is a major cause of growth failure in children in resource-poor environments (10, 11). Consequently, ID and IDA remain without an effective cure and we have so far been unable to decrease the burden of this disease in children, even after considerable effort and investment in the past 20 years (Figure 1).

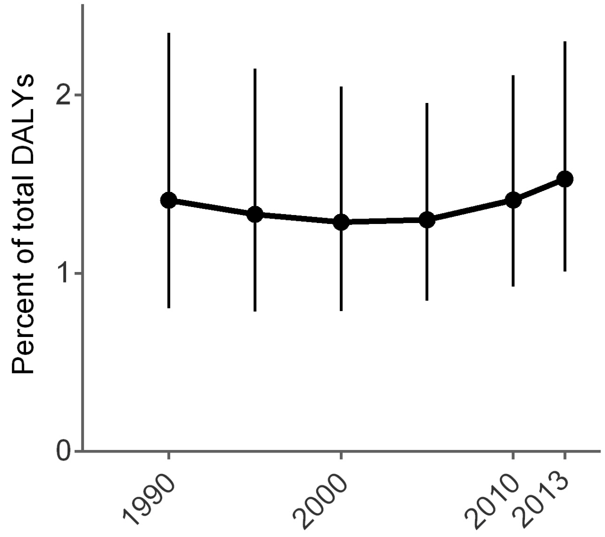

Figure 1. Global burden of iron deficiency anemia as a cause of disease in children under 5 y. Data is expressed as percent of disability adjusted life years (DALYs). Reference: Institute for Health Metrics and Evaluation (IHME). GBD Compare. Seattle, WA: IHME, University of Washington, 2015. Available from http://vizhub.healthdata.org/gbd-compare. (Accessed [13/01/2016])

The team at the MRC Human Nutrition Research Unit, have developed an innovative oral iron supplement (IHAT) for the safe treatment of iron deficiency and anaemia in resource-poor countries. Dr Pereira (who has been working at MRC HNR for the past 11 years) is a co-inventor of IHAT and is leading on its clinical development. As a next step in development we propose to conduct the first Phase II clinical trial with IHAT and obtain clinical data for its safety and efficacy in correcting IDA in young children. As mentioned above, many studies have shown that this is

the population group that suffers the most debilitating effects of current iron supplementation and, therefore, the group most at need of an alternative oral iron supplement that is safer, particularly in the gastrointestinal tract. Please refer to Section 11 (Ethical Considerations) for more details on our rationale for conducting this trial directly in children.

If IHAT is successful in the trial, these data will provide the evidence needed to encourage further investment so that IHAT can be implemented as a novel iron source for use in micronutrient intervention strategies aimed at children and women living in resource-poor countries and, hence, reduce the global burden of IDA.

The specific problem this study seeks to address relates to these serious side effects of oral iron supplements for treating anaemia in young children. Specifically, we will be addressing the fact that current iron supplements increase risk of infectious diarrhoea (3, 6-8) which promotes a pathogen-driven inflammatory response (8, 9, 12, 13) that impacts on hepcidin with reduction of iron absorption (14) and, therefore, offers limited benefit with added risk.

In the 21st century, we simply cannot accept the current view that increased risk of infectious diarrhoea in young children is normal collateral damage of oral iron supplementation in populations at high risk of enteric infection. This is a problem of current iron supplements that are all based on providing large bolus of non-physiological forms of ‘gut-reactive’ iron, that is soluble in the gut lumen, and to which humans have not previously been naturally exposed through diet. These harmful effects are caused by supplements that circumvent the natural chaperone and absorption/exclusion mechanisms of the gut.

We know that growth and virulence of bacterial enteropathogens is stimulated by these soluble iron supplements which are bioavailable to both the gut microbiome and the human host.

Conversely, our nano iron IHAT is effectively absorbed in humans without requiring solubilisation and, because it is not solubilised, it is not accessible as an iron source to bacterial enteropathogens or available to undergo significant redox cycling in the gut lumen (please refer to the summary of our pre-clinical and proof-of-concept data in Section 1.3 for details about IHAT’s absorption, safety and metabolism)**. This means that supplementation with IHAT should correct IDA without increasing the burden from infectious diarrhoea and should result in improved overall response to iron supplementation compared to conventional soluble iron. <u>In essence, our nano iron looks like dietary non-haem iron and should behave like dietary iron.</u>**

1.2 Currently used oral iron and why IHAT is novel and dietary-like

The current paradigm for iron delivery is that only soluble ionic iron can be efficiently absorbed through DMT1 present in the apical membrane of duodenal enterocytes. This paradigm has supported the development, and use, over the past 20 years of interventions that aim to deliver a large bolus of ionic iron to the enterocytes. Soluble ionic iron is not naturally present in the diet and, therefore, our bodies have only recently been exposed to significant amounts of this iron form through supplementation and fortification. Many different forms of iron have been tested, and are currently being used, but these all rely in the solubilisation of the compound in the stomach prior to the delivery of ionic iron, in the ferrous or ferric form, to the enterocyte cell.

Soluble ferrous iron (e.g. Fe(II) fumarate, Fe(II) bisglycinate) is redox reactive in the upper gastrointestinal tract and can redox cycle via Fenton chemistry promoting mucosal inflammation which leads to the acute side-effects normally reported with iron supplementation, such as heartburn, nausea and abdominal pain (15). Soluble ferric iron (e.g. Na Fe(III) EDTA, Fe(III) citrate, Fe(III) trimaltol, Fe(III) trisglycinate) is less redox reactive but soluble Fe(III) must still be reduced at the mucosal surface and may yield some 'sub-symptomatic' free radicals in the process, whilst the unabsorbed iron still delivers soluble iron to the colon where it is available to be utilized by pathogenic bacteria and to induce detrimental changes to the gut microbiome (8), and potentially increases risk of colorectal cancer (16-18). These effects may result in the distal and more chronic side effects of oral iron, namely diarrhoea or constipation. Furthermore, the high cost of ferric chelates limits their use in resource-poor countries. Haem iron supplements, such as heme iron polypeptide, are also expensive and there are marked concerns over haem safety in the colon in terms of catalizing the formation of N-nitroso compounds associated with cancer risk (19, 20). Other protein-bound forms of iron, such as lactoferrin and ferric mannitol ovoalbumin are several order of magnitude higher cost (~100 x), have limited shelf-life or are only useful for niche groups such as newborn babies.

Insoluble forms of iron, such as iron phosphates and elemental iron are not sufficiently bioavailable in humans to merit serious clinical use because they still require solubilisation in the stomach prior to absorption and the efficiency of this process is very low for these forms of iron. Ferric iron polymaltose is an insoluble iron compound made of aggregates of small iron oxo-hydroxide particles encased in a carbohydrate (polymaltose) coating. Even in the case these particles would be taken up whole by the enterocyte, they are too stable to be broken down and deliver enough iron for systemic use, hence their reported low bioavailability (21, 22).

Therefore, ferrous iron salts remain the oral iron compounds of choice because they are cheap and well absorbed and significant commercial effort and investment in the alternatives mentioned above has been unable to convince prescribers and governments that new oral iron preparations merit widespread use. Even in patients severely intolerant of standard oral iron, or in those where

risk outweighs benefit, the increasing second line preference is for intravenous (IV) iron, in Western countries, and for 'no iron', in resource-poor countries.

IHAT is distinct from all these forms of iron, in that it is not soluble nor does it require solubilisation in the stomach to be absorbed since it is taken up by enterocytes as whole nanoparticles (Section 1.3). This means that the unabsorbed fraction of the compound that transits to the lower gut, and inherently this is at least 60% of all ingested oral iron irrespective of the form, will remain nanoparticulate and, therefore not soluble, and as such will not be available to promote pathogen growth and tissue inflammation. An important aspect of IHAT structure is that this is sufficiently labile to break down effectively inside the enterocyte and deliver its iron because the native iron oxo-hydroxide structure (i.e. ferrihydrite) in IHAT has been purposely destabilised with the incorporation of dietary tartaric and adipic acids (23), much in the same way to what occurs in the ferritin iron core due to interactions with the aminoacid residues in the protein shell (24). IHAT is a tartrate-modified, nano-disperse Fe(III) oxo-hydroxide in the ferrihydrite mineral phase, formed in an adipate buffer, with similar functional properties and small primary particle size (~2 nm) as the iron form found in the ferritin core (i.e. ferrihydrite). Full physicochemical characterisation in relation to particle size, morphology, iron phase, XRD pattern, infrared spectra, and dissolution are presented in Powell *et al* (23). All components of IHAT (i.e. iron oxide, tartaric and adipic acids) are naturally present in foods and are approved food additives with a generally recognised as safe (GRAS) status by the US Food and Drug Administration (FDA). Besides composing the ferritin core, iron oxides are naturally present in foods and soils and are approved food additives (E172). Tartaric acid is naturally present in foods, most notably grapes, and is an approved antioxidant and acidifier food additive (E334). Adipic acid is also naturally found in foods and is an approved flavouring and gelling agent (E355).

Each daily dose of IHAT in this study will contain in addition to the 20 mg elemental Fe, 21 mg of tartaric acid and 4.7 mg of adipic acid. These amounts of the organic acids are less than 20% of the estimated ADI (acceptable daily intake) for a 5 kg child and less than 10% of the respective ADI for a 10 kg child (http://ec.europa.eu/food/fs/sfp/addit_flavor/flav15_en.pdf).

A molecular representation of IHAT is presented in Figure 2.

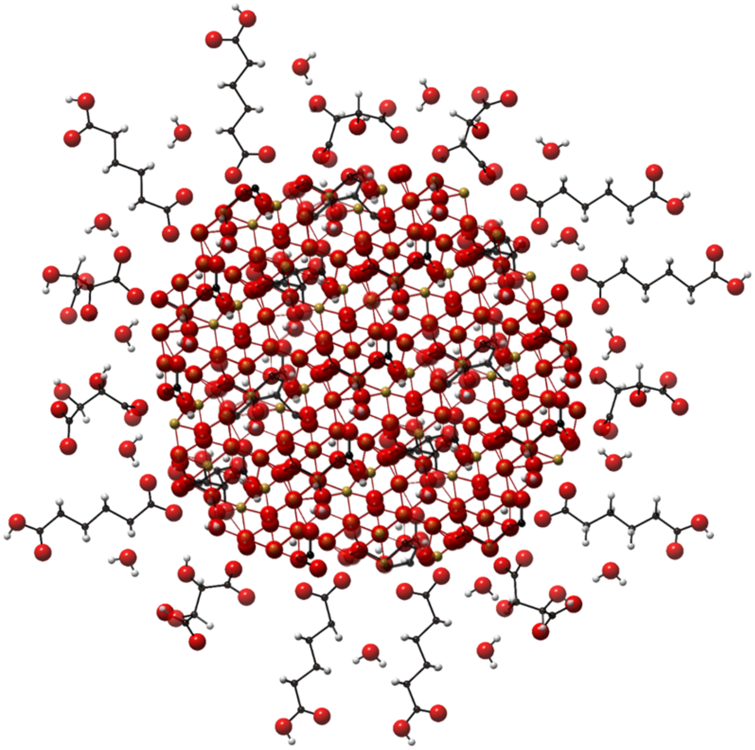

Figure 2. Molecular model of one IHAT 2 nm particle. The colours used to represent the atoms are: O (red), H (white), Fe (brown), C (black). The organic acids (tartaric and adipic) are represented adsorbed to the surface and incorporated into the structure core. We estimate that one IHAT particle would contain 150 Fe atoms (based on the ferrihydrite structure by Michel *et al* (25)). Chemical modelling by Dr Helen Chappell (unpublished).

Ferritin is composed of up to eight nanoparticles of iron oxo-hydroxide, also in the ferrihydrite mineral phase, stored in a protein shell that renders the iron particles both nano-dispersible and sufficiently labile to be utilized biologically. When ingested in either plant or animal based foods, it is well absorbed (26-28). The exact mechanism of absorption is not resolved- either ferritin is gradually broken down in the acidic, gastric lumen at a rate that matches later intestinal iron absorption or the particle resists total gastric degradation and is taken up whole by the enterocytes and gradually broken down intralysosomally to join the common iron pool. Either way, undigested nanoparticulate iron oxo-hydroxide should not be reactive in the same way that soluble iron is and, thus, should be poorly available to colonic bacteria or to participate in redox reactions at the epithelial surface (Section 1.3).

It was with this in mind, and based upon 20+ years of research into the chemistry of dietary minerals in the gastrointestinal lumen, that the co-PI and the team at MRC Human Nutrition Research have developed IHAT as a paradigm-shift from existing or pipeline products. IHAT is well absorbed and due to its nanoparticulate nature should carry very low side-effects as it should *not* undergo luminal redox cycling and it should *not* give up its unabsorbed iron to commensal and pathogenic bacteria (Section 1.3).

We are confident of IHAT's ability to outpace any other form of supplemental iron in use or under development. The anticipated advantages to the end user over competing and pipeline solutions will be (i) lack of acute 'sub-symptomatic', but nonetheless chemically undesirable, redox effects on the colonic mucosa and microbiome, (ii) reduced risk of intestinal infection and diarrhoea, and (iii) lack of symptomatic side-effects, all of which will ultimately result in better efficacy at correcting IDA.

1.3 Rationale

In this clinical trial we will test the hypothesis that supplementation with IHAT eliminates iron deficiency and improves haemoglobin levels in young children without increasing infectious diarrhoea or promoting negative changes in the gut microbiome or inducing gut inflammation. We propose to conduct the trial in the North Bank villages of the URR because these are some of the most deprived communities in the country where risk of infection, particularly enteropathogenic infection, is high. This will allow us to better distinguish IHAT and ferrous sulphate in terms of safety, which is where most of the clinical need for better iron supplementation lies.

Over the past ~10 years we have established sufficient pre-clinical and proof-of-concept data for IHAT to support moving to the Phase II trial proposed here. These data are summarised below and for the most part have been published. We have prepared an Investigator Brochure for IHAT comprising essentially the data summarised below, and have submitted this to the UK Medicines and Healthcare Products Regulatory Agency (MHRA), and asked for their scientific advice in relation to this protocol and the future development plan for IHAT, the MHRA's positive response letter is attached to this proposal. The Investigator Brochure for IHAT was also submitted to the Gambia Medicines Board, when we applied for clinical trial authorisation.

<u>IP protection</u>: IHAT is protected by an MRC-owned patent (WO2008096130) that has been granted in most major territories (e.g. Europe, US and China). The claims of this patent protect the composition-of-matter for the oral iron formulation to be taken into the clinic, as well as methods of manufacture and use for the preparation of a medicament for therapeutic delivery of

IHAT to a subject. The claims also protect the chemistry underlying the generation of oxo-hydroxide metal ion structures modified with organic moieties and thus offers broad protection of the chemical landscape.

Manufacture: A crucial advantage of IHAT, over competitors, is its facile synthesis leading to low manufacture cost compared to other complex iron formulations (namely iron chelates or protein-bound complexes). The cost of IHAT manufacture at scale is estimated as $0.3-$1.5 for a 28-day supply for one child (scale range 1-150 ton). This is only ~3x the cost of simple ferrous salts. Manufacture of IHAT involves a simple, but carefully-controlled, aqueous co-precipitation technique and product recovery is either by tray-drying or spray-drying. Based on feedback received from three contract-manufacture organizations (CMO) for the scale-up manufacture of IHAT, we are reassured that there will not be any significant hurdles in accommodating larger scale GMP manufacture.

Importantly, IHAT's manufacture is easily scalable and has a low cost due to the facile synthesis and inexpensive FDA GRAS (generally recognised as safe) raw materials. We anticipate that following this initial trial, we will have high-quality clinical data to support using IHAT instead of soluble iron (e.g. ferrous fumarate or sulphate) as the iron source in the micronutrient supplementation and home fortification (e.g. MNPs) interventions recommended by WHO to improve the iron status of populations. Following this initial pilot award, we would apply to BMGF for a full award that would be used to fund a trial where we would be investigating these different delivery systems with IHAT, different iron dosage regimens, and also assess efficacy in pregnant women. At that stage we would engage with the manufacturers of MNPs (for example DSM) to produce a formulation with IHAT instead of ferrous fumarate for testing.

Regulatory aspects: Scientific advice from the UK MHRA have assured us that the existing non-clinical and clinical data with IHAT would be sufficient to support the proposed trial (please refer to the MHRA letter dated 21st December 2016 attached to this proposal). Furthermore, advice from the MHRA in relation to a similar, but not identical, iron compound developed by our Group for a different indication was that toxicology studies would not be required and that the toxicology section of the CTA application could be comprised of data for the individual dietary components drawn from the literature. This compound has now completed Phase II testing in the USA.

For a Phase II/III adaptive trial in women (pregnant and non-pregnant) we also asked for advice from a regulatory consultancy and their report (commissioned to form part of a Wellcome Trust proposal pack) is also attached to this submission (Alacrita_151001 IHAT Regulatory Gap Analysis).

Commercial strategy: IHAT received the first prize at the 2014 Emerging Technologies Prize from the UK Royal Society of Chemistry (see http://www.rsc.org/chemistryworld/2014/12/solving-iron-solubility-problem-profile-mrc). As part of that prize we have been partnered with GSK who will continue to offer 'in-kind' support and mentorship as needed to help translate IHAT and bring it to those most at need of an alternative oral iron compound. MRC already has significant commercial interest from various commercial partners. The results of this trial will be very beneficial to a company and open up a new market for IHAT in the developing world.

Pre-clinical data: The main findings from the proof-of-concept and pre-clinical studies that support our hypothesis are presented below.

Phase 0 pharmacokinetics human studies: A study with 4 iron deficient women in the UK showed that (i) bioavailability (i.e. red blood cell incorporation) of iron from IHAT was ~ 75% that of iron from ferrous sulphate and (ii) IHAT was successful in reducing the non-physiological post-absorptive iron surge caused by ferrous sulphate that raises transferrin saturation (29). A recent single-dose study in 30 pre-menopausal women in West Kiang, The Gambia (SCC1422 – IHAT) has confirmed that a new recovery-method for IHAT's large-scale manufacture does not affect IHAT's bioavailability (Figure 3A); showed again that IHAT was successful in reducing the non-physiological post-absorptive iron surge caused by ferrous sulphate, that raises transferrin saturation (Figure 3B); and IHAT was successful in reducing *ex vivo* pathogen growth in the serum collected from the women following the single-dose (Figure 3C). We note that the relative bioavailability (RBV) of IHAT in the Gambia study (range 10-69%) was lower than that in the UK study (range 58-89%). However, the UK study had very few numbers, only 4 women, and, therefore, the full range of IHAT bioavailability was not observed.

For the purpose of calculating the bioequivalent dose for IHAT to use in the proposed trial we have considered the upper 75% centile of the median RBV (i.e. 60%), we took into account the data from both studies (n=25) since they were both conducted in the same population group (i.e. pre-menopausal IDA women). Our reasoning for using the upper 75% centile rather than the median value was that in IDA young children (in the absence of infection) we expect to have increased iron absorption due to their increased erythropoietic needs for growth and brain development relative to adult women and, therefore, we did not want to overestimate the dose needed.

Most importantly, IHAT was developed to simulate dietary iron absorption and slowly release the iron into the circulation so as not to cause an abnormal rise in transferrin saturation and this was indeed confirmed with the Gambia study (SCC 1422), in which the vast majority of women had TSAT always below 40% following the 60 mg Fe dose. This feature of IHAT has a number of advantages over ferrous sulphate which induces the un-physiological iron bolus release into the circulation: (i) the lower rise in serum Fe and TSAT after each daily dose of IHAT should not

cause a bolus rise in hepcidin which has been recently shown to inhibit absorption of the next-day dose with ferrous sulphate (30), (ii) less likelihood of formation of 'true' NTBI when TSAT is maintained below 40% (31), (iii) less 'free' iron highly available to sustain growth of systemic extracellular pathogens (32, 33).

A

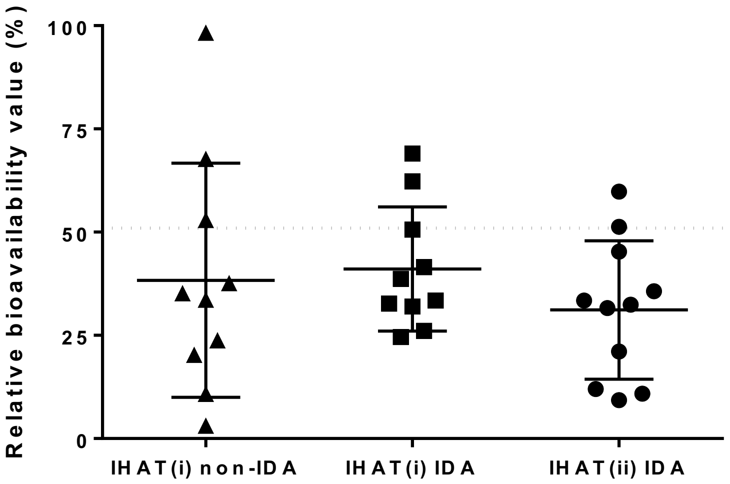

B

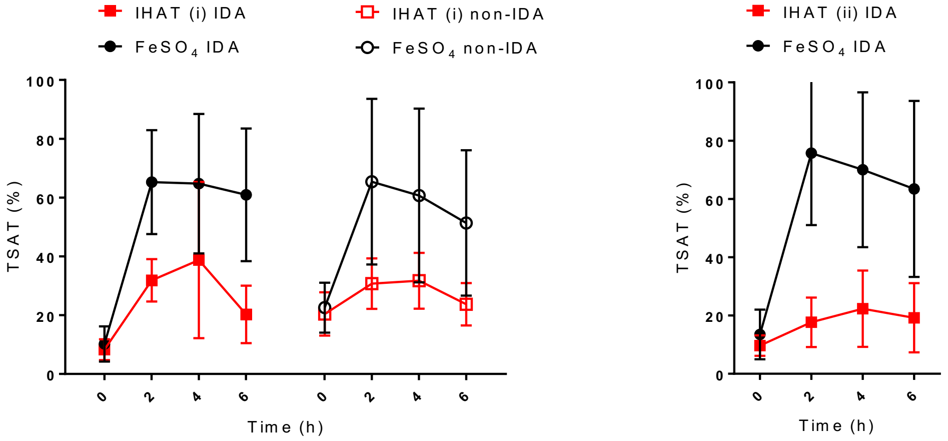

C

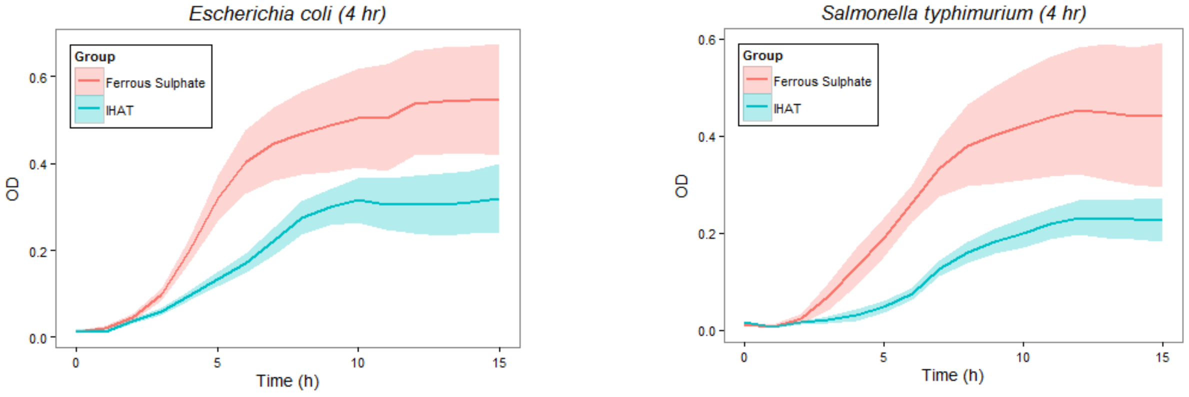

Figure 3. Unpublished data from the recent single-dose absorption study in pre-menopausal women in West Kiang, The Gambia (SCC 1422). A, relative bioavailability value (RBV) to ferrous sulphate (100%) determined from the red blood cell incorporation of the iron isotopes, box and whisker plots show median, minimum and maximum for n = 10 in each group, differences are not statistically significant; **B,** transferrin saturation following a single-dose of IHAT or ferrous sulphate (60 mg elemental Fe equivalent), data shown as mean (± SD, n=10/group), differences between IHAT and FeSO4 are statistically significant at the 2, 4 and 6 hours time-points, $p<0.01$; **C,** *ex vivo* bacterial growth of *E. coli* and *Salmonella typhimurium* in serum collected 4 hours following the single-dose of 60 mg Fe as IHAT or ferrous sulphate. There was strong evidence for the effect of supplementation group on bacterial growth ($p<0.0001$ using non-linear mixed effects modelling).

NOTE: Two identical IHAT powders were tested, these powders were recovered using a different procedure: IHAT (i) – tray-dried at 45 °C, IHAT (ii) – concentrated by solvent recovery and then tray-dried at 45 °C. The *ex vivo* assays were only conducted for IHAT(i).

Efficacy/haemoglobin repletion study in rats (14-days): Two independent studies have shown that IHAT is equivalent to ferrous sulphate at correcting haemoglobin levels in anaemia. No differences in bodyweight were observed between groups and there was no detectable iron deposition in the mucosa of the small intestine with IHAT (23, 29).

Pharmacokinetics mouse study (4 hours, radio-labelled): Absorption of IHAT was significantly increased in iron-deficient mice compared to iron-sufficient mice, showing that systemic absorption of IHAT is normally regulated by body iron levels (34).

ADME mouse studies (7 & 28 days): In two independent studies, IHAT was equivalent to ferrous sulphate at correcting haemoglobin levels. Systemic absorption of IHAT, i.e. basolateral export from the intestinal enterocyte into the blood circulation, was via ferroportin (as with ferrous sulphate) and was under regulation of normal iron homeostasis. Expression of duodenal DMT1, expression of liver hepcidin, liver iron, splenic iron and duodenal iron levels were all similar between the 2 groups (34, 35).

Mechanism of intestinal apical uptake - *in vitro* and *in vivo* studies:

In cellular and animal studies, IHAT was shown to be taken up apically as whole nanoparticles- i.e. a separate mechanism to the DMT1-driven uptake of conventional soluble iron supplements – and therefore did not require luminal or mucosal iron redox activity:

In mice, IHAT's absorption did not require redox activity (i.e. reduction of Fe(III) to Fe(II)) (23).

In Caco-2 differentiated cells (cells that resemble the duodenal enterocytes once differentiated), IHAT was taken up by the cells by endocytosis and this was followed by breakdown of IHAT inside endosomes/lysosomes within the cell to release the Fe (36).

In HuTu 80 cells (duodenal adenocarcinoma cells), IHAT was taken up by a mechanism independent of the divalent metal transporter 1 (DMT1) and of the duodenal cytochrome b (Dcytb) (34).

Correlation with human absorption: Caco2 Fe uptake studies

IHAT absorption in humans correlated with direct *in vitro* cellular uptake, but not with gastric solubility (29).

Importantly, we wish to note that even though IHAT is taken up apically by the enterocyte via a different mechanism to soluble iron, once inside the cell IHAT dissolves rapidly inside late-endosomes and lysosomes due to the combined action of low pH and high ligand-affinity for ferric iron (typically ~10mM citrate). Then, the iron released from IHAT in these cellular vesicles joins the common cellular labile iron pool (LIP) and does not bypass the normal physiological mechanisms that regulate iron absorption. Our data above indicate that IHAT nanoparticles do not translocate intact from the gut epithelium into the blood circulation.

Safety proof-of-concept *in vitro* assays: IHAT had no adverse effect on Caco-2 gut cell viability, even at doses 10-20 fold in excess of those possible in the gut lumen, whereas soluble ferrous iron was markedly toxic at 14-fold lower doses than IHAT (29). IHAT had no adverse effects on epithelial cell monolayer integrity (36).

IHAT had no adverse effects on Caco-2 (colon) and Hutu-80 (duodenum) gut cell viability or on cell growth and proliferation even at doses much higher than those expected in the gut lumen. Unpublished data indicate that ferric maltol is more toxic still.

Safety proof-of-concept *in vivo* studies: In an 'enteric infection' (i.e. microbiome dysbiosis) rodent model, IHAT was much less available to potential enteropathogens than ferrous sulphate and led to a more beneficial gut microbiome, with increasing Lactobacilli after 14 days supplementation. (29).

In a 'healthy colon' rodent model, IHAT did not negatively impact the gut microbiome and appeared to be less available as an iron source to bacteria after 28 days of supplementation (37).

1.4 Potential risks and benefits

Our vision is that our novel iron compound (IHAT) will significantly contribute to the goal of safely eliminating iron deficiency and iron deficiency anaemia in women and children living in developing countries. Our existing evidence strongly supports the contention that IHAT offers very important advantages over any other form of supplemental iron in use or under development.

The anticipated advantages to the end user over competing and pipeline solutions will be (i) lack of acute sub-symptomatic, but nonetheless chemically undesirable, redox effects on the colonic mucosa and microbiome, (ii) reduced risk of intestinal infection and diarrhoea, and (iii) lack of symptomatic side-effects leading to better compliance; all of which will ultimately result in better efficacy at correcting iron deficiency anaemia. Furthermore, as described above, IHAT's absorption into serum follows a slower 'dietary-like' kinetics and it should not result in the non-physiological post-absorptive iron surge caused by ferrous iron salts, therefore, supplementation with IHAT should carry less risk of systemic infection such as tuberculosis and other co-infections normally associated with malaria. IHAT has a low-cost of manufacture due to its facile synthesis and inexpensive raw materials and, therefore, there should be no constraints to its implementation as an iron supplement for populations in resource-poor countries.

If IHAT is successful in this first trial, i.e. safer in terms of diarrhoea and gut effects than ferrous sulphate and not worse at correcting IDA, these data will be used to support a full award application to the BMGF together with a translation/commercial partner so that IHAT can be implemented as a novel iron supplement for use in micronutrient intervention strategies in

developing countries and, hence, help to reduce the global burden of IDA. Ultimately, following the next trial (supposedly in pregnant women and children) we envisage that IHAT would be used instead of ferrous sulphate and ferrous fumarate in micronutrient intervention strategies across the developing world. This fast-track clinical development (i.e. ability to move directly to Phase II and not Phase I studies) is only possible due to (i) the fact that iron supplements already have a WHO recommended daily active dose for the several age groups and (ii) the dietary-like nature of IHAT.

IHAT is an analogue of natural food iron and is made-up of dietary constituents that are approved food ingredients by the US Food and Drug Administration (FDA), namely iron oxide, tartaric acid and adipic acid and have the FDA generally recognised as safe (GRAS) status. More details about the dietary-like molecular structure of IHAT are explained in Section 1.2.

We have prepared a risk mitigation strategy for the proposed trial, in discussion with our program officer at the BMGF, and this is shown below. We are also required by the Sponsor to develop a risk assessment and risk mitigation strategies prior to study starting and we are currently working with MRCG to develop such documentation.

1.4.1 Risk mitigation

- Stability of MRC Unit The Gambia at LSHTM; the Unit has just been approved for renewed funding from the UK Medical Research Council until 2021.

- Regulatory approval for the clinical trial in children; based on scientific advice from the UK MHRA (mentioned above) and our previous experience with an iron compound targeting a different clinical application (phosphate binder for end-stage CKD patients) we are confident that we will obtain authorisation to proceed directly to the trial proposed here. With the MRC Unit The Gambia at LSHTM we have already conducted an early-stage clinical trial with IHAT (single-dose study SCC 1422) and both the Ethics Committee and the Medicines Control Agency in the Gambia have reviewed the pre-clinical data with IHAT for that study.

  We are submitting a clinical trial authorisation (CTA) to both the Gambia and the UK MHRA (for protocol review), even though we are not legally required to apply to the MHRA, since we feel this is best practice and in line with the EU/Horizon 2020 guidelines for best-practice for clinical trials conducted in developing countries (http://ec.europa.eu/research/participants/portal/doc/call/h2020/h2020-msca-if-2015/1645175-h2020_-_guidance_ethics_self_assess_en.pdf).

We have contacted MHRA in relation to our intention to submit a CTA dossier for the trial proposed here and received confirmation that it would be appropriate to seek MHRA approval/review of the trial design and supporting pre-clinical data as part of a scientific advice meeting alongside local Gambia approvals. This MHRA meeting has now taken place and their scientific advice letter is attached to this proposal. The trial will be conducted in accordance with the principles of GCP as laid down in the Consolidated Guideline for Good Clinical Practice published by the International Conference on Harmonization in 1996 (ICH GCP Guideline) and the MRC Unit The Gambia at the London School of Hygiene & Tropical Medicine will Sponsor the research.

- Ethical approval; even though the planned trial is early-stage, given the long experience of the MRC Unit The Gambia at LSHTM in conducting oral iron interventions in children in The Gambia, the relatively low burden on study participants, and the use of children that are generally healthy apart from having IDA, we do not anticipate any ethical issues that could delay approval. The study protocol will be explained to the child mother/guardian orally in the presence of an independent and literate witness following MRCG SOPs in case they are illiterate or in writing and we will not start any study specific procedures before informed consent is obtained. The study investigates oral iron used in doses specifically recommended for this age group and even though IHAT is a novel compound, it is made up entirely of compounds naturally present in the diet and all our pre-clinical data supports that it should not behave any differently from dietary ferritin. In any case, even if IHAT is not absorbed and does not correct anaemia, it would not be worse in terms of safety that the forms of iron already in use in supplementation and home fortification strategies in this age group. We are assured that there should not be any ethical concerns over delaying 4-months the supply of iron treatment to the children enrolled in the placebo group since that it is not standard-of-care to provide routine iron supplementation to children in The Gambia and most children are not regularly screened for anaemia or IDA. All our children will be closely monitored during the study and all children that remain anaemic (Hb<11 g/dL) at the end of the study period (3 months intervention + 1 month AE follow-up), irrespective of their treatment group allocation, will be provided with iron supplementation for 3 consecutive months according to national and WHO guidelines. Severely anaemic children (i.e. Hb<7 g/dL) will not be enrolled in the study and if during the study a child (in any group) is found to become severely anaemic, they will stop participating in the study and will be offered iron supplementation for 3 consecutive months according to national and WHO guidelines. These children in the data analysis will be regarded as treatment failures. A full rationale for the inclusion of the placebo group is provided in Section 11.1.1.

- GMP manufacture of the clinical batch (low): as mentioned above, based on the feedback received for the scale-up manufacture of IHAT from CMOs, we are reassured that there won't be any significant hurdles in accommodating larger scale GMP manufacture. For the trial proposed here, the clinical batch of IHAT will be manufactured under cGMP conditions by Shasun

Pharma Solutions in the UK and the powder-filling of the capsules, packaging and labelling will be provided by Capsugel in Belgium, also under cGMP conditions.

- Recruitment of participants (low); mitigated by highly experienced staff and well engaged communities in The Gambia. Assuming that ~ 50% of children under 3y in the Upper River region would fit the study eligibility criteria (the prevalence of low ferritin in this age group is based on our current data from the HIGH study) then we would need to screen ~1500 children. There are ~1700 children 6-35 mo. of age in the Wuli and Sandu districts in the North Bank within a 20 km distance of the Basse field station from where we would initially recruit into the study. We would start recruitment in Nov 2017 for the 1st cohort and would finish in May 2018 for the 3rd cohort. We will monitor recruitment rates closely and if necessary we can extend the catchment area. The catchment area of the Upper River Division will be sensitised and a team led by the local PI (Clinical Trial Coordinator) and the field coordinator will tour all the villages in this area to sensitise them on the project.

- Increased malaria and co-infections risk during and after oral iron supplementation; there are risks associated with a large intake of iron supplements especially in areas of malaria endemicity. The dose of iron given daily in the reference arm (12.5 mg) is according to WHO guidelines for the age group children in non-malarious areas or malaria-endemic areas where it should be implemented in conjunction with measures to prevent, diagnose and treat malaria and co-infections. We have put in place the following strategies to mitigate the risk of possible interactions between iron supplements and malaria or other co-infections: (1) data from the Gambia over the last 5 years (medical records from the Kiang West region) shows that the peak malaria months are October and November and, therefore, we have timed the study intervention periods to avoid these months so that there will be no intervention/supplementation during Oct and Nov 2017 (2) trained field workers will be visiting all children every day during the 12 weeks supplementation period in order to supervise the administration of the iron supplements or placebo and on these occasions they will check on the children's health status and actively look for signs of malaria and co-infections, if a child shows signs of these infections the study nurse will perform adequate tests and the child will be offered the appropriate treatment/referral to the next Health Centre. In case of a fever, a malaria rapid test will be performed and if positive the child will be treated according to national guidelines. A sick child will always be visited by a study nurse for further clinical investigations and if needed referred to the nearest Health Centre. These visits will carry on for 4 weeks after the end of the study intervention and, during both the intervention and this follow-up period, morbidity data will be captured every other day. This is similar to what was done in the HIGH study and we do not anticipate any difficulties of implementation. Every week, the investigators will check whether children are RDT positive. Note: according to national guidelines, all children at birth are provided with bed nets and children in the URR receive intermittent preventive treatment (IPT) of malaria and so we have not anticipated we would need to provide insecticide-treated bed nets to the study population, but we could do this is deemed necessary.

- Analytical (low); mitigated by high level expertise of the laboratory technicians at the MRC Unit The Gambia at LSHTM and the internationally-recognised excellence of the Sanger Institute Group in microbiome and pathogen analysis and the expertise of the group at Kings College London in NTBI analysis.

2 Study objectives

In this trial we will test the hypothesis that supplementation with IHAT eliminates iron deficiency and improves haemoglobin levels in young children without increasing infectious diarrhoea or promoting negative changes in the gut microbiome or inducing gut inflammation.

There are four null hypotheses in the study:

1) non-inferiority of IHAT compared to ferrous sulphate for efficacy (in terms of Hb and iron deficiency correction: i.e. IDA) response probability (or prevalence). In this case we are testing the null hypothesis that: response probability in the IHAT arm minus the response probability in the ferrous sulphate arm is less than or equal to – 0.1. **We define 'response' for IDA as correction of iron deficiency and either achieving a normal Hb or an increase of at least 1 g/dL after 12 weeks of iron supplementation.**

2) superiority of IHAT compared to ferrous sulphate in terms of incidence density of diarrhoea. Here the null hypothesis is that the mean number of new episodes in the IHAT arm is greater than or equal to the mean number in the $FeSO_4$ arm.

3) superiority of IHAT compared to ferrous sulphate in terms of prevalence of diarrhoea. Here the null hypothesis is that the prevalence of diarrhoea in the IHAT arm is greater than or equal to the prevalence in the $FeSO_4$ arm.

4) non-inferiority of IHAT compared to placebo in terms of prevalence of diarrhoea. Here the null hypothesis is that the prevalence of diarrhoea in the placebo arm minus the prevalence in the IHAT arm is less than or equal – 0.1.

The primary objective for this trial is to show non-inferiority of IHAT in relation to ferrous sulphate at correcting IDA, and in terms of diarrhoea to show superiority in relation to ferrous sulphate and non-inferiority in relation to placebo (as defined above). Secondary objectives are to show that IHAT is non-detrimental with respect to enteric pathogen burden, the gut microbiome, and intestinal inflammation.

2.1 Study endpoints

Primary endpoints:

There are 4 primary endpoints of the trial:

1. iron deficiency at 12 weeks

2. haemoglobin levels at 12 weeks

3. 'incidence density' of moderate-severe diarrhoea over the 12 weeks (i.e. the number of new moderate-severe diarrhoea episodes per child over the 12 weeks intervention)

4. 'period prevalence' of moderate-severe diarrhoea over the 12 weeks intervention period (i.e. the proportion of children with at least one episode of moderate-severe diarrhoea over the 12 weeks intervention)

Efficacy primary endpoints:

To assess iron deficiency we will take into consideration the most up-to-date recommendation from WHO who are currently conducting a consultation on this matter. The new WHO guidelines will include a recommendation for the best marker of iron deficiency in the context of inflammation and we expect this to be either using ferritin alone or the sTfR/logferritin index, where in both cases ferritin values will be inflammation-adjusted. There are several models we can use to adjust ferritin for inflammation, for example use unadjusted ferritin with the cut-off with optimal sensitivity and specificity from the Malawi study (<18 μg/L), use the CRP/AGP regression model being investigated by Parmi Suchdev (BRINDA) to adjust ferritin values, use the Thurnham et al (38) stratified adjustment or the continuous adjustment used by Engle-Stone et al (39). The choice of the method for adjusting ferritin concentrations and of which marker to use to define ID (ferritin alone or the sTfR/logferritin index) will be made at the time of locking the data analysis plan, in consultation with an expert on this matter (Dr Sant-Rayn Pasricha), our BMGF Program Officer (Dr Ken Brown) and taking into consideration the results from Brinda and the results from the ongoing WHO consultation on this matter.

Iron deficiency and haemoglobin levels at 12 weeks will be used to define the prevalence of IDA which we will use to assess non-inferiority of IHAT relative to $FeSO_4$ in terms of efficacy. **We will determine the proportion of children who resolve iron deficiency <u>and</u> either achieve a**

normal Hb or an increase in Hb of at least 1 g/dL after 12 weeks of iron supplementation.

Safety primary endpoints:

Incidence density and period prevalence of moderate-severe diarrhoea will be used to show superiority of IHAT in relation to ferrous sulphate and non-inferiority of IHAT in relation to placebo.

Diarrhoea is defined as 3 or more loose or watery stools per day and **moderate-severe diarrhoea** refers to those diarrhoea episodes where (i) the child passes more than 5 loose or watery stools per day, (ii) there is blood in the stool (dysentery), or (iii) the child shows signs of clinical dehydration (assessed by the study nurse based on physical signs such as little or no urination, sunken eyes, and skin that lacks its normal elasticity). These episodes will require treatment (including ORS) and will be recorded as adverse-events.

'Incidence density' is defined as the number of new moderate-severe diarrhoea episodes per child over the 12 weeks intervention and 'period prevalence' is defined as the proportion of children with at least one episode of moderate-severe diarrhoea over the 12 weeks intervention.

Secondary endpoints:

Secondary endpoints will be faecal microbiome diversity and profile (particularly in terms of abundance of Enterobacteria), abundance of enteric pathogens, faecal calprotectin (marker of gut inflammation), hospitalisation and morbidity (data collected three times per week using the questionnaire developed in the HIGH study), malaria infection, treatment failures (i.e. the number of children who have to stop the study because their Hb falls below 7 g/dL), the proportion of days a child has diarrhoea over the 12 weeks intervention period ('longitudinal prevalence' of diarrhoea), the proportion of days a child has moderate-severe diarrhoea over the 12 weeks period ('longitudinal prevalence' of moderate-severe diarrhoea), 'incidence density' of bloody diarrhoea (i.e. the number of bloody diarrhoea episodes per child-month of observation), markers of systemic inflammation (serum CRP and AGP), and systemic markers of iron handling (hepcidin, sTfR, transferrin saturation and circulating non-transferrin bound iron - NTBI).

sTfR and hepcidin will be assessed at 0, 12 weeks and all other outcome measures will be assessed at 0, 2 and 12 weeks in all the children.

The 2 weeks time-point will provide an indication of acute compound-related effects and the 12 weeks time-point of chronic (i.e. longer term/chronic) effects.

3 Study design

3.1 Type of study and design

3-arm, parallel, randomised, placebo-controlled, double blind.

Children will be randomised (1:1:1) to IHAT, ferrous sulphate or placebo, each arm will include an intervention period of 12 weeks, faecal and blood samples will be collected at the time-points indicated below.

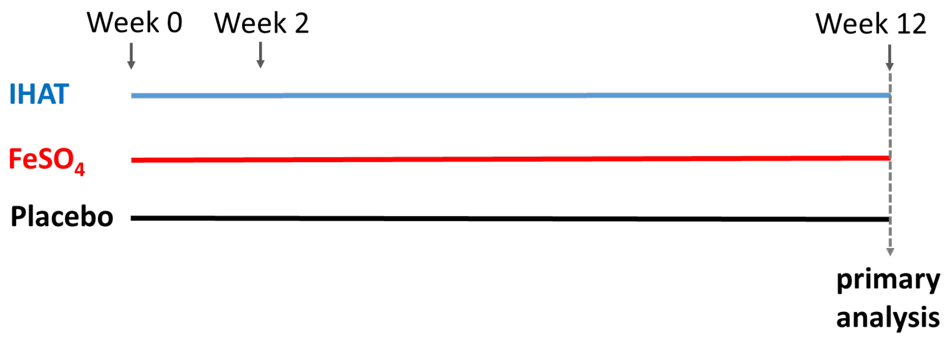

Participants will be iron deficient anaemic young children (n=705, accounting for 15% loss to follow-up) living in rural communities in the Upper River Division in The Gambia. Children will be recruited and randomised into the 3 study arms (n=235 per arm). Inclusion criteria will be: apparently healthy as judged by a study nurse on the day of recruitment, age 6-35 months, no malaria (RDT negative) and IDA defined as 7≤Hb<11 g/dl (for recruitment we will use Medonic Hb values rather than HemoCue) with serum ferritin<30 µg/L.

Each arm will include an intervention period of 12 weeks. As suggested by our BMGF proposal reviewers, we have included a placebo arm to allow us to fully evaluate IHAT effects, particularly

to rule-out negative impact on diarrhoea episodes, the gut microbiome and compound-related bacterial infection, and to determine treatment effect sizes with either IHAT and ferrous sulphate that will be necessary to power future iron supplementation studies (please refer to Section 11.1.1.).

3.2 Randomisation and blinding procedures

3.2.1 Randomisation

Recruited children (N=705) will be randomly assigned (computer generated) to receive one of the 3 treatment arms (n=235 in each arm) stratified by the Hb concentration prior to enrolment and age. This will assure that Hb concentration at baseline (as a proxy for erythropoietic demand, which is the main driver for iron absorption) will not differ between the 3 arms.

At recruitment, each child will be categorised into two Hb classes (below or equal to/above the median Hb for that recruited cohort) and also according to age into 3 classes (6-11 months, 12-23 months and 24-35 months). This will divide children into 6 different strata and in each strata the children will be randomly assigned to one of the 3 treatment arms using a computer program and a block randomisation approach with fixed block size by age and Hb levels as defined above.

Ideally subjects will be recruited so that there will be balanced numbers in each of the 3 age classes; each of the age classes are further categorised into two groups based on the Hb level. Thus, in the balanced case, a total of 117-118 subjects each will belong to one of the six strata, created by Hb level (Low, High) and age group (Young, Middle, Old) combinations:

a. Low Hb, young age group
b. Low Hb, middle age group
c. Low Hb, old age group
d. High Hb, young age group
e. High Hb, middle age group
f. High Hb, old age group

However, 204 random treatment allocations will be generated for each strata in order to allow flexibility with respect to the numbers recruited in each of the six groups (a)-(f). That is, the list of treatment sequences in the balanced case will be extended by 86-87 to account for inbalance in the ages of children enrolled in the study as it may not be possible due to the age demographics of the study communities to enrol equal numbers in each of the age classes above. In any case,

we will ensure that equal numbers within each strata are assigned to one of the treatment arms. This will be based on a pre-specified list of IDs ranging from 001-204, 205-408, 409-612, 613-816, 817-1020 and 1021-1224 for groups (a)-(f) above, respectively.

The study supplements will be provided in 750 individual bottles from the manufacturer. These bottles will be labelled using a random bottle ID ranging from 1 to 750 with a check digit character. The check digit character will be augmented to each of these numbers to make up the full subject IDs which will be generated using the Damm algorithm (https://en.wikipedia.org/wiki/Damm_algorithm; date accessed 08 November 2016). Here the check digit numbers 0-9 will be converted to letters A-H and J-K. Thus, there will be 250 bottles with randomly assigned bottle IDs for each of the three treatments (A, B or C). An electronic copy of the list will be maintained to allow electronic randomisation. The key code to which IMP (IHAT, $FeSO_4$ or placebo) corresponds to A, B or C will not be known to the statistician running the randomisation nor to anyone in the study team.

After children in each cohort are classified into one of the six strata based on their age and Hb level, a single bottle will be assigned to the child by selecting a bottle from one of the three groups (A, B or C) according to the treatment allocation, as determined above. The procedure will be repeated until the target number of subjects (n=705) are randomised. This will be performed using a computer programme. A complimentary programme will also be developed with a Microsoft Access application that will generate the appropriate bottle ID for a given screening ID which can be used on site. The bottle IDs will be used as subject/randomisation IDs and written on all relevant forms.

The randomisation code and the application to generate subject IDs on site will be written by Nuredin Mohammed (the trial statistician based at MRCG) and reviewed by David Jeffries (the head of Statistics MRCG). The final code will be run using a random seed number by a member of the Statistics department who is independent of the trial. The generated randomisation list and the seed number will be recorded in a randomisation database (MS access database). The database and the application will be password protected and stored in a secure server.

After randomisation, the allocation list will be kept electronically and access will be granted to an independent clinician/nurse at the Basse site. A paper copy of the allocation list will be kept securely in the Sponsor's office (by an independent statistician). The DSMB will have access to grouped data but we would provide unblinded data to the DSMB for their closed meetings if requested. If emergency unblinding is required at the request of the DSMB, only the particular study subject in question will be unblinded, since we will have an individual supplement code for each participant. Following investigation by the DSMB of an emergency unblinding case, and at the request of the DSMB, we may also unblind a whole treatment arm or the entire study. In all cases, someone independent from the trial team will perform the unblinding.

3.2.2 Blinding

Participants, field workers (Senior Field Assistants, Field Assistants, Field Supervisor and Field Coordinator), study nurses, scientific officers, research clinician, local PI (Clinical Trial Coordinator), CI, Clinical Trial Monitor and the entire study team will be blinded as to which treatment group participants belong to. Each treatment dose (iron compounds and placebo) will be encapsulated in identical capsules (also containing powders of identical colour) by Capsugel in France and the supply of capsules for each child will be packed in one bottle that will be individually labelled with a random bottle ID ranging 001-750 with a check digit character. This means that each child will have a unique treatment code that will also be their study ID/randomisation number as specified in 3.2.1. This individual labelling has the advantage of allowing to unblind only one child, if it becomes necessary, without compromising the blinding of the study.

The manufacturer will provide 750 individual bottles (allowing for some spares) comprising 250 bottles with randomly assigned bottle IDs for each of the three treatments.

MRCG will pass on the randomisation list to Capsugel with the treatment code key (A, B, or C) corresponding to each of the bottle IDs and Capsugel will make the decision of which compound is A, B or C. A Sponsor representative will keep the allocation list safe and since Dr Pereira (co-PI) will also remain blinded, we suggest an independent statistician acts as the key holder for the treatment arm codes and allocation list. This list will be kept in electronic format (password protected) and in paper copy (locked cabinet).

The pre-packed weekly supplies of the capsules will be prepared following a study-specific procedure (SSP) by the Scientific Officer (SO) and the process supervised by the clinical trial coordinator (local PI) in Basse and labelled with each participant’s ID, they will then be handed over to the Field Coordinator who is responsible for distribution to the field workers who will supervise consumption according to the pre-defined protocol for administration of the oral doses. Staff will be fully trained in all aspects of this activity and quality-control measures will be put in place to ensure that the SSP is followed exactly.

The blinding for a particular study participant may be broken if safety issues arise and we are advised by the DSMB to do so. A study-specific procedure describing the unblinding process will be developed.

3.3 Sub-studies

None.

3.4 Investigational products

3.4.1 Description of products

The trial has 3 arms:

IHAT- iron hydroxide adipate tartrate: an analogue of natural food ferritin iron (as detailed in Section 1.2); **Ferrous sulphate**- the gold standard for iron supplementation and **Placebo** (sucrose).

To avoid the need to use any tablet compression excipients or encapsulation materials, and to investigate the effects of just the active IMP compounds, the iron preparations, i.e. IHAT and ferrous sulphate, and placebo comparator, will be supplied as powders with each dose contained in a hard-gelatine powder-filled easy-open capsule.

3.4.2 Formulation, packaging and labelling

Each daily dose will be contained in a hard-gelatine powder-filled easy-open capsule. Each capsule will contain the single active IMP compound (IHAT or ferrous sulphate) or the placebo compound mixed with a small amount of a food colorant to colour-match the IMP powders. Capsules for each treatment will be packed in medicine bottles, each bottle containing enough capsules for one child for the entire study duration (allowing for some spares in case of accidental loss), and these bottles will be individually labelled with a unique code per participant, which will also be the subject randomisation code as described above. The key to which treatment corresponds to which code will be unknown to the study team or study PIs. The weekly supplies of each treatment for each child will be packed in Basse by the SO supervised by the clinical trial coordinator (local PI) and labelled with each participant's ID, which will be the same as the bottle ID as described in the randomisation procedure above. The bags with the weekly supplies per child will then be handed over to the Field Coordinator who is responsible for distribution to the field workers who will supervise consumption according to the pre-defined protocol for administration of the oral doses.

3.4.3 Product storage and stability

The capsules will be stored in dry conditions (the capsules will be packed in bottles containing desiccator sachets incorporated in the lids) and away from direct sunlight at MRCG Basse (this will be in an air-conditioning room in MRCG Basse clinical services below 25°C). The products are stable for 24 months if kept below 25°C. A full long-term storage stability assessment is going to be performed by Capsugel, the formulation company preparing the capsules (following recommended ICH protocols for the Gambia climate region: 25°C/60%Relative Humidity (representing normal storage conditions) and 30°C/65%Relative Humidity (representing transport conditions)) for both IHAT and comparators and the capsules will be packed and stored accordingly to these findings (these data will be available prior to study starting when the cGMP capsules are released by Capsugel to be shipped to the Gambia).

The weekly supplies taken out to the field do not require any special short-term storage conditions and will be kept by the field workers in their study bags in sealed plastic bags.

3.4.4 Dosage, preparation and administration of investigational products

The very recent WHO recommendation for the iron supplementation daily dose is 10-12.5 mg elemental Fe for infants 6-23 mo. and 30 mg elemental iron for children 24-59 mo. (40). The iron dose for ferrous sulphate will be 12.5 mg elemental Fe (~38 mg ferrous sulphate monohydrate) once a day for our entire study population (6-35 mo.), rather than having 2 different dosages according to the age groups, as this is still in line with the Gambian national guidelines. The iron dose for IHAT will be the iron-bioavailability equivalent (bioequivalent) in relation to ferrous sulphate as defined above in Section 1.2, i.e. assuming RBV of 60%. The bioequivalent iron dose for IHAT will be 20 mg elemental Fe (equivalent to 80 mg IHAT powder).

Each daily dose will be contained in a hard-gelatine powder-filled easy-open capsule. On the day of administration, the field assistant will open the respective capsule (corresponding to the child study ID according to the randomisation) and add the entire powder content of the capsule to 10 ml of a sugar juice drink contained in a disposable plastic cup. The drink will be used to mask the metallic taste of the iron powders, so that the supplementation is more acceptable to the child. The dose will be administered directly into the child's mouth using a disposable Pasteur pipette (in the younger children), a big spoon or drunk directly from the plastic cup (in the older children). There will be a Study Specific Procedure for this dose-administration protocol, which will be followed by each field worker and specific training will be provided before the start of the study. Whenever possible, each dose will be ingested after a feed or within 1 hour of the last meal, since iron supplements are generally better tolerated when ingested after meals rather

than on an empty stomach. If, exceptionally, the child has not been fed before supplementation, the mother will be encouraged to feed the child immediately after supplementation.

In order to be able to do this protocol for supplement administration and not compromise the double-blind design of the trial we have been working with the formulation company (i.e. Capsugel) producing the capsules to ensure that all powders (IHAT, ferrous sulphate and placebo) have the same colour and a small amount of a food colorant will be added to all the powders prior to filling the capsules). The IHAT powder will be produced by Shasun Pharma Solutions in the UK (now called Sterling Pharma) and sent to Capsugel who will formulate the IHAT and source and formulate the ferrous sulphate and placebo (sucrose) in the gelatine capsules.

3.4.5 Concomitant medications/treatments

Participants will remain under the care of the Wuli and Sandu RCH teams.

Any subject whose Hb falls below 7g/dL will be referred to the closest health centre to receive iron supplementation for 3 consecutive months according to national and WHO guidelines. We will support the RCH systems by ensuring a constant supply of malaria diagnostics, anti-helminth treatment, basic drugs and iron syrup (for all young children – not just study participants) for the duration of this trial. All children participating in the study who remain anaemic after the study intervention and follow-up period (i.e. after the 4 months) will be offered the iron syrup for 3 consecutive months according to national and WHO guidelines.

No specific medication is prohibited during the study, with the exception that participants in the study are not allowed to take any other form of iron supplementation, including multimicronutrient powders containing iron and iron syrups for the entire duration of the study, i.e. during the 12 weeks intervention and 4 weeks AEs follow-up.

4 Selection and withdrawal of participants

4.1 Selection of participants

Since the welfare card/immunisation cards in the North Bank of the URR are not available for all children and records are unreliable, children between the age of 6-35 months will be identified through the enumeration data collected by the study field team within the study catchment area in the Wuli (17 study villages) and Sandu (12 study villages) districts (distance of ~ 20 km North of Basse, Figure 4). The compounds within the study catchment area were identified from the mapping exercise conducted by our field team. These mapping and enumeration/census data are currently being collated in an electronic database and will be linked with the eCRFs.

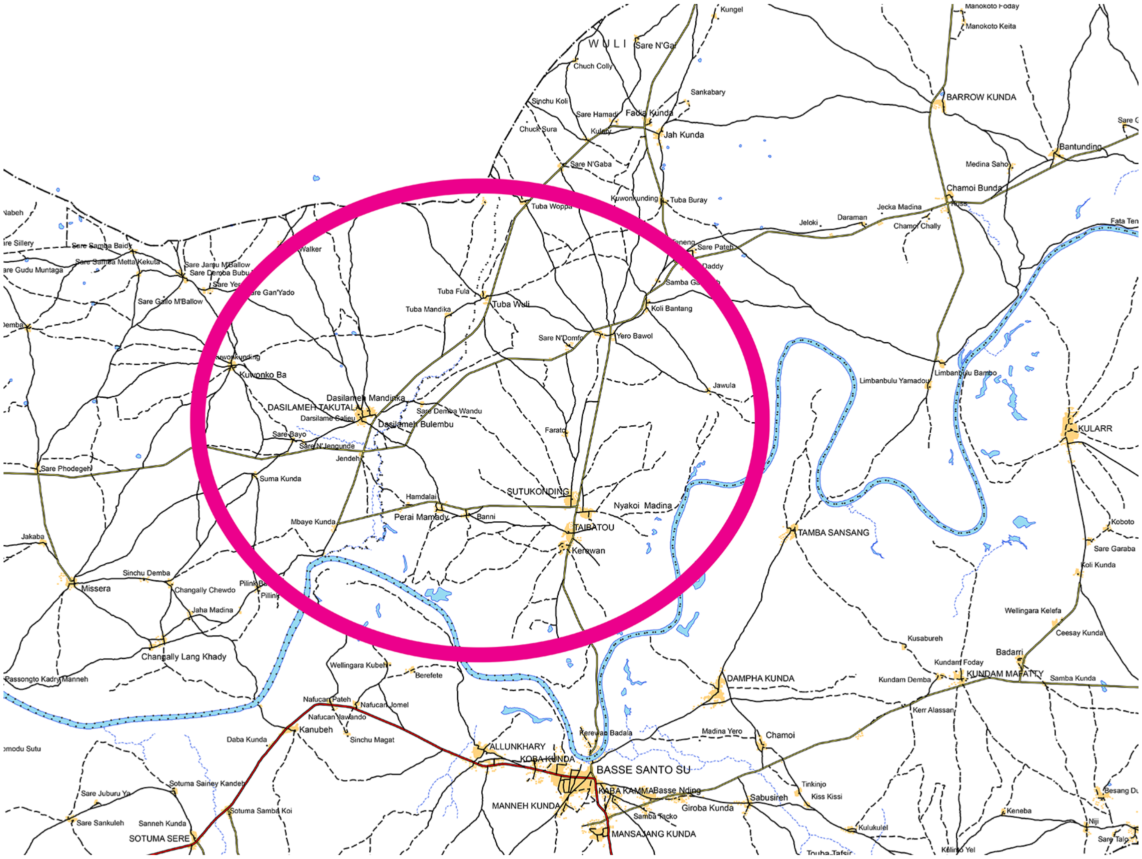

Figure 4. Study Catchment area. The area demarked represents an area of ~ 15 Km radius (at a maximum distance of 20 km by straight line from Basse), and corresponds to an area perimeter of ~ 45 km. Study nurses will be stationed at the following health facilities in the Nortn Bank of the URR: Yorrobawol Health Center, Darsilami Community Health Post, Konkuba Community Health Post, and Taibatu Health Post.

Assuming that ~ 50% of children under 3 y. in the Upper River Region would fit the study eligibility criteria (the prevalence of low ferritin in this age group is based on our current data from the HIGH study) then we would need to screen ~1500 children. From our community census

exercise, we estimate that there are ~1700 children 6-35 mo. of age in the Wuli and Sandu districts in the north Bank within a 20 km distance of the Basse field station from where we would initially recruit into the study. We would start recruitment in Nov 2017 for the 1st cohort and would finish in May 2018 for the 3rd cohort. We will monitor recruitment rates closely and if necessary we can extend the catchment area.

The catchment area of the Upper River Division will be sensitised and a team led by the local PI (Clinical Trial Coordinator) will tour all the villages in this area to sensitise them on the project. For the sensitisation we will use the Study Communication Plan developed together with the MRCG Communications Team.
The Regional Health Team (RHT) and MRCG Basse will advise on all studies taking place in the area. All mothers/guardians of eligible children will be asked if their child participates in another study and children will only recruited if this is not the case. They will also be asked if they intend to remain in the study catchment area for the entire duration of the study period (i.e. 4 months).

4.2 Eligibility of participants

Participants must meet all of the inclusion criteria and none of the exclusion criteria to be eligible to participate in the trial.

4.2.1 Inclusion criteria

Apparently healthy as judged by a study nurse at day of screening and recruitment
Age 6-35 mo.
Free of malaria (RDT negative)
HAZ, WAZ, WHZ >-3 SD
IDA defined as 7≤ Hb <11 g/dl AND ferritin<30 µg/L, as per WHO recommendation for children under 5y that live in regions with high infection burden (14)
Resident in the study area (and planning to remain in the study area for the duration of the trial)
Ability and willingness to comply with the study protocol (daily intake of supplement and daily study visits with weekly finger prick)
Informed consent given by parent or guardian

4.2.2 Exclusion criteria

Congenital disorders

Chronic disease
Currently participating in another study
Currently taking iron supplements/multiple micronutrient supplements
Currently experiencing moderate-severe diarrhoea, defined as those diarrhoea episodes where (i) the child passes more than 5 loose or watery stools per day, (ii) there is blood in the stool (dysentery), or (iii) the child shows signs of clinical dehydration (assessed by the study nurse based on physical signs such as little or no urination, sunken eyes, and skin that lacks its normal elasticity), will usually require treatment (including ORS)

4.3 Withdrawal of participants

A study participant will be discontinued from participation in the study if:
- Hb concentration falls < 7 g/dL
- Any clinically-significant adverse event (SAE), laboratory abnormality, intercurrent illness, or other medical condition or situation occurs such that continued participation in the study would not be in the best interest of the participant
- Development of a chronic disease
- Participation in another study

Note that there is no formal withdrawal criteria for participants that develop moderate-severe diarhoea episodes since this is a primary outcome of the trial. However, children that show clear signs of infectious diarrhea, such as blood in the stool, will be provided with appropriate treatment and the causative agent for the infection will be determined using the TaqMan Array Card (TAC) system (41).

Participants are free to withdraw from the study at any time without giving a reason.

5 Study procedures and evaluations

5.1 Study schedule

5.1.1 Study sensitisation

The Management and Field team of MRCG Basse have already been engaged with the proposed trial and have offered their support. An initial mapping of the catchment area has also been carried out by the Field Coordinator and visits to some of the health clinics in the area were conducted. The Regional Health Team (RHT) and staff of the health facilities responsible for the

catchment areas of Wuli and Sandu have been sensitised and approval from the Director of Health Services to proceed with the study in the North Bank of the URR has also been granted. The study nurses will be based at the following health facilities in the Nortn Bank of the URR: Yorrobawol Health Center, Darsilami Community Health Post, Konkuba Community Health Post and Taibatu Health Post. A team lead by the local PI (Clinical Trial Coordinator), the Research Clinician and Field Coordinator will tour all the communities to sensitise them on the study during April-early May 2017. The community sensitisation will involve village to village sensitisation and a sentisation program in Yorrobawol and Darsilami where satellite village elders, Alkalos and the chiefs will be invited. The attached information sheet will be used as the basis for the sensitisations.

5.1.2 Screening and enrolment (baseline)

Young children in the participating communities will be identified using the enumeration/census data currently being collected by the study field team within the study catchment area (Figure 5). At screening, once mothers/guardians of the child have signed the informed consent form, the child will be physically examined by a study nurse and, if the child is considered as generally healthy (e.g. no fever, not severely malnourished), their height and weight will be measured and a finger prick blood sample will be collected for Hb and RDT testing at one of the health facilities supervised by the study nurses (Figure 4). If z-scores are >-3, 7 ≤Hb< 11 g/dL and the RDT is negative, then a small venous blood sample (1 mL in total divided into 0.5 ml EDTA and 0.5 ml serum blood collection tubes) will be collected to confirm the Hb levels (Medonic analyser) and determine serum ferritin (Cobas analyser). If 7 ≤Hb< 11 g/dL and serum ferritin < 30 ng/ml, the child will be eligible to enrol in the study. Children with Hb < 7 g/dL will not be enrolled and will be referred to the regional health centre for treatment according to national guidelines. Children with Hb ≥ 11 will not be enrolled as they don't need iron. Malaria positive children (positive RDT and confirmation by blood film) will not be enrolled and will be treated according to national guidelines. Severely malnourished children will also be excluded.

Screening will be divided into 3 cohorts, each recruitment period will be of approximately 1 month prior to enrolment into each of the sequential cohorts and we expect to screen ~ 500 children in each cohort or 50 children/day for 2 weeks, with 2 weeks left to get all screening results back from the lab.

Prior to enrolment (Day 0), which will be a maximum 4 weeks after the screening visit, eligible children will be taken back to the clinic for a finger prick to confirm absence of malaria by RDT and haemoglobin concentration by hemocue. Children will be called back in clusters of 58-60 on a Monday, Tuesday, Wednesday and Thursday. This is necessary before they can be enrolled and randomised to the study because of the time it takes to get all the screening results back from the labs for all children in that cohort (i.e. around 4 weeks). On the Friday of the same week, the

list of all the children that remain RDT negative and with 7 ≤Hb< 11 g/dL will be sent to the study statistician who will run the randomisation to one of the three study arms, stratified by Hb and age as described in 3.2.1., and send the list with the study IDs to the PIs that same day.

The week after, children will be taken back to the clinic again in the same cluster (i.e. either on the Mond, Tue, Wed or Thur as per their Day 0 cluster) and this will be their study Day 1 (see below).

We will enrol children in 3 cohorts (n=235 children each) that will run sequentially.

Each study cohort will be organised as follows. Each cohort will have 4 clusters (58-60 children per cluster), children in each cluster will be allocated to one of the study health facilities according to the child's compound proximity to each of the 4 study health facilities. Children in the first cluster will all have their study visits on a Monday, children in the second cluster will all have their study visits on the Tuesday, and the third cluster on the Wednesday and fourth cluster on the Thursday. Children in each cluster will also have their weekly check-ups at the study health facility on the same day of the week and would have had their Day 0 visit on the same day of the week.

5.1.3 Follow-up (study visits)

Each arm will include an intervention period (follow-up) of 12 weeks plus an additional active follow-up period of 4 weeks post intervention. As suggested by our grant proposal reviewers, we have included a placebo arm to allow us to fully evaluate IHAT effects, particularly to rule-out negative impact on diarrhoea episodes, the gut microbiome and compound-related bacterial infection, and to determine true treatment effect sizes with both IHAT and ferrous sulphate (for more details refer to Section 11.1.1.).

On study Day 1, we will take a photo of the child (with consent) and print and laminate a study ID card that we will ask the mother to keep safe during the study. This ID card will contain the photo of the child and their randomisation/study ID number. We will also provide the child with a wrist band showing the same study ID number so that there is no confusion regarding the identity of the study participants. This was considered necessary in the context of the North Bank of the URR since there is a poor health record system in place and most children do not have welfare cards. Then, we will collect a venous blood (total of 5 ml divided into 1 ml EDTA, 1 ml LH and 3 ml serum collection tubes) and a stool sample from the children (baseline samples). Blood sample collection will be done either before the first meal of the day or, in cases where this is impossible, at least 1 hour after the last meal (so that the more dynamic iron parameters, such as hepcidin and NTBI, are not influenced by the iron absorption from the previous meal). Stool

samples may be collected by the mother at home on the morning of the study visit using the toilet pots and disposable liners supplied by the study team for each child. The field worker will then aliquot the stool sample into the stool sample pots to be brought to the study clinic as soon as feasible after collection of the sample and will transport them to the clinic, when it is not possible to collect the sample from the child at the clinic.

On Day 1, we will also collect demographic and immunisation data and the morbidity questionnaire will be completed.

At the end of this visit, the mother will be encouraged to feed the child and once the child is settled, they will be given the iron supplementation or placebo. The treatment arm intervention will be administered directly into the child's mouth homogenised in 10 ml of a sugar juice drink. The iron or placebo powder doses will be added to the small amount of juice in a disposable plastic cup immediately before administration, this will be done by the field worker. This first administration of the study supplement/placebo to a child will be supervised by the study nurse and/or the study clinician to offer extra reassurance to the mother.

Highly trained and experienced field workers will be visiting all children every day during the 12 weeks supplementation period in order to administer the iron supplements or placebo and on these occasions they will check on the children's general health and actively look for signs of malaria and co-infections. If a child shows signs of these infections, the field worker will refer to the study nurse who will perform adequate tests and the child will be offered the appropriate treatment/referral to the next health center. Prior to study start, all field workers will receive appropriate training, provided by the study nurses and supervised by the study clinicians, on which AEs and SAEs to look out for and on when to refer the children to the study nurses for follow-up. Three times per week, morbidity data (including questions regarding fever, diarrhoea, vomiting, cough, malaria symptoms, any other illness, appetite and any mediation taken and assessment of body temperature) will be captured (as part of the CRF). Every week children will be taken back to the health facility in their cluster for a check-up and a finger prick to determine their malaria and Hb status. Hb will be determined with HemoCue. RDT testing will be used for malaria and children found with a positive RDT during the study will be further tested with a blood film and treated according to national guidelines. These check-ups will continue 4 weeks post intervention to follow-up on AE/SAEs.

Each field assistant (FA) will be responsible for 10-12 children within each cohort of 235 children, and will be supported by senior field assistants (SFA) and field supervisors (FS). The study will have a field team comprising of 21 FA, 4 SFA and 2 FS. All will be managed by the Field Coordinator (FC). Each SFA will be responsible for supervising the FA working in the study cluster

comprising 58-60 children. The FS will each be responsible for the communities in the Wuli and Sandu districts, respectively.

Every day during the intervention period of 12 weeks, the treatment arm interventions will be administered directly into the child's mouth homogenised in 10 ml of a sugar juice drink. The iron or placebo powder doses will be added to the small amount of juice in a disposable plastic cup immediately before administration, this will be done by the field worker. Before supplementation, the mother will be encouraged to feed the child so that the supplements are not ingested on an empty stomach (as detailed in 3.4.4).

On Days 15 and 85, the child will visit the clinic and another stool sample and another venous blood sample (total of 5 ml divided into 1 ml EDTA, 1 ml LH and 3 ml serum collection tubes) will be collected. Height and weight will be measured at day 85.

Any child where Hb falls below 7 g/dL during the follow-up study period will stop the study supplementation and will be referred to the next health centre for management and will be provided with iron supplements for 3 consecutive months according to national and WHO guidelines. These children will be treated in the data analysis as treatment failures. These children will continue to be followed up for adverse events by the field team but not receive any of the study treatments.

At each study visit (i.e. day 1, 15, 85) we will check the child's welfare card or ask the mother and note in the CRF if there was a vaccine in the past week, since some of the acute inflammatory markers we are measuring, particularly CRP, can transiently increase after immunisation (42, 43).

At the end of the follow-up period (12+4 weeks), the children in any arm who still have anaemia (Hb<11 g/dL) will be provided with iron supplementation for 3 consecutive months as per national and WHO guidelines.

5.1.4 Final study visit

The final study visit will be on day 113 (in the post-intervention follow-up period) when a finger prick blood sample will be collected and those children that remain anaemic will be offered iron supplementation for 3 consecutive months according to the national and WHO guidelines.

5.1.5 Early termination visit

An early termination visit may occur in this study because of a participant's voluntary withdrawal, trial team decision or at the discretion of the Data Safety Monitoring Board as described in Section 7. Apart from the safety evaluations, no other evaluations required for the final study visit will be done.

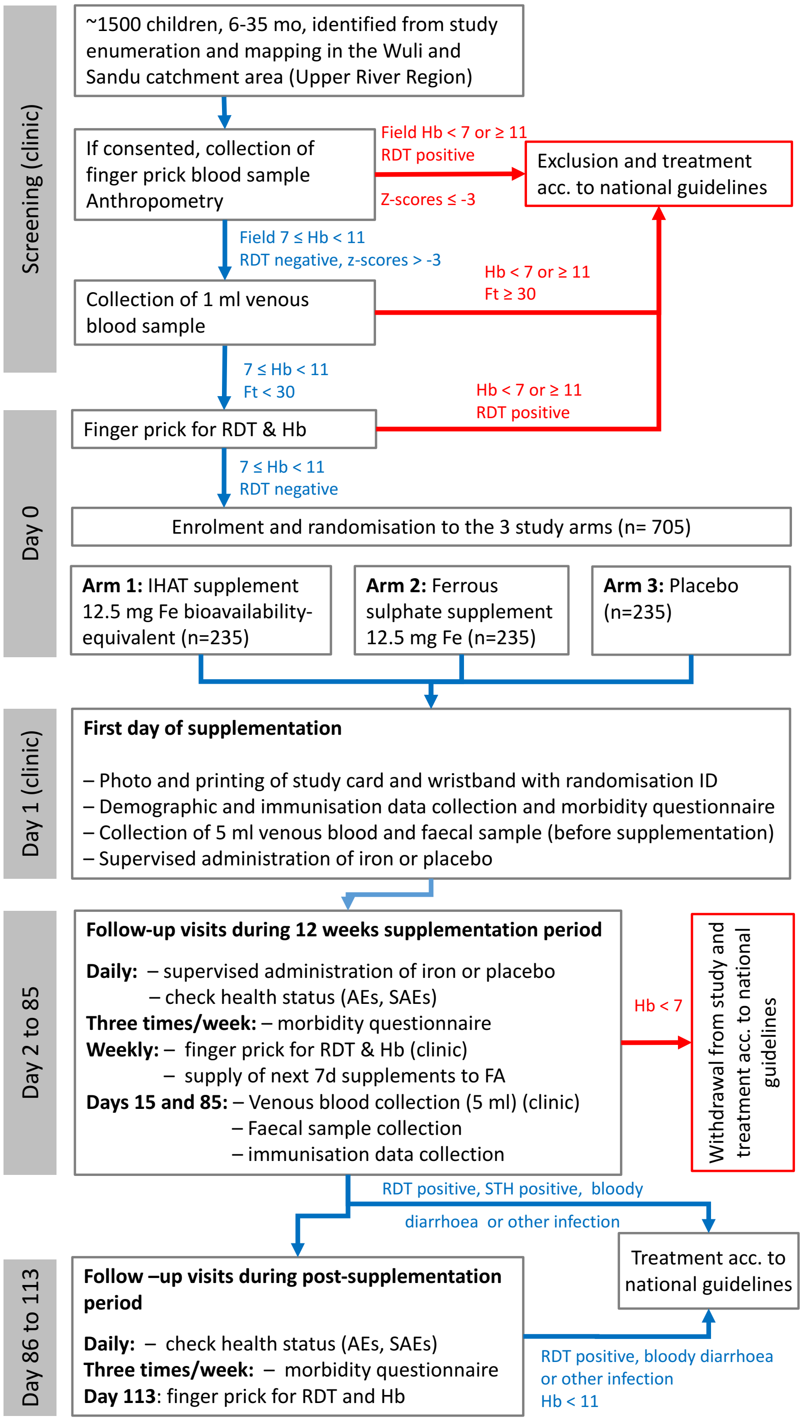
Screening (clinic)
~1500 children, 6-35 mo, identified from study enumeration and mapping in the Wuli and Sandu catchment area (Upper River Region)
If consented, collection of finger prick blood sample Anthropometry
Field Hb < 7 or ≥ 11
RDT positive
Z-scores ≤ -3
Exclusion and treatment acc. to national guidelines
Field 7 ≤ Hb < 11
RDT negative, z-scores > -3
Collection of 1 ml venous blood sample
Hb < 7 or ≥ 11
Ft ≥ 30
7 ≤ Hb < 11
Ft < 30
Day 0
Finger prick for RDT & Hb
Hb < 7 or ≥ 11
RDT positive
7 ≤ Hb < 11
RDT negative
Enrolment and randomisation to the 3 study arms (n= 705)
Arm 1: IHAT supplement 12.5 mg Fe bioavailability-equivalent (n=235)
Arm 2: Ferrous sulphate supplement 12.5 mg Fe (n=235)
Arm 3: Placebo (n=235)
Day 1 (clinic)
First day of supplementation
– Photo and printing of study card and wristband with randomisation ID
– Demographic and immunisation data collection and morbidity questionnaire
– Collection of 5 ml venous blood and faecal sample (before supplementation)
– Supervised administration of iron or placebo
Day 2 to 85
Follow-up visits during 12 weeks supplementation period
Daily: – supervised administration of iron or placebo
– check health status (AEs, SAEs)
Three times/week: – morbidity questionnaire
Weekly: – finger prick for RDT & Hb (clinic)
– supply of next 7d supplements to FA
Days 15 and 85: – Venous blood collection (5 ml) (clinic)
– Faecal sample collection
– immunisation data collection
Hb < 7
Withdrawal from study and treatment acc. to national guidelines
RDT positive, STH positive, bloody diarrhoea or other infection
Treatment acc. to national guidelines
Day 86 to 113
Follow –up visits during post-supplementation period
Daily: – check health status (AEs, SAEs)
Three times/week: – morbidity questionnaire
Day 113: finger prick for RDT and Hb
RDT positive, bloody diarrhoea or other infection
Hb < 11

Figure 5. Study Flow Chart. Abbreviations: Hb, haemoglobin; Ft, ferritin; RDT, rapid diagnostics test; STH, soil-transmitted helminths; AE, adverse events; SAEs, serious adverse events; FA, field assistant; Fe, iron.

5.2 Study evaluations

This trial is powered to test non-inferiority of IHAT in relation to ferrous sulphate at correcting IDA, and in terms of diarrhoea to show superiority in relation to ferrous sulphate and non-inferiority in relation to placebo.

Non-inferiority of IHAT in relation to $FeSO_4$ at correcting IDA will be based on the response probability in the IHAT and $FeSO_4$ arms at the Day 85 time-point, i.e. the proportion of children in each arm that resolve iron deficiency <u>and</u> achieve a normal Hb or an increase of at least 1 g/dL in Hb after 12 weeks of the intervention.

Superiority of IHAT in relation to $FeSO_4$ in terms of diarrhoea will be based in both 'incidence density', i.e. the mean number of new moderate-severe diarrhoea episodes per child over the 12 weeks intervention period, and 'period prevalence', i.e. the proportion of children with at least one episode of moderate-severe diarrhoea over the 12 weeks intervention period, of moderate-severe diarrhoea.

Secondary objectives are to show that IHAT is non-detrimental with respect to enteric pathogen burden, the gut microbiome, and intestinal inflammation.

Please refer to Section 8 for more details on this analysis and power of the trial.

5.2.1 Clinical evaluations

Health status of the children at enrolment will be assessed through a physical examination and blood screening results by a study nurse in consultation with the study clinicians. During the study period, diarrhoea, hospitalisation and morbidity data will be collected three times per week in the field using a morbidity questionnaire.

5.2.2 Laboratory evaluations

Blood samples:

The analysis bellow will be performed in the ***venous blood samples*** collected on study days 1 (baseline), 15 and 85 and blood will be taken at the health facilities in the catchment area. Sample collection will be either before the first meal of the day or, in cases where this is impossible, at least 1 hour after the last meal (so that the more dynamic iron parameters, such as hepcidin and NTBI, are not influenced by the iron absorption from the previous meal). Blood will be transported to the MRCG Basse laboratory for processing/analysis as soon as possible after collection.

Haemoglobin will be determined in EDTA anticoagulated blood (~500 µl) as part of a full haematology panel using the Medonic Haematology Analyser at MRCG Basse (the MRCG Quality team is currently working towards setting up the validation of the Medonic assay in Basse).

Four serum aliquots will be frozen (-20°C) at MRCG Basse on the day of blood collection for the following analysis. One aliquot (500 µl) where serum ferritin, sTfR (soluble transferrin receptor), serum iron & total iron binding capacity (for calculation of transferrin saturation), CRP (C-reactive protein) and AGP (alpha-1-acid glycoprotein) will be determined using the fully automated biochemistry analyser (Cobas Integra 400 plus) at MRCG Keneba (the MRCG Quality team is currently working towards setting up the validation of the iron panel assays in Keneba).

Serum hepcidin will be measured in another aliquot (200 µl) using the DRG® Hepcidin 25 (bioactive) HS ELISA test kit and the Thermo Multiskan FC Microplate Photometer at MRCG Keneba.

Serum NTBI (aliquot of 300 µl) will be measured at King's College London (UK) by the new fluorescent beads method (44) that correlates closely with the traditional NTA method at lower TSAT but offers a crucial advantage over the NTA method because this can overestimate NTBI since NTA can remove some Fe bound to transferrin, particularly at TSAT above 70% (45, 46).

One serum aliquot (500 µl) and one plasma aliquot (500 µl) will be kept stored at -70°C for future analysis, particularly as new biomarkers of adverse effects due to iron emerge. This was a request from the funders. This may include DNA analysis and export of samples. We will obtain informed consent from the mothers/guardians for this to be the case within the study informed consent. Any future use would require PI, MRCG SCC and EC approval. This medium-long term storage will most likely be at MRCG Fajara.

In the ***finger prick blood samples***, Hb will be determined using a HemoCue 301 (5 µl) and an RDT (5 µl) will be performed every week at one of the study health facilities. In case of a positive RDT, a blood film (20 µl) will be prepared and read at the MRCG laboratory in Basse.

Stool samples:

Stool samples (~20 g) will be collected in toilet pots provided by the study team and lined with a disposable plastic liner at study days 1, 15 and 85 either at the participant's home on the morning of the study visit or at the health facilities where the venous blood sample will be collected. The samples will be kept in the toilet pots covered with the plastic liner in a cool place until the field worker process them. The field worker will aliquot the sample into the two labelled sterile stool sample tubes with scoop and screw lid as soon as possible after the child passes the stool. Approximately 15g of stool will be placed into a sterile Sarstedt stool collection tube and 5g of stool into an OMNIgene GUT sample collection kit tube containing a DNA preservative that ensures that samples for microbiome analysis can be kept at room temperature. The field workers will avoid soil contamination and cross-contamination of the samples at all times. The samples will then be transported to the MRCG Basse laboratory. Once samples arrive at the MRCG Basse, the Sarstedt sample will be further divided into three aliquots: one aliquot will be used for helminth egg count and will be kept refrigerated until processing (~ 5 g) and the remaining two aliquots (~5 g each) will be frozen at -70°C, within 24 hours of collection. The OMNIgene sample will be kept at room temperature in the lab at MRCG Basse until DNA extraction, which should be done within 8 weeks for all samples.

The national policy for anti-helminth treatment in The Gambia is that every child should receive de-worming tablets every 6 months, however, we still expect a proportion of the study population to be infected with soil-transmitted helminths (STH). Since STH infection can affect iron absorption and impact body iron status in young children (47, 48), we will perform a microscopic examination of a small sample of stool (sample kept refrigerated, not frozen) to determine the presence and number of STH eggs using the current WHO recommended Kato-Katz method, based on duplicate slides (49). Sample aliquots for this analysis will not be frozen but will be kept refrigerated at 4°C for up to 3 weeks as this has been shown to not cause significant changes in the egg counts and the morphology of eggs. Those children who test positive for STH infection (assessed on study days 1, 15 and 85) will be provided with anti-helminth treatment according to national guidelines but will not be excluded from the study.

The second Sarstedt sample aliquot (5 g) will be used for calprotectin analysis using the Calprotectin ELISA (EK-CAL, Bühlmann Laboratories). This analysis will be performed at the Basse Lab.

An additional aliquot (5 g) will remain frozen at -70°C for future analysis. This medium-long term storage will most likely be at MRCG Fajara.

The DNA will be extracted from the OMNIgene aliquot sample using the MoBio Soil extraction kit at the MRCG Basse laboratory. Faecal DNA will be kept frozen at -70°C until it is sent to the Sanger Institute in Cambridge (UK) for microbiome analysis at the end of the study. Microbiome analysis will be carried out by 16S rRNA sequencing (Illumina MiSeq). Minimum entropy decomposition (50) and targeted qPCR will be used to probe specifically the enteric pathogens. A study scientific officer will be trained in this analysis at the Sanger to help with the PCR lab work load.

Finally, for those children presenting with bloody diarrhoea episodes, an additional stool sample will be collected at that point and we will use stool microbiology, at the Basse laboratory, and the TaqMan Array Card (TAC) system (41) (same used in the GEMS study), at the Fajara laboratory, to identify the causative infectious agent.

6 Safety considerations

A trial steering committee (TSC) has been setup in discussion with the Sponsor. Membership of this committee includes the co-PI (Dr Pereira), the MRC BSU biostatistician advisor (Dr James Wason), an independent Chair (Dr Margaret Pinder, MRCG Basse), a sponsor representative (Mr Pa Cheboh, Head of Operations at MRCG Basse), a community representative (local teacher), and the local PI (Dr Mohammad Ilias Hossain, MRCG Basse).

This trial will be overseen by a Data Safety Monitoring Board (DSMB) (the chair of this committee is Professor James Jay Berkley of the KEMRI-Wellcome Kilifi Institute)). The DSMB will be responsible for reviewing:

- the trial protocol (before the trial is started)
- all interim data from the trial
- treatment safety and efficacy including the protection of the rights and well-being of the participants
- the overall progress of the study

The DSMB will additionally review all Serious Adverse Events (SAEs).

In addition to the DSMB, an independent Local Safety Monitor will regularly review all AEs and SAEs. This review will focus particularly on AEs causality and reasons for losses to follow up, raising any concerns or issues that present immediate safety concern with the PIs for reporting to the DSMB, while protecting the confidentiality of the trial data and the results of monitoring.

6.1 Methods and timing for assessing, recording, and analysing safety parameters

The trial will be conducted according to Good Clinical Practice (GCP) principles. The DSMB will determine how they will monitor the data and safety interest of the participants. The DSMB will also determine how and the frequency of its meetings but we propose that the DSMB meets at least 3 times during the study intervention period to review AE/SAE rates in each arm and to have a concluding meeting towards the end of the study period. SAEs will be sent in real-time to the DSMB. The DSMB's responsibilities will be clearly defined in the DSMB charter, which will include how real-time SAE submissions are to be handled, i.e. mechanisims that would trigger ad-hoc meetings, etc. The DSMB charter has been prepared by the co-PI with Dr Jonas Lexow and has been reviewed by the DSMB Chair.

6.1.1 Adverse events

An adverse event (AE) is defined as any untoward or unfavourable medical occurrence in a human subject, including signs and symptoms which are temporally associated with the subject's participation in the research, whether or not considered related to the subject's participation in the research. Participants will be monitored for AEs on each scheduled follow up day. All symptoms or signs reported or observed will be assessed by the study Field Assistant and will be recorded as an AE after evaluation by the study nurse. Persistently low Hb will be considered as an AE and will be followed up.

Diarrhoea, defined as 3 or more loose or watery stools per day will NOT be considered as an AE (but will be recorded as part of the morbidity questionnaires), unless it requires treatment. However, moderate-severe diarrhoea, referring to those diarrhoea episodes where (i) the child passes 5 or more loose or watery stools per day, (ii) there is blood in the stool (dysentery), OR (iii) the child shows signs of clinical dehydration (assessed by the study nurse based on physical signs such as little or no urination, sunken eyes, and skin that lacks its normal elasticity), will usually require treatment (including ORS) and will be recorded as an AE.

6.1.2 Reactogenicity

N/A

6.1.3 Serious adverse events (SAEs)

A SAE is any AE that is life-threatening or results in death or requires hospitalisation or prolongation of hospitalisation or is a persistent or significant disability/incapacity. All SAEs will be investigated by the study clinicians.

6.1.4 Assessment of intensity of AEs

The study clinicians, i.e. the research clinician and the local PI (paediatric clinician and Clinical Trial Coordinator) with support from the clinical team in Basse, will assess the severity and intensity of the AEs and laboratory changes as defined below and record it into the AE form.
The local safety monitor is also a paediatric clinician independent from the research team and her primary responsibility will be to review individual and cumulative AEs and report to the local PI (Clinical Trial Coordinator).

Grade		Description
1	Mild	Awareness of sign or symptom, but easily tolerated
2	Moderate	Enough discomfort to cause interference with usual activity
3	Severe	Incapacitating with inability to work or do usual activity
4	Life-threatening	This grade will be considered as SAE

The term "severe" is often used to describe the intensity (severity) of a specific event (as in mild, moderate, or severe myocardial infarction); the event itself, however, may be of relatively minor medical significance (such as severe headache). This is not the same as "serious", which is based on the outcome or criteria defined under the SAE definition. An event can be considered serious without being severe if it conforms to the seriousness criteria; similarly severe events that do not conform to the criteria are not necessarily serious. Seriousness (not severity) serves as a guide for defining regulatory reporting obligations.

6.1.5 Assessment of causality

Every effort will be made by the study clinicians (the research clinician and the local PI), in discussion with the clinical team in Basse, and the co-PI (Dr Pereira), to explain each AE and

assess its causal relationship to administration of the investigational product. This explanation will be based on the type of event, the relationship of the event to the time of trial intervention, and the natural history of the underlying diseases, concomitant therapy, etc. The results will be documented on the AE form. The relationship of an AE to the investigational product will be assessed according to the definitions in the MRCG Unit's SOP:

Unrelated

No temporal association with the study supplementation/drug; related to other aetiologies such as concomitant medications or conditions, or participant's known clinical state.

Unlikely

Temporal association with the study supplementation is improbable, but not impossible; other aetiologies such as concomitant medications or conditions, or participant's known clinical state provide plausible explanations.

Possible

Less clear temporal association; event could also be explained by alternate aetiology (clinical state, environmental or other interventions).

Probable

Clear-cut temporal association, with improvement upon drug withdrawal and not reasonably explained by alternate aetiologies (patient's known clinical state, environmental, or other interventions).

Definite

Clear-cut temporal association, with a positive re-challenge test or laboratory confirmation.

The mothers/guardians of participating children will be instructed to contact the field assistant or a member of the study team, should the child manifest any signs or symptoms they perceive as severe during the period extending from performance of the first trial procedure to the end of the study.

All findings observed or reported from the day of the first administration of the investigational product will be recorded on an AE Form electronically by the team. Whenever possible, AEs will be documented in terms of a diagnosis or syndrome rather than multiple symptoms that are clear manifestations of the same diagnosis/syndrome. In case signs and symptoms are reported by the participants, a medical diagnosis will be obtained by the study clinicians. If a diagnosis cannot be obtained then each sign or symptom will be recorded as separate events.

The action taken (e.g. discontinuation of investigational product, withdrawal of the participant from the trial, requirement of concomitant medication or treatment, others) will be recorded on the AE Form. If hospitalisation or its prolongation is required this will be reported as a SAE.
All AEs will be followed until resolution of the event and/or the end of the trial. The outcome will be assessed as follows:

- Resolved
- Resolved with sequelae
- Ongoing
- Death
- Lost to follow up

Treatment of any AE and SAE will be recorded in the appropriate section of the CRF.

6.1.6 Serious Adverse Reaction (SAR)

SAR is any SAE deemed to be **probably related or definitely related** to the study supplementation (as defined above).

Trial recruitment will be stopped with immediate effect if any child has a SAR to any of the iron supplements (or placebo) until review by the DSMB. Follow-up of children already enrolled in the study will continue as planned pending the review of the DSMB, as indicated in the DSMB Charter.

6.2 Reporting procedures

The Local Safety Monitor will prepare regular reports concerning AEs and SAEs for submission to the local PI. The local PI (Clinical Trial Coordinator), shall report all SAEs (defined as in 6.1.3) without filtration, whether or not related to the trial intervention, within 24 hours of becoming aware of the event to the DSMB, the Sponsor, and the MRCG CTSO (dct@mrc.gm). If the SAE is related to the trial intervention, the Ethics Committee will be notified within 7 calendar days if fatal or life-threatening, and all others within 15 calendar days.

All SAE will be reported to the Medicines Control Agency in accordance with the applicable requirement.

The minimum information required for this initial SAE report is:

- Trial number and (short) title
- Participant's ID
- Date and time of onset
- Description of the event (clinical history, associated signs and symptoms)
- Reporter's name

The information about which intervention was administered (unblinding) will only be provided at the request of the DSMB.

The local PI will not wait for additional information to fully document the event before notifying. The report is then to be followed by submission of a completed SAE Report Form as soon as possible, detailing relevant aspects of the SAE in question. All actions taken by the local PI and the outcome of the event must also be reported immediately.

For documentation of the SAE, any actions taken, outcome and follow-up, the SAE Report Forms will be used. All follow-up activities have to be reported, if necessary on one or more consecutive SAE report forms in a timely manner. All fields with additional or changed information must be completed and the report form should be forwarded to the DSMB within 5 calendar days after receipt of new information. Hospital case records and autopsy reports, including verbal autopsy, will be obtained where applicable.

6.3 Safety oversight

Safety oversight shall be provided by a DSMB who will provide independent advice as stipulated in Section 6 above. An independent Local Safety Monitor will review all adverse events more regularly and report regularly to the PIs and DSMB.

7 Discontinuation criteria

7.1 Participant's premature termination

Mothers/guardians of participating children have the right to stop their children's participation in the study at any time without giving a reason and this will not affect the medical care that would normally be received. The trial team may also withdraw a participant from the study if deemed necessary at any time taking in to consideration the reasons mentioned below. The reason for a participant's premature termination will be documented on the appropriate page of the CRF and specified which of the following possible reasons were responsible for the premature termination:

- Serious Adverse Event
- Adverse Event
- Participant's consent withdrawal
- Development of withdrawal criterion
- Protocol deviation
- Migrated/moved from the study area
- Lost to follow-up

A 'lost to follow-up' is any participant who completed all protocol specific procedures up to the administration of the investigational product or intervention, but was then lost during the study period to any further follow-up, with no safety information and no efficacy endpoint data ever became available.

In case the participant decides to withdraw participation or consent during the study, we will not work on participant's samples without permission, but any information already generated from the samples will be kept and used. The study clinician may also ask for tests for the participant's safety. The PI will inquire about the reason for any withdrawal and follow-up with the participant regarding any unresolved AEs.

For withdrawn participants no specific data will be collected. Our sample size takes into account a dropout rate of 15% but this will be monitored closely and if the observed dropout rate increases above this level we will replace subjects to ensure we reach our target sample size of n=600 completed.

7.2 Study discontinuation

The rules for study termination will be set by the DSMB at their first meeting.

8 Statistical considerations

8.1 Sample size determination

This section was prepared with the expert input of Dr James Wason, MRC Biostatistics Unit, Cambridge. Dr James Wason is a Programme Leader Track senior statistician who primarily works on statistical methodology for novel clinical trial designs and is one of the few UK experts in design and analysis of adaptive clinical trials. Dr Wason is a long term collaborator of Dr Pereira for the design of the IHAT trials and is a collaborator in the proposed trial. He has provided advice on the trial design and conducted the trial power modelling and sample size calculations presented in this Section. Dr Wason will continue to provide advice on the trial design to the PIs and will supervise an MRCG statistician in the data analysis corresponding to the four trial hypothesis (below). Dr Wason will be a member of the TSC and will review and approve the data analysis plan for the trial, but it will be the responsibility of the MRCG statistician (Dr Nuredin Mohammed supervised by Dr David Jeffries) to develop the initial drafts of the data analysis plan together with the study PIs.

There are four null hypotheses in the study:

1) non-inferiority of IHAT compared to ferrous sulphate for efficacy (in terms of Hb and iron deficiency correction: i.e. IDA) response probability (or prevalence). In this case we are testing the null hypothesis that: response in the IHAT arm minus the response probability in the ferrous sulphate arm is less than or equal to – 0.1. We define 'response' for IDA as correction of iron deficiency and either achieving a normal Hb or an increase of at least 1 g/dL after 12 weeks of iron supplementation.

2) superiority of IHAT compared to ferrous sulphate in terms of incidence density of diarrhoea. Here the null hypothesis is that the mean number of new episodes in the IHAT arm is greater than or equal to the mean number in the $FeSO_4$ arm.

3) superiority of IHAT compared to ferrous sulphate in terms of prevalence of diarrhoea. Here the null hypothesis is that the prevalence of diarrhoea in the IHAT arm is greater than or equal to the prevalence in the $FeSO_4$ arm.

4) non-inferiority of IHAT compared to placebo in terms of prevalence of diarrhoea. Here the null hypothesis is that the prevalence of diarrhoea in the placebo arm minus the prevalence in the IHAT arm is less than or equal – 0.1.

Because this is a pilot study we do not formally adjust for multiple testing due to the four hypotheses. This is because any significant results will be tested again in a pivotal study.

The trial is powered to show non-inferiority of IHAT relative to ferrous sulphate for the 12 weeks efficacy response probability. At the 12 weeks time-point (i.e. Day 85), the estimated odds ratio between the IHAT and $FeSO_4$ arms will be calculated, together with a 90% one-sided confidence interval. If the lower limit of the confidence interval for this difference is above 0.583 (equivalent to a 10% absolute difference if $FeSO_4$ response rate is 0.3), then non-inferiority of IHAT in terms of efficacy will be declared.

Because many trials conducted in Africa and other developing countries with the gold standard ferrous sulphate or ferrous fumarate supplementation still fail to provide anaemia resolution in interventions shorter than 6 months, we have considered here that an increase of 1 g/dl in Hb after 3 months is an indication of efficacy, even though we accept that this is not clinical efficacy. We have based our assay sensitivity from data published with relevant iron supplement studies in children available in the literature (3, 7, 52). We plan, however, to use the data collected in the proposed study to conduct an exploratory analysis to test for assay sensitivity. These data will be very useful so that adequate treatment effect sizes can be used to power any future studies, in particular a pivotal trial, both for ferrous sulphate and IHAT supplementation effects.

Due to this trial being a pilot study, any significant results will be re-tested in the pivotal 'full' trial (BMGF full award). Thus, we considered a larger than traditional type-I error rate of 10%. Assuming that 30% (i.e. a response probability of 0.3) of the children in both arms will resolve iron deficiency and either achieve a normal Hb or an increase of at least 1 g/dL after 12 weeks of iron supplementation (3, 7, 52), then a sample size of 200 in the IHAT arm and 200 in the ferrous sulphate arm will provide 89% power to show non-inferiority with a non-inferiority margin of 0.1 (0.583 on the odds-ratio scale). In other words, the trial will have 89% power (at a 10% one-sided significance level) to show that the lower boundary of the confidence interval of the odds ratio between IHAT and ferrous sulphate is more than 0.583 at 12 weeks.

We have based our calculation above on assay sensitivity data taken from other studies with iron supplements reported in the literature, but we will also perform an exploratory analysis to test for assay sensitivity, and this information will be used to power the potential future pivotal study.

We have considered two options for modelling the diarrhoea outcome: 1) 'period prevalence', i.e. the proportion of children with at least one episode of moderate-severe diarrhoea over the 12 weeks intervention period; 2) 'incidence density'. i.e. the number of new diarrhoea episodes per child over the 12 weeks intervention period. The modelling of how the power of the trial varies according to assumptions for each of these outcomes is detailed below.

The trial sample size will also provide 90% power to show superiority of IHAT in terms of moderate-severe diarrhoea 'incidence density', assuming that IHAT provides a 20% reduction in mean moderate-severe diarrhoea 'incidence density' relative to ferrous sulphate; and 90% power to show superiority of IHAT in terms of 'period prevalence' of moderate-severe diarrhoea, assuming that 25% of children will develop moderate-severe diarrhoea episodes with ferrous

sulphate and 15% will have moderate-severe diarrhoea with IHAT over the 12 weeks intervention. For the comparison between the IHAT and placebo arms, the trial is powered to show non-inferiority of IHAT relative to placebo, for example, the trial will have 93% power for the moderate-severe diarrhoea 'period prevalence' outcome. All the assumptions for these calculations were based on data from studies conducted in The Gambia for the same age group, please refer to the extra information below for further details.

As part of the rationale for the study is to provide information for a pivotal study, we also examined the precision that the sample size would provide on the confidence interval width of the outcomes considered. Since the width of the confidence interval is highly dependent on the proportion in the control group as well as the true odds ratio, we consider several different values in the table below.

Binomial proportion in control group	True odds-ratio	95% confidence interval width
0.1	0.5	0.85
	1	1.38
	1.5	1.91
0.3	0.5	0.49
	1	0.88
	1.5	1.27
0.5	0.5	0.42
	1	0.80
	1.5	1.21

The power calculations for some of the secondary outcomes are also presented in more detail below. Briefly, the trial (n=200 per arm) will have over 85% power to detect significant differences between all the arms in terms of Enterobacteria, NTBI and calprotectin.

Taking into consideration a non-completion rate of 15% (this is based on data from previous studies in The Gambia), we expect to **enrol 705 children in the study**.

Detailed calculations of power for the diarrhoea and secondary outcomes:

I. POWER OF THE TRIAL FOR THE DIARRHEAL OUTCOME.

<u>1. Period Prevalence (i.e. proportion of children with >= 1 episode of moderate-severe diarrhoea over the 12 weeks intervention period)</u>

Based on previous data from the case-control GEMS (Global Enteric Multicentre Study) for the Basse (Upper River BHDSS) region in the Gambia, the background point prevalence of moderate-severe diarrhoea in children under 3 years of age can be as high as 14%, whereas data from the same region in a previous survey HUAS (Health Care Utilization and Attitudes Survey) showed that this number can be as high as 20%.

For our calculations, we chose to consider 15% as the proportion of children with at least one episode of diarrhoea in the IHAT group over the 12 wk. intervention period, which we hypothesise would be similar to the background period prevalence. This prevalence is similar to that found in other iron intervention studies for the no-iron groups (7, 8).

The graph below shows the power of the trial (200 patients per arm at a 10% one-sided type I error rate) to detect a significant difference between IHAT and ferrous sulphate (FS) as the ferrous sulphate diarrhoea period-prevalence varies:

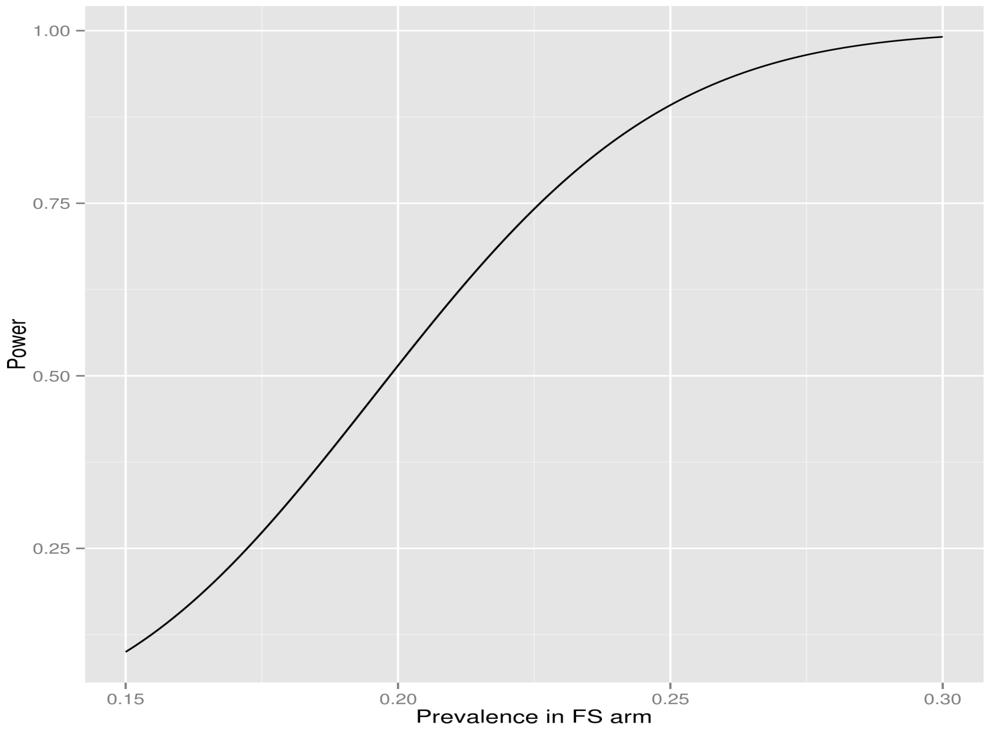

For a period prevalence of 0.15 in the IHAT arm and 0.25 in the ferrous sulphate arm, the power of the trial is around 90%. We note that previous data for ferrous sulphate suggest that 25% is a reasonable figure for the period prevalence: for example Zlotkin et al 2013 reported this to be 16.8% in the iron group and Jaeggi et al 2014 reported 27.3%. Furthermore, for the Gambia specifically, preliminary data from the 84 days period of the current HIGH study in the Soma region shows that, during the 84-days of the intervention, moderate-severe diarrhoea was recorded at least once as an adverse event in 36% of the children.

For comparing placebo and IHAT, we used a non-inferiority approach. With 200 patients per arm, we would have 93% power to show non-inferiority of IHAT with a 10% non-inferiority margin (at a 10% one-sided type I error rate). This means that, if the true diarrhoea period prevalence in the IHAT and placebo arms is 0.15, this sample size has 93% power to show that the upper confidence interval of the difference in prevalence between these two arms is less than 10%.

2. Incidence density (i.e. number of moderate-severe diarrhoea episodes per child over the 12 weeks intervention)

Preliminary data from the 84 days data collected in the HIGH study in the Soma region in the Gambia suggest that the incidence density of moderate-severe diarrhoea is 1.28 episodes per child over the 84-days study period. Using the same type I error rate and sample size as above, the graph below shows the power of the trial to detect a significant difference between arms as the rate ratio varies.

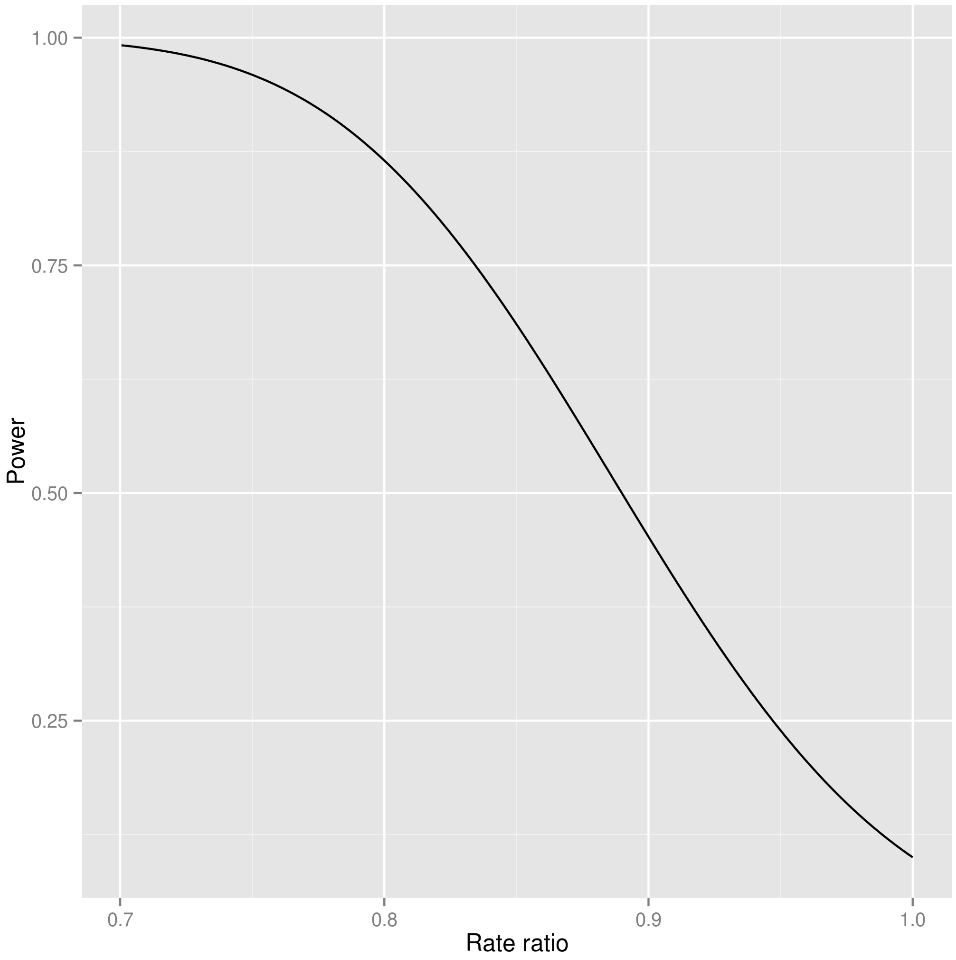

For a rate ratio of 0.8 (i.e. IHAT provides a 20% reduction in mean incidence density of moderate-severe diarrhoea relative to ferrous sulphate), the power is almost 90%.

II. POWER of the trial for the secondary endpoints

1) Microbiome (Enterobacteria log10 counts):

We based our calculation on the data for Enterobacteria (the genus that contains most enteropathogens including *E. coli*) reported in Zimmermann 2014 study conducted with 4 months supplementation (8): with iron 8.9 (0.3) and without iron 8.0 (0.4). Taking into consideration that in this study the analysis combined the groups with 2.5 mg Fe and 12.5 mg Fe MNP, then the combined sample size implies an individual standard deviation of 3 for the Enterobacteria and assuming a 0.9 difference between the groups and a superiority margin of 0.2 (i.e. an absolute difference in Enterobacteria between the IHAT and ferrous sulphate arms of - 0.2), the sample size of 200 children per group would give us 85% (at 10% one-sided significance level).

2) Calprotectin

Again, we based this calculation in the Zimmermann 2014 study (8). Calprotectin concentration with iron: 248.9 +/- 2.2 μg/g and without iron: 102.5 +/- 2.2 μg/g in the 12.5 mg MNP. Here the individual SD should be around 11.8 considering the study sample size in the 12.5mg Fe MNP group, which would mean that with 200 per arm we could detect an absolute mean difference of 3 with 90% power (10% one-sided sig level) and with 50 per arm we would be able to detect a mean difference of 6.1.

3) NTBI

For this calculation we have used the 'normal serum' data provided by Robert Hider with the same method we intend to use in the study (44). Taking the values reported in the paper for 'normal serum' of 0.24 +/- 0.15 (mean +/- SD), n=9 range 0.04 – 0.41, the individual standard deviation would be 0.45. There is no published data with this method for NTBI after an iron dose so we have assumed we would like to detect a mean difference of 0.5 between the IHAT and the ferrous sulphate arm, which would mean that with 50 per arm the power would be more than 90%. To be able to detect smaller differences with this method we would need to increase sample size.

8.2 Statistical analysis

The data analysis plan will be fully developed prior to commencing trial recruitment and will be done with the expert input from Dr James Wason of the MRC Biostatistics Unit in Cambridge. Briefly, for the primary analysis, we will fit a logistic regression to the efficacy outcome data, adjusting for the strata created by age and Hb level groups prior to enrolment. The odds ratio for effect of IHAT relative to ferrous sulphate will be estimated, and if the lower 90% one-sided confidence interval is above 0.583 (equivalent to the 10% non-inferiority margin above), non-inferiority will be declared for IHAT. The diarrhoea endpoint will be analysed in a similar way, but tested for superiority (i.e. if the one-sided p-value for the Wald test of the effect of IHAT is less than 0.1) for the comparison between IHAT and ferrous sulphate. For the primary non-inferiority hypothesis we will conduct per-protocol analyses and for the superiority hypotheses we will conduct intention-to-treat analyses.

The secondary endpoints (calprotectin, NTBI etc) will be tested using linear regression with the same covariates. Morbidity data will be analysed using multiple regression analysis controlling for possible confounders.

In relation to the microbiome data analysis we will work together with our collaborators in the Sanger Institute and use the most up to date bioinformatics methodology available at the time of data analysis to investigate the impact of treatment arm and time in the composition of the gut microbiome. Since this analysis will only occur in late 2017, it is premature to decide the strategy for data analysis now as this is a fast moving field with new and better bioinformatics tools becoming available every couple of months. The Sanger Institute works very closely with the European Bioinformatics Institute (EBI) (housed in the same campus) to ensure they use the very latest tools in their microbiome analysis.

9 Data handling and record keeping

9.1 Data management and processing

All protocol data will be captured in Case Report Forms (CRF) that will be completed for each included participant using electronic data capture. On the CRF, a reference to the source

document will be provided. Instructions for completing all forms, including the CRF, used in the study will be developed.

The following data will be recorded: date of informed consent, personal data (ID, initials, date of birth), socioeconomic data, height and weight, information on health status and regarding participation in other studies, date and time of all venous and finger prick blood collections, date and time of all faecal sample collections, date and time of supplement administration, data on morbidity, lab results.

All trial data will be stored and managed within a clinical database built on the OpenClinica Enterprise platform, an application specifically designed to collect and store clinical trial data and customised for Electronic Data Capture (EDC) in the field. Where appropriate, data entry fields will incorporate appropriate range checks. At the point of data collection, data will be entered directly into tablet computers as already done in the MRCG studies, and uploaded to the main server either immediately, or at least daily when network signal is weak. Data export options from the clinical database include CDISC ODM XML, a vendor neutral, platform independent format for interchange and archive of data collected in clinical trials. This should ensure sharing and long term validity of data. This data management system is a fully supported, externally validated (GCP compliant to 21 CFR part 11) clinical database.

Prior to the first participant enrolment a detailed trial specific data management plan (t_DMP) will be created, reviewed and signed off by the Data Manager and the TSC. This t-DMP will outline in detail the specific procedures that will be used to ensure high quality data will be delivered for statistical analysis and future use. The t-DMP will complement the trial risk assessment and statistical analysis plan. Review of data collection tools against the protocol will occur prior to database build by the Data Manager to ensure all data is being captured, at the correct time points, and in a format conducive to the proposed statistical analysis. Site based staff will be fully trained in conducting any study assessments and subsequent completion of the CRF and a Site delegation log will be maintained. Data entry staff will be trained in the use of OpenClinica. Principles of good clinical practice will be adhered to and all training will be documented. To ensure standardisation of processes, standard operating procedures with respect to trial management, quality assurance, data management, IT & security, and statistics will be adhered to.

9.2 Source documents and access to source data

The local PI will maintain appropriate medical and research records for this study in compliance with the principles of good clinical practice and regulatory and institutional requirements for the protection of confidentiality of participants. The study team members will have access to records.

The authorised representatives of the sponsor, the ethics committee(s) or regulatory bodies may inspect all documents and records required to be maintained by the investigator, including but not limited to, medical records (office, clinic, or hospital) for the participants in this study. The clinical study site will permit access to such records.

9.3 Protocol deviations

A protocol deviation (PD) is any noncompliance with the clinical trial protocol, good clinical practice (GCP), or other applicable regulatory requirements. The noncompliance may be either on the part of the participant or the investigator including the study team members, and may result in significant added risk to the study participant. As a result of a deviation, corrective actions will be developed and implemented promptly.

If a deviation from, or a change of, the protocol is implemented to eliminate an immediate hazard(s) to trial participant without prior ethics approval, the PI or designee will submit the implemented deviation or change, the reasons for it, and, if appropriate, the proposed protocol amendment(s) as soon as possible to the sponsor for agreement and the relevant independent ethics committee (IEC) for review and approval.

The local PI or designee will document and explain any deviation from the approved protocol on the CRF, where appropriate, and record and explain any deviation according to the Unit's SOP.

10 QUALITY CONTROL AND QUALITY ASSURANCE

Quality control will be applied to each stage of the study. We will work together with the Sponsor, through their Quality Management team (Mrs *Yai Louise Ndure-Bensouda*) and Research Governance teams (led by Dr Jonas Lexow) to finalise a plan for the trial quality monitoring.

It will be the responsibility of the local PI or designated trial team member to ensure that all source documents and CRFs are reviewed for accuracy and completeness. Any correction will be accurately accounted for.

Finger prick and venous blood collection will be done by the trial nurses. Collection of all samples will be recorded on the CRF.

All Field assistants and their supervisors including trial nurses will be fully trained. Selected participants from the RCH teams will also be trained. Further refresher training will be conducted as the trial progresses. Weekly field team meetings and monthly meetings of the entire trial team will be convened in order to discuss all problems and lessons from the trial.

We will also work with the Quality and Laboratory Management teams to write a detailed Analytical Plan for the trial and appoint a study team member (likely one SO) as the Analytical Project Manager who will be responsible for overseeing and ensuring the quality of the laboratory activities for the trial.

10.1 Study monitoring

A risk-based monitoring plan will developed with the MRCG Clinical Trial Support Office prior to study starting. This will describe monitoring frequencies and content of Site Initiation, Interim Montoring and Close-out Visits.

11 Ethical considerations

This study is conducted in accordance with the principles set forth in the ICH Harmonised Tripartite Guideline for Good Clinical Practice and the Declaration of Helsinki in its current version (see appendix), whichever affords the greater protection to the participants.

11.1 General considerations on human subject protection

Study participants will be young children and the study protocol will be explained to their mothers/guardians orally in the presence of an independent witness in case they are illiterate or in writing. No children will start any study specific procedure before informed consent is obtained. The study investigates iron supplementation in doses specifically recommended for this age group by the WHO. Participants will not get any remuneration but will have free basic medical care for the duration of the study.

11.1.1 Rationale for participant selection

Young children, together with pregnant women, are the two most affected population groups in relation to anaemia and iron deficiency anaemia, and the groups for which better iron supplementation strategies are required.

In pregnant women the main problems of current iron supplements are the gastrointestinal side-effects such as nausea, abdominal cramps, heartburn and constipation (15), which affect compliance with treatment. Our future aim is to be able to also conduct a trial in pregnancy to test the hypothesis that IHAT would not be associated with these side-effects in the pregnant women. We are actively seeking funding for this.

However, in our opinion the most pressing need in relation to iron supplementation in developing countries is to find an alternative iron supplement for use in young children living in areas at risk of enteric infection. As mentioned in the Background Section, RCTs with current iron supplementation in nearly ten thousand young children living in developing countries have consistently shown that these are associated with increased infection including bloody diarrhoea (3, 6-8) and detrimental changes to the gut microbiome and gut inflammation (8, 9), further increasing the burden from enteric infection and environmental enteropathy (i.e. persistent gut damage and inflammation that leads to malabsorption), which is a major cause of growth failure in children in resource-poor environments (10, 11). Furthermore, it appears that these effects are much more relevant in those resource-poor countries where enteric infection risk is higher and in the pre-school age group since in South-African 6-11 years old children with a low enteropathogen burden, iron supplementation did not significantly affect the dominant bacterial groups in the gut or gut inflammation (53). The specific problem this proposal seeks to address relates to these effects of oral iron supplements for treating anaemia in young children. Hence, why we propose to conduct the trial in young children living in the most deprived areas of The Gambia where the risk for enteric infection is higher.

More than 70% of all children under 5 y. in The Gambia will be anaemic and in most cases this will be due to iron deficiency. The consequences of anaemia and iron deficiency include impaired neurocognitive development and immunocompetence leading to substantial loss of human potential.
Combating anaemia due to iron deficiency is a challenge due to the potential negative side-effects of iron when given to people with infections, as is extremely common in young children in The Gambia.

Specifically, we will be addressing the fact that current iron supplements increase risk of moderate-severe diarrhoea in these children, particularly in settings where risk for enteric infection is high, as we mentioned above. In the 21st century, we simply cannot accept the

current view that increased risk of infectious diarrhoea in young children is normal collateral damage of oral iron supplementation in populations at high risk of enteric infection. This is a problem of current iron supplements and we believe that IHAT will work differently for all the reasons explained in the Rationale Section of this protocol. IHAT may also offer other advantages in relation to conventional iron supplements in relation to efficiency of absorption of consecutive daily doses and systemic infection risk as explained in the Rationale Section of this protocol.

Finally, we wish to note that we believe that IHAT's main target population group, where IHAT's benefit will really outweight its added cost over current iron supplements, are young children from resource-poor countries. This is because the main advantage of IHAT over other iron supplements relates to its intestinal safety and, as we mentioned above, young children living in areas of enteric infection risk are the main population group affected by the negative effect of current iron supplements on intestinal infection (including bloody diarrhoea) and inflammation (contributing to environmental enteropathy). Essentially, if IHAT works in the proposed trial, where it will be tested in the population group that is more responsive to the adverse effects of iron supplementation (i.e. young children at risk of enteric infection), then it will work in any other population group. But the reverse is not true, i.e. if IHAT works in adults it does not mean it would work in children, and similarly if IHAT works in older children it does not mean it would work in the young children group, since these are the subjects with the most immature gut where the microbiome is not stable and where the mucosa is more susceptible.

This is why it is crucial to conduct the trial in the young children living in some of the most deprived and infectious communities in the Gambia. Conducting the study in adults would not provide us with clinically-meaningful information about the impact of IHAT on enteric infection and diarrhoea risk, since this effect is not commonly observed in adults (possibly due to the immature gut microbiome and mucosa in young children) with iron supplementation, and would just delay further investment and the authorisation of IHAT for use in children, which would be unethical in our view. As such, we follow the recommendation of the European Medicines Agency to study medicines intended to be used in children in a paediatric population rather than inferring the information and dosages from adult trials (Regulation (EC) No 1901/2006). We appreciate that children are a more vulnerable group for the first Phase II trial of IHAT or indeed any other form of iron supplementation, but they are also the group that will most benefit from a better iron supplement. The health of all children in our study will be closely monitored as will all adverse events including all diarrhoea episodes, malaria and other co-infections. Therefore, ethically we will not be subjecting children to any unnecessary risk or, in fact, any more risk that when current iron supplements are used as per national guidelines. In the worst case scenario, IHAT will be the same as currently used ferrous suphate or fumarate in terms of diarrhoea and other adverse events.

This study will provide the first Phase II trial data with IHAT and will enable us to obtain high-quality clinical data for its safety and efficacy in correcting IDA in children, the population group most in need of an alternative oral iron supplement. If IHAT is successful in the trial, these data will provide the evidence needed to encourage further investment so that IHAT can be implemented as a novel iron source for use in micronutrient intervention strategies aimed at children and women living in resource-poor countries and, hence, reduce the global burden of IDA.

11.1.2 Rationale for use of a placebo group

We have thought very carefully about the lower threshold of Hb inclusion for all study arms and we wish to note that we are not contravening the current national guidelines, since there is no mandatory iron supplementation for this age group in the Gambia. All our prior iron intervention trials in The Gambia, Kenya and Tanzania, have used the Hb 7-11 g/dl inclusion criteria. The rationale being that Hb below 7 g/dl is the current WHO cut-off for severe anaemia in children under 59 months of age (WHO/NMH/NHD/MNM/11.1), and these children would be the ones presenting with more visible symptoms of anaemia and most likely to be automatically given oral iron treatment.

Since recent trials have demonstrated a possible detrimental effect of iron supplements in pre-school age children, there is now a strong ethical rationale for a 'no-iron' control group (i.e. placebo). It is crucial to have this placebo control group in our study, not only for determining background 'side-effects' but also, and importantly, for assessing the true treatment effect size of current iron supplementation (i.e. ferrous sulphate) in Hb levels. There have been numerous iron supplementation studies in young children in resource-poor countries, including in the Gambia, that have failed to show any decrease in anaemia prevalence with iron supplementation, and we feel it is necessary that we determine in this study if iron supplements are preventing Hb from falling further in relation to a 'no iron' (or placebo) group, which we consider to be an important positive effect, even when anaemia prevalence may not decrease for this age group due to the high demands for iron during fast growth.
It is also important to have a placebo group in this study so that we can perform an exploratory analysis and test for assay sensitivity and to determine treatment effect size with IHAT so that these data can be used to power future studies.

In the proposed study we will include only children with mild-moderate anaemia, and we will exclude any children with severe anaemia, who would be most likely identified and referred for iron treatment according to the national policy in The Gambia. The children with Hb between 7 and 11 g/dl would probably not be identified as anaemic had they not been offered screening through our study and would not have been provided with oral iron supplements. Within this

group, the children with Hb between 7 and 8 g/dl are the ones most in need of an alternative oral iron treatment, one that is both effective and safe. In any case, as we mentioned above, we will monitor very closely the Hb levels in all study arms and exclude and treat according to the national guidelines any children where Hb falls below 7 g/dl at any point during the study. Furthermore, at the end of the study, any child who remains anaemic will be offered the standard iron supplementation according to national policy. This means that we will only be delaying treatment for the children in the placebo group by 4 months (unless they become severely anaemic during the study, in which case they receive treatment immediately).

11.1.3 Evaluation of risks and benefits

There are risks associated with a large intake of iron supplements especially in areas of malaria endemicity. The dose of iron given daily in the reference arm (12.5 mg) is according to WHO guidelines for the age group children in non-malarious areas or malaria-endemic areas where it should be implemented in conjunction with measures to prevent, diagnose and treat malaria and co-infections. The iron dose in the IHAT arm (20 mg) is the bioequivalent dose, i.e. the same absolute amount of iron should be absorbed as in the ferrous sulphate arm, and because IHAT should be safer to the gut, the unabsorbed fraction should not cause detrimental effects, such as infectious diarrhoea. This dose (20 mg Fe) is still less than the new WHO recommendation for children in our 24-35 mo. age group (i.e. 30 mg, (40)). In any case, in all arms of the trial these adverse events will be closely monitored.

Additionally, we have put in place the following strategies to mitigate the risk of possible interactions between iron supplements and malaria or other co-infections:

(1) data from the Gambia over the last 5 years (medical records from the Kiang West region) shows that the peak malaria months are Oct and Nov and, therefore, we have timed the intervention period to avoid these months, (2) trained field workers will be visiting all children every day during the 12 weeks supplementation period in order to supervise the administration of the iron supplements or placebo and on these occasions they will check on the children's health status and actively look for signs of malaria and co-infections, if a child shows signs of these infections the study nurse will perform adequate tests and the child will be offered the appropriate treatment/referral to the next Health Centre. In case of a fever, a malaria rapid test will be performed and if positive the child will be treated according to national guidelines. A sick child will always be visited by a study nurse for further clinical investigations and if needed referred to the nearest Health Centre. These visits will carry on for 4 weeks after the end of the study intervention and, during both the intervention and this follow-up period, morbidity data will be

captured every other day. This is similar to what was done in the HIGH study and we do not anticipate any difficulties of implementation. Every week, the child will visit one of the study health facilities for a haemoglobin check-up and malaria RDT testing, and this will ensure that children that develop asymptomatic malaria are identified and treated according to national guidelines.

Participants will experience some transient pain during blood sampling, which will be minimised by recruiting well-trained nurses for the study.

Children will benefit from daily monitoring of their health and from immediate care in case of illness. Although the mechanism of combating iron deficiency is problematic, it is clear that iron is a key micronutrient for the development of the immune system and cognitive function in these young children.

11.2 Informed consent

All field workers taking part in the recruitment of participants will be trained on translating the contents of the information sheet and the details of it will be explained to illiterate mothers in a language they understand in the presence of an independent literate witness. The literate mothers/parental guardian will be allowed to read the information sheet in their own time. They will be given enough time to ask questions and decide if they want their child to participate. Informed consent will be recorded by a signature or thumbprint on the consent form. The consent form together with the trial protocol and information sheet is attached to this submission.

11.3 Participant confidentiality

Each participant will be allocated an individual identification (ID) number and these will be used to label all samples collected for the study and on the CRF during the course of the study. All data will be linked-anonymised and the linkage to the ID will not be possible without a lookup table, which will be held only by the data manager and designated data staff during the course of the study. Once data collection is complete, analysis will be performed on an anonymised copy of the data. At all stages, staff/collaborators responsible for sample analysis will be blinded as to the subject's identification. Together, these processes will ensure complete confidentiality of the data gathered and impartiality of data analysis.

11.4 Future use of stored specimen

Some of the samples will be transferred to Kings College London (serum) and the Wellcome Trust Sanger Institute (faecal DNA) for analysis of outcomes not available in the Gambia.

Aliquots of blood and stool samples will be kept frozen at -70°C for future analysis. This may include DNA analysis and export of samples. This was a request from the funders. We will obtain informed consent from the mothers/guardians for this to be the case within the study informed consent. Any future use would require PI, MRCG SCC and EC approval.

12 Financing and insurance

The research related costs of the proposed trial will be paid by a grant from the Bill & Melinda Gates Foundation.

The London School of Hygiene and Tropical Medicine (LSHTM) will sponsor this research and as such research participants will be protected in accordance with the LSHTM Clinical Trial/Non Negligent Harm Insurance and Medical Malpractice Insurance.

13 Publication policy

Our planned dissemination avenues include: at least three publications in high impact peer-reviewed open-access scientific journals with a wide readership (e.g. Lancet, JAMA, NEJM), presentation at international conferences (e.g. the Micronutrient Forum) and dissemination of the trial findings to organizations such as the WHO, UNICEF, UN World Food Programme and to the National Nutrition Agency and the Ministry of Health in The Gambia.

14 References

1. WHO. The global burden of disease: 2004 update. Geneva: WHO, 2008.
2. Sazawal S, Black RE, Ramsan M, Chwaya HM, Stoltzfus RJ, Dutta A, et al. Effects of routine prophylactic supplementation with iron and folic acid on admission to hospital and mortality in

preschool children in a high malaria transmission setting: community-based, randomised, placebo-controlled trial. Lancet. 2006;367(9505):133-43.
3. Soofi S, Cousens S, Iqbal SP, Akhund T, Khan J, Ahmed I, et al. Effect of provision of daily zinc and iron with several micronutrients on growth and morbidity among young children in Pakistan: a cluster-randomised trial. Lancet. 2013;382(9886):29-40.
4. Prentice AM, Verhoef H, Cerami C. Iron fortification and malaria risk in children. Jama. 2013;310(9):914-5.
5. Prentice AM. Iron metabolism, malaria, and other infections: what is all the fuss about? J Nutr. 2008;138(12):2537-41.
6. Mayo-Wilson E, Imdad A, Junior J, Dean S, Bhutta ZA. Preventive zinc supplementation for children, and the effect of additional iron: a systematic review and meta-analysis. BMJ Open. 2014;4(6):e004647.
7. Zlotkin S, Newton S, Aimone AM, et al. Effect of iron fortification on malaria incidence in infants and young children in ghana: A randomized trial. Jama. 2013;310(9):938-47.
8. Jaeggi T, Kortman GA, Moretti D, Chassard C, Holding P, Dostal A, et al. Iron fortification adversely affects the gut microbiome, increases pathogen abundance and induces intestinal inflammation in Kenyan infants. Gut. 2014.
9. Zimmermann MB, Chassard C, Rohner F, N'Goran E K, Nindjin C, Dostal A, et al. The effects of iron fortification on the gut microbiota in African children: a randomized controlled trial in Cote d'Ivoire. Am J Clin Nutr. 2010;92(6):1406-15.
10. Naylor C, Lu M, Haque R, Mondal D, Buonomo E, Nayak U, et al. Environmental Enteropathy, Oral Vaccine Failure and Growth Faltering in Infants in Bangladesh. EBioMedicine. 2015;2(11):1759-66.
11. Lin A, Arnold BF, Afreen S, Goto R, Huda TM, Haque R, et al. Household environmental conditions are associated with enteropathy and impaired growth in rural Bangladesh. Am J Trop Med Hyg. 2013;89(1):130-7.
12. Werner T, Wagner SJ, Martinez I, Walter J, Chang JS, Clavel T, et al. Depletion of luminal iron alters the gut microbiota and prevents Crohn's disease-like ileitis. Gut. 2011;60(3):325-33.
13. Dogan B, Suzuki H, Herlekar D, Sartor RB, Campbell BJ, Roberts CL, et al. Inflammation-associated Adherent-invasive Escherichia coli Are Enriched in Pathways for Use of Propanediol and Iron and M-cell Translocation. Inflamm Bowel Dis. 2014;20(11):1919-32.
14. Prentice AM, Doherty CP, Abrams SA, Cox SE, Atkinson SH, Verhoef H, et al. Hepcidin is the major predictor of erythrocyte iron incorporation in anemic African children. Blood. 2012;119(8):1922-8.
15. Tolkien Z, Stecher L, Mander AP, Pereira DI, Powell JJ. Ferrous sulfate supplementation causes significant gastrointestinal side-effects in adults: a systematic review and meta-analysis. PLoS One. 2015;10(2):e0117383.
16. Radulescu S, Brookes MJ, Salgueiro P, Ridgway RA, McGhee E, Anderson K, et al. Luminal iron levels govern intestinal tumorigenesis after apc loss in vivo. Cell Rep. 2012;2(2):270-82.

17. Seril DN, Liao J, Ho KL, Warsi A, Yang CS, Yang GY. Dietary iron supplementation enhances DSS-induced colitis and associated colorectal carcinoma development in mice. Dig Dis Sci. 2002;47(6):1266-78.
18. Seril DN, Liao J, Yang CS, Yang GY. Systemic iron supplementation replenishes iron stores without enhancing colon carcinogenesis in murine models of ulcerative colitis: comparison with iron-enriched diet. Dig Dis Sci. 2005;50(4):696-707.
19. Loh YH, Jakszyn P, Luben RN, Mulligan AA, Mitrou PN, Khaw KT. N-nitroso compounds and cancer incidence: the European Prospective Investigation into Cancer and Nutrition (EPIC)-Norfolk Study. American Journal of Clinical Nutrition. 2011;93(5):1053-61.
20. Lunn JC, Kuhnle G, Mai V, Frankenfeld C, Shuker DE, Glen RC, et al. The effect of haem in red and processed meat on the endogenous formation of N-nitroso compounds in the upper gastrointestinal tract. Carcinogenesis. 2007;28(3):685-90.
21. Santiago P. Ferrous versus ferric oral iron formulations for the treatment of iron deficiency: a clinical overview. Scientific World Journal. 2012;2012:846824.
22. Ruiz-Arguelles GJ, Diaz-Hernandez A, Manzano C, Ruiz-Delgado GJ. Ineffectiveness of oral iron hydroxide polymaltose in iron-deficiency anemia. Hematology. 2007;12(3):255-6.
23. Powell JJ, Bruggraber SFA, Faria N, Poots LK, Hondow N, Pennycook TJ, et al. A nano-disperse ferritin-core mimetic that efficiently corrects anemia without luminal iron redox activity. Nanomedicine: Nanotechnology, Biology and Medicine. 2014;10(7):1529-38.
24. Pan YH, Sader K, Powell JJ, Bleloch A, Gass M, Trinick J, et al. 3D morphology of the human hepatic ferritin mineral core: new evidence for a subunit structure revealed by single particle analysis of HAADF-STEM images. J Struct Biol. 2009;166(1):22-31.
25. Michel FM, Ehm L, Antao SM, Lee PL, Chupas PJ, Liu G, et al. The structure of ferrihydrite, a nanocrystalline material. Science. 2007;316(5832):1726-9.
26. Theil EC, Chen H, Miranda C, Janser H, Elsenhans B, Nunez MT, et al. Absorption of iron from ferritin is independent of heme iron and ferrous salts in women and rat intestinal segments. J Nutr. 2012;142(3):478-83.
27. Bejjani S, Pullakhandam R, Punjal R, Nair KM. Gastric digestion of pea ferritin and modulation of its iron bioavailability by ascorbic and phytic acids in caco-2 cells. World J Gastroenterol. 2007;13(14):2083-8.
28. Lonnerdal B, Bryant A, Liu X, Theil EC. Iron absorption from soybean ferritin in nonanemic women. Am J Clin Nutr. 2006;83(1):103-7.
29. Pereira DIA, Bruggraber SFA, Faria N, Poots LK, Tagmount MA, Aslam MF, et al. Nanoparticulate iron(III) oxo-hydroxide delivers safe iron that is well absorbed and utilised in humans. Nanomedicine: Nanotechnology, Biology and Medicine. 2014;10(8):1877-86.
30. Moretti D, Goede JS, Zeder C, Jiskra M, Chatzinakou V, Tjalsma H, et al. Oral iron supplements increase hepcidin and decrease iron absorption from daily or twice-daily doses in iron-depleted young women. Blood. 2015;126(17):1981-9.
31. Hutchinson C, Al-Ashgar W, Liu DY, Hider RC, Powell JJ, Geissler CA. Oral ferrous sulphate leads to a marked increase in pro-oxidant nontransferrin-bound iron. Eur J Clin Invest. 2004;34(11):782-4.

32. Barton Pai A, Pai MP, Depczynski J, McQuade CR, Mercier RC. Non-transferrin-bound iron is associated with enhanced Staphylococcus aureus growth in hemodialysis patients receiving intravenous iron sucrose. Am J Nephrol. 2006;26(3):304-9.
33. Cross JH, Bradbury RS, Fulford AJ, Jallow AT, Wegmuller R, Prentice AM, et al. Oral iron acutely elevates bacterial growth in human serum. Sci Rep. 2015;5:16670.
34. Latunde-Dada GO, Pereira DI, Tempest B, Ilyas H, Flynn AC, Aslam MF, et al. A Nanoparticulate Ferritin-Core Mimetic Is Well Taken Up by HuTu 80 Duodenal Cells and Its Absorption in Mice Is Regulated by Body Iron. The Journal of Nutrition. 2014;144(12):1896-902.
35. Aslam MF, Frazer DM, Faria N, Bruggraber SF, Wilkins SJ, Mirciov C, et al. Ferroportin mediates the intestinal absorption of iron from a nanoparticulate ferritin core mimetic in mice. Faseb J. 2014;28(8):3671-8.
36. Pereira DI, Mergler BI, Faria N, Bruggraber SF, Aslam MF, Poots LK, et al. Caco-2 Cell Acquisition of Dietary Iron(III) Invokes a Nanoparticulate Endocytic Pathway. PLoS One. 2013;8(11):e81250.
37. Pereira DIA, Aslam MF, Frazer DM, Schmidt A, Walton GE, McCartney AL, et al. Dietary iron depletion at weaning imprints low microbiome diversity and this is not recovered with oral nano Fe(III). MicrobiologyOpen. 2015;4(1):12-27.
38. Thurnham DI, McCabe LD, Haldar S, Wieringa FT, Northrop-Clewes CA, McCabe GP. Adjusting plasma ferritin concentrations to remove the effects of subclinical inflammation in the assessment of iron deficiency: a meta-analysis. Am J Clin Nutr. 2010;92(3):546-55.
39. Engle-Stone R, Nankap M, Ndjebayi AO, Erhardt JG, Brown KH. Plasma ferritin and soluble transferrin receptor concentrations and body iron stores identify similar risk factors for iron deficiency but result in different estimates of the national prevalence of iron deficiency and iron-deficiency anemia among women and children in Cameroon. J Nutr. 2013;143(3):369-77.
40. WHO. Guideline: Daily iron supplementation in infants and children. Geneva, Switzerland: World Health Organization, 2016.
41. Liu J, Gratz J, Amour C, Kibiki G, Becker S, Janaki L, et al. A laboratory-developed TaqMan Array Card for simultaneous detection of 19 enteropathogens. Journal of clinical microbiology. 2013;51(2):472-80.
42. Christian LM, Iams JD, Porter K, Glaser R. Inflammatory responses to trivalent influenza virus vaccine among pregnant women. Vaccine. 2011;29(48):8982-7.
43. Paine NJ, Ring C, Bosch JA, Drayson MT, Veldhuijzen van Zanten JJ. The time course of the inflammatory response to the Salmonella typhi vaccination. Brain Behav Immun. 2013;30:73-9.
44. Ma Y, Podinovskaia M, Evans PJ, Emma G, Schaible UE, Porter J, et al. A novel method for non-transferrin-bound iron quantification by chelatable fluorescent beads based on flow cytometry. Biochem J. 2014;463(3):351-62.
45. Singh S, Hider RC, Porter JB. A direct method for quantification of non-transferrin-bound iron. Anal Biochem. 1990;186(2):320-3.

46. Sebastiani G, Pantopoulos K. NTBI unveiled by chelatable fluorescent beads. Biochem J. 2014;463(3):e7-9.
47. Suchdev PS, Davis SM, Bartoces M, Ruth LJ, Worrell CM, Kanyi H, et al. Soil-transmitted helminth infection and nutritional status among urban slum children in Kenya. Am J Trop Med Hyg. 2014;90(2):299-305.
48. Ahmed A, Al-Mekhlafi HM, Al-Adhroey AH, Ithoi I, Abdulsalam AM, Surin J. The nutritional impacts of soil-transmitted helminths infections among Orang Asli schoolchildren in rural Malaysia. Parasit Vectors. 2012;5:119.
49. Committee WE. Prevention and control of schistosomiasis and soil-transmitted helminthiasis. World Health Organization technical report series. 2002;912:i-vi, 1-57.
50. Eren AM, Morrison HG, Lescault PJ, Reveillaud J, Vineis JH, Sogin ML. Minimum entropy decomposition: unsupervised oligotyping for sensitive partitioning of high-throughput marker gene sequences. Isme J. 2015;9(4):968-79.
51. Stallard N. Optimal sample sizes for phase II clinical trials and pilot studies. Statistics in medicine. 2012;31(11-12):1031-42.
52. De-Regil LM, Suchdev PS, Vist GE, Walleser S, Pena-Rosas JP. Home fortification of foods with multiple micronutrient powders for health and nutrition in children under two years of age. Cochrane Database Syst Rev. 2011(9):CD008959.
53. Dostal A, Baumgartner J, Riesen N, Chassard C, Smuts CM, Zimmermann MB, et al. Effects of iron supplementation on dominant bacterial groups in the gut, faecal SCFA and gut inflammation: a randomised, placebo-controlled intervention trial in South African children. The British journal of nutrition. 2014;112(4):547-56.

Supplements, appendices and other documents

Protocol #: 1489

Appendix 1: Project Timeline

Task Name	Start	Finish
**Project**	**Fri 01/04/16**	**Fri 01/03/19**
**Regulatory and Ethical approvals**	**Fri 01/04/16**	**Wed 30/09/16**
Protocol written	Fri 01/04/16	Fri 29/04/16
Sponsor review	Mon 02/05/16	Mon 20/06/16
CTA application	Tue 21/06/16	Wed 09/11/16
Ethics application	Tue 21/06/16	Fri 09/09/16
GMP clinical batch IHAT ordered	Wed 01/06/16	Fri 10/03/17
Interim report submitted (M 1)	Mon 01/08/16	Wed 30/09/16
Milestone 1	Wed 30/09/16	Wed 30/09/16
**Trial Setup and Recruitment**	**Tue 02/08/16**	**Wed 31/05/17**
GMP encapsulation of IHAT and comparators ordered	Thu 01/09/16	Thu 15/06/17
Protocol amendments	Mon 27/02/17	Fri 31/03/17
Trial protocol submitted	Mon 01/05/17	Fri 28/07/17
Trial supplies ordered	Mon 01/08/16	Fri 31/03/17
Trial documentation prepared	Mon 01/08/16	Fri 31/03/17
Trial database built	Mon 01/08/16	Fri 28/04/17
Setup of trial committees	Thu 01/09/16	Fri 16/12/16
Staff training at MRCG Basse	Mon 01/08/16	Thur 31/08/17
Upper river communities sensitization	Mon 12/12/16	Fri 28/07/17
Interim report submitted	Fri 05/05/17	Fri 05/05/17

Protocol #: 1489

Task Name	Start	Finish
Participant recruitment cohort 1	Mon 30/10/17	Fri 24/11/17
Participant recruitment cohort 2	Mon 29/01/18	Fri 23/02/18
Participant recruitment cohort 3	Mon 30/04/18	Fri 25/05/18
Local analysis of screening samples	Mon 30/10/17	Fri 01/06/18
Interim report submitted (M 2)	Fri 08/06/17	Fri 08/06/18
Milestone 2	Fri 08/06/18	Fri 08/06/18
**Trial Field Data Collection**	**Mon 04/12/17**	**Fri 31/08/18**
Cohort 1 intervention	Mon 04/12/17	Fri 23/02/18
Cohort 2 intervention	Mon 12/03/18	Fri 25/05/18
Cohort 3 intervention	Mon 18/06/18	Fri 14/09/18
AEs follow-up	Mon 04/12/17	Fri 05/10/18
Local analysis of trial samples	Mon 04/12/17	Fri 05/10/18
Interim report submitted (M 3)	Fri 26/10/18	Fri 26/10/18
Milestone 3	Fri 26/10/18	Fri 26/10/18
**Analysis and Reporting**	**Mon 06/08/18**	**Fri 01/03/19**
External analysis of trial samples (microbiome and NTBI)	Mon 17/09/18	Fri 21/12/18
Data queries resolved	Mon 30/10/17	Fri 05/10/18
Data analysis	Mon 08/10/18	Fri 25/01/19
Data monitoring committee report	Wed 28/01/19	Fri 01/02/19
Final project report	Mon 28/01/19	Fri 01/03/19
Publication of findings submitted	Mon 04/03/19	Sun 31/03/19
**Milestone 4 and Project End**	**Sun 31/03/19**	**Sun 31/03/19**

Appendix 2: Target Product Profile (TPP)

Proposed Targeted Product Profile (TPP) for IHAT for the treatment of IDA in children (i.e. following the proposed trial and once market authorisation is secured). For the current proposed trial we are working towards meeting the minimum acceptable results rather than the target, which would be for the next stage trial.

Product Properties	Minimum Acceptable Result	Target Result
**Dosing regimen**	Oral, at least four days/ week	Oral, once a day
**Formulation**	Single-API dose containing 20 mg Fe (bioequivalent to 12.5 mg Fe as $FeSO_4$) for children.	Multi-micronutrient powder formulations with taste masking for paediatrics, containing 20 mg Fe.
**Cost of treatment**	≤$0.05 per child dose	≤$0.01 per child dose
**Shelf-life of formulated product**	2 yr at ≤ 25°C	2 yr at ≤ 40°C
**Nutrient-nutrient interactions**	No unmanageable risk in terms of solid state or pharmacokinetic interactions	No risks in terms of solid state or pharmacokinetic interactions
**Clinical efficacy (reduction in anaemia after 3 months)**	30% of patients with Hb increase ≥ 1 g/dL	30% of patients with anaemia resolved (HB>11 g/dL)
**Clinical efficacy (reduction in iron deficiency after 6 months)**	30% of patients with ID resolved (sTfR/log10 ferritin (sTfR-F) index ≤2)	50% of patients with ID resolved (sTfR-F index ≤2)
**Safety**	Few drug-related SAEs, including diarrhoea	No drug-related SAEs<br>Minimal drug-related AEs, including diarrhoea
**Intestinal infection**	Drug-related bacterial infection in ≤ 15% of subjects	No drug-related bacterial infection
**Gut microbiome changes**	No significant increase in Enterobacteria or Enterobacteria/(Bifidobacteria +Lactobacillus	Less Enterobacteria relative to Bifidobacteria and Lactobacillus.

Appendix 3:

WORLD MEDICAL ASSOCIATION DECLARATION OF HELSINKI
Ethical Principles for Medical Research Involving Human Subjects

Adopted by the 18th WMA General Assembly, Helsinki, Finland, June 1964
and amended by the:
29th WMA General Assembly, Tokyo, Japan, October 1975
35th WMA General Assembly, Venice, Italy, October 1983
41st WMA General Assembly, Hong Kong, September 1989
48th WMA General Assembly, Somerset West, Republic of South Africa, October 1996
52nd WMA General Assembly, Edinburgh, Scotland, October 2000
53rd WMA General Assembly, Washington DC, USA, October 2002 (Note of Clarification added)
55th WMA General Assembly, Tokyo, Japan, October 2004 (Note of Clarification added)
59th WMA General Assembly, Seoul, Republic of Korea, October 2008
64th WMA General Assembly, Fortaleza, Brazil, October 2013

Preamble

1. The World Medical Association (WMA) has developed the Declaration of Helsinki as a statement of ethical principles for medical research involving human subjects, including research on identifiable human material and data.

The Declaration is intended to be read as a whole and each of its constituent paragraphs should be applied with consideration of all other relevant paragraphs.

2. Consistent with the mandate of the WMA, the Declaration is addressed primarily to physicians. The WMA encourages others who are involved in medical research involving human subjects to adopt these principles.

General Principles

3. The Declaration of Geneva of the WMA binds the physician with the words, "The health of my patient will be my first consideration," and the International Code of Medical Ethics declares that, "A physician shall act in the patient's best interest when providing medical care."

4. It is the duty of the physician to promote and safeguard the health, well-being and rights of patients, including those who are involved in medical research. The physician's knowledge and conscience are dedicated to the fulfilment of this duty.

5. Medical progress is based on research that ultimately must include studies involving human subjects.

6. The primary purpose of medical research involving human subjects is to understand the causes, development and effects of diseases and improve preventive, diagnostic and therapeutic interventions (methods, procedures and treatments). Even the best proven interventions must be evaluated continually through research for their safety, effectiveness, efficiency, accessibility and quality.

7. Medical research is subject to ethical standards that promote and ensure respect for all human subjects and protect their health and rights.

8. While the primary purpose of medical research is to generate new knowledge, this goal can never take precedence over the rights and interests of individual research subjects.

9. It is the duty of physicians who are involved in medical research to protect the life, health, dignity, integrity, right to self-determination, privacy, and confidentiality of personal information of research subjects. The responsibility for the protection of research subjects must always rest with the physician or other health care professionals and never with the research subjects, even though they have given consent.

10. Physicians must consider the ethical, legal and regulatory norms and standards for research involving human subjects in their own countries as well as applicable international norms and standards. No national or international ethical, legal or regulatory requirement should reduce or eliminate any of the protections for research subjects set forth in this Declaration.

11. Medical research should be conducted in a manner that minimises possible harm to the environment.

12. Medical research involving human subjects must be conducted only by individuals with the appropriate ethics and scientific education, training and qualifications. Research on patients or healthy volunteers requires the supervision of a competent and appropriately qualified physician or other health care professional.

13. Groups that are underrepresented in medical research should be provided appropriate access to participation in research.

14. Physicians who combine medical research with medical care should involve their patients in research only to the extent that this is justified by its potential preventive, diagnostic or therapeutic value and if the physician has good reason to believe that participation in the research study will not adversely affect the health of the patients who serve as research subjects.

15. Appropriate compensation and treatment for subjects who are harmed as a result of participating in research must be ensured.

Risks, Burdens and Benefits

16. In medical practice and in medical research, most interventions involve risks and burdens.

Medical research involving human subjects may only be conducted if the importance of the objective outweighs the risks and burdens to the research subjects.

17. All medical research involving human subjects must be preceded by careful assessment of predictable risks and burdens to the individuals and groups involved in the research in comparison with foreseeable benefits to them and to other individuals or groups affected by the condition under investigation.

Measures to minimise the risks must be implemented. The risks must be continuously monitored, assessed and documented by the researcher.

18. Physicians may not be involved in a research study involving human subjects unless they are confident that the risks have been adequately assessed and can be satisfactorily managed.

When the risks are found to outweigh the potential benefits or when there is conclusive proof of definitive outcomes, physicians must assess whether to continue, modify or immediately stop the study.

Vulnerable Groups and Individuals

19. Some groups and individuals are particularly vulnerable and may have an increased likelihood of being wronged or of incurring additional harm.

All vulnerable groups and individuals should receive specifically considered protection.

20. Medical research with a vulnerable group is only justified if the research is responsive to the health needs or priorities of this group and the research cannot be carried out in a non-vulnerable group. In addition, this group should stand to benefit from the knowledge, practices or interventions that result from the research.

Scientific Requirements and Research Protocols

21. Medical research involving human subjects must conform to generally accepted scientific principles, be based on a thorough knowledge of the scientific literature, other relevant sources of information, and adequate laboratory and, as appropriate, animal experimentation. The welfare of animals used for research must be respected.

22. The design and performance of each research study involving human subjects must be clearly described and justified in a research protocol.

The protocol should contain a statement of the ethical considerations involved and should indicate how the principles in this Declaration have been addressed. The protocol should include information regarding funding, sponsors, institutional affiliations, potential conflicts of interest, incentives for subjects and information regarding provisions for treating and/or

compensating subjects who are harmed as a consequence of participation in the research study.

In clinical trials, the protocol must also describe appropriate arrangements for post-trial provisions.

Research Ethics Committees

23. The research protocol must be submitted for consideration, comment, guidance and approval to the concerned research ethics committee before the study begins. This committee must be transparent in its functioning, must be independent of the researcher, the sponsor and any other undue influence and must be duly qualified. It must take into consideration the laws and regulations of the country or countries in which the research is to be performed as well as applicable international norms and standards but these must not be allowed to reduce or eliminate any of the protections for research subjects set forth in this Declaration.

The committee must have the right to monitor ongoing studies. The researcher must provide monitoring information to the committee, especially information about any serious adverse events. No amendment to the protocol may be made without consideration and approval by the committee. After the end of the study, the researchers must submit a final report to the committee containing a summary of the study's findings and conclusions.

Privacy and Confidentiality

24. Every precaution must be taken to protect the privacy of research subjects and the confidentiality of their personal information.

Informed Consent

25. Participation by individuals capable of giving informed consent as subjects in medical research must be voluntary. Although it may be appropriate to consult family members or community leaders, no individual capable of giving informed consent may be enrolled in a research study unless he or she freely agrees.

26. In medical research involving human subjects capable of giving informed consent, each potential subject must be adequately informed of the aims, methods, sources of funding, any possible conflicts of interest, institutional affiliations of the researcher, the anticipated benefits and potential risks of the study and the discomfort it may entail, post-study provisions and any other relevant aspects of the study. The potential subject must be informed of the right to refuse to participate in the study or to withdraw consent to participate at any time without reprisal. Special attention should be given to the specific information needs of individual potential subjects as well as to the methods used to deliver the information.

After ensuring that the potential subject has understood the information, the physician or another appropriately qualified individual must then seek the potential subject's freely-given informed consent, preferably in writing. If the consent cannot be expressed in writing, the non-written consent must be formally documented and witnessed.

All medical research subjects should be given the option of being informed about the general outcome and results of the study.

27. When seeking informed consent for participation in a research study the physician must be particularly cautious if the potential subject is in a dependent relationship with the physician or may consent under duress. In such situations the informed consent must be sought by an appropriately qualified individual who is completely independent of this relationship.

28. For a potential research subject who is incapable of giving informed consent, the physician must seek informed consent from the legally authorised representative. These individuals must not be included in a research study that has no likelihood of benefit for them unless it is intended to promote the health of the group represented by the potential subject, the research cannot instead be performed with persons capable of providing informed consent, and the research entails only minimal risk and minimal burden.

29. When a potential research subject who is deemed incapable of giving informed consent is able to give assent to decisions about participation in research, the physician must seek that assent in addition to the consent of the legally authorised representative. The potential subject's dissent should be respected.

30. Research involving subjects who are physically or mentally incapable of giving consent, for example, unconscious patients, may be done only if the physical or mental condition that prevents giving informed consent is a necessary characteristic of the research group. In such circumstances the physician must seek informed consent from the legally authorised representative. If no such representative is available and if the research cannot be delayed, the study may proceed without informed consent provided that the specific reasons for involving subjects with a condition that renders them unable to give informed consent have been stated in the research protocol and the study has been approved by a research ethics committee. Consent to remain in the research must be obtained as soon as possible from the subject or a legally authorised representative.

31. The physician must fully inform the patient which aspects of their care are related to the research. The refusal of a patient to participate in a study or the patient's decision to withdraw from the study must never adversely affect the patient-physician relationship.

32. For medical research using identifiable human material or data, such as research on material or data contained in biobanks or similar repositories, physicians must seek informed consent for its collection, storage and/or reuse. There may be exceptional situations where consent would be impossible or impracticable to obtain for such research. In such situations the research may be done only after consideration and approval of a research ethics committee.

Use of Placebo

33. The benefits, risks, burdens and effectiveness of a new intervention must be tested against those of the best proven intervention(s), except in the following circumstances:

Where no proven intervention exists, the use of placebo, or no intervention, is acceptable; or

Where for compelling and scientifically sound methodological reasons the use of any intervention less effective than the best proven one, the use of placebo, or no intervention is necessary to determine the efficacy or safety of an intervention.

and the patients who receive any intervention less effective than the best proven one, placebo, or no intervention will not be subject to additional risks of serious or irreversible harm as a result of not receiving the best proven intervention.

Extreme care must be taken to avoid abuse of this option.

Post-Trial Provisions

34. In advance of a clinical trial, sponsors, researchers and host country governments should make provisions for post-trial access for all participants who still need an intervention identified as beneficial in the trial. This information must also be disclosed to participants during the informed consent process.

Research Registration and Publication and Dissemination of Results

35. Every research study involving human subjects must be registered in a publicly accessible database before recruitment of the first subject. We will register this trial with the www.isrctn.com.

36. Researchers, authors, sponsors, editors and publishers all have ethical obligations with regard to the publication and dissemination of the results of research. Researchers have a duty to make publicly available the results of their research on human subjects and are accountable for the completeness and accuracy of their reports. All parties should adhere to accepted guidelines for ethical reporting. Negative and inconclusive as well as positive results must be published or otherwise made publicly available. Sources of funding, institutional affiliations and conflicts of interest must be declared in the publication. Reports of research not in accordance with the principles of this Declaration should not be accepted for publication.

Unproven Interventions in Clinical Practice

37. In the treatment of an individual patient, where proven interventions do not exist or other known interventions have been ineffective, the physician, after seeking expert advice, with informed consent from the patient or a legally authorised representative, may use an unproven intervention if in the physician's judgement it offers hope of saving life, re-establishing health or alleviating suffering. This intervention should subsequently be made the object of research, designed to evaluate its safety and efficacy. In all cases, new information must be recorded and, where appropriate, made publicly available.

CLINICAL TRIAL PROTOCOL

A novel nano-iron supplement (IHAT) to safely combat iron deficiency and anaemia (IDA) in young children: a double-blind randomised controlled trial

---

Protocol No:

**SCC No:**	**1489**
**Brief Title**	IHAT-Gut
**Other Number(s)**	
**Protocol Version – Date**	V3.0, 24 March 2017
**Sponsor**	Medical Research Council Unit The Gambia<br>PO Box 273 Banjul,<br>The Gambia, West Africa
**Chief Investigator**	Professor Andrew Prentice
**Local Principal Investigator**	Dr Mohammad Ilias Hossain
**Co PI:**	Dr Dora Pereira (University of Cambridge)

Table of contents

Page
Signature page 2
Protocol amendment(s) 3
Key roles 6
List of abbreviations 8
Protocol summary 10
1 Background information and rationale 13
1.1 Background information 13
1.2 Currently used oral iron and why IHAT is novel and dietary-like 14
1.3 Rationale 18
1.4 Potential risks and benefits 24
1.4.1 Risk mitigation 25
2 Study objectives 28
2.1 Study endpoints 29
3 Study design 31
3.1 Type of study and design 31
3.2 Randomisation and blinding procedures 32
3.2.1 Randomisation 32
3.2.2 Blinding 33
3.3 Sub-studies 34
3.4 Investigational products 34
3.4.1 Description of products 34
3.4.2 Formulation, packaging and labelling 35
3.4.3 Product storage and stability 35
3.4.4 Dosage, preparation and administration of investigational products 36
3.4.5 Concomitant medications/treatments 37
4 Selection and withdrawal of participants 37
4.1 Selection of participants 37
4.2 Eligibility of participants 39
4.2.1 Inclusion criteria 39
4.2.2 Exclusion criteria 39
4.3 Withdrawal of participants 40
5 Study procedures and evaluations 40
5.1 Study schedule 40
5.1.1 Study sensitisation 40
5.1.2 Screening and enrolment (baseline) 41
5.1.3 Follow-up (study visits) 42
5.1.4 Final study visit 44
5.1.5 Early termination visit 44
5.2 Study evaluations 46
5.2.1 Clinical evaluations 46

5.2.2 Laboratory evaluations 46
6 Safety considerations 49
6.1 Methods and timing for assessing, recording, and analysing safety parameters 49
6.1.1 Adverse events 50
6.1.2 Reactogenicity 50
6.1.3 Serious adverse events (SAEs) 50
6.1.4 Assessment of intensity of AEs 51
6.1.5 Assessment of causality 51
6.1.6 Serious Adverse Reaction (SAR) 53
6.2 Reporting procedures 53
6.3 Safety oversight 54
7 Discontinuation criteria 54
8 Statistical considerations 55
8.1 Sample size determination 55
9 Data handling and record keeping 63
9.1 Data management and processing 63
9.2 Source documents and access to source data 64
9.3 Protocol deviations 64
10 Quality control and quality assurance 64
10.1 Study monitoring 65
11 Ethical considerations 65
11.1 General considerations on human subject protection 65
11.1.1 Rationale for participant selection 66
11.1.2 Rationale for use of a placebo group 68
11.1.3 Evaluation of risks and benefits 69
11.2 Informed consent 70
11.3 Participant confidentiality 70
11.4 Future use of stored specimen 71
12 Financing and insurance 71
13 Publication policy 71
14 References 72
Supplements, appendices and other documents 76
Appendix 1: Project Timeline 77
Appendix 2: Target Product Profile (TPP) 79

Key roles

For questions regarding this protocol, contact Dr Dora Pereira, Department of Pathology, University of Cambridge, UK, diap2@cam.ac.uk, 0044-1223764864.

**Author(s):**	Dr Dora Pereira (University of Cambridge, diap2@cam.ac.uk) and Professor Andrew Prentice (MRCG, andrew.prentice@lshtm.ac.uk)
**Sponsor's representative:**	Dr Jonas Lexow (MRCG, jlexow@mrc.gm)
**Chief Investigator:**	Professor Andrew Prentice (MRCG, andrew.prentice@lshtm.ac.uk)
**Principal Investigator(s):**	Local PI: Dr Mohammad Ilias Hossain (Paediatrician, MRCG Basse, mihossain@mrc.gm)<br>Co-PI: Dr Dora Pereira (University of Cambridge, diap2@cam.ac.uk)
**Sub-Investigator(s):**	Ebrima Sise MRCG Keneba (support for iron assays)<br>Golam Sarwar MRCG Basse (study data manager/developer)<br>Lady Chilel Sanyang MRCG Basse (laboratory assays)<br>Amadou Jallow MRCG Keneba (laboratory assays)<br>Dr Davis Nwakanma MRC Unit The Gambia (Head of Laboratory Services)<br>Bolarinde Lawal MRC Unit The Gambia (Manager of Clinical Laboratories Fajara)<br>Tolulope Osunnuyi MRC Unit The Gambia, Keneba (Keneba laboratory manager, Analytical Project Manager)
**Trial Physicians:**	Dr Ogochukwu Ofordile, Study Research Clinician<br>Dr Mohammad Ilias Hossain, Local PI (Clinical Trial Coordinator)
**Sponsor Medical Expert:**	To be confirmed (we will consult Dr Muhammad Afolabi, Dr Ed Clark and other MRCG clinicians with paediatric expertise)

**Trial monitor(s):**	Sey Gibbi (MRCG Clinical Trials Support Office)
**Safety monitor(s):**	Local safety monitor (Dr Aderonke Odutola, Paediatrician)
**Chair of DSMB:**	Professor James Jay Berkley, Professor of Paediatric Infectious Diseases, KEMRI-Wellcome (Kilifi, Kenya)
**Chair of the TSC:**	Dr Margaret Pinder, MRCG Basse
**Statistician:**	Dr Nuredin Ibrahim Mohammed and Dr David Jeffries MRC Unit The Gambia (statistical support)<br>Dr James Wason MRC Biostatistics Unit, Cambridge (advisor for trial design, power calculation and primary outcome analysis, and member of TSC)
**Clinical Laboratory/ies:**	MRC Unit The Gambia, Keneba<br>MRC Unit The Gambia, Basse<br>MRC Unit The Gambia, Fajara
**Other institutions/ Collaborators:**	National Nutrition Agency [NaNA] (Modou Phall)<br>Wellcome Trust Sanger Institute, Cambridge, UK (Professor Julian Parkhill and Dr Josef Wagner)<br>King's College London, London, UK (Professor Robert Hider)
**Local Ethics Committee**	Gambia Government/MRC Joint Ethics Committee,<br>c/o MRC Unit The Gambia,<br>PO Box 273, Banjul, The Gambia, West Africa

List of abbreviations

ADME	Absorption, Distribution, Metabolism & Excretion
AE	Adverse Event
AGP	Alpha-1 acid Glycoprotein
API	Active Pharmaceutical Ingredient
BMGF	Bill and Melinda Gates Foundation
BSU	Biostatistics Unit
CI	Chief Investigator
CMO	Contract manufacturing Organisation
CRF	Case Report Form
CRP	C-reactive protein
DMT1	Divalent Metal Transporter 1
DSMB	Data & Safety Monitoring Board
EDTA	Ethylenediaminetetraacetic acid
FA	Field Assistant
FDA	Food and Drug Administration
FS	Field Supervisor
$FeSO_4$	Ferrous Sulphate
GCP	Good Clinical Practice
GMP	Good Manufacturing Practice
cGMP	Current Good Manufacturing Practice
GRAS	Generally Recognised as Safe
Hb	Haemoglobin
HNR	Human Nutrition Research (Unit)
ID	Iron Deficiency
IDA	Iron Deficiency Anaemia
ICH	International Conference on Harmonization
IEC	Independent Ethics Committee

IHAT	Iron Hydroxide Adipate Tartrate
IMP	Investigational Medicinal Product
LH	Lithium heparin
MHRA	Medicines and Healthcare products Regulatory Agency
MNP	Multimicronutrient Powder
MRC	Medical Research Council
MRCG	Medical Research Council Unit, The Gambia
NaNa	National Nutrition Agency
NTA	Nitrilotriacetic acid
NTBI	Non-transferrin bound iron
PI	Principal Investigator
RBV	Relative Bioavailability Value
RCT	Randomised Controlled Trial
RDT	Rapid Diagnostic Test
RHT	Regional Health Team
SAE	Serious Adverse Event
SD	Standard Deviation
SFA	Senior Field Assistant
SO	Scientific Officer
SOP	Standard Operating Procedure
SSP	Study Specific Procedure
sTfR	Soluble transferrin receptor
STH	Soil-Transmitted Helminths
TSAT	Transferrin saturation
TSC	Trial Steering Committee
TPP	Target Product Profile
XRD	X-Ray Diffraction
WHO	World Health Organization

Protocol summary

**Title:**	A novel nano-iron supplement (IHAT) to safely combat iron deficiency and anaemia (IDA) in young children: a double-blind randomised controlled trial.
**Alias :**	IHAT-Gut
**Phase**:	Phase II
**Population:**	Young Children (6-35 mo.)
**Number of participants:**	705
**Number of Sites:**	1
**Location of Sites (including satellite sites):**	One site, Communities in Upper River Division (North Bank villages within a 20 km distance from Basse)
**Trial Duration:**	
**- Clinical Phase:**	9 months of study supplementation/intervention
**- Whole trial:**	11 months field data collection
**Duration for Participants:**	12 weeks intervention<br>+ 4 weeks AE/SAEs active follow-up post-intervention
**Description of Investigational Products:**	- 1 dose/day single IMP containing IHAT powder bioequivalent to 12.5 mg Fe (i.e. 20 mg Fe taking into account IHAT's relative bioavailability to $FeSO_4$)<br><br>- 1 dose/day single IMP containing $FeSO_4$ powder equivalent to 12.5 mg Fe<br><br>- 1 dose/day containing a placebo powder (no-iron, 'sugar' compound)
**Objectives:**	In this trial we will test the hypothesis that supplementation with IHAT eliminates iron deficiency and improves haemoglobin levels in young children without increasing infectious diarrhoea or promoting negative changes in the gut microbiome or inducing gut inflammation.<br>The **primary objective** for this trial is to show non-inferiority of IHAT in relation to ferrous sulphate at correcting IDA, and in terms of diarrhoea to show superiority in relation to ferrous sulphate and non-inferiority in relation to placebo.

Secondary objectives are to show that IHAT is non-detrimental with respect to enteric pathogen burden, the gut microbiome, and intestinal inflammation.

Endpoints:

Primary endpoints:

There are 4 primary endpoints of the trial:

1. iron deficiency at 12 weeks
2. haemoglobin levels at 12 weeks
3. 'incidence density' of moderate-severe diarrhoea over the 12 weeks (i.e. the number of new moderate-severe diarrhoea episodes per child over the 12 weeks intervention)
4. 'period prevalence' of moderate-severe diarrhoea over the 12 weeks intervention period (i.e. the proportion of children with at least one episode of moderate-severe diarrhoea over the 12 weeks intervention)

To assess iron deficiency we will take into consideration the most up-to-date recommendation from WHO, who are currently conducting a consultation on this matter. The new WHO guidelines will include a recommendation for the best marker of iron deficiency in the context of inflammation and we expect this to be either using ferritin alone or the sTfR/logferritin index, where in both cases ferritin values will be inflammation-adjusted. More details on the possible methods to adjust ferritin values for inflammation are given in Section 2.1.

Iron deficiency and haemoglobin levels at 12 weeks will be used to define the prevalence of IDA which we will use to assess non-inferiority of IHAT relative to $FeSO_4$ in terms of efficacy at correcting IDA. We will determine the proportion of children in each arm which resolve iron deficiency **and** either achieve a normal Hb or an increase in Hb of at least 1 g/dL after the 12 weeks of the intervention.

'Incidence density' and 'period prevalence' of moderate-severe diarrhoea will both be used to assess superiority of IHAT relative to $FeSO_4$ for the diarrhoea outcome and non-

	inferiority relative to placebo for the diarrhoea outcome (i.e. safety).<br><br>More details about the 4 study hypothesis and evaluations are provided in Sections 2.1 and 8.1.<br><br>Moderate-severe diarrhoea refers to those diarrhoea episodes where (i) the child passes more than 5 loose or liquid stools per day, (ii) there is blood in the stool (dysentery), or (iii) the child shows signs of clinical dehydration (assessed by the study nurse based on physical signs such as little or no urination, sunken eyes, and skin that lacks its normal elasticity).<br><br>**Secondary endpoints:**<br>Secondary endpoints will be faecal microbiome diversity and profile (particularly in terms of abundance of Enterobacteria), abundance of enteric pathogens, faecal calprotectin (marker of gut inflammation), hospitalisation and morbidity (data collected three times per week using the questionnaire developed in the HIGH study), malaria infection (data collected every week with RDT), treatment failures (i.e. the number of children who have to stop the study because their Hb falls below 7 g/dL), the proportion of days a child has diarrhoea over the intervention period ('longitudinal prevalence'), the proportion of days a child has moderate-severe diarrhoea over the intervention period ('longitudinal prevalence' of moderate-severe diarrhoea), 'incidence density' of bloody diarrhoea (i.e. the number of bloody diarrhoea episodes per child-month of observation), markers of systemic inflammation (serum CRP and AGP), and systemic markers of iron handling (hepcidin, transferrin saturation and circulating non-transferrin bound iron - NTBI).
**Description of Study Design:**	3-arm (IHAT, $FeSO_4$, placebo), parallel, randomised, double-blind, placebo-controlled clinical trial.

1 Background information and rationale

1.1 Background information

Iron deficiency (ID) and its associated anaemia (IDA) is the largest nutritional deficiency disorder in the world and affects a total of 1.2 billion people, the majority of whom are children and women from resource-poor countries. Iron deficiency is frequently exacerbated by concomitant parasitic and bacterial enteropathogenic infections and contributes to almost 0.8 million deaths a year as well as irreparably limiting the cognitive development of children and leading to poor outcomes in pregnancy (1). Currently available iron compounds are cheap and readily available but constitute an unphysiological approach to providing iron that leads to significant side effects and serious adverse events (2-5). Data and meta-analysis from trials involving nearly ten thousand young children in developing countries have consistently shown that conventional soluble oral iron supplementation used to treat IDA is associated with increased infection including bloody diarrhoea (3, 6-8) and detrimental changes to the gut microbiome and gut inflammation (8, 9), further increasing the burden from enteric infection and environmental enteropathy (i.e. persistent gut damage and inflammation that leads to malabsorption), which is a major cause of growth failure in children in resource-poor environments (10, 11). Consequently, ID and IDA remain without an effective cure and we have so far been unable to decrease the burden of this disease in children, even after considerable effort and investment in the past 20 years (Figure 1).

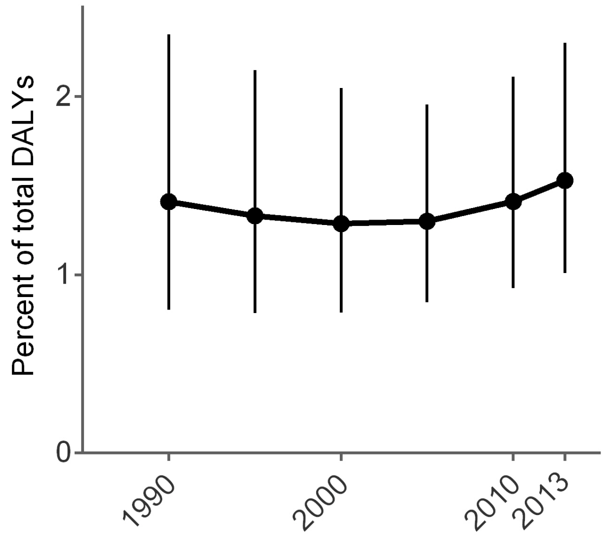

Figure 1. Global burden of iron deficiency anemia as a cause of disease in children under 5 y. Data is expressed as percent of disability adjusted life years (DALYs). Reference: Institute for Health Metrics and Evaluation (IHME). GBD Compare. Seattle, WA: IHME, University of Washington, 2015. Available from http://vizhub.healthdata.org/gbd-compare. (Accessed [13/01/2016])

The team at the MRC Human Nutrition Research Unit, have developed an innovative oral iron supplement (IHAT) for the safe treatment of iron deficiency and anaemia in resource-poor countries. Dr Pereira (who has been working at MRC HNR for the past 11 years) is a co-inventor of IHAT and is leading on its clinical development. As a next step in development we propose to conduct the first Phase II clinical trial with IHAT and obtain clinical data for its safety and efficacy in correcting IDA in young children. As mentioned above, many studies have shown that this is the population group that suffers the most debilitating effects of current iron supplementation

and, therefore, the group most at need of an alternative oral iron supplement that is safer, particularly in the gastrointestinal tract. Please refer to Section 11 (Ethical Considerations) for more details on our rationale for conducting this trial directly in children.

If IHAT is successful in the trial, these data will provide the evidence needed to encourage further investment so that IHAT can be implemented as a novel iron source for use in micronutrient intervention strategies aimed at children and women living in resource-poor countries and, hence, reduce the global burden of IDA.

The specific problem this study seeks to address relates to these serious side effects of oral iron supplements for treating anaemia in young children. Specifically, we will be addressing the fact that current iron supplements increase risk of infectious diarrhoea (3, 6-8) which promotes a pathogen-driven inflammatory response (8, 9, 12, 13) that impacts on hepcidin with reduction of iron absorption (14) and, therefore, offers limited benefit with added risk.

In the 21st century, we simply cannot accept the current view that increased risk of infectious diarrhoea in young children is normal collateral damage of oral iron supplementation in populations at high risk of enteric infection. This is a problem of current iron supplements that are all based on providing large bolus of non-physiological forms of 'gut-reactive' iron, that is soluble in the gut lumen, and to which humans have not previously been naturally exposed through diet. These harmful effects are caused by supplements that circumvent the natural chaperone and absorption/exclusion mechanisms of the gut.

We know that growth and virulence of bacterial enteropathogens is stimulated by these soluble iron supplements which are bioavailable to both the gut microbiome and the human host.

Conversely, our nano iron IHAT is effectively absorbed in humans without requiring solubilisation and, because it is not solubilised, it is not accessible as an iron source to bacterial enteropathogens or available to undergo significant redox cycling in the gut lumen (please refer to the summary of our pre-clinical and proof-of-concept data in Section 1.3 for details about IHAT's absorption, safety and metabolism)**. This means that supplementation with IHAT should correct IDA without increasing the burden from infectious diarrhoea and should result in improved overall response to iron supplementation compared to conventional soluble iron. <u>In essence, our nano iron looks like dietary non-haem iron and should behave like dietary iron.</u>**

1.2 Currently used oral iron and why IHAT is novel and dietary-like

The current paradigm for iron delivery is that only soluble ionic iron can be efficiently absorbed through DMT1 present in the apical membrane of duodenal enterocytes. This paradigm has supported the development, and use, over the past 20 years of interventions that aim to deliver

a large bolus of ionic iron to the enterocytes. Soluble ionic iron is not naturally present in the diet and, therefore, our bodies have only recently been exposed to significant amounts of this iron form through supplementation and fortification. Many different forms of iron have been tested, and are currently being used, but these all rely in the solubilisation of the compound in the stomach prior to the delivery of ionic iron, in the ferrous or ferric form, to the enterocyte cell.

Soluble ferrous iron (e.g. Fe(II) fumarate, Fe(II) bisglycinate) is redox reactive in the upper gastrointestinal tract and can redox cycle via Fenton chemistry promoting mucosal inflammation which leads to the acute side-effects normally reported with iron supplementation, such as heartburn, nausea and abdominal pain (15). Soluble ferric iron (e.g. Na Fe(III) EDTA, Fe(III) citrate, Fe(III) trimaltol, Fe(III) trisglycinate) is less redox reactive but soluble Fe(III) must still be reduced at the mucosal surface and may yield some 'sub-symptomatic' free radicals in the process, whilst the unabsorbed iron still delivers soluble iron to the colon where it is available to be utilized by pathogenic bacteria and to induce detrimental changes to the gut microbiome (8), and potentially increases risk of colorectal cancer (16-18). These effects may result in the distal and more chronic side effects of oral iron, namely diarrhoea or constipation. Furthermore, the high cost of ferric chelates limits their use in resource-poor countries. Haem iron supplements, such as heme iron polypeptide, are also expensive and there are marked concerns over haem safety in the colon in terms of catalizing the formation of N-nitroso compounds associated with cancer risk (19, 20). Other protein-bound forms of iron, such as lactoferrin and ferric mannitol ovoalbumin are several order of magnitude higher cost (~100 x), have limited shelf-life or are only useful for niche groups such as newborn babies.

Insoluble forms of iron, such as iron phosphates and elemental iron are not sufficiently bioavailable in humans to merit serious clinical use because they still require solubilisation in the stomach prior to absorption and the efficiency of this process is very low for these forms of iron. Ferric iron polymaltose is an insoluble iron compound made of aggregates of small iron oxo-hydroxide particles encased in a carbohydrate (polymaltose) coating. Even in the case these particles would be taken up whole by the enterocyte, they are too stable to be broken down and deliver enough iron for systemic use, hence their reported low bioavailability (21, 22).

Therefore, ferrous iron salts remain the oral iron compounds of choice because they are cheap and well absorbed and significant commercial effort and investment in the alternatives mentioned above has been unable to convince prescribers and governments that new oral iron preparations merit widespread use. Even in patients severely intolerant of standard oral iron, or in those where risk outweighs benefit, the increasing second line preference is for intravenous (IV) iron, in Western countries, and for 'no iron', in resource-poor countries.

IHAT is distinct from all these forms of iron, in that it is not soluble nor does it require solubilisation in the stomach to be absorbed since it is taken up by enterocytes as whole nanoparticles (Section 1.3). This means that the unabsorbed fraction of the compound that transits to the lower gut, and inherently this is at least 60% of all ingested oral iron irrespective

of the form, will remain nanoparticulate and, therefore not soluble, and as such will not be available to promote pathogen growth and tissue inflammation. An important aspect of IHAT structure is that this is sufficiently labile to break down effectively inside the enterocyte and deliver its iron because the native iron oxo-hydroxide structure (i.e. ferrihydrite) in IHAT has been purposely destabilised with the incorporation of dietary tartaric and adipic acids (23), much in the same way to what occurs in the ferritin iron core due to interactions with the aminoacid residues in the protein shell (24). IHAT is a tartrate-modified, nano-disperse Fe(III) oxo-hydroxide in the ferrihydrite mineral phase, formed in an adipate buffer, with similar functional properties and small primary particle size (~2 nm) as the iron form found in the ferritin core (i.e. ferrihydrite). Full physicochemical characterisation in relation to particle size, morphology, iron phase, XRD pattern, infrared spectra, and dissolution are presented in Powell *et al* (23). All components of IHAT (i.e. iron oxide, tartaric and adipic acids) are naturally present in foods and are approved food additives with a generally recognised as safe (GRAS) status by the US Food and Drug Administration (FDA). Besides composing the ferritin core, iron oxides are naturally present in foods and soils and are approved food additives (E172). Tartaric acid is naturally present in foods, most notably grapes, and is an approved antioxidant and acidifier food additive (E334). Adipic acid is also naturally found in foods and is an approved flavouring and gelling agent (E355).

Each daily dose of IHAT in this study will contain in addition to the 20 mg elemental Fe, 21 mg of tartaric acid and 4.7 mg of adipic acid. These amounts of the organic acids are less than 20% of the estimated ADI (acceptable daily intake) for a 5 kg child and less than 10% of the respective ADI for a 10 kg child (http://ec.europa.eu/food/fs/sfp/addit_flavor/flav15_en.pdf).

A molecular representation of IHAT is presented in Figure 2.

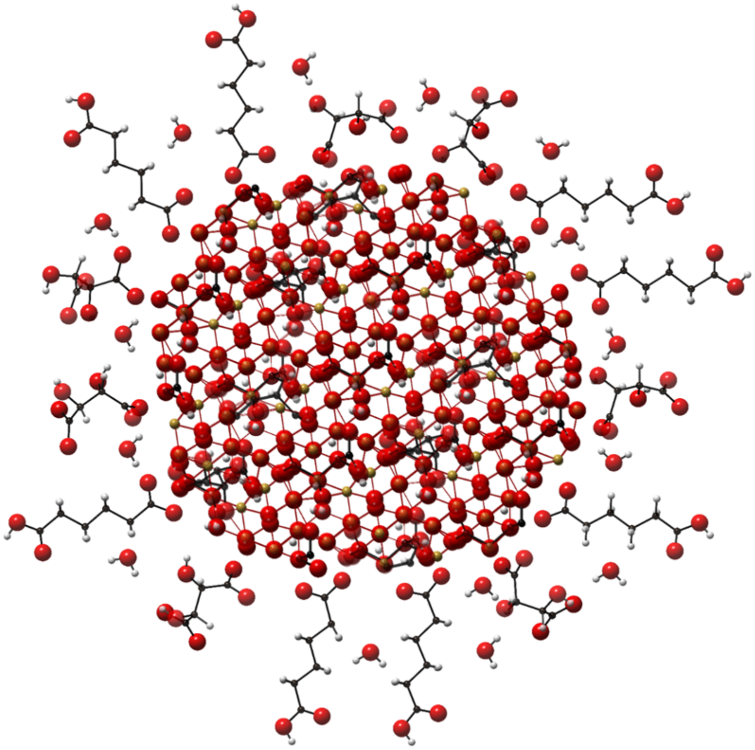

Figure 2. Molecular model of one IHAT 2 nm particle. The colours used to represent the atoms are: O (red), H (white), Fe (brown), C (black). The organic acids (tartaric and adipic) are represented adsorbed to the surface and incorporated into the structure core. We estimate that one IHAT particle would contain 150 Fe atoms (based on the ferrihydrite structure by Michel *et al* (25)). Chemical modelling by Dr Helen Chappell (unpublished).

Ferritin is composed of up to eight nanoparticles of iron oxo-hydroxide, also in the ferrihydrite mineral phase, stored in a protein shell that renders the iron particles both nano-dispersible and sufficiently labile to be utilized biologically. When ingested in either plant or animal based foods, it is well absorbed (26-28). The exact mechanism of absorption is not resolved- either ferritin is gradually broken down in the acidic, gastric lumen at a rate that matches later intestinal iron absorption or the particle resists total gastric degradation and is taken up whole by the enterocytes and gradually broken down intralysosomally to join the common iron pool. Either way, undigested nanoparticulate iron oxo-hydroxide should not be reactive in the same way that soluble iron is and, thus, should be poorly available to colonic bacteria or to participate in redox reactions at the epithelial surface (Section 1.3).

It was with this in mind, and based upon 20+ years of research into the chemistry of dietary minerals in the gastrointestinal lumen, that the co-PI and the team at MRC Human Nutrition Research have developed IHAT as a paradigm-shift from existing or pipeline products. IHAT is well absorbed and due to its nanoparticulate nature should carry very low side-effects as it should *not* undergo luminal redox cycling and it should *not* give up its unabsorbed iron to commensal and pathogenic bacteria (Section 1.3).

We are confident of IHAT's ability to outpace any other form of supplemental iron in use or under development. The anticipated advantages to the end user over competing and pipeline solutions will be (i) lack of acute 'sub-symptomatic', but nonetheless chemically undesirable, redox effects on the colonic mucosa and microbiome, (ii) reduced risk of intestinal infection and diarrhoea, and (iii) lack of symptomatic side-effects, all of which will ultimately result in better efficacy at correcting IDA.

1.3 Rationale

In this clinical trial we will test the hypothesis that supplementation with IHAT eliminates iron deficiency and improves haemoglobin levels in young children without increasing infectious diarrhoea or promoting negative changes in the gut microbiome or inducing gut inflammation. We propose to conduct the trial in the North Bank villages of the URR because these are some of the most deprived communities in the country where risk of infection, particularly enteropathogenic infection, is high. This will allow us to better distinguish IHAT and ferrous sulphate in terms of safety, which is where most of the clinical need for better iron supplementation lies.

Over the past ~10 years we have established sufficient pre-clinical and proof-of-concept data for IHAT to support moving to the Phase II trial proposed here. These data are summarised below and for the most part have been published. We have prepared an Investigator Brochure for IHAT comprising essentially the data summarised below, and have submitted this to the UK Medicines and Healthcare Products Regulatory Agency (MHRA), and asked for their scientific advice in relation to this protocol and the future development plan for IHAT, the MHRA's positive response letter is attached to this proposal. The Investigator Brochure for IHAT was also submitted to the Gambia Medicines Board, when we applied for clinical trial authorisation.

<u>IP protection</u>: IHAT is protected by an MRC-owned patent (WO2008096130) that has been granted in most major territories (e.g. Europe, US and China). The claims of this patent protect the composition-of-matter for the oral iron formulation to be taken into the clinic, as well as methods of manufacture and use for the preparation of a medicament for therapeutic delivery of IHAT to a subject. The claims also protect the chemistry underlying the generation of oxo-

hydroxide metal ion structures modified with organic moieties and thus offers broad protection of the chemical landscape.

Manufacture: A crucial advantage of IHAT, over competitors, is its facile synthesis leading to low manufacture cost compared to other complex iron formulations (namely iron chelates or protein-bound complexes). The cost of IHAT manufacture at scale is estimated as \$0.3-\$1.5 for a 28-day supply for one child (scale range 1-150 ton). This is only ~3x the cost of simple ferrous salts. Manufacture of IHAT involves a simple, but carefully-controlled, aqueous co-precipitation technique and product recovery is either by tray-drying or spray-drying. Based on feedback received from three contract-manufacture organizations (CMO) for the scale-up manufacture of IHAT, we are reassured that there will not be any significant hurdles in accommodating larger scale GMP manufacture.

Importantly, IHAT's manufacture is easily scalable and has a low cost due to the facile synthesis and inexpensive FDA GRAS (generally recognised as safe) raw materials. We anticipate that following this initial trial, we will have high-quality clinical data to support using IHAT instead of soluble iron (e.g. ferrous fumarate or sulphate) as the iron source in the micronutrient supplementation and home fortification (e.g. MNPs) interventions recommended by WHO to improve the iron status of populations. Following this initial pilot award, we would apply to BMGF for a full award that would be used to fund a trial where we would be investigating these different delivery systems with IHAT, different iron dosage regimens, and also assess efficacy in pregnant women. At that stage we would engage with the manufacturers of MNPs (for example DSM) to produce a formulation with IHAT instead of ferrous fumarate for testing.

Regulatory aspects: Scientific advice from the UK MHRA have assured us that the existing non-clinical and clinical data with IHAT would be sufficient to support the proposed trial (please refer to the MHRA letter dated 21st December 2016 attached to this proposal). Furthermore, advice from the MHRA in relation to a similar, but not identical, iron compound developed by our Group for a different indication was that toxicology studies would not be required and that the toxicology section of the CTA application could be comprised of data for the individual dietary components drawn from the literature. This compound has now completed Phase II testing in the USA.

For a Phase II/III adaptive trial in women (pregnant and non-pregnant) we also asked for advice from a regulatory consultancy and their report (commissioned to form part of a Wellcome Trust proposal pack) is also attached to this submission (Alacrita_151001 IHAT Regulatory Gap Analysis).

Commercial strategy: IHAT received the first prize at the 2014 Emerging Technologies Prize from the UK Royal Society of Chemistry (see http://www.rsc.org/chemistryworld/2014/12/solving-

iron-solubility-problem-profile-mrc). As part of that prize we have been partnered with GSK who will continue to offer 'in-kind' support and mentorship as needed to help translate IHAT and bring it to those most at need of an alternative oral iron compound. MRC already has significant commercial interest from various commercial partners. The results of this trial will be very beneficial to a company and open up a new market for IHAT in the developing world.

Pre-clinical data: The main findings from the proof-of-concept and pre-clinical studies that support our hypothesis are presented below.

Phase 0 pharmacokinetics human studies: A study with 4 iron deficient women in the UK showed that (i) bioavailability (i.e. red blood cell incorporation) of iron from IHAT was ~ 75% that of iron from ferrous sulphate and (ii) IHAT was successful in reducing the non-physiological post-absorptive iron surge caused by ferrous sulphate that raises transferrin saturation (29). A recent single-dose study in 30 pre-menopausal women in West Kiang, The Gambia (SCC1422 – IHAT) has confirmed that a new recovery-method for IHAT's large-scale manufacture does not affect IHAT's bioavailability (Figure 3A); showed again that IHAT was successful in reducing the non-physiological post-absorptive iron surge caused by ferrous sulphate, that raises transferrin saturation (Figure 3B); and IHAT was successful in reducing *ex vivo* pathogen growth in the serum collected from the women following the single-dose (Figure 3C). We note that the relative bioavailability (RBV) of IHAT in the Gambia study (range 10-69%) was lower than that in the UK study (range 58-89%). However, the UK study had very few numbers, only 4 women, and, therefore, the full range of IHAT bioavailability was not observed.

For the purpose of calculating the bioequivalent dose for IHAT to use in the proposed trial we have considered the upper 75% centile of the median RBV (i.e. 60%), we took into account the data from both studies (n=25) since they were both conducted in the same population group (i.e. pre-menopausal IDA women). Our reasoning for using the upper 75% centile rather than the median value was that in IDA young children (in the absence of infection) we expect to have increased iron absorption due to their increased erythropoietic needs for growth and brain development relative to adult women and, therefore, we did not want to overestimate the dose needed.

Most importantly, IHAT was developed to simulate dietary iron absorption and slowly release the iron into the circulation so as not to cause an abnormal rise in transferrin saturation and this was indeed confirmed with the Gambia study (SCC 1422), in which the vast majority of women had TSAT always below 40% following the 60 mg Fe dose. This feature of IHAT has a number of advantages over ferrous sulphate which induces the un-physiological iron bolus release into the circulation: (i) the lower rise in serum Fe and TSAT after each daily dose of IHAT should not cause a bolus rise in hepcidin which has been recently shown to inhibit absorption of the next-day dose with ferrous sulphate (30), (ii) less likelihood of formation of 'true' NTBI when TSAT is

maintained below 40% (31), (iii) less 'free' iron highly available to sustain growth of systemic extracellular pathogens (32, 33).

A

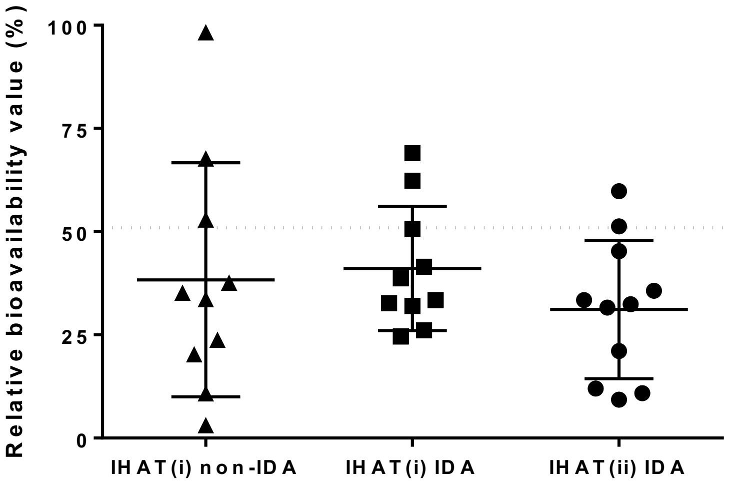

B

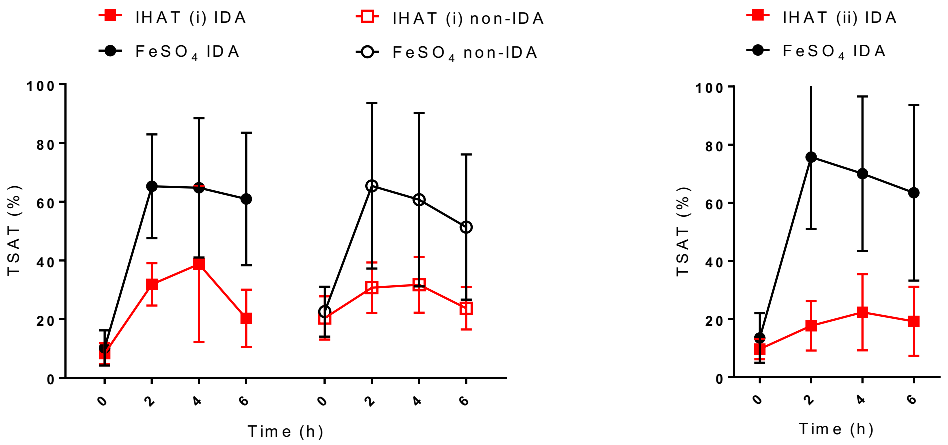

C

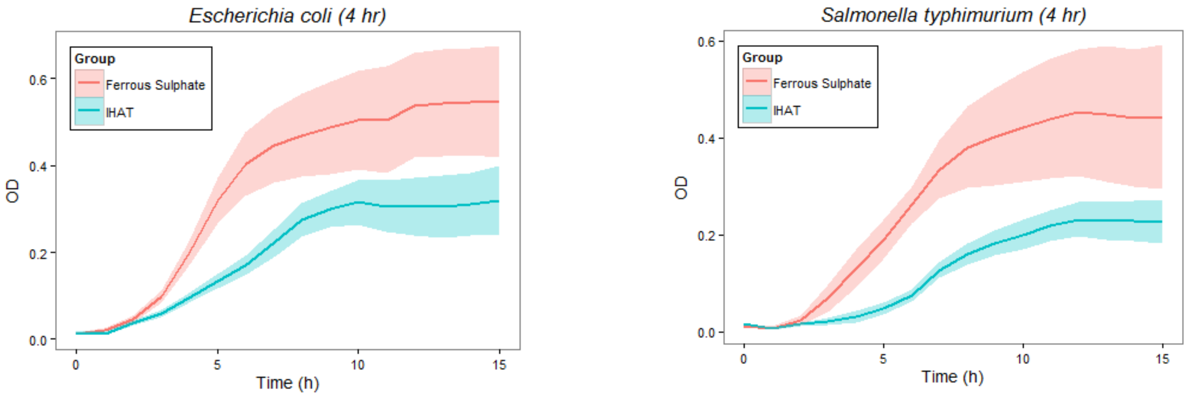

Figure 3. Unpublished data from the recent single-dose absorption study in pre-menopausal women in West Kiang, The Gambia (SCC 1422). A, relative bioavailability value (RBV) to ferrous sulphate (100%) determined from the red blood cell incorporation of the iron isotopes, box and whisker plots show median, minimum and maximum for n = 10 in each group, differences are not statistically significant; **B,** transferrin saturation following a single-dose of IHAT or ferrous sulphate (60 mg elemental Fe equivalent), data shown as mean (± SD, n=10/group), differences between IHAT and FeSO4 are statistically significant at the 2, 4 and 6 hours time-points, $p<0.01$; **C,** *ex vivo* bacterial growth of *E. coli* and *Salmonella typhimurium* in serum collected 4 hours following the single-dose of 60 mg Fe as IHAT or ferrous sulphate. There was strong evidence for the effect of supplementation group on bacterial growth ($p<0.0001$ using non-linear mixed effects modelling).

NOTE: Two identical IHAT powders were tested, these powders were recovered using a different procedure: IHAT (i) – tray-dried at 45 ^{o}C, IHAT (ii) – concentrated by solvent recovery and then tray-dried at 45 ^{o}C. The *ex vivo* assays were only conducted for IHAT(i).

Efficacy/haemoglobin repletion study in rats (14-days): Two independent studies have shown that IHAT is equivalent to ferrous sulphate at correcting haemoglobin levels in anaemia. No differences in bodyweight were observed between groups and there was no detectable iron deposition in the mucosa of the small intestine with IHAT (23, 29).

Pharmacokinetics mouse study (4 hours, radio-labelled): Absorption of IHAT was significantly increased in iron-deficient mice compared to iron-sufficient mice, showing that systemic absorption of IHAT is normally regulated by body iron levels (34).

ADME mouse studies (7 & 28 days): In two independent studies, IHAT was equivalent to ferrous sulphate at correcting haemoglobin levels. Systemic absorption of IHAT, i.e. basolateral export from the intestinal enterocyte into the blood circulation, was via ferroportin (as with ferrous sulphate) and was under regulation of normal iron homeostasis. Expression of duodenal DMT1, expression of liver hepcidin, liver iron, splenic iron and duodenal iron levels were all similar between the 2 groups (34, 35).

Mechanism of intestinal apical uptake - *in vitro* and *in vivo* studies:

In cellular and animal studies, IHAT was shown to be taken up apically as whole nanoparticles- i.e. a separate mechanism to the DMT1-driven uptake of conventional soluble iron supplements – and therefore did not require luminal or mucosal iron redox activity:

In mice, IHAT's absorption did not require redox activity (i.e. reduction of Fe(III) to Fe(II)) (23).

In Caco-2 differentiated cells (cells that resemble the duodenal enterocytes once differentiated), IHAT was taken up by the cells by endocytosis and this was followed by breakdown of IHAT inside endosomes/lysosomes within the cell to release the Fe (36).

In HuTu 80 cells (duodenal adenocarcinoma cells), IHAT was taken up by a mechanism independent of the divalent metal transporter 1 (DMT1) and of the duodenal cytochrome b (Dcytb) (34).

Correlation with human absorption: Caco2 Fe uptake studies

IHAT absorption in humans correlated with direct *in vitro* cellular uptake, but not with gastric solubility (29).

Importantly, we wish to note that even though IHAT is taken up apically by the enterocyte via a different mechanism to soluble iron, once inside the cell IHAT dissolves rapidly inside late-endosomes and lysosomes due to the combined action of low pH and high ligand-affinity for ferric iron (typically ~10mM citrate). Then, the iron released from IHAT in these cellular vesicles joins the common cellular labile iron pool (LIP) and does not bypass the normal physiological mechanisms that regulate iron absorption. Our data above indicate that IHAT nanoparticles do not translocate intact from the gut epithelium into the blood circulation.

Safety proof-of-concept *in vitro* assays: IHAT had no adverse effect on Caco-2 gut cell viability, even at doses 10-20 fold in excess of those possible in the gut lumen, whereas soluble ferrous

iron was markedly toxic at 14-fold lower doses than IHAT (29). IHAT had no adverse effects on epithelial cell monolayer integrity (36).

IHAT had no adverse effects on Caco-2 (colon) and Hutu-80 (duodenum) gut cell viability or on cell growth and proliferation even at doses much higher than those expected in the gut lumen. Unpublished data indicate that ferric maltol is more toxic still.

Safety proof-of-concept *in vivo* studies: In an 'enteric infection' (i.e. microbiome dysbiosis) rodent model, IHAT was much less available to potential enteropathogens than ferrous sulphate and led to a more beneficial gut microbiome, with increasing Lactobacilli after 14 days supplementation. (29).

In a 'healthy colon' rodent model, IHAT did not negatively impact the gut microbiome and appeared to be less available as an iron source to bacteria after 28 days of supplementation (37).

1.4 Potential risks and benefits

Our vision is that our novel iron compound (IHAT) will significantly contribute to the goal of safely eliminating iron deficiency and iron deficiency anaemia in women and children living in developing countries. Our existing evidence strongly supports the contention that IHAT offers very important advantages over any other form of supplemental iron in use or under development.

The anticipated advantages to the end user over competing and pipeline solutions will be (i) lack of acute sub-symptomatic, but nonetheless chemically undesirable, redox effects on the colonic mucosa and microbiome, (ii) reduced risk of intestinal infection and diarrhoea, and (iii) lack of symptomatic side-effects leading to better compliance; all of which will ultimately result in better efficacy at correcting iron deficiency anaemia. Furthermore, as described above, IHAT's absorption into serum follows a slower 'dietary-like' kinetics and it should not result in the non-physiological post-absorptive iron surge caused by ferrous iron salts, therefore, supplementation with IHAT should carry less risk of systemic infection such as tuberculosis and other co-infections normally associated with malaria. IHAT has a low-cost of manufacture due to its facile synthesis and inexpensive raw materials and, therefore, there should be no constraints to its implementation as an iron supplement for populations in resource-poor countries.

If IHAT is successful in this first trial, i.e. safer in terms of diarrhoea and gut effects than ferrous sulphate and not worse at correcting IDA, these data will be used to support a full award application to the BMGF together with a translation/commercial partner so that IHAT can be implemented as a novel iron supplement for use in micronutrient intervention strategies in developing countries and, hence, help to reduce the global burden of IDA. Ultimately, following the next trial (supposedly in pregnant women and children) we envisage that IHAT would be used instead of ferrous sulphate and ferrous fumarate in micronutrient intervention strategies

across the developing world. This fast-track clinical development (i.e. ability to move directly to Phase II and not Phase I studies) is only possible due to (i) the fact that iron supplements already have a WHO recommended daily active dose for the several age groups and (ii) the dietary-like nature of IHAT.

IHAT is an analogue of natural food iron and is made-up of dietary constituents that are approved food ingredients by the US Food and Drug Administration (FDA), namely iron oxide, tartaric acid and adipic acid and have the FDA generally recognised as safe (GRAS) status. More details about the dietary-like molecular structure of IHAT are explained in Section 1.2.

We have prepared a risk mitigation strategy for the proposed trial, in discussion with our program officer at the BMGF, and this is shown below. We are also required by the Sponsor to develop a risk assessment and risk mitigation strategies prior to study starting and we are currently working with MRCG to develop such documentation.

1.4.1 Risk mitigation

- Stability of MRC Unit The Gambia; the Unit has just been approved for renewed funding from the UK Medical Research Council until 2021.
- Regulatory approval for the clinical trial in children; based on scientific advice from the UK MHRA (mentioned above) and our previous experience with an iron compound targeting a different clinical application (phosphate binder for end-stage CKD patients) we are confident that we will obtain authorisation to proceed directly to the trial proposed here. With the MRC Unit The Gambia we have already conducted an early-stage clinical trial with IHAT (single-dose study SCC 1422) and both the Ethics Committee and the Medicines Control Agency in the Gambia have reviewed the pre-clinical data with IHAT for that study.

  We are submitting a clinical trial authorisation (CTA) to both the Gambia and the UK MHRA (for protocol review), even though we are not legally required to apply to the MHRA, since we feel this is best practice and in line with the EU/Horizon 2020 guidelines for best-practice for clinical trials conducted in developing countries (http://ec.europa.eu/research/participants/portal/doc/call/h2020/h2020-msca-if-2015/1645175-h2020_-_guidance_ethics_self_assess_en.pdf).

  We have contacted MHRA in relation to our intention to submit a CTA dossier for the trial proposed here and received confirmation that it would be appropriate to seek MHRA approval/review of the trial design and supporting pre-clinical data as part of a scientific advice meeting alongside local Gambia approvals. This MHRA meeting has now taken place and their scientific advice letter is attached to this proposal. The trial will be conducted in accordance

with the principles of GCP as laid down in the Consolidated Guideline for Good Clinical Practice published by the International Conference on Harmonization in 1996 (ICH GCP Guideline) and the MRC Unit The Gambia will Sponsor the research.

- Ethical approval; even though the planned trial is early-stage, given the long experience of the MRC in conducting oral iron interventions in children in The Gambia, the relatively low burden on study participants, and the use of children that are generally healthy apart from having IDA, we do not anticipate any ethical issues that could delay approval. The study protocol will be explained to the child mother/guardian orally in the presence of an independent and literate witness following MRCG SOPs in case they are illiterate or in writing and we will not start any study specific procedures before informed consent is obtained. The study investigates oral iron used in doses specifically recommended for this age group and even though IHAT is a novel compound, it is made up entirely of compounds naturally present in the diet and all our pre-clinical data supports that it should not behave any differently from dietary ferritin. In any case, even if IHAT is not absorbed and does not correct anaemia, it would not be worse in terms of safety that the forms of iron already in use in supplementation and home fortification strategies in this age group. We are assured that there should not be any ethical concerns over delaying 4-months the supply of iron treatment to the children enrolled in the placebo group since that it is not standard-of-care to provide routine iron supplementation to children in The Gambia and most children are not regularly screened for anaemia or IDA. All our children will be closely monitored during the study and all children that remain anaemic (Hb<11 g/dL) at the end of the study period (3 months intervention + 1 month AE follow-up), irrespective of their treatment group allocation, will be provided with iron supplementation for 3 consecutive months according to national and WHO guidelines. Severely anaemic children (i.e. Hb<7 g/dL) will not be enrolled in the study and if during the study a child (in any group) is found to become severely anaemic, they will stop participating in the study and will be offered iron supplementation for 3 consecutive months according to national and WHO guidelines. These children in the data analysis will be regarded as treatment failures. A full rationale for the inclusion of the placebo group is provided in Section 11.1.1.

- GMP manufacture of the clinical batch (low): as mentioned above, based on the feedback received for the scale-up manufacture of IHAT from CMOs, we are reassured that there won't be any significant hurdles in accommodating larger scale GMP manufacture. For the trial proposed here, the clinical batch of IHAT will be manufactured under cGMP conditions by Shasun Pharma Solutions in the UK and the powder-filling of the capsules, packaging and labelling will be provided by Capsugel in Belgium, also under cGMP conditions.

- Recruitment of participants (low); mitigated by highly experienced staff and well engaged communities in The Gambia. Assuming that ~ 50% of children under 3y in the Upper River region would fit the study eligibility criteria (the prevalence of low ferritin in this age group is based on our current data from the HIGH study) then we would need to screen ~1500 children. There are ~1700 children 6-35 mo. of age in the Wuli and Sandu districts in the North Bank

within a 20 km distance of the Basse field station from where we would initially recruit into the study. We would start recruitment in Nov 2017 for the 1st cohort and would finish in May 2018 for the 3rd cohort. We will monitor recruitment rates closely and if necessary we can extend the catchment area. The catchment area of the Upper River Division will be sensitised and a team led by the local PI (Clinical Trial Coordinator) and the field coordinator will tour all the villages in this area to sensitise them on the project.

- Increased malaria and co-infections risk during and after oral iron supplementation; there are risks associated with a large intake of iron supplements especially in areas of malaria endemicity. The dose of iron given daily in the reference arm (12.5 mg) is according to WHO guidelines for the age group children in non-malarious areas or malaria-endemic areas where it should be implemented in conjunction with measures to prevent, diagnose and treat malaria and co-infections. We have put in place the following strategies to mitigate the risk of possible interactions between iron supplements and malaria or other co-infections: (1) data from the Gambia over the last 5 years (medical records from the Kiang West region) shows that the peak malaria months are October and November and, therefore, we have timed the study intervention periods to avoid these months so that there will be no intervention/supplementation during Oct and Nov 2017 (2) trained field workers will be visiting all children every day during the 12 weeks supplementation period in order to supervise the administration of the iron supplements or placebo and on these occasions they will check on the children's health status and actively look for signs of malaria and co-infections, if a child shows signs of these infections the study nurse will perform adequate tests and the child will be offered the appropriate treatment/referral to the next Health Centre. In case of a fever, a malaria rapid test will be performed and if positive the child will be treated according to national guidelines. A sick child will always be visited by a study nurse for further clinical investigations and if needed referred to the nearest Health Centre. These visits will carry on for 4 weeks after the end of the study intervention and, during both the intervention and this follow-up period, morbidity data will be captured every other day. This is similar to what was done in the HIGH study and we do not anticipate any difficulties of implementation. Every week, the investigators will check whether children are RDT positive. Note: according to national guidelines, all children at birth are provided with bed nets and children in the URR receive intermittent preventive treatment (IPT) of malaria and so we have not anticipated we would need to provide insecticide-treated bed nets to the study population, but we could do this is deemed necessary.

- Analytical (low); mitigated by high level expertise of the laboratory technicians at the MRC Unit The Gambia and the internationally-recognised excellence of the Sanger Institute Group in microbiome and pathogen analysis and the expertise of the group at Kings College London in NTBI analysis.

2 Study objectives

In this trial we will test the hypothesis that supplementation with IHAT eliminates iron deficiency and improves haemoglobin levels in young children without increasing infectious diarrhoea or promoting negative changes in the gut microbiome or inducing gut inflammation.

There are four null hypotheses in the study:

1) non-inferiority of IHAT compared to ferrous sulphate for efficacy (in terms of Hb and iron deficiency correction: i.e. IDA) response probability (or prevalence). In this case we are testing the null hypothesis that: response probability in the IHAT arm minus the response probability in the ferrous sulphate arm is less than or equal to – 0.1. **We define 'response' for IDA as correction of iron deficiency and either achieving a normal Hb or an increase of at least 1 g/dL after 12 weeks of iron supplementation.**

2) superiority of IHAT compared to ferrous sulphate in terms of incidence density of diarrhoea. Here the null hypothesis is that the mean number of new episodes in the IHAT arm is greater than or equal to the mean number in the $FeSO_4$ arm.

3) superiority of IHAT compared to ferrous sulphate in terms of prevalence of diarrhoea. Here the null hypothesis is that the prevalence of diarrhoea in the IHAT arm is greater than or equal to the prevalence in the $FeSO_4$ arm.

4) non-inferiority of IHAT compared to placebo in terms of prevalence of diarrhoea. Here the null hypothesis is that the prevalence of diarrhoea in the placebo arm minus the prevalence in the IHAT arm is less than or equal – 0.1.

The primary objective for this trial is to show non-inferiority of IHAT in relation to ferrous sulphate at correcting IDA, and in terms of diarrhoea to show superiority in relation to ferrous sulphate and non-inferiority in relation to placebo (as defined above). Secondary objectives are to show that IHAT is non-detrimental with respect to enteric pathogen burden, the gut microbiome, and intestinal inflammation.

2.1 Study endpoints

Primary endpoints:

There are 4 primary endpoints of the trial:

1. iron deficiency at 12 weeks

2. haemoglobin levels at 12 weeks

3. 'incidence density' of moderate-severe diarrhoea over the 12 weeks (i.e. the number of new moderate-severe diarrhoea episodes per child over the 12 weeks intervention)

4. 'period prevalence' of moderate-severe diarrhoea over the 12 weeks intervention period (i.e. the proportion of children with at least one episode of moderate-severe diarrhoea over the 12 weeks intervention)

Efficacy primary endpoints:

To assess iron deficiency we will take into consideration the most up-to-date recommendation from WHO who are currently conducting a consultation on this matter. The new WHO guidelines will include a recommendation for the best marker of iron deficiency in the context of inflammation and we expect this to be either using ferritin alone or the sTfR/logferritin index, where in both cases ferritin values will be inflammation-adjusted. There are several models we can use to adjust ferritin for inflammation, for example use unadjusted ferritin with the cut-off with optimal sensitivity and specificity from the Malawi study (<18 µg/L), use the CRP/AGP regression model being investigated by Parmi Suchdev (BRINDA) to adjust ferritin values, use the Thurnham et al (38) stratified adjustment or the continuous adjustment used by Engle-Stone et al (39). The choice of the method for adjusting ferritin concentrations and of which marker to use to define ID (ferritin alone or the sTfR/logferritin index) will be made at the time of locking the data analysis plan, in consultation with an expert on this matter (Dr Sant-Rayn Pasricha), our BMGF Program Officer (Dr Ken Brown) and taking into consideration the results from Brinda and the results from the ongoing WHO consultation on this matter.

Iron deficiency and haemoglobin levels at 12 weeks will be used to define the prevalence of IDA which we will use to assess non-inferiority of IHAT relative to $FeSO_4$ in terms of efficacy. **We will determine the proportion of children who resolve iron deficiency <u>and</u> either achieve a normal Hb or an increase in Hb of at least 1 g/dL after 12 weeks of iron supplementation.**

Safety primary endpoints:

Incidence density and period prevalence of moderate-severe diarrhoea will be used to show superiority of IHAT in relation to ferrous sulphate and non-inferiority of IHAT in relation to placebo.

Diarrhoea is defined as 3 or more loose or watery stools per day and **moderate-severe diarrhoea** refers to those diarrhoea episodes where (i) the child passes more than 5 loose or watery stools per day, (ii) there is blood in the stool (dysentery), or (iii) the child shows signs of clinical dehydration (assessed by the study nurse based on physical signs such as little or no urination, sunken eyes, and skin that lacks its normal elasticity). These episodes will require treatment (including ORS) and will be recorded as adverse-events.

'Incidence density' is defined as the number of new moderate-severe diarrhoea episodes per child over the 12 weeks intervention and 'period prevalence' is defined as the proportion of children with at least one episode of moderate-severe diarrhoea over the 12 weeks intervention.

Secondary endpoints:

Secondary endpoints will be faecal microbiome diversity and profile (particularly in terms of abundance of Enterobacteria), abundance of enteric pathogens, faecal calprotectin (marker of gut inflammation), hospitalisation and morbidity (data collected three times per week using the questionnaire developed in the HIGH study), malaria infection, treatment failures (i.e. the number of children who have to stop the study because their Hb falls below 7 g/dL), the proportion of days a child has diarrhoea over the 12 weeks intervention period ('longitudinal prevalence' of diarrhoea), the proportion of days a child has moderate-severe diarrhoea over the 12 weeks period ('longitudinal prevalence' of moderate-severe diarrhoea), 'incidence density' of bloody diarrhoea (i.e. the number of bloody diarrhoea episodes per child-month of observation), markers of systemic inflammation (serum CRP and AGP), and systemic markers of iron handling (hepcidin, sTfR, transferrin saturation and circulating non-transferrin bound iron - NTBI).

sTfR and hepcidin will be assessed at 0, 12 weeks and all other outcome measures will be assessed at 0, 2 and 12 weeks in all the children.

The 2 weeks time-point will provide an indication of acute compound-related effects and the 12 weeks time-point of chronic (i.e. longer term/chronic) effects.

3 Study design

3.1 Type of study and design

3-arm, parallel, randomised, placebo-controlled, double blind.

Children will be randomised (1:1:1) to IHAT, ferrous sulphate or placebo, each arm will include an intervention period of 12 weeks, faecal and blood samples will be collected at the time-points indicated below.

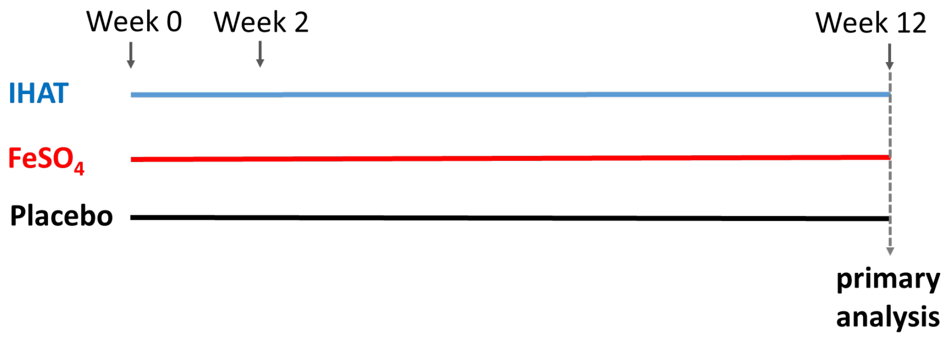

Participants will be iron deficient anaemic young children (n=705, accounting for 15% loss to follow-up) living in rural communities in the Upper River Division in The Gambia. Children will be recruited and randomised into the 3 study arms (n=235 per arm). Inclusion criteria will be: apparently healthy as judged by a study nurse on the day of recruitment, age 6-35 months, no malaria (RDT negative) and IDA defined as $7 \leq Hb < 11$ g/dl (for recruitment we will use Medonic Hb values rather than HemoCue) with serum ferritin<30 µg/L.

Each arm will include an intervention period of 12 weeks. As suggested by our BMGF proposal reviewers, we have included a placebo arm to allow us to fully evaluate IHAT effects, particularly to rule-out negative impact on diarrhoea episodes, the gut microbiome and compound-related bacterial infection, and to determine treatment effect sizes with either IHAT and ferrous sulphate that will be necessary to power future iron supplementation studies (please refer to Section 11.1.1.).

3.2 Randomisation and blinding procedures

3.2.1 Randomisation

Recruited children (N=705) will be randomly assigned (computer generated) to receive one of the 3 treatment arms (n=235 in each arm) stratified by the Hb concentration prior to enrolment and age. This will assure that Hb concentration at baseline (as a proxy for erythropoietic demand, which is the main driver for iron absorption) will not differ between the 3 arms.

At recruitment, each child will be categorised into two Hb classes (below or equal to/above the median Hb for that recruited cohort) and also according to age into 3 classes (6-11 months, 12-23 months and 24-35 months). This will divide children into 6 different strata and in each strata the children will be randomly assigned to one of the 3 treatment arms using a computer program and a block randomisation approach with fixed block size by age and Hb levels as defined above.

Ideally subjects will be recruited so that there will be balanced numbers in each of the 3 age classes; each of the age classes are further categorised into two groups based on the Hb level. Thus, in the balanced case, a total of 117-118 subjects each will belong to one of the six strata, created by Hb level (Low, High) and age group (Young, Middle, Old) combinations:

a. Low Hb, young age group
b. Low Hb, middle age group
c. Low Hb, old age group
d. High Hb, young age group
e. High Hb, middle age group
f. High Hb, old age group

However, 204 random treatment allocations will be generated for each strata in order to allow flexibility with respect to the numbers recruited in each of the six groups (a)-(f). That is, the list of treatment sequences in the balanced case will be extended by 86-87 to account for inbalance in the ages of children enrolled in the study as it may not be possible due to the age demographics of the study communities to enrol equal numbers in each of the age classes above. In any case, we will ensure that equal numbers within each strata are assigned to one of the treatment arms. This will be based on a pre-specified list of IDs ranging from 001-204, 205-408, 409-612, 613-816, 817-1020 and 1021-1224 for groups (a)-(f) above, respectively.

The study supplements will be provided in 750 individual bottles from the manufacturer. These bottles will be labelled using a random bottle ID ranging from 1 to 750 with a check digit character. The check digit character will be augmented to each of these numbers to make up the full subject IDs which will be generated using the Damm algorithm (https://en.wikipedia.org/wiki/Damm_algorithm; date accessed 08 November 2016). Here the

check digit numbers 0-9 will be converted to letters A-H and J-K. Thus, there will be 250 bottles with randomly assigned bottle IDs for each of the three treatments (A, B or C). An electronic copy of the list will be maintained to allow electronic randomisation. The key code to which IMP (IHAT, $FeSO_4$ or placebo) corresponds to A, B or C will not be known to the statistician running the randomisation nor to anyone in the study team.

After children in each cohort are classified into one of the six strata based on their age and Hb level, a single bottle will be assigned to the child by selecting a bottle from one of the three groups (A, B or C) according to the treatment allocation, as determined above. The procedure will be repeated until the target number of subjects (n=705) are randomised. This will be performed using a computer programme. A complimentary programme will also be developed with a Microsoft Access application that will generate the appropriate bottle ID for a given screening ID which can be used on site. The bottle IDs will be used as subject/randomisation IDs and written on all relevant forms.

The randomisation code and the application to generate subject IDs on site will be written by Nuredin Mohammed (the trial statistician based at MRCG) and reviewed by David Jeffries (the head of Statistics MRCG). The final code will be run using a random seed number by a member of the Statistics department who is independent of the trial. The generated randomisation list and the seed number will be recorded in a randomisation database (MS access database). The database and the application will be password protected and stored in a secure server.

After randomisation, the allocation list will be kept electronically and access will be granted to an independent clinician/nurse at the Basse site. A paper copy of the allocation list will be kept securely in the Sponsor's office (by an independent statistician). The DSMB will have access to grouped data but we would provide unblinded data to the DSMB for their closed meetings if requested. If emergency unblinding is required at the request of the DSMB, only the particular study subject in question will be unblinded, since we will have an individual supplement code for each participant. Following investigation by the DSMB of an emergency unblinding case, and at the request of the DSMB, we may also unblind a whole treatment arm or the entire study. In all cases, someone independent from the trial team will perform the unblinding.

3.2.2 Blinding

Participants, field workers (Senior Field Assistants, Field Assistants, Field Supervisor and Field Coordinator), study nurses, scientific officers, research clinician, local PI (Clinical Trial Coordinator), CI, Clinical Trial Monitor and the entire study team will be blinded as to which treatment group participants belong to. Each treatment dose (iron compounds and placebo) will be encapsulated in identical capsules (also containing powders of identical colour) by Capsugel in France and the supply of capsules for each child will be packed in one bottle that will be

individually labelled with a random bottle ID ranging 001-750 with a check digit character. This means that each child will have a unique treatment code that will also be their study ID/ randomisation number as specified in 3.2.1. This individual labelling has the advantage of allowing to unblind only one child, if it becomes necessary, without compromising the blinding of the study.

The manufacturer will provide 750 individual bottles (allowing for some spares) comprising 250 bottles with randomly assigned bottle IDs for each of the three treatments.

MRCG will pass on the randomisation list to Capsugel with the treatment code key (A, B, or C) corresponding to each of the bottle IDs and Capsugel will make the decision of which compound is A, B or C. A Sponsor representative will keep the allocation list safe and since Dr Pereira (co-PI) will also remain blinded, we suggest an independent statistician acts as the key holder for the treatment arm codes and allocation list. This list will be kept in electronic format (password protected) and in paper copy (locked cabinet).

The pre-packed weekly supplies of the capsules will be prepared following a study-specific procedure (SSP) by the Scientific Officer (SO) and the process supervised by the clinical trial coordinator (local PI) in Basse and labelled with each participant's ID, they will then be handed over to the Field Coordinator who is responsible for distribution to the field workers who will supervise consumption according to the pre-defined protocol for administration of the oral doses. Staff will be fully trained in all aspects of this activity and quality-control measures will be put in place to ensure that the SSP is followed exactly.

The blinding for a particular study participant may be broken if safety issues arise and we are advised by the DSMB to do so. A study-specific procedure describing the unblinding process will be developed.

3.3 Sub-studies

None.

3.4 Investigational products

3.4.1 Description of products

The trial has 3 arms:

IHAT- iron hydroxide adipate tartrate: an analogue of natural food ferritin iron (as detailed in Section 1.2); **Ferrous sulphate**- the gold standard for iron supplementation and **Placebo** (sucrose).

To avoid the need to use any tablet compression excipients or encapsulation materials, and to investigate the effects of just the active IMP compounds, the iron preparations, i.e. IHAT and ferrous sulphate, and placebo comparator, will be supplied as powders with each dose contained in a hard-gelatine powder-filled easy-open capsule.

3.4.2 Formulation, packaging and labelling

Each daily dose will be contained in a hard-gelatine powder-filled easy-open capsule. Each capsule will contain the single active IMP compound (IHAT or ferrous sulphate) or the placebo compound mixed with a small amount of a food colorant to colour-match the IMP powders. Capsules for each treatment will be packed in medicine bottles, each bottle containing enough capsules for one child for the entire study duration (allowing for some spares in case of accidental loss), and these bottles will be individually labelled with a unique code per participant, which will also be the subject randomisation code as described above. The key to which treatment corresponds to which code will be unknown to the study team or study PIs. The weekly supplies of each treatment for each child will be packed in Basse by the SO supervised by the clinical trial coordinator (local PI) and labelled with each participant's ID, which will be the same as the bottle ID as described in the randomisation procedure above. The bags with the weekly supplies per child will then be handed over to the Field Coordinator who is responsible for distribution to the field workers who will supervise consumption according to the pre-defined protocol for administration of the oral doses.

3.4.3 Product storage and stability

The capsules will be stored in dry conditions (the capsules will be packed in bottles containing desiccator sachets incorporated in the lids) and away from direct sunlight at MRC Basse (this will be in an air-conditioning room in MRC Basse clinical services below 25°C). The products are stable for 24 months if kept below 25°C. A full long-term storage stability assessment is going to be performed by Capsugel, the formulation company preparing the capsules (following recommended ICH protocols for the Gambia climate region: 25°C/60%Relative Humidity (representing normal storage conditions) and 30°C/65%Relative Humidity (representing transport conditions)) for both IHAT and comparators and the capsules will be packed and stored accordingly to these findings (these data will be available prior to study starting when the cGMP capsules are released by Capsugel to be shipped to the Gambia).

The weekly supplies taken out to the field do not require any special short-term storage conditions and will be kept by the field workers in their study bags in sealed plastic bags.

3.4.4 Dosage, preparation and administration of investigational products

The very recent WHO recommendation for the iron supplementation daily dose is 10-12.5 mg elemental Fe for infants 6-23 mo. and 30 mg elemental iron for children 24-59 mo. (40). The iron dose for ferrous sulphate will be 12.5 mg elemental Fe (62.5 mg ferrous sulphate heptahydrate) once a day for our entire study population (6-35 mo.), rather than having 2 different dosages according to the age groups, as this is still in line with the Gambian national guidelines. The iron dose for IHAT will be the iron-bioavailability equivalent (bioequivalent) in relation to ferrous sulphate as defined above in Section 1.2, i.e. assuming RBV of 60%. The bioequivalent iron dose for IHAT will be 20 mg elemental Fe (equivalent to 80 mg IHAT powder).

Each daily dose will be contained in a hard-gelatine powder-filled easy-open capsule. On the day of administration, the field assistant will open the respective capsule (corresponding to the child study ID according to the randomisation) and add the entire powder content of the capsule to 10 ml of a sugar juice drink contained in a disposable plastic cup. The drink will be used to mask the metallic taste of the iron powders, so that the supplementation is more acceptable to the child. The dose will be administered directly into the child's mouth using a disposable Pasteur pipette (in the younger children), a big spoon or drunk directly from the plastic cup (in the older children). There will be a Study Specific Procedure for this dose-administration protocol, which will be followed by each field worker and specific training will be provided before the start of the study. Whenever possible, each dose will be ingested after a feed or within 1 hour of the last meal, since iron supplements are generally better tolerated when ingested after meals rather than on an empty stomach. If, exceptionally, the child has not been fed before supplementation, the mother will be encouraged to feed the child immediately after supplementation.

In order to be able to do this protocol for supplement administration and not compromise the double-blind design of the trial we have been working with the formulation company (i.e. Capsugel) producing the capsules to ensure that all powders (IHAT, ferrous sulphate and placebo) have the same colour and a small amount of a food colorant will be added to all the powders prior to filling the capsules). The IHAT powder will be produced by Shasun Pharma Solutions in the UK (now called Sterling Pharma) and sent to Capsugel who will formulate the IHAT and source and formulate the ferrous sulphate and placebo (sucrose) in the gelatine capsules.

3.4.5 Concomitant medications/treatments

Participants will remain under the care of the Wuli and Sandu RCH teams.

Any subject whose Hb falls below 7g/dL will be referred to the closest health centre to receive iron supplementation for 3 consecutive months according to national and WHO guidelines. We will support the RCH systems by ensuring a constant supply of malaria diagnostics, anti-helminth treatment, basic drugs and iron syrup (for all young children – not just study participants) for the duration of this trial. All children participating in the study who remain anaemic after the study intervention and follow-up period (i.e. after the 4 months) will be offered the iron syrup for 3 consecutive months according to national and WHO guidelines.

No specific medication is prohibited during the study, with the exception that participants in the study are not allowed to take any other form of iron supplementation, including multimicronutrient powders containing iron and iron syrups for the entire duration of the study, i.e. during the 12 weeks intervention and 4 weeks AEs follow-up.

4 Selection and withdrawal of participants

4.1 Selection of participants

Since the welfare card/immunisation cards in the North Bank of the URR are not available for all children and records are unreliable, children between the age of 6-35 months will be identified through the enumeration data collected by the study field team within the study catchment area in the Wuli (17 study villages) and Sandu (12 study villages) districts (distance of ~ 20 km North of Basse, Figure 4). The compounds within the study catchment area were identified from the mapping exercise conducted by our field team. These mapping and enumeration/census data are currently being collated in an electronic database and will be linked with the eCRFs.

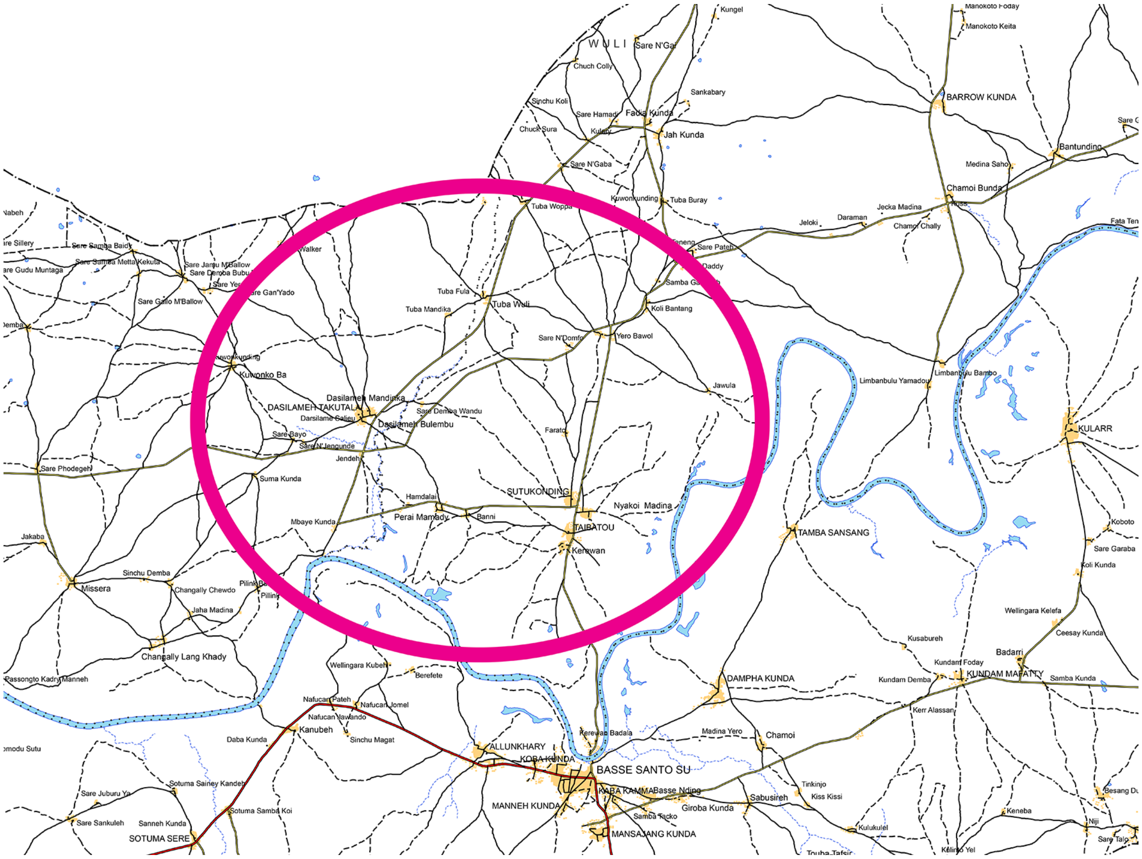

Figure 4. Study Catchment area. The area demarked represents an area of ~ 15 Km radius (at a maximum distance of 20 km by straight line from Basse), and corresponds to an area perimeter of ~ 45 km. Study nurses will be stationed at the following health facilities in the Nortn Bank of the URR: Yorrobawol Health Center, Darsilami Community Health Post, Konkuba Community Health Post, and Taibatu Health Post.

Assuming that ~ 50% of children under 3 y. in the Upper River region would fit the study eligibility criteria (the prevalence of low ferritin in this age group is based on our current data from the HIGH study) then we would need to screen ~1500 children. From our community census exercise, we estimate that there are ~1700 children 6-35 mo. of age in the Wuli and Sandu districts in the north Bank within a 20 km distance of the Basse field station from where we would initially recruit into the study. We would start recruitment in Nov 2017 for the 1st cohort and would finish in May 2018 for the 3rd cohort. We will monitor recruitment rates closely and if necessary we can extend the catchment area.

The catchment area of the Upper River Division will be sensitised and a team led by the local PI (Clinical Trial Coordinator) will tour all the villages in this area to sensitise them on the project.

For the sensitisation we will use the Study Communication Plan developed together with the MRCG Communications Team.
The Regional Health Team (RHT) and MRC Basse will advise on all studies taking place in the area. All mothers/guardians of eligible children will be asked if their child participates in another study and children will only recruited if this is not the case. They will also be asked if they intend to remain in the study catchment area for the entire duration of the study period (i.e. 4 months).

4.2 Eligibility of participants

Participants must meet all of the inclusion criteria and none of the exclusion criteria to be eligible to participate in the trial.

4.2.1 Inclusion criteria

Apparently healthy as judged by a study nurse at day of screening and recruitment
Age 6-35 mo.
Free of malaria (RDT negative)
HAZ, WAZ, WHZ >-3 SD
IDA defined as 7≤ Hb <11 g/dl AND ferritin<30 μg/L, as per WHO recommendation for children under 5y that live in regions with high infection burden (14)
Resident in the study area (and planning to remain in the study area for the duration of the trial)
Ability and willingness to comply with the study protocol (daily intake of supplement and daily study visits with weekly finger prick)
Informed consent given by parent or guardian

4.2.2 Exclusion criteria

Congenital disorders
Chronic disease
Currently participating in another study
Currently taking iron supplements/multiple micronutrient supplements
Currently experiencing moderate-severe diarrhoea, defined as those diarrhoea episodes where (i) the child passes more than 5 loose or watery stools per day, (ii) there is blood in the stool (dysentery), or (iii) the child shows signs of clinical dehydration (assessed by the study nurse based on physical signs such as little or no urination, sunken eyes, and skin that lacks its normal elasticity), will usually require treatment (including ORS)

4.3 Withdrawal of participants

A study participant will be discontinued from participation in the study if:

- Hb concentration falls < 7 g/dL
- Any clinically-significant adverse event (SAE), laboratory abnormality, intercurrent illness, or other medical condition or situation occurs such that continued participation in the study would not be in the best interest of the participant
- Development of a chronic disease
- Participation in another study

Note that there is no formal withdrawal criteria for participants that develop moderate-severe diarhoea episodes since this is a primary outcome of the trial. However, children that show clear signs of infectious diarrhea, such as blood in the stool, will be provided with appropriate treatment and the causative agent for the infection will be determined using the TaqMan Array Card (TAC) system (41).

Participants are free to withdraw from the study at any time without giving a reason.

5 Study procedures and evaluations

5.1 Study schedule

5.1.1 Study sensitisation

The Management and Field team of MRC Basse have already been engaged with the proposed trial and have offered their support. An initial mapping of the catchment area has also been carried out by the Field Coordinator and visits to some of the health clinics in the area were conducted. The Regional Health Team (RHT) and staff of the health facilities responsible for the catchment areas of Wuli and Sandu have been sensitised and approval from the Director of Health Services to proceed with the study in the North Bank of the URR has also been granted. The study nurses will be based at the following health facilities in the Nortn Bank of the URR: Yorrobawol Health Center, Darsilami Community Health Post, Konkuba Community Health Post and Taibatu Health Post. A team lead by the local PI (Clinical Trial Coordinator), the Research Clinician and Field Coordinator will tour all the communities to sensitise them on the study during April-early May 2017. The community sensitisation will involve village to village sensitisation and a sentisation program in Yorrobawol and Darsilami where satellite village elders, Alkalos and the

chiefs will be invited. The attached information sheet will be used as the basis for the sensitisations.

5.1.2 Screening and enrolment (baseline)

Young children in the participating communities will be identified using the enumeration/census data currently being collected by the study field team within the study catchment area (Figure 5). At screening, once mothers/guardians of the child have signed the informed consent form, the child will be physically examined by a study nurse and, if the child is considered as generally healthy (e.g. no fever, not severely malnourished), their height and weight will be measured and a finger prick blood sample will be collected for Hb and RDT testing at one of the health facilities supervised by the study nurses (Figure 4). If z-scores are >-3, 7 ≤Hb< 11 g/dL and the RDT is negative, then a small venous blood sample (1 mL in total divided into 0.5 ml EDTA and 0.5 ml serum blood collection tubes) will be collected to confirm the Hb levels (Medonic analyser) and determine serum ferritin (Cobas analyser). If 7 ≤Hb< 11 g/dL and serum ferritin < 30 ng/ml, the child will be eligible to enrol in the study. Children with Hb < 7 g/dL will not be enrolled and will be referred to the regional health centre for treatment according to national guidelines. Children with Hb ≥ 11 will not be enrolled as they don't need iron. Malaria positive children (positive RDT and confirmation by blood film) will not be enrolled and will be treated according to national guidelines. Severely malnourished children will also be excluded.

Screening will be divided into 3 cohorts (each recruitment period will be of approximately 1 month prior to enrolment into each of the sequential cohorts and we expect to screen ~ 500 children in each cohort or 50 children/day for 2 weeks, with 2 weeks left to get all screening results back from the lab.

Prior to enrolment (Day 0), which will be a maximum 4 weeks after the screening visit, eligible children will be taken back to the clinic for a finger prick to confirm absence of malaria by RDT and haemoglobin concentration by hemocue. Children will be called back in clusters of 58-60 on a Monday, Tuesday, Wednesday and Thursday. This is necessary before they can be enrolled and randomised to the study because of the time it takes to get all the screening results back from the labs for all children in that cohort (i.e. around 4 weeks). On the Friday of the same week, the list of all the children that remain RDT negative and with 7 ≤Hb< 11 g/dL will be sent to the study statistician who will run the randomisation to one of the three study arms, stratified by Hb and age as described in 3.2.1., and send the list with the study IDs to the PIs that same day.

The week after, children will be taken back to the clinic again in the same cluster (i.e. either on the Mond, Tue, Wed or Thur as per their Day 0 cluster) and this will be their study Day 1 (see below).

We will enrol children in 3 cohorts (n=235 children each) that will run sequentially.

Each study cohort will be organised as follows. Each cohort will have 4 clusters (58-60 children per cluster), children in each cluster will be allocated to one of the study health facilities according to the child's compound proximity to each of the 4 study health facilities. Children in the first cluster will all have their study visits on a Monday, children in the second cluster will all have their study visits on the Tuesday, and the third cluster on the Wednesday and fourth cluster on the Thursday. Children in each cluster will also have their weekly check-ups at the study health facility on the same day of the week and would have had their Day 0 visit on the same day of the week.

5.1.3 Follow-up (study visits)

Each arm will include an intervention period (follow-up) of 12 weeks plus an additional active follow-up period of 4 weeks post intervention. As suggested by our grant proposal reviewers, we have included a placebo arm to allow us to fully evaluate IHAT effects, particularly to rule-out negative impact on diarrhoea episodes, the gut microbiome and compound-related bacterial infection, and to determine true treatment effect sizes with both IHAT and ferrous sulphate (for more details refer to Section 11.1.1.).

On study Day 1, we will take a photo of the child (with consent) and print and laminate a study ID card that we will ask the mother to keep safe during the study. This ID card will contain the photo of the child and their randomisation/study ID number. We will also provide the child with a wrist band showing the same study ID number so that there is no confusion regarding the identity of the study participants. This was considered necessary in the context of the North Bank of the URR since there is a poor health record system in place and most children do not have welfare cards. Then, we will collect a venous blood (total of 5 ml divided into 1 ml EDTA, 1 ml LH and 3 ml serum collection tubes) and a stool sample from the children (baseline samples). Blood sample collection will be done either before the first meal of the day or, in cases where this is impossible, at least 1 hour after the last meal (so that the more dynamic iron parameters, such as hepcidin and NTBI, are not influenced by the iron absorption from the previous meal). Stool samples may be collected by the mother at home on the morning of the study visit using the toilet pots and disposable liners supplied by the study team for each child. The field worker will then aliquot the stool sample into the stool sample pots to be brought to the study clinic as soon as feasible after collection of the sample and will transport them to the clinic, when it is not possible to collect the sample from the child at the clinic.

On Day 1, we will also collect demographic and immunisation data and the morbidity questionnaire will be completed.

At the end of this visit, the mother will be encouraged to feed the child and once the child is settled, they will be given the iron supplementation or placebo. The treatment arm intervention

will be administered directly into the child's mouth homogenised in 10 ml of a sugar juice drink. The iron or placebo powder doses will be added to the small amount of juice in a disposable plastic cup immediately before administration, this will be done by the field worker. This first administration of the study supplement/placebo to a child will be supervised by the study nurse and/or the study clinician to offer extra reassurance to the mother.

Highly trained and experienced field workers will be visiting all children every day during the 12 weeks supplementation period in order to administer the iron supplements or placebo and on these occasions they will check on the children's general health and actively look for signs of malaria and co-infections. If a child shows signs of these infections, the field worker will refer to the study nurse who will perform adequate tests and the child will be offered the appropriate treatment/referral to the next health center. Prior to study start, all field workers will receive appropriate training, provided by the study nurses and supervised by the study clinicians, on which AEs and SAEs to look out for and on when to refer the children to the study nurses for follow-up. Three times per week, morbidity data (including questions regarding fever, diarrhoea, vomiting, cough, malaria symptoms, any other illness, appetite and any mediation taken and assessment of body temperature) will be captured (as part of the CRF). Every week children will be taken back to the health facility in their cluster for a check-up and a finger prick to determine their malaria and Hb status. Hb will be determined with HemoCue. RDT testing will be used for malaria and children found with a positive RDT during the study will be further tested with a blood film and treated according to national guidelines. These check-ups will continue 4 weeks post intervention to follow-up on AE/SAEs.

Each field assistant (FA) will be responsible for 10-12 children within each cohort of 235 children, and will be supported by senior field assistants (SFA) and field supervisors (FS). The study will have a field team comprising of 21 FA, 4 SFA and 2 FS. All will be managed by the Field Coordinator (FC). Each SFA will be responsible for supervising the FA working in the study cluster comprising 58-60 children. The FS will each be responsible for the communities in the Wuli and Sandu districts, respectively.

Every day during the intervention period of 12 weeks, the treatment arm interventions will be administered directly into the child's mouth homogenised in 10 ml of a sugar juice drink. The iron or placebo powder doses will be added to the small amount of juice in a disposable plastic cup immediately before administration, this will be done by the field worker. Before supplementation, the mother will be encouraged to feed the child so that the supplaments are not ingested on an empty stomach (as detailed in 3.4.4).

On Days 15 and 85, the child will visit the clinic and another stool sample and another venous blood sample (total of 5 ml divided into 1 ml EDTA, 1 ml LH and 3 ml serum collection tubes) will be collected. Height and weight will be measured at day 85.

Any child where Hb falls below 7 g/dL during the follow-up study period will stop the study supplementation and will be referred to the next health centre for management and will be provided with iron supplements for 3 consecutive months according to national and WHO guidelines. These children will be treated in the data analysis as treatment failures. These children will continue to be followed up for adverse events by the field team but not receive any of the study treatments.

At each study visit (i.e. day 1, 15, 85) we will check the child's welfare card or ask the mother and note in the CRF if there was a vaccine in the past week, since some of the acute inflammatory markers we are measuring, particularly CRP, can transiently increase after immunisation (42, 43).

At the end of the follow-up period (12+4 weeks), the children in any arm who still have anaemia (Hb<11 g/dLl) will be provided with iron supplementation for 3 consecutive months as per national and WHO guidelines.

5.1.4 Final study visit

The final study visit will be on day 113 (in the post-intervention follow-up period) when a finger prick blood sample will be collected and those children that remain anaemic will be offered iron supplementation for 3 consecutive months according to the national and WHO guidelines.

5.1.5 Early termination visit

An early termination visit may occur in this study because of a participant's voluntary withdrawal, trial team decision or at the discretion of the Data Safety Monitoring Board as described in Section 7. Apart from the safety evaluations, no other evaluations required for the final study visit will be done.

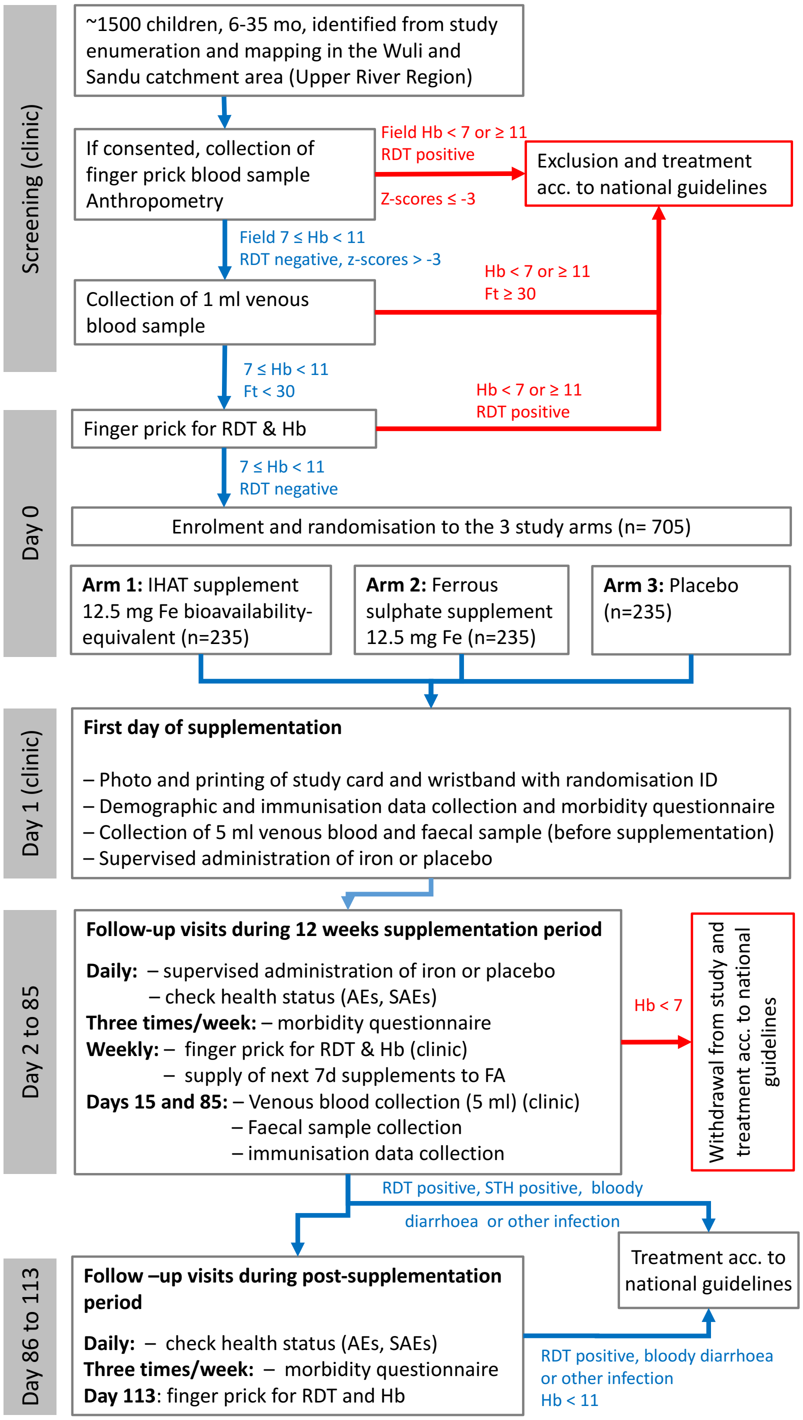

Figure 5. Study Flow Chart. Abbreviations: Hb, haemoglobin; Ft, ferritin; RDT, rapid diagnostics test; STH, soil-transmitted helminths; AE, adverse events; SAEs, serious adverse events; FA, field assistant; Fe, iron.

5.2 Study evaluations

This trial is powered to test non-inferiority of IHAT in relation to ferrous sulphate at correcting IDA, and in terms of diarrhoea to show superiority in relation to ferrous sulphate and non-inferiority in relation to placebo.

Non-inferiority of IHAT in relation to $FeSO_4$ at correcting IDA will be based on the response probability in the IHAT and $FeSO_4$ arms at the Day 85 time-point, i.e. the proportion of children in each arm that resolve iron deficiency and achieve a normal Hb or an increase of at least 1 g/dL in Hb after 12 weeks of the intervention.

Superiority of IHAT in relation to $FeSO_4$ in terms of diarrhoea will be based in both 'incidence density', i.e. the mean number of new moderate-severe diarrhoea episodes per child over the 12 weeks intervention period, and 'period prevalence', i.e. the proportion of children with at least one episode of moderate-severe diarrhoea over the 12 weeks intervention period, of moderate-severe diarrhoea.

Secondary objectives are to show that IHAT is non-detrimental with respect to enteric pathogen burden, the gut microbiome, and intestinal inflammation.

Please refer to Section 8 for more details on this analysis and power of the trial.

5.2.1 Clinical evaluations

Health status of the children at enrolment will be assessed through a physical examination and blood screening results by a study nurse in consultation with the study clinicians. During the study period, diarrhoea, hospitalisation and morbidity data will be collected three times per week in the field using a morbidity questionnaire.

5.2.2 Laboratory evaluations

Blood samples:

The analysis bellow will be performed in the ***venous blood samples*** collected on study days 1 (baseline), 15 and 85 and blood will be taken at the health facilities in the catchment area. Sample collection will be either before the first meal of the day or, in cases where this is impossible, at least 1 hour after the last meal (so that the more dynamic iron parameters, such as hepcidin and NTBI, are not influenced by the iron absorption from the previous meal). Blood will be transported to the MRC Basse laboratory for processing/analysis as soon as possible after collection.

Haemoglobin will be determined in EDTA anticoagulated blood (~500 µl) as part of a full haematology panel using the Medonic Haematology Analyser at MRC Basse (the MRCG Quality team is currently working towards setting up the validation of the Medonic assay in Basse).

Four serum aliquots will be frozen (-20°C) at MRC Basse on the day of blood collection for the following analysis. One aliquot (500 µl) where serum ferritin, sTfR (soluble transferrin receptor), serum iron & total iron binding capacity (for calculation of transferrin saturation), CRP (C-reactive protein) and AGP (alpha-1-acid glycoprotein) will be determined using the fully automated biochemistry analyser (Cobas Integra 400 plus) at MRC Keneba (the MRCG Quality team is currently working towards setting up the validation of the iron panel assays in Keneba).

Serum hepcidin will be measured in another aliquot (200 µl) using the DRG® Hepcidin 25 (bioactive) HS ELISA test kit and the Thermo Multiskan FC Microplate Photometer at MRC Keneba.

Serum NTBI (aliquot of 300 µl) will be measured at King's College London (UK) by the new fluorescent beads method (44) that correlates closely with the traditional NTA method at lower TSAT but offers a crucial advantage over the NTA method because this can overestimate NTBI since NTA can remove some Fe bound to transferrin, particularly at TSAT above 70% (45, 46).

One serum aliquot (500 µl) and one plasma aliquot (500 µl) will be kept stored at -70°C for future analysis, particularly as new biomarkers of adverse effects due to iron emerge. This was a request from the funders. This may include DNA analysis and export of samples. We will obtain informed consent from the mothers/guardians for this to be the case within the study informed consent. Any future use would require PI, MRCG SCC and EC approval. This medium-long term storage will most likely be at MRC Fajara.

In the ***finger prick blood samples***, Hb will be determined using a HemoCue 301 (5 µl) and an RDT (5 µl) will be performed every week at one of the study health facilities. In case of a positive RDT, a blood film (20 µl) will be prepared and read at the MRC laboratory in Basse.

Stool samples:

Stool samples (~20 g) will be collected in toilet pots provided by the study team and lined with a disposable plastic liner at study days 1, 15 and 85 either at the participant's home on the morning of the study visit or at the health facilities where the venous blood sample will be

collected. The samples will be kept in the toilet pots covered with the plastic liner in a cool place until the field worker process them. The field worker will aliquot the sample into the two labelled sterile stool sample tubes with scoop and screw lid as soon as possible after the child passes the stool. Approximately 15g of stool will be placed into a sterile Sarstedt stool collection tube and 5g of stool into an OMNIgene GUT sample collection kit tube containing a DNA preservative that ensures that samples for microbiome analysis can be kept at room temperature. The field workers will avoid soil contamination and cross-contamination of the samples at all times. The samples will then be transported to the MRC Basse laboratory. Once samples arrive at the MRC Basse, the Sarstedt sample will be further divided into three aliquots: one aliquot will be used for helminth egg count and will be kept refrigerated until processing (~ 5 g) and the remaining two aliquots (~5 g each) will be frozen at -70°C, within 24 hours of collection. The OMNIgene sample will be kept at room temperature in the lab at MRC Basse until DNA extraction, which should be done within 8 weeks for all samples.

The national policy for anti-helminth treatment in The Gambia is that every child should receive de-worming tablets every 6 months, however, we still expect a proportion of the study population to be infected with soil-transmitted helminths (STH). Since STH infection can affect iron absorption and impact body iron status in young children (47, 48), we will perform a microscopic examination of a small sample of stool (sample kept refrigerated, not frozen) to determine the presence and number of STH eggs using the current WHO recommended Kato-Katz method, based on duplicate slides (49). Sample aliquots for this analysis will not be frozen but will be kept refrigerated at 4°C for up to 3 weeks as this has been shown to not cause significant changes in the egg counts and the morphology of eggs. Those children who test positive for STH infection (assessed on study days 1, 15 and 85) will be provided with anti-helminth treatment according to national guidelines but will not be excluded from the study.

The second Sarstedt sample aliquot (5 g) will be used for calprotectin analysis using the Calprotectin ELISA (EK-CAL, Bühlmann Laboratories). This analysis will be performed at the Basse Lab.

An additional aliquot (5 g) will remain frozen at -70°C for future analysis. This medium-long term storage will most likely be at MRC Fajara.

The DNA will be extracted from the OMNIgene aliquot sample using the MoBio Soil extraction kit at the MRC Basse laboratory. Faecal DNA will be kept frozen at -70°C until it is sent to the Sanger Institute in Cambridge (UK) for microbiome analysis at the end of the study. Microbiome analysis will be carried out by 16S rRNA sequencing (Illumina MiSeq). Minimum entropy decomposition (50) and targeted qPCR will be used to probe specifically the enteric pathogens. A study scientific officer will be trained in this analysis at the Sanger to help with the PCR lab work load.

Finally, for those children presenting with bloody diarrhoea episodes, an additional stool sample will be collected at that point and we will use stool microbiology, at the Basse laboratory, and the TaqMan Array Card (TAC) system (41) (same used in the GEMS study), at the Fajara laboratory, to identify the causative infectious agent.

6 Safety considerations

A trial steering committee (TSC) has been setup in discussion with the Sponsor. Membership of this committee includes the co-PI (Dr Pereira), the MRC BSU biostatistician advisor (Dr James Wason), an independent Chair (Dr Margaret Pinder, MRCG Basse), a sponsor representative (Mr Pa Cheboh, Head of Operations at MRCG Basse), a community representative (local teacher), and the local PI (Dr Mohammad Ilias Hossain, MRCG Basse).

This trial will be overseen by a Data Safety Monitoring Board (DSMB) (the chair of this committee is Professor James Jay Berkley of the KEMRI-Wellcome Kilifi Institute)). The DSMB will be responsible for reviewing:

- the trial protocol (before the trial is started)
- all interim data from the trial
- treatment safety and efficacy including the protection of the rights and well-being of the participants
- the overall progress of the study

The DSMB will additionally review all Serious Adverse Events (SAEs).

In addition to the DSMB, an independent Local Safety Monitor will regularly review all AEs and SAEs. This review will focus particularly on AEs causality and reasons for losses to follow up, raising any concerns or issues that present immediate safety concern with the PIs for reporting to the DSMB, while protecting the confidentiality of the trial data and the results of monitoring.

6.1 Methods and timing for assessing, recording, and analysing safety parameters

The trial will be conducted according to Good Clinical Practice (GCP) principles. The DSMB will determine how they will monitor the data and safety interest of the participants. The DSMB will

also determine how and the frequency of its meetings but we propose that the DSMB meets at least 3 times during the study intervention period to review AE/SAE rates in each arm and to have a concluding meeting towards the end of the study period. SAEs will be sent in real-time to the DSMB. The DSMB's responsibilities will be clearly defined in the DSMB charter, which will include how real-time SAE submissions are to be handled, i.e. mechanisims that would trigger ad-hoc meetings, etc. The DSMB charter has been prepared by the co-PI with Dr Jonas Lexow and has been reviewed by the DSMB Chair.

6.1.1 Adverse events

An adverse event (AE) is defined as any untoward or unfavourable medical occurrence in a human subject, including signs and symptoms which are temporally associated with the subject's participation in the research, whether or not considered related to the subject's participation in the research. Participants will be monitored for AEs on each scheduled follow up day. All symptoms or signs reported or observed will be assessed by the study Field Assistant and will be recorded as an AE after evaluation by the study nurse. Persistently low Hb will be considered as an AE and will be followed up.

Diarrhoea, defined as 3 or more loose or water stools per day will NOT be considered as an AE (but will be recorded as part of the morbidity questionnaires), unles it requires treatment. However, moderate-severe diarrhoea, referring to those diarrhoea episodes where (i) the child passes 5 or more loose or watery stools per day, (ii) there is blood in the stool (dysentery), OR (iii) the child shows signs of clinical dehydration (assessed by the study nurse based on physical signs such as little or no urination, sunken eyes, and skin that lacks its normal elasticity), will usually require treatment (including ORS) and will be recorded as an AE.

6.1.2 Reactogenicity

N/A

6.1.3 Serious adverse events (SAEs)

A SAE is any AE that is life-threatening or results in death or requires hospitalisation or prolongation of hospitalisation or is a persistent or significant disability/incapacity. All SAEs will be investigated by the study clinicians.

6.1.4 Assessment of intensity of AEs

The study clinicians, i.e. the research clinician and the local PI (paediatric clinician and Clinical Trial Coordinator) with support from the clinical team in Basse, will assess the severity and intensity of the AEs and laboratory changes as defined below and record it into the AE form.
The local safety monitor is also a paediatric clinician independent from the research team and her primary responsibility will be to review individual and cumulative AEs and report to the local PI (Clinical Trial Coordinator).

Grade		Description
1	Mild	Awareness of sign or symptom, but easily tolerated
2	Moderate	Enough discomfort to cause interference with usual activity
3	Severe	Incapacitating with inability to work or do usual activity
4	Life-threatening	This grade will be considered as SAE

The term "severe" is often used to describe the intensity (severity) of a specific event (as in mild, moderate, or severe myocardial infarction); the event itself, however, may be of relatively minor medical significance (such as severe headache). This is not the same as "serious", which is based on the outcome or criteria defined under the SAE definition. An event can be considered serious without being severe if it conforms to the seriousness criteria; similarly severe events that do not conform to the criteria are not necessarily serious. Seriousness (not severity) serves as a guide for defining regulatory reporting obligations.

6.1.5 Assessment of causality

Every effort will be made by the study clinicians (the research clinician and the local PI), in discussion with the clinical team in Basse, and the co-PI (Dr Pereira), to explain each AE and assess its causal relationship to administration of the investigational product. This explanation will be based on the type of event, the relationship of the event to the time of trial intervention, and the natural history of the underlying diseases, concomitant therapy, etc. The results will be documented on the AE form. The relationship of an AE to the investigational product will be assessed according to the definitions in the MRC Unit's SOP:

Unrelated

No temporal association with the study supplementation/drug; related to other aetiologies such as concomitant medications or conditions, or participant's known clinical state.

Unlikely

Temporal association with the study supplementation is improbable, but not impossible; other aetiologies such as concomitant medications or conditions, or participant's known clinical state provide plausible explanations.

Possible

Less clear temporal association; event could also be explained by alternate aetiology (clinical state, environmental or other interventions).

Probable

Clear-cut temporal association, with improvement upon drug withdrawal and not reasonably explained by alternate aetiologies (patient's known clinical state, environmental, or other interventions).

Definite

Clear-cut temporal association, with a positive re-challenge test or laboratory confirmation.

The mothers/guardians of participating children will be instructed to contact the field assistant or a member of the study team, should the child manifest any signs or symptoms they perceive as severe during the period extending from performance of the first trial procedure to the end of the study.

All findings observed or reported from the day of the first administration of the investigational product will be recorded on an AE Form electronically by the team. Whenever possible, AEs will be documented in terms of a diagnosis or syndrome rather than multiple symptoms that are clear manifestations of the same diagnosis/syndrome. In case signs and symptoms are reported by the participants, a medical diagnosis will be obtained by the study clinicians. If a diagnosis cannot be obtained then each sign or symptom will be recorded as separate events.

The action taken (e.g. discontinuation of investigational product, withdrawal of the participant from the trial, requirement of concomitant medication or treatment, others) will be recorded on the AE Form. If hospitalisation or its prolongation is required this will be reported as a SAE.

All AEs will be followed until resolution of the event and/or the end of the trial. The outcome will be assessed as follows:

- Resolved
- Resolved with sequelae
- Ongoing
- Death
- Lost to follow up

Treatment of any AE and SAE will be recorded in the appropriate section of the CRF.

6.1.6 Serious Adverse Reaction (SAR)

SAR is any SAE deemed to be **probably related or definitely related** to the study supplementation (as defined above).

Trial recruitment will be stopped with immediate effect if any child has a SAR to any of the iron supplements (or placebo) until review by the DSMB. Follow-up of children already enrolled in the study will continue as planned pending the review of the DSMB, as indicated in the DSMB Charter.

6.2 Reporting procedures

The Local Safety Monitor will prepare regular reports concerning AEs and SAEs for submission to the local PI. The local PI (Clinical Trial Coordinator), shall report all SAEs (defined as in 6.1.3) without filtration, whether or not related to the trial intervention, within 24 hours of becoming aware of the event to the DSMB, the Sponsor, and the MRC CTSO (dct@mrc.gm). If the SAE is related to the trial intervention, the Ethics Committee will be notified within 7 calendar days if fatal or life-threatening, and all others within 15 calendar days.
All SAE will be reported to the Medicines Control Agency in accordance with the applicable requirement.

The minimum information required for this initial SAE report is:

- Trial number and (short) title
- Participant's ID
- Date and time of onset
- Description of the event (clinical history, associated signs and symptoms)
- Reporter's name

The information about which intervention was administered (unblinding) will only be provided at the request of the DSMB.

The local PI will not wait for additional information to fully document the event before notifying. The report is then to be followed by submission of a completed SAE Report Form as soon as possible, detailing relevant aspects of the SAE in question. All actions taken by the local PI and the outcome of the event must also be reported immediately.

For documentation of the SAE, any actions taken, outcome and follow-up, the SAE Report Forms will be used. All follow-up activities have to be reported, if necessary on one or more consecutive SAE report forms in a timely manner. All fields with additional or changed information must be completed and the report form should be forwarded to the DSMB within 5 calendar days after receipt of new information. Hospital case records and autopsy reports, including verbal autopsy, will be obtained where applicable.

6.3 Safety oversight

Safety oversight shall be provided by a DSMB who will provide independent advice as stipulated in Section 6 above. An independent Local Safety Monitor will review all adverse events more regularly and report regularly to the PIs and DSMB.

7 Discontinuation criteria

7.1 Participant’s premature termination

Mothers/guardians of participating children have the right to stop their children’s participation in the study at any time without giving a reason and this will not affect the medical care that would normally be received. The trial team may also withdraw a participant from the study if deemed necessary at any time taking in to consideration the reasons mentioned below. The reason for a participant’s premature termination will be documented on the appropriate page of the CRF and specified which of the following possible reasons were responsible for the premature termination:

- Serious Adverse Event
- Adverse Event
- Participant’s consent withdrawal
- Development of withdrawal criterion

- Protocol deviation
- Migrated/moved from the study area
- Lost to follow-up

A 'lost to follow-up' is any participant who completed all protocol specific procedures up to the administration of the investigational product or intervention, but was then lost during the study period to any further follow-up, with no safety information and no efficacy endpoint data ever became available.

In case the participant decides to withdraw participation or consent during the study, we will not work on participant’s samples without permission, but any information already generated from the samples will be kept and used. The study clinician may also ask for tests for the participant’s safety. The PI will inquire about the reason for any withdrawal and follow-up with the participant regarding any unresolved AEs.

For withdrawn participants no specific data will be collected. Our sample size takes into account a dropout rate of 15% but this will be monitored closely and if the observed dropout rate increases above this level we will replace subjects to ensure we reach our target sample size of n=600 completed.

7.2 Study discontinuation

The rules for study termination will be set by the DSMB at their first meeting.

8 Statistical considerations

8.1 Sample size determination

This section was prepared with the expert input of Dr James Wason, MRC Biostatistics Unit, Cambridge. Dr James Wason is a Programme Leader Track senior statistician who primarily works on statistical methodology for novel clinical trial designs and is one of the few UK experts in design and analysis of adaptive clinical trials. Dr Wason is a long term collaborator of Dr Pereira for the design of the IHAT trials and is a collaborator in the proposed trial. He has provided advice on the trial design and conducted the trial power modelling and sample size calculations presented in this Section. Dr Wason will continue to provide advice on the trial design to the PIs and will supervise an MRCG statistician in the data analysis corresponding to the four trial hypothesis (below). Dr Wason will be a member of the TSC and will review and approve the data analysis plan for the trial, but it will be the responsibility of the MRCG statistician (Dr Nuredin Mohammed supervised by Dr David Jeffries) to develop the initial drafts of the data analysis plan together with the study PIs.

There are four null hypotheses in the study:

1) non-inferiority of IHAT compared to ferrous sulphate for efficacy (in terms of Hb and iron deficiency correction: i.e. IDA) response probability (or prevalence). In this case we are testing the null hypothesis that: response in the IHAT arm minus the response probability in the ferrous sulphate arm is less than or equal to – 0.1. We define 'response' for IDA as correction of iron deficiency and either achieving a normal Hb or an increase of at least 1 g/dL after 12 weeks of iron supplementation.

2) superiority of IHAT compared to ferrous sulphate in terms of incidence density of diarrhoea. Here the null hypothesis is that the mean number of new episodes in the IHAT arm is greater than or equal to the mean number in the $FeSO_4$ arm.

3) superiority of IHAT compared to ferrous sulphate in terms of prevalence of diarrhoea. Here the null hypothesis is that the prevalence of diarrhoea in the IHAT arm is greater than or equal to the prevalence in the $FeSO_4$ arm.

4) non-inferiority of IHAT compared to placebo in terms of prevalence of diarrhoea. Here the null hypothesis is that the prevalence of diarrhoea in the placebo arm minus the prevalence in the IHAT arm is less than or equal – 0.1.

Because this is a pilot study we do not formally adjust for multiple testing due to the four hypotheses. This is because any significant results will be tested again in a pivotal study.

The trial is powered to show non-inferiority of IHAT relative to ferrous sulphate for the 12 weeks efficacy response probability. At the 12 weeks time-point (i.e. Day 85), the estimated odds ratio between the IHAT and $FeSO_4$ arms will be calculated, together with a 90% one-sided confidence interval. If the lower limit of the confidence interval for this difference is above 0.583 (equivalent to a 10% absolute difference if $FeSO_4$ response rate is 0.3), then non-inferiority of IHAT in terms of efficacy will be declared.

Because many trials conducted in Africa and other developing countries with the gold standard ferrous sulphate or ferrous fumarate supplementation still fail to provide anaemia resolution in interventions shorter than 6 months, we have considered here that an increase of 1 g/dl in Hb after 3 months is an indication of efficacy, even though we accept that this is not clinical efficacy. We have based our assay sensitivity from data published with relevant iron supplement studies in children available in the literature (3, 7, 52). We plan, however, to use the data collected in the proposed study to conduct an exploratory analysis to test for assay sensitivity. These data will be very useful so that adequate treatment effect sizes can be used to power any future studies, in particular a pivotal trial, both for ferrous sulphate and IHAT supplementation effects.

Due to this trial being a pilot study, any significant results will be re-tested in the pivotal 'full' trial (BMGF full award). Thus, we considered a larger than traditional type-I error rate of 10%. Assuming that 30% (i.e. a response probability of 0.3) of the children in both arms will resolve iron deficiency and either achieve a normal Hb or an increase of at least 1 g/dL after 12 weeks of iron supplementation (3, 7, 52), then a sample size of 200 in the IHAT arm and 200 in the ferrous sulphate arm will provide 89% power to show non-inferiority with a non-inferiority margin of 0.1 (0.583 on the odds-ratio scale). In other words, the trial will have 89% power (at a 10% one-sided significance level) to show that the lower boundary of the confidence interval of the odds ratio between IHAT and ferrous sulphate is more than 0.583 at 12 weeks.

We have based our calculation above on assay sensitivity data taken from other studies with iron supplements reported in the literature, but we will also perform an exploratory analysis to test for assay sensitivity, and this information will be used to power the potential future pivotal study.

We have considered two options for modelling the diarrhoea outcome: 1) 'period prevalence', i.e. the proportion of children with at least one episode of moderate-severe diarrhoea over the 12 weeks intervention period; 2) 'incidence density'. i.e. the number of new diarrhoea episodes per child over the 12 weeks intervention period. The modelling of how the power of the trial varies according to assumptions for each of these outcomes is detailed below.

The trial sample size will also provide 90% power to show superiority of IHAT in terms of moderate-severe diarrhoea 'incidence density', assuming that IHAT provides a 20% reduction in mean moderate-severe diarrhoea 'incidence density' relative to ferrous sulphate; and 90% power to show superiority of IHAT in terms of 'period prevalence' of moderate-severe diarrhoea, assuming that 25% of children will develop moderate-severe diarrhoea episodes with ferrous sulphate and 15% will have moderate-severe diarrhoea with IHAT over the 12 weeks intervention. For the comparison between the IHAT and placebo arms, the trial is powered to show non-inferiority of IHAT relative to placebo, for example, the trial will have 93% power for the moderate-severe diarrhoea 'period prevalence' outcome. All the assumptions for these calculations were based on data from studies conducted in The Gambia for the same age group, please refer to the extra information below for further details.

As part of the rationale for the study is to provide information for a pivotal study, we also examined the precision that the sample size would provide on the confidence interval width of the outcomes considered. Since the width of the confidence interval is highly dependent on the proportion in the control group as well as the true odds ratio, we consider several different values in the table below.

Binomial proportion in control group	True odds-ratio	95% confidence interval width
0.1	0.5	0.85
	1	1.38
	1.5	1.91
0.3	0.5	0.49
	1	0.88
	1.5	1.27
0.5	0.5	0.42
	1	0.80
	1.5	1.21

The power calculations for some of the secondary outcomes are also presented in more detail below. Briefly, the trial (n=200 per arm) will have over 85% power to detect significant differences between all the arms in terms of Enterobacteria, NTBI and calprotectin.

Taking into consideration a non-completion rate of 15% (this is based on data from previous studies in The Gambia), we expect to **enrol 705 children in the study**.

Detailed calculations of power for the diarrhoea and secondary outcomes:

I. POWER OF THE TRIAL FOR THE DIARRHEAL OUTCOME.

1. Period Prevalence (i.e. proportion of children with >= 1 episode of moderate-severe diarrhoea over the 12 weeks intervention period)

Based on previous data from the case-control GEMS (Global Enteric Multicentre Study) for the Basse (Upper River BHDSS) region in the Gambia, the background point prevalence of moderate-severe diarrhoea in children under 3 years of age can be as high as 14%, whereas data from the same region in a previous survey HUAS (Health Care Utilization and Attitudes Survey) showed that this number can be as high as 20%.

For our calculations, we chose to consider 15% as the proportion of children with at least one episode of diarrhoea in the IHAT group over the 12 wk. intervention period, which we hypothesise would be similar to the background period prevalence. This prevalence is similar to that found in other iron intervention studies for the no-iron groups (7, 8).

The graph below shows the power of the trial (200 patients per arm at a 10% one-sided type I error rate) to detect a significant difference between IHAT and ferrous sulphate (FS) as the ferrous sulphate diarrhoea period-prevalence varies:

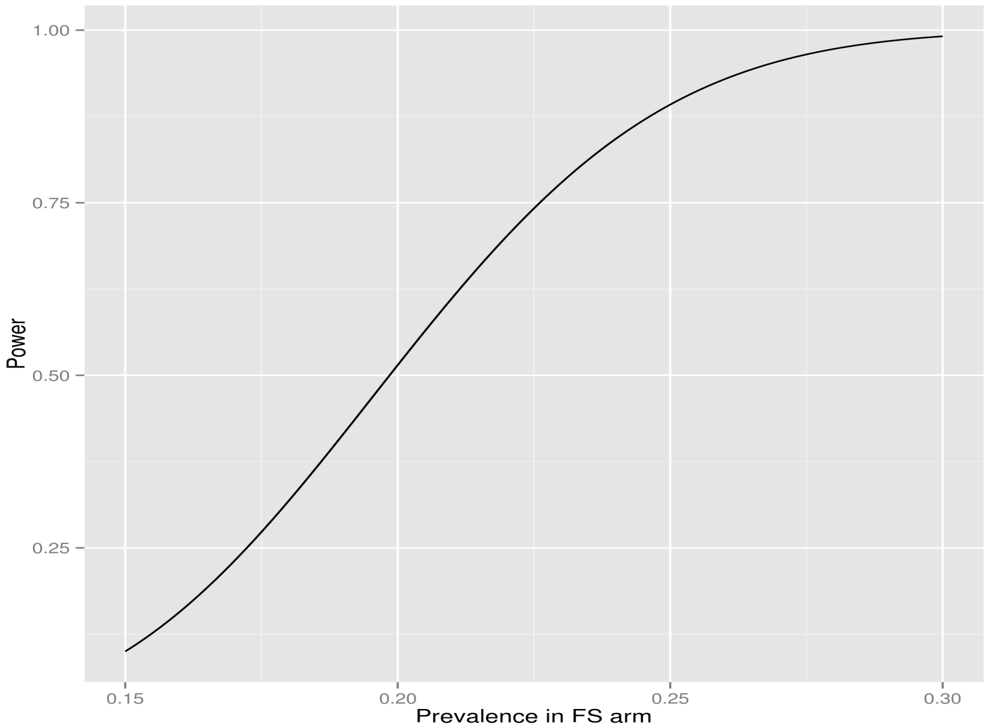

For a period prevalence of 0.15 in the IHAT arm and 0.25 in the ferrous sulphate arm, the power of the trial is around 90%. We note that previous data for ferrous sulphate suggest that 25% is a reasonable figure for the period prevalence: for example Zlotkin et al 2013 reported this to be 16.8% in the iron group and Jaeggi et al 2014 reported 27.3%. Furthermore, for the Gambia specifically, preliminary data from the 84 days period of the current HIGH study in the Soma region shows that, during the 84-days of the intervention, moderate-severe diarrhoea was recorded at least once as an adverse event in 36% of the children.

For comparing placebo and IHAT, we used a non-inferiority approach. With 200 patients per arm, we would have 93% power to show non-inferiority of IHAT with a 10% non-inferiority

margin (at a 10% one-sided type I error rate). This means that, if the true diarrhoea period prevalence in the IHAT and placebo arms is 0.15, this sample size has 93% power to show that the upper confidence interval of the difference in prevalence between these two arms is less than 10%.

<u>2. Incidence density (i.e. number of moderate-severe diarrhoea episodes per child over the 12 weeks intervention)</u>

Preliminary data from the 84 days data collected in the HIGH study in the Soma region in the Gambia suggest that the incidence density of moderate-severe diarrhoea is 1.28 episodes per child over the 84-days study period. Using the same type I error rate and sample size as above, the graph below shows the power of the trial to detect a significant difference between arms as the rate ratio varies.

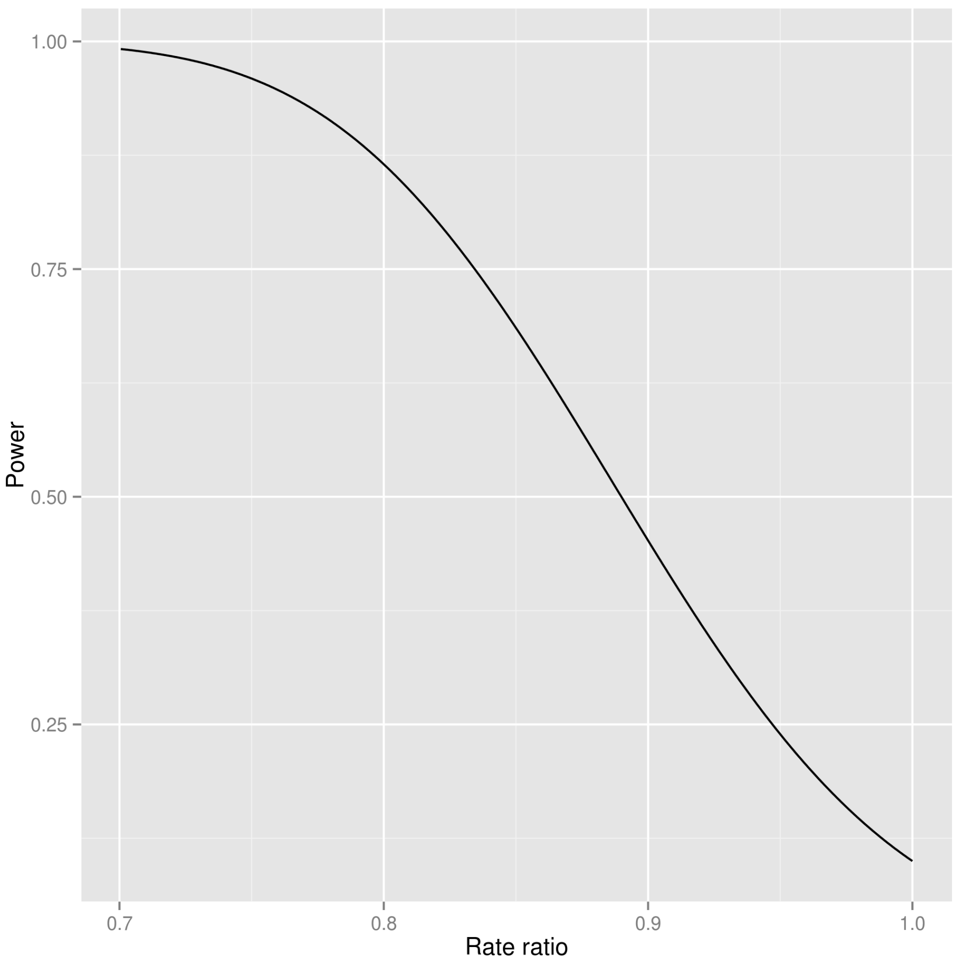

For a rate ratio of 0.8 (i.e. IHAT provides a 20% reduction in mean incidence density of moderate-severe diarrhoea relative to ferrous sulphate), the power is almost 90%.

II. POWER of the trial for the secondary endpoints

1) Microbiome (Enterobacteria log10 counts):

We based our calculation on the data for Enterobacteria (the genus that contains most enteropathogens including *E. coli*) reported in Zimmermann 2014 study conducted with 4 months supplementation (8): with iron 8.9 (0.3) and without iron 8.0 (0.4). Taking into consideration that in this study the analysis combined the groups with 2.5 mg Fe and 12.5 mg Fe MNP, then the combined sample size implies an individual standard deviation of 3 for the Enterobacteria and assuming a 0.9 difference between the groups and a superiority margin of 0.2 (i.e. an absolute difference in Enterobacteria between the IHAT and ferrous sulphate arms of - 0.2), the sample size of 200 children per group would give us 85% (at 10% one-sided significance level).

2) Calprotectin

Again, we based this calculation in the Zimmermann 2014 study (8). Calprotectin concentration with iron: 248.9 +/- 2.2 μg/g and without iron: 102.5 +/- 2.2 μg/g in the 12.5 mg MNP. Here the individual SD should be around 11.8 considering the study sample size in the 12.5mg Fe MNP group, which would mean that with 200 per arm we could detect an absolute mean difference of 3 with 90% power (10% one-sided sig level) and with 50 per arm we would be able to detect a mean difference of 6.1.

3) NTBI

For this calculation we have used the 'normal serum' data provided by Robert Hider with the same method we intend to use in the study (44). Taking the values reported in the paper for 'normal serum' of 0.24 +/- 0.15 (mean +/- SD), n=9 range 0.04 – 0.41, the individual standard deviation would be 0.45. There is no published data with this method for NTBI after an iron dose so we have assumed we would like to detect a mean difference of 0.5 between the IHAT and the ferrous sulphate arm, which would mean that with 50 per arm the power would be more than

90%. To be able to detect smaller differences with this method we would need to increase sample size.

8.2 Statistical analysis

The data analysis plan will be fully developed prior to commencing trial recruitment and will be done with the expert input from Dr James Wason of the MRC Biostatistics Unit in Cambridge. Briefly, for the primary analysis, we will fit a logistic regression to the efficacy outcome data, adjusting for the strata created by age and Hb level groups prior to enrolment. The odds ratio for effect of IHAT relative to ferrous sulphate will be estimated, and if the lower 90% one-sided confidence interval is above 0.583 (equivalent to the 10% non-inferiority margin above), non-inferiority will be declared for IHAT. The diarrhoea endpoint will be analysed in a similar way, but tested for superiority (i.e. if the one-sided p-value for the Wald test of the effect of IHAT is less than 0.1) for the comparison between IHAT and ferrous sulphate. For the primary non-inferiority hypothesis we will conduct per-protocol analyses and for the superiority hypotheses we will conduct intention-to-treat analyses.

The secondary endpoints (calprotectin, NTBI etc) will be tested using linear regression with the same covariates. Morbidity data will be analysed using multiple regression analysis controlling for possible confounders.

In relation to the microbiome data analysis we will work together with our collaborators in the Sanger Institute and use the most up to date bioinformatics methodology available at the time of data analysis to investigate the impact of treatment arm and time in the composition of the gut microbiome. Since this analysis will only occur in late 2017, it is premature to decide the strategy for data analysis now as this is a fast moving field with new and better bioinformatics tools becoming available every couple of months. The Sanger Institute works very closely with the European Bioinformatics Institute (EBI) (housed in the same campus) to ensure they use the very latest tools in their microbiome analysis.

9 Data handling and record keeping

9.1 Data management and processing

All protocol data will be captured in Case Report Forms (CRF) that will be completed for each included participant using electronic data capture. On the CRF, a reference to the source document will be provided. Instructions for completing all forms, including the CRF, used in the study will be developed.

The following data will be recorded: date of informed consent, personal data (ID, initials, date of birth), socioeconomic data, height and weight, information on health status and regarding participation in other studies, date and time of all venous and finger prick blood collections, date and time of all faecal sample collections, date and time of supplement administration, data on morbidity, lab results.

All trial data will be stored and managed within a clinical database built on the OpenClinica Enterprise platform, an application specifically designed to collect and store clinical trial data and customised for Electronic Data Capture (EDC) in the field. Where appropriate, data entry fields will incorporate appropriate range checks. At the point of data collection, data will be entered directly into tablet computers as already done in the MRC Gambia studies, and uploaded to the main server either immediately, or at least daily when network signal is weak. Data export options from the clinical database include CDISC ODM XML, a vendor neutral, platform independent format for interchange and archive of data collected in clinical trials. This should ensure sharing and long term validity of data. This data management system is a fully supported, externally validated (GCP compliant to 21 CFR part 11) clinical database.

Prior to the first participant enrolment a detailed trial specific data management plan (t_DMP) will be created, reviewed and signed off by the Data Manager and the TSC. This t-DMP will outline in detail the specific procedures that will be used to ensure high quality data will be delivered for statistical analysis and future use. The t-DMP will complement the trial risk assessment and statistical analysis plan. Review of data collection tools against the protocol will occur prior to database build by the Data Manager to ensure all data is being captured, at the correct time points, and in a format conducive to the proposed statistical analysis. Site based staff will be fully trained in conducting any study assessments and subsequent completion of the CRF and a Site delegation log will be maintained. Data entry staff will be trained in the use of OpenClinica. Principles of good clinical practice will be adhered to and all training will be documented. To ensure standardisation of processes, standard operating procedures with respect to trial

management, quality assurance, data management, IT & security, and statistics will be adhered to.

9.2 Source documents and access to source data

The local PI will maintain appropriate medical and research records for this study in compliance with the principles of good clinical practice and regulatory and institutional requirements for the protection of confidentiality of participants. The study team members will have access to records.

The authorised representatives of the sponsor, the ethics committee(s) or regulatory bodies may inspect all documents and records required to be maintained by the investigator, including but not limited to, medical records (office, clinic, or hospital) for the participants in this study. The clinical study site will permit access to such records.

9.3 Protocol deviations

A protocol deviation (PD) is any noncompliance with the clinical trial protocol, good clinical practice (GCP), or other applicable regulatory requirements. The noncompliance may be either on the part of the participant or the investigator including the study team members, and may result in significant added risk to the study participant. As a result of a deviation, corrective actions will be developed and implemented promptly.

If a deviation from, or a change of, the protocol is implemented to eliminate an immediate hazard(s) to trial participant without prior ethics approval, the PI or designee will submit the implemented deviation or change, the reasons for it, and, if appropriate, the proposed protocol amendment(s) as soon as possible to the sponsor for agreement and the relevant independent ethics committee (IEC) for review and approval.

The local PI or designee will document and explain any deviation from the approved protocol on the CRF, where appropriate, and record and explain any deviation according to the Unit's SOP.

10 QUALITY CONTROL AND QUALITY ASSURANCE

Quality control will be applied to each stage of the study. We will work together with the Sponsor, through their Quality Management team (Mrs *Yai Louise Ndure-Bensouda*) and Research Governance teams (led by Dr Jonas Lexow) to finalise a plan for the trial quality monitoring.

It will be the responsibility of the local PI or designated trial team member to ensure that all source documents and CRFs are reviewed for accuracy and completeness. Any correction will be accurately accounted for.

Finger prick and venous blood collection will be done by the trial nurses. Collection of all samples will be recorded on the CRF.

All Field assistants and their supervisors including trial nurses will be fully trained. Selected participants from the RCH teams will also be trained. Further refresher training will be conducted as the trial progresses. Weekly field team meetings and monthly meetings of the entire trial team will be convened in order to discuss all problems and lessons from the trial.

We will also work with the Quality and Laboratory Management teams to write a detailed Analytical Plan for the trial and appoint a study team member (likely one SO) as the Analytical Project Manager who will be responsible for overseeing and ensuring the quality of the laboratory activities for the trial.

10.1 Study monitoring

A risk-based monitoring plan will developed with the MRCG Clinical Trial Support Office prior to study starting. This will describe monitoring frequencies and content of Site Initiation, Interim Montoring and Close-out Visits.

11 Ethical considerations

This study is conducted in accordance with the principles set forth in the ICH Harmonised Tripartite Guideline for Good Clinical Practice and the Declaration of Helsinki in its current version (see appendix), whichever affords the greater protection to the participants.

11.1 General considerations on human subject protection

Study participants will be young children and the study protocol will be explained to their mothers/guardians orally in the presence of an independent witness in case they are illiterate or in writing. No children will start any study specific procedure before informed consent is obtained.

The study investigates iron supplementation in doses specifically recommended for this age group by the WHO. Participants will not get any remuneration but will have free basic medical care for the duration of the study.

11.1.1 Rationale for participant selection

Young children, together with pregnant women, are the two most affected population groups in relation to anaemia and iron deficiency anaemia, and the groups for which better iron supplementation strategies are required.

In pregnant women the main problems of current iron supplements are the gastrointestinal side-effects such as nausea, abdominal cramps, heartburn and constipation (15), which affect compliance with treatment. Our future aim is to be able to also conduct a trial in pregnancy to test the hypothesis that IHAT would not be associated with these side-effects in the pregnant women. We are actively seeking funding for this.

However, in our opinion the most pressing need in relation to iron supplementation in developing countries is to find an alternative iron supplement for use in young children living in areas at risk of enteric infection. As mentioned in the Background Section, RCTs with current iron supplementation in nearly ten thousand young children living in developing countries have consistently shown that these are associated with increased infection including bloody diarrhoea (3, 6-8) and detrimental changes to the gut microbiome and gut inflammation (8, 9), further increasing the burden from enteric infection and environmental enteropathy (i.e. persistent gut damage and inflammation that leads to malabsorption), which is a major cause of growth failure in children in resource-poor environments (10, 11). Furthermore, it appears that these effects are much more relevant in those resource-poor countries where enteric infection risk is higher and in the pre-school age group since in South-African 6-11 years old children with a low enteropathogen burden, iron supplementation did not significantly affect the dominant bacterial groups in the gut or gut inflammation (53). The specific problem this proposal seeks to address relates to these effects of oral iron supplements for treating anaemia in young children. Hence, why we propose to conduct the trial in young children living in the most deprived areas of The Gambia where the risk for enteric infection is higher.

More than 70% of all children under 5 y. in The Gambia will be anaemic and in most cases this will be due to iron deficiency. The consequences of anaemia and iron deficiency include impaired neurocognitive development and immunocompetence leading to substantial loss of human potential.
Combating anaemia due to iron deficiency is a challenge due to the potential negative side-effects of iron when given to people with infections, as is extremely common in young children in The Gambia.

Specifically, we will be addressing the fact that current iron supplements increase risk of moderate-severe diarrhoea in these children, particularly in settings where risk for enteric infection is high, as we mentioned above. In the 21st century, we simply cannot accept the current view that increased risk of infectious diarrhoea in young children is normal collateral damage of oral iron supplementation in populations at high risk of enteric infection. This is a problem of current iron supplements and we believe that IHAT will work differently for all the reasons explained in the Rationale Section of this protocol. IHAT may also offer other advantages in relation to conventional iron supplements in relation to efficiency of absorption of consecutive daily doses and systemic infection risk as explained in the Rationale Section of this protocol.

Finally, we wish to note that we believe that IHAT's main target population group, where IHAT's benefit will really outweight its added cost over current iron supplements, are young children from resource-poor countries. This is because the main advantage of IHAT over other iron supplements relates to its intestinal safety and, as we mentioned above, young children living in areas of enteric infection risk are the main population group affected by the negative effect of current iron supplements on intestinal infection (including bloody diarrhoea) and inflammation (contributing to environmental enteropathy). Essentially, if IHAT works in the proposed trial, where it will be tested in the population group that is more responsive to the adverse effects of iron supplementation (i.e. young children at risk of enteric infection), then it will work in any other population group. But the reverse is not true, i.e. if IHAT works in adults it does not mean it would work in children, and similarly if IHAT works in older children it does not mean it would work in the young children group, since these are the subjects with the most immature gut where the microbiome is not stable and where the mucosa is more susceptible.

This is why it is crucial to conduct the trial in the young children living in some of the most deprived and infectious communities in the Gambia. Conducting the study in adults would not provide us with clinically-meaningful information about the impact of IHAT on enteric infection and diarrhoea risk, since this effect is not commonly observed in adults (possibly due to the immature gut microbiome and mucosa in young children) with iron supplementation, and would just delay further investment and the authorisation of IHAT for use in children, which would be unethical in our view. As such, we follow the recommendation of the European Medicines Agency to study medicines intended to be used in children in a paediatric population rather than inferring the information and dosages from adult trials (Regulation (EC) No 1901/2006). We appreciate that children are a more vulnerable group for the first Phase II trial of IHAT or indeed any other form of iron supplementation, but they are also the group that will most benefit from a better iron supplement. The health of all children in our study will be closely monitored as will all adverse events including all diarrhoea episodes, malaria and other co-infections. Therefore, ethically we will not be subjecting children to any unnecessary risk or, in fact, any more risk that when current iron supplements are used as per national guidelines. In the worst case scenario, IHAT will be

the same as currently used ferrous suphate or fumarate in terms of diarrhoea and other adverse events.

This study will provide the first Phase II trial data with IHAT and will enable us to obtain high-quality clinical data for its safety and efficacy in correcting IDA in children, the population group most in need of an alternative oral iron supplement. If IHAT is successful in the trial, these data will provide the evidence needed to encourage further investment so that IHAT can be implemented as a novel iron source for use in micronutrient intervention strategies aimed at children and women living in resource-poor countries and, hence, reduce the global burden of IDA.

11.1.2 Rationale for use of a placebo group

We have thought very carefully about the lower threshold of Hb inclusion for all study arms and we wish to note that we are not contravening the current national guidelines, since there is no mandatory iron supplementation for this age group in the Gambia. All our prior iron intervention trials in The Gambia, Kenya and Tanzania, have used the Hb 7-11 g/dl inclusion criteria. The rationale being that Hb below 7 g/dl is the current WHO cut-off for severe anaemia in children under 59 months of age (WHO/NMH/NHD/MNM/11.1), and these children would be the ones presenting with more visible symptoms of anaemia and most likely to be automatically given oral iron treatment.

Since recent trials have demonstrated a possible detrimental effect of iron supplements in pre-school age children, there is now a strong ethical rationale for a ‘no-iron’ control group (i.e. placebo). It is crucial to have this placebo control group in our study, not only for determining background ‘side-effects’ but also, and importantly, for assessing the true treatment effect size of current iron supplementation (i.e. ferrous sulphate) in Hb levels. There have been numerous iron supplementation studies in young children in resource-poor countries, including in the Gambia, that have failed to show any decrease in anaemia prevalence with iron supplementation, and we feel it is necessary that we determine in this study if iron supplements are preventing Hb from falling further in relation to a ‘no iron’ (or placebo) group, which we consider to be an important positive effect, even when anaemia prevalence may not decrease for this age group due to the high demands for iron during fast growth.
It is also important to have a placebo group in this study so that we can perform an exploratory analysis and test for assay sensitivity and to determine treatment effect size with IHAT so that these data can be used to power future studies.

In the proposed study we will include only children with mild-moderate anaemia, and we will exclude any children with severe anaemia, who would be most likely identified and referred for iron treatment

according to the national policy in The Gambia. The children with Hb between 7 and 11 g/dl would probably not be identified as anaemic had they not been offered screening through our study and would not have been provided with oral iron supplements. Within this group, the children with Hb between 7 and 8 g/dl are the ones most in need of an alternative oral iron treatment, one that is both effective and safe. In any case, as we mentioned above, we will monitor very closely the Hb levels in all study arms and exclude and treat according to the national guidelines any children where Hb falls below 7 g/dl at any point during the study. Furthermore, at the end of the study, any child who remains anaemic will be offered the standard iron supplementation according to national policy. This means that we will only be delaying treatment for the children in the placebo group by 4 months (unless they become severely anaemic during the study, in which case they receive treatment immediately).

11.1.3 Evaluation of risks and benefits

There are risks associated with a large intake of iron supplements especially in areas of malaria endemicity. The dose of iron given daily in the reference arm (12.5 mg) is according to WHO guidelines for the age group children in non-malarious areas or malaria-endemic areas where it should be implemented in conjunction with measures to prevent, diagnose and treat malaria and co-infections. The iron dose in the IHAT arm (20 mg) is the bioequivalent dose, i.e. the same absolute amount of iron should be absorbed as in the ferrous sulphate arm, and because IHAT should be safer to the gut, the unabsorbed fraction should not cause detrimental effects, such as infectious diarrhoea. This dose (20 mg Fe) is still less than the new WHO recommendation for children in our 24-35 mo. age group (i.e. 30 mg, (40)). In any case, in all arms of the trial these adverse events will be closely monitored.

Additionally, we have put in place the following strategies to mitigate the risk of possible interactions between iron supplements and malaria or other co-infections:

(1) data from the Gambia over the last 5 years (medical records from the Kiang West region) shows that the peak malaria months are Oct and Nov and, therefore, we have timed the intervention period to avoid these months, (2) trained field workers will be visiting all children every day during the 12 weeks supplementation period in order to supervise the administration of the iron supplements or placebo and on these occasions they will check on the children's health status and actively look for signs of malaria and co-infections, if a child shows signs of these infections the study nurse will perform adequate tests and the child will be offered the appropriate treatment/referral to the next Health Centre. In case of a fever, a malaria rapid test will be performed and if positive the child will be treated according to national guidelines. A sick child will always be visited by a study nurse for further clinical investigations and if needed referred to

the nearest Health Centre. These visits will carry on for 4 weeks after the end of the study intervention and, during both the intervention and this follow-up period, morbidity data will be captured every other day. This is similar to what was done in the HIGH study and we do not anticipate any difficulties of implementation. Every week, the child will visit one of the study health facilities for a haemoglobin check-up and malaria RDT testing, and this will ensure that children that develop asymptomatic malaria are identified and treated according to national guidelines.

Participants will experience some transient pain during blood sampling, which will be minimised by recruiting well-trained nurses for the study.

Children will benefit from daily monitoring of their health and from immediate care in case of illness. Although the mechanism of combating iron deficiency is problematic, it is clear that iron is a key micronutrient for the development of the immune system and cognitive function in these young children.

11.2 Informed consent

All field workers taking part in the recruitment of participants will be trained on translating the contents of the information sheet and the details of it will be explained to illiterate mothers in a language they understand in the presence of an independent literate witness. The literate mothers/parental guardian will be allowed to read the information sheet in their own time. They will be given enough time to ask questions and decide if they want their child to participate. Informed consent will be recorded by a signature or thumbprint on the consent form. The consent form together with the trial protocol and information sheet is attached to this submission.

11.3 Participant confidentiality

Each participant will be allocated an individual identification (ID) number and these will be used to label all samples collected for the study and on the CRF during the course of the study. All data will be linked-anonymised and the linkage to the ID will not be possible without a lookup table, which will be held only by the data manager and designated data staff during the course of the study. Once data collection is complete, analysis will be performed on an anonymised copy of the data. At all stages, staff/collaborators responsible for sample analysis will be blinded as to

the subject's identification. Together, these processes will ensure complete confidentiality of the data gathered and impartiality of data analysis.

11.4 Future use of stored specimen

Some of the samples will be transferred to Kings College London (serum) and the Wellcome Trust Sanger Institute (faecal DNA) for analysis of outcomes not available in the Gambia.

Aliquots of blood and stool samples will be kept frozen at -70°C for future analysis. This may include DNA analysis and export of samples. This was a request from the funders. We will obtain informed consent from the mothers/guardians for this to be the case within the study informed consent. Any future use would require PI, MRCG SCC and EC approval.

12 Financing and insurance

The research related costs of the proposed trial will be paid by a grant from the Bill & Melinda Gates Foundation.

The MRC will sponsor this research and as such research participants will be protected in accordance with the MRC Statement on Indemnity:

'In relation to instances where the MRC is the sponsor of research the MRC may consider making an ex gratia payment when a significant adverse reaction in the form of a personal injury has occurred which is likely to have been caused by, or materially contributed to, by participation in a research study. In deciding whether to make such a payment, the MRC will not require the research participant to demonstrate that the personal injury has been caused by a breach of any duty of care that may have been owed by the MRC.'

13 Publication policy

Our planned dissemination avenues include: at least three publications in high impact peer-reviewed open-access scientific journals with a wide readership (e.g. Lancet, JAMA, NEJM), presentation at international conferences (e.g. the Micronutrient Forum) and dissemination of the trial findings to organizations such as the WHO, UNICEF, UN World Food Programme and to the National Nutrition Agency and the Ministry of Health in The Gambia.

14 References

1. WHO. The global burden of disease: 2004 update. Geneva: WHO, 2008.
2. Sazawal S, Black RE, Ramsan M, Chwaya HM, Stoltzfus RJ, Dutta A, et al. Effects of routine prophylactic supplementation with iron and folic acid on admission to hospital and mortality in preschool children in a high malaria transmission setting: community-based, randomised, placebo-controlled trial. Lancet. 2006;367(9505):133-43.
3. Soofi S, Cousens S, Iqbal SP, Akhund T, Khan J, Ahmed I, et al. Effect of provision of daily zinc and iron with several micronutrients on growth and morbidity among young children in Pakistan: a cluster-randomised trial. Lancet. 2013;382(9886):29-40.
4. Prentice AM, Verhoef H, Cerami C. Iron fortification and malaria risk in children. Jama. 2013;310(9):914-5.
5. Prentice AM. Iron metabolism, malaria, and other infections: what is all the fuss about? J Nutr. 2008;138(12):2537-41.
6. Mayo-Wilson E, Imdad A, Junior J, Dean S, Bhutta ZA. Preventive zinc supplementation for children, and the effect of additional iron: a systematic review and meta-analysis. BMJ Open. 2014;4(6):e004647.
7. Zlotkin S, Newton S, Aimone AM, et al. Effect of iron fortification on malaria incidence in infants and young children in ghana: A randomized trial. Jama. 2013;310(9):938-47.
8. Jaeggi T, Kortman GA, Moretti D, Chassard C, Holding P, Dostal A, et al. Iron fortification adversely affects the gut microbiome, increases pathogen abundance and induces intestinal inflammation in Kenyan infants. Gut. 2014.
9. Zimmermann MB, Chassard C, Rohner F, N'Goran E K, Nindjin C, Dostal A, et al. The effects of iron fortification on the gut microbiota in African children: a randomized controlled trial in Cote d'Ivoire. Am J Clin Nutr. 2010;92(6):1406-15.
10. Naylor C, Lu M, Haque R, Mondal D, Buonomo E, Nayak U, et al. Environmental Enteropathy, Oral Vaccine Failure and Growth Faltering in Infants in Bangladesh. EBioMedicine. 2015;2(11):1759-66.
11. Lin A, Arnold BF, Afreen S, Goto R, Huda TM, Haque R, et al. Household environmental conditions are associated with enteropathy and impaired growth in rural Bangladesh. Am J Trop Med Hyg. 2013;89(1):130-7.
12. Werner T, Wagner SJ, Martinez I, Walter J, Chang JS, Clavel T, et al. Depletion of luminal iron alters the gut microbiota and prevents Crohn's disease-like ileitis. Gut. 2011;60(3):325-33.
13. Dogan B, Suzuki H, Herlekar D, Sartor RB, Campbell BJ, Roberts CL, et al. Inflammation-associated Adherent-invasive Escherichia coli Are Enriched in Pathways for Use of Propanediol and Iron and M-cell Translocation. Inflamm Bowel Dis. 2014;20(11):1919-32.
14. Prentice AM, Doherty CP, Abrams SA, Cox SE, Atkinson SH, Verhoef H, et al. Hepcidin is the major predictor of erythrocyte iron incorporation in anemic African children. Blood. 2012;119(8):1922-8.

15. Tolkien Z, Stecher L, Mander AP, Pereira DI, Powell JJ. Ferrous sulfate supplementation causes significant gastrointestinal side-effects in adults: a systematic review and meta-analysis. PLoS One. 2015;10(2):e0117383.
16. Radulescu S, Brookes MJ, Salgueiro P, Ridgway RA, McGhee E, Anderson K, et al. Luminal iron levels govern intestinal tumorigenesis after apc loss in vivo. Cell Rep. 2012;2(2):270-82.
17. Seril DN, Liao J, Ho KL, Warsi A, Yang CS, Yang GY. Dietary iron supplementation enhances DSS-induced colitis and associated colorectal carcinoma development in mice. Dig Dis Sci. 2002;47(6):1266-78.
18. Seril DN, Liao J, Yang CS, Yang GY. Systemic iron supplementation replenishes iron stores without enhancing colon carcinogenesis in murine models of ulcerative colitis: comparison with iron-enriched diet. Dig Dis Sci. 2005;50(4):696-707.
19. Loh YH, Jakszyn P, Luben RN, Mulligan AA, Mitrou PN, Khaw KT. N-nitroso compounds and cancer incidence: the European Prospective Investigation into Cancer and Nutrition (EPIC)-Norfolk Study. American Journal of Clinical Nutrition. 2011;93(5):1053-61.
20. Lunn JC, Kuhnle G, Mai V, Frankenfeld C, Shuker DE, Glen RC, et al. The effect of haem in red and processed meat on the endogenous formation of N-nitroso compounds in the upper gastrointestinal tract. Carcinogenesis. 2007;28(3):685-90.
21. Santiago P. Ferrous versus ferric oral iron formulations for the treatment of iron deficiency: a clinical overview. Scientific World Journal. 2012;2012:846824.
22. Ruiz-Arguelles GJ, Diaz-Hernandez A, Manzano C, Ruiz-Delgado GJ. Ineffectiveness of oral iron hydroxide polymaltose in iron-deficiency anemia. Hematology. 2007;12(3):255-6.
23. Powell JJ, Bruggraber SFA, Faria N, Poots LK, Hondow N, Pennycook TJ, et al. A nano-disperse ferritin-core mimetic that efficiently corrects anemia without luminal iron redox activity. Nanomedicine: Nanotechnology, Biology and Medicine. 2014;10(7):1529-38.
24. Pan YH, Sader K, Powell JJ, Bleloch A, Gass M, Trinick J, et al. 3D morphology of the human hepatic ferritin mineral core: new evidence for a subunit structure revealed by single particle analysis of HAADF-STEM images. J Struct Biol. 2009;166(1):22-31.
25. Michel FM, Ehm L, Antao SM, Lee PL, Chupas PJ, Liu G, et al. The structure of ferrihydrite, a nanocrystalline material. Science. 2007;316(5832):1726-9.
26. Theil EC, Chen H, Miranda C, Janser H, Elsenhans B, Nunez MT, et al. Absorption of iron from ferritin is independent of heme iron and ferrous salts in women and rat intestinal segments. J Nutr. 2012;142(3):478-83.
27. Bejjani S, Pullakhandam R, Punjal R, Nair KM. Gastric digestion of pea ferritin and modulation of its iron bioavailability by ascorbic and phytic acids in caco-2 cells. World J Gastroenterol. 2007;13(14):2083-8.
28. Lonnerdal B, Bryant A, Liu X, Theil EC. Iron absorption from soybean ferritin in nonanemic women. Am J Clin Nutr. 2006;83(1):103-7.
29. Pereira DIA, Bruggraber SFA, Faria N, Poots LK, Tagmount MA, Aslam MF, et al. Nanoparticulate iron(III) oxo-hydroxide delivers safe iron that is well absorbed and utilised in humans. Nanomedicine: Nanotechnology, Biology and Medicine. 2014;10(8):1877-86.

30. Moretti D, Goede JS, Zeder C, Jiskra M, Chatzinakou V, Tjalsma H, et al. Oral iron supplements increase hepcidin and decrease iron absorption from daily or twice-daily doses in iron-depleted young women. Blood. 2015;126(17):1981-9.
31. Hutchinson C, Al-Ashgar W, Liu DY, Hider RC, Powell JJ, Geissler CA. Oral ferrous sulphate leads to a marked increase in pro-oxidant nontransferrin-bound iron. Eur J Clin Invest. 2004;34(11):782-4.
32. Barton Pai A, Pai MP, Depczynski J, McQuade CR, Mercier RC. Non-transferrin-bound iron is associated with enhanced Staphylococcus aureus growth in hemodialysis patients receiving intravenous iron sucrose. Am J Nephrol. 2006;26(3):304-9.
33. Cross JH, Bradbury RS, Fulford AJ, Jallow AT, Wegmuller R, Prentice AM, et al. Oral iron acutely elevates bacterial growth in human serum. Sci Rep. 2015;5:16670.
34. Latunde-Dada GO, Pereira DI, Tempest B, Ilyas H, Flynn AC, Aslam MF, et al. A Nanoparticulate Ferritin-Core Mimetic Is Well Taken Up by HuTu 80 Duodenal Cells and Its Absorption in Mice Is Regulated by Body Iron. The Journal of Nutrition. 2014;144(12):1896-902.
35. Aslam MF, Frazer DM, Faria N, Bruggraber SF, Wilkins SJ, Mirciov C, et al. Ferroportin mediates the intestinal absorption of iron from a nanoparticulate ferritin core mimetic in mice. Faseb J. 2014;28(8):3671-8.
36. Pereira DI, Mergler BI, Faria N, Bruggraber SF, Aslam MF, Poots LK, et al. Caco-2 Cell Acquisition of Dietary Iron(III) Invokes a Nanoparticulate Endocytic Pathway. PLoS One. 2013;8(11):e81250.
37. Pereira DIA, Aslam MF, Frazer DM, Schmidt A, Walton GE, McCartney AL, et al. Dietary iron depletion at weaning imprints low microbiome diversity and this is not recovered with oral nano Fe(III). MicrobiologyOpen. 2015;4(1):12-27.
38. Thurnham DI, McCabe LD, Haldar S, Wieringa FT, Northrop-Clewes CA, McCabe GP. Adjusting plasma ferritin concentrations to remove the effects of subclinical inflammation in the assessment of iron deficiency: a meta-analysis. Am J Clin Nutr. 2010;92(3):546-55.
39. Engle-Stone R, Nankap M, Ndjebayi AO, Erhardt JG, Brown KH. Plasma ferritin and soluble transferrin receptor concentrations and body iron stores identify similar risk factors for iron deficiency but result in different estimates of the national prevalence of iron deficiency and iron-deficiency anemia among women and children in Cameroon. J Nutr. 2013;143(3):369-77.
40. WHO. Guideline: Daily iron supplementation in infants and children. Geneva, Switzerland: World Health Organization, 2016.
41. Liu J, Gratz J, Amour C, Kibiki G, Becker S, Janaki L, et al. A laboratory-developed TaqMan Array Card for simultaneous detection of 19 enteropathogens. Journal of clinical microbiology. 2013;51(2):472-80.
42. Christian LM, Iams JD, Porter K, Glaser R. Inflammatory responses to trivalent influenza virus vaccine among pregnant women. Vaccine. 2011;29(48):8982-7.
43. Paine NJ, Ring C, Bosch JA, Drayson MT, Veldhuijzen van Zanten JJ. The time course of the inflammatory response to the Salmonella typhi vaccination. Brain Behav Immun. 2013;30:73-9.

44. Ma Y, Podinovskaia M, Evans PJ, Emma G, Schaible UE, Porter J, et al. A novel method for non-transferrin-bound iron quantification by chelatable fluorescent beads based on flow cytometry. Biochem J. 2014;463(3):351-62.
45. Singh S, Hider RC, Porter JB. A direct method for quantification of non-transferrin-bound iron. Anal Biochem. 1990;186(2):320-3.
46. Sebastiani G, Pantopoulos K. NTBI unveiled by chelatable fluorescent beads. Biochem J. 2014;463(3):e7-9.
47. Suchdev PS, Davis SM, Bartoces M, Ruth LJ, Worrell CM, Kanyi H, et al. Soil-transmitted helminth infection and nutritional status among urban slum children in Kenya. Am J Trop Med Hyg. 2014;90(2):299-305.
48. Ahmed A, Al-Mekhlafi HM, Al-Adhroey AH, Ithoi I, Abdulsalam AM, Surin J. The nutritional impacts of soil-transmitted helminths infections among Orang Asli schoolchildren in rural Malaysia. Parasit Vectors. 2012;5:119.
49. Committee WE. Prevention and control of schistosomiasis and soil-transmitted helminthiasis. World Health Organization technical report series. 2002;912:i-vi, 1-57.
50. Eren AM, Morrison HG, Lescault PJ, Reveillaud J, Vineis JH, Sogin ML. Minimum entropy decomposition: unsupervised oligotyping for sensitive partitioning of high-throughput marker gene sequences. Isme J. 2015;9(4):968-79.
51. Stallard N. Optimal sample sizes for phase II clinical trials and pilot studies. Statistics in medicine. 2012;31(11-12):1031-42.
52. De-Regil LM, Suchdev PS, Vist GE, Walleser S, Pena-Rosas JP. Home fortification of foods with multiple micronutrient powders for health and nutrition in children under two years of age. Cochrane Database Syst Rev. 2011(9):CD008959.
53. Dostal A, Baumgartner J, Riesen N, Chassard C, Smuts CM, Zimmermann MB, et al. Effects of iron supplementation on dominant bacterial groups in the gut, faecal SCFA and gut inflammation: a randomised, placebo-controlled intervention trial in South African children. The British journal of nutrition. 2014;112(4):547-56.

Supplements, appendices and other documents

Appendix 1: Project Timeline

Task Name	Start	Finish
**Project**	**Fri 01/04/16**	**Fri 01/03/19**
**Regulatory and Ethical approvals**	**Fri 01/04/16**	**Wed 30/09/16**
Protocol written	Fri 01/04/16	Fri 29/04/16
Sponsor review	Mon 02/05/16	Mon 20/06/16
CTA application	Tue 21/06/16	Wed 09/11/16
Ethics application	Tue 21/06/16	Fri 09/09/16
GMP clinical batch IHAT ordered	Wed 01/06/16	Fri 10/03/17
Interim report submitted (M 1)	Mon 01/08/16	Wed 30/09/16
Milestone 1	Wed 30/09/16	Wed 30/09/16
**Trial Setup and Recruitment**	**Tue 02/08/16**	**Wed 31/05/17**
GMP encapsulation of IHAT and comparators ordered	Thu 01/09/16	Thu 15/06/17
Protocol amendments	Mon 27/02/17	Fri 31/03/17
Trial protocol submitted	Mon 01/05/17	Fri 28/07/17
Trial supplies ordered	Mon 01/08/16	Fri 31/03/17
Trial documentation prepared	Mon 01/08/16	Fri 31/03/17
Trial database built	Mon 01/08/16	Fri 28/04/17
Setup of trial committees	Thu 01/09/16	Fri 16/12/16
Staff training at MRC Basse	Mon 01/08/16	Thur 31/08/17
Upper river communities sensitization	Mon 12/12/16	Fri 28/07/17
Interim report submitted	Fri 05/05/17	Fri 05/05/17

Protocol #: 1489

Task Name	Start	Finish
Participant recruitment cohort 1	Mon 30/10/17	Fri 24/11/17
Participant recruitment cohort 2	Mon 29/01/18	Fri 23/02/18
Participant recruitment cohort 3	Mon 30/04/18	Fri 25/05/18
Local analysis of screening samples	Mon 30/10/17	Fri 01/06/18
Interim report submitted (M 2)	Fri 08/06/17	Fri 08/06/18
Milestone 2	Fri 08/06/18	Fri 08/06/18
**Trial Field Data Collection**	**Mon 04/12/17**	**Fri 31/08/18**
Cohort 1 intervention	Mon 04/12/17	Fri 23/02/18
Cohort 2 intervention	Mon 12/03/18	Fri 25/05/18
Cohort 3 intervention	Mon 18/06/18	Fri 14/09/18
AEs follow-up	Mon 04/12/17	Fri 05/10/18
Local analysis of trial samples	Mon 04/12/17	Fri 05/10/18
Interim report submitted (M 3)	Fri 26/10/18	Fri 26/10/18
Milestone 3	Fri 26/10/18	Fri 26/10/18
**Analysis and Reporting**	**Mon 06/08/18**	**Fri 01/03/19**
External analysis of trial samples (microbiome and NTBI)	Mon 17/09/18	Fri 21/12/18
Data queries resolved	Mon 30/10/17	Fri 05/10/18
Data analysis	Mon 08/10/18	Fri 25/01/19
Data monitoring committee report	Wed 28/01/19	Fri 01/02/19
Final project report	Mon 28/01/19	Fri 01/03/19
Publication of findings submitted	Mon 04/03/19	Sun 31/03/19
**Milestone 4 and Project End**	**Sun 31/03/19**	**Sun 31/03/19**

Appendix 2: Target Product Profile (TPP)

Proposed Targeted Product Profile (TPP) for IHAT for the treatment of IDA in children (i.e. following the proposed trial and once market authorisation is secured). For the current proposed trial we are working towards meeting the minimum acceptable results rather than the target, which would be for the next stage trial.

Product Properties	Minimum Acceptable Result	Target Result
**Dosing regimen**	Oral, at least four days/ week	Oral, once a day
**Formulation**	Single-API dose containing 20 mg Fe (bioequivalent to 12.5 mg Fe as $FeSO_4$) for children.	Multi-micronutrient powder formulations with taste masking for paediatrics, containing 20 mg Fe.
**Cost of treatment**	≤$0.05 per child dose	≤$0.01 per child dose
**Shelf-life of formulated product**	2 yr at ≤ 25°C	2 yr at ≤ 40°C
**Nutrient-nutrient interactions**	No unmanageable risk in terms of solid state or pharmacokinetic interactions	No risks in terms of solid state or pharmacokinetic interactions
**Clinical efficacy (reduction in anaemia after 3 months)**	30% of patients with Hb increase ≥ 1 g/dL	30% of patients with anaemia resolved (HB>11 g/dL)
**Clinical efficacy (reduction in iron deficiency after 6 months)**	30% of patients with ID resolved (sTfR/log10 ferritin (sTfR-F) index ≤2)	50% of patients with ID resolved (sTfR-F index ≤2)
**Safety**	Few drug-related SAEs, including diarrhoea	No drug-related SAEs Minimal drug-related AEs, including diarrhoea
**Intestinal infection**	Drug-related bacterial infection in ≤ 15% of subjects	No drug-related bacterial infection
**Gut microbiome changes**	No significant increase in Enterobacteria or Enterobacteria/(Bifidobacteria +Lactobacillus	Less Enterobacteria relative to Bifidobacteria and Lactobacillus.

Appendix 3:

WORLD MEDICAL ASSOCIATION DECLARATION OF HELSINKI
Ethical Principles for Medical Research Involving Human Subjects

Adopted by the 18th WMA General Assembly, Helsinki, Finland, June 1964
and amended by the:
29th WMA General Assembly, Tokyo, Japan, October 1975
35th WMA General Assembly, Venice, Italy, October 1983
41st WMA General Assembly, Hong Kong, September 1989
48th WMA General Assembly, Somerset West, Republic of South Africa, October 1996
52nd WMA General Assembly, Edinburgh, Scotland, October 2000
53rd WMA General Assembly, Washington DC, USA, October 2002 (Note of Clarification added)
55th WMA General Assembly, Tokyo, Japan, October 2004 (Note of Clarification added)
59th WMA General Assembly, Seoul, Republic of Korea, October 2008
64th WMA General Assembly, Fortaleza, Brazil, October 2013

Preamble

1. The World Medical Association (WMA) has developed the Declaration of Helsinki as a statement of ethical principles for medical research involving human subjects, including research on identifiable human material and data.

The Declaration is intended to be read as a whole and each of its constituent paragraphs should be applied with consideration of all other relevant paragraphs.

2. Consistent with the mandate of the WMA, the Declaration is addressed primarily to physicians. The WMA encourages others who are involved in medical research involving human subjects to adopt these principles.

General Principles

3. The Declaration of Geneva of the WMA binds the physician with the words, "The health of my patient will be my first consideration," and the International Code of Medical Ethics declares that, "A physician shall act in the patient's best interest when providing medical care."

4. It is the duty of the physician to promote and safeguard the health, well-being and rights of patients, including those who are involved in medical research. The physician's knowledge and conscience are dedicated to the fulfilment of this duty.

5. Medical progress is based on research that ultimately must include studies involving human subjects.

6. The primary purpose of medical research involving human subjects is to understand the causes, development and effects of diseases and improve preventive, diagnostic and therapeutic interventions (methods, procedures and treatments). Even the best proven interventions must be evaluated continually through research for their safety, effectiveness, efficiency, accessibility and quality.

7. Medical research is subject to ethical standards that promote and ensure respect for all human subjects and protect their health and rights.

8. While the primary purpose of medical research is to generate new knowledge, this goal can never take precedence over the rights and interests of individual research subjects.

9. It is the duty of physicians who are involved in medical research to protect the life, health, dignity, integrity, right to self-determination, privacy, and confidentiality of personal information of research subjects. The responsibility for the protection of research subjects must always rest with the physician or other health care professionals and never with the research subjects, even though they have given consent.

10. Physicians must consider the ethical, legal and regulatory norms and standards for research involving human subjects in their own countries as well as applicable international norms and standards. No national or international ethical, legal or regulatory requirement should reduce or eliminate any of the protections for research subjects set forth in this Declaration.

11. Medical research should be conducted in a manner that minimises possible harm to the environment.

12. Medical research involving human subjects must be conducted only by individuals with the appropriate ethics and scientific education, training and qualifications. Research on patients or healthy volunteers requires the supervision of a competent and appropriately qualified physician or other health care professional.

13. Groups that are underrepresented in medical research should be provided appropriate access to participation in research.

14. Physicians who combine medical research with medical care should involve their patients in research only to the extent that this is justified by its potential preventive, diagnostic or therapeutic value and if the physician has good reason to believe that participation in the research study will not adversely affect the health of the patients who serve as research subjects.

15. Appropriate compensation and treatment for subjects who are harmed as a result of participating in research must be ensured.

Risks, Burdens and Benefits

16. In medical practice and in medical research, most interventions involve risks and burdens.

Medical research involving human subjects may only be conducted if the importance of the objective outweighs the risks and burdens to the research subjects.

17. All medical research involving human subjects must be preceded by careful assessment of predictable risks and burdens to the individuals and groups involved in the research in comparison with foreseeable benefits to them and to other individuals or groups affected by the condition under investigation.

Measures to minimise the risks must be implemented. The risks must be continuously monitored, assessed and documented by the researcher.

18. Physicians may not be involved in a research study involving human subjects unless they are confident that the risks have been adequately assessed and can be satisfactorily managed.

When the risks are found to outweigh the potential benefits or when there is conclusive proof of definitive outcomes, physicians must assess whether to continue, modify or immediately stop the study.

Vulnerable Groups and Individuals

19. Some groups and individuals are particularly vulnerable and may have an increased likelihood of being wronged or of incurring additional harm.

All vulnerable groups and individuals should receive specifically considered protection.

20. Medical research with a vulnerable group is only justified if the research is responsive to the health needs or priorities of this group and the research cannot be carried out in a non-vulnerable group. In addition, this group should stand to benefit from the knowledge, practices or interventions that result from the research.

Scientific Requirements and Research Protocols

21. Medical research involving human subjects must conform to generally accepted scientific principles, be based on a thorough knowledge of the scientific literature, other relevant sources of information, and adequate laboratory and, as appropriate, animal experimentation. The welfare of animals used for research must be respected.

22. The design and performance of each research study involving human subjects must be clearly described and justified in a research protocol.

The protocol should contain a statement of the ethical considerations involved and should indicate how the principles in this Declaration have been addressed. The protocol should include information regarding funding, sponsors, institutional affiliations, potential conflicts of interest, incentives for subjects and information regarding provisions for treating and/or compensating subjects who are harmed as a consequence of participation in the research study.

In clinical trials, the protocol must also describe appropriate arrangements for post-trial provisions.

Research Ethics Committees

23. The research protocol must be submitted for consideration, comment, guidance and approval to the concerned research ethics committee before the study begins. This committee must be transparent in its functioning, must be independent of the researcher, the sponsor and any other undue influence and must be duly qualified. It must take into consideration the laws and regulations of the country or countries in which the research is to be performed as well as applicable international norms and standards but these must not be allowed to reduce or eliminate any of the protections for research subjects set forth in this Declaration.

The committee must have the right to monitor ongoing studies. The researcher must provide monitoring information to the committee, especially information about any serious adverse events. No amendment to the protocol may be made without consideration and approval by the committee. After the end of the study, the researchers must submit a final report to the committee containing a summary of the study's findings and conclusions.

Privacy and Confidentiality

24. Every precaution must be taken to protect the privacy of research subjects and the confidentiality of their personal information.

Informed Consent

25. Participation by individuals capable of giving informed consent as subjects in medical research must be voluntary. Although it may be appropriate to consult family members or community leaders, no individual capable of giving informed consent may be enrolled in a research study unless he or she freely agrees.

26. In medical research involving human subjects capable of giving informed consent, each potential subject must be adequately informed of the aims, methods, sources of funding, any possible conflicts of interest, institutional affiliations of the researcher, the anticipated benefits and potential risks of the study and the discomfort it may entail, post-study provisions and any other relevant aspects of the study. The potential subject must be informed of the right to refuse to participate in the study or to withdraw consent to participate at any time without reprisal. Special attention should be given to the specific information needs of individual potential subjects as well as to the methods used to deliver the information.

After ensuring that the potential subject has understood the information, the physician or another appropriately qualified individual must then seek the potential subject's freely-given informed consent, preferably in writing. If the consent cannot be expressed in writing, the non-written consent must be formally documented and witnessed.

All medical research subjects should be given the option of being informed about the general outcome and results of the study.

27. When seeking informed consent for participation in a research study the physician must be particularly cautious if the potential subject is in a dependent relationship with the physician or may consent under duress. In such situations the informed consent must be

sought by an appropriately qualified individual who is completely independent of this relationship.

28. For a potential research subject who is incapable of giving informed consent, the physician must seek informed consent from the legally authorised representative. These individuals must not be included in a research study that has no likelihood of benefit for them unless it is intended to promote the health of the group represented by the potential subject, the research cannot instead be performed with persons capable of providing informed consent, and the research entails only minimal risk and minimal burden.

29. When a potential research subject who is deemed incapable of giving informed consent is able to give assent to decisions about participation in research, the physician must seek that assent in addition to the consent of the legally authorised representative. The potential subject's dissent should be respected.

30. Research involving subjects who are physically or mentally incapable of giving consent, for example, unconscious patients, may be done only if the physical or mental condition that prevents giving informed consent is a necessary characteristic of the research group. In such circumstances the physician must seek informed consent from the legally authorised representative. If no such representative is available and if the research cannot be delayed, the study may proceed without informed consent provided that the specific reasons for involving subjects with a condition that renders them unable to give informed consent have been stated in the research protocol and the study has been approved by a research ethics committee. Consent to remain in the research must be obtained as soon as possible from the subject or a legally authorised representative.

31. The physician must fully inform the patient which aspects of their care are related to the research. The refusal of a patient to participate in a study or the patient's decision to withdraw from the study must never adversely affect the patient-physician relationship.

32. For medical research using identifiable human material or data, such as research on material or data contained in biobanks or similar repositories, physicians must seek informed consent for its collection, storage and/or reuse. There may be exceptional situations where consent would be impossible or impracticable to obtain for such research. In such situations the research may be done only after consideration and approval of a research ethics committee.

Use of Placebo

33. The benefits, risks, burdens and effectiveness of a new intervention must be tested against those of the best proven intervention(s), except in the following circumstances:

Where no proven intervention exists, the use of placebo, or no intervention, is acceptable; or

Where for compelling and scientifically sound methodological reasons the use of any intervention less effective than the best proven one, the use of placebo, or no intervention is necessary to determine the efficacy or safety of an intervention.

and the patients who receive any intervention less effective than the best proven one, placebo, or no intervention will not be subject to additional risks of serious or irreversible harm as a result of not receiving the best proven intervention.

Extreme care must be taken to avoid abuse of this option.

Post-Trial Provisions

34. In advance of a clinical trial, sponsors, researchers and host country governments should make provisions for post-trial access for all participants who still need an intervention identified as beneficial in the trial. This information must also be disclosed to participants during the informed consent process.

Research Registration and Publication and Dissemination of Results

35. Every research study involving human subjects must be registered in a publicly accessible database before recruitment of the first subject. We will register this trial with the www.isrctn.com.

36. Researchers, authors, sponsors, editors and publishers all have ethical obligations with regard to the publication and dissemination of the results of research. Researchers have a duty to make publicly available the results of their research on human subjects and are accountable for the completeness and accuracy of their reports. All parties should adhere to accepted guidelines for ethical reporting. Negative and inconclusive as well as positive results must be published or otherwise made publicly available. Sources of funding, institutional affiliations and conflicts of interest must be declared in the publication. Reports of research not in accordance with the principles of this Declaration should not be accepted for publication.

Unproven Interventions in Clinical Practice

37. In the treatment of an individual patient, where proven interventions do not exist or other known interventions have been ineffective, the physician, after seeking expert advice, with informed consent from the patient or a legally authorised representative, may use an unproven intervention if in the physician's judgement it offers hope of saving life, re-establishing health or alleviating suffering. This intervention should subsequently be made the object of research, designed to evaluate its safety and efficacy. In all cases, new information must be recorded and, where appropriate, made publicly available.
