# Supplementary material for: A novel nano-iron supplement versus standard treatment for iron deficiency anaemia in children 6–35 months (IHAT-GUT trial): a double-blind, randomised, placebo-controlled non-inferiority phase II trial in The Gambia
Source: eClinicalMedicine. 2023 Feb 9;56:101853. doi: 10.1016/j.eclinm.2023.101853 (PMC9985047; doi:10.1016/j.eclinm.2023.101853)
Supplement: Supplementary Data S2 [file mmc2.docx]

A novel nano-iron supplement versus standard treatment for iron deficiency anaemia in children 6-35 months (IHAT-GUT trial): A double-blind, randomised, placebo-controlled non-inferiority trial in The Gambia

Authors:

Nuredin I. Mohammed^1#^, James Wason^2,3#^, Thomas Mendy^1^, Stefan Akio Naß^1,4^, Ogochukwu Ofordile^1^, Famalang Camara^1^, Bakary Baldeh^1^, Chilel Sanyang^1^, Amadou T. Jallow^1^, Ilias Hossain^1^, Nuno Faria^5^, Jonathan J. Powell^5^, Andrew M. Prentice^1^, and Dora I.A. Pereira^1,6^*

**Supplementary Methods**

**Trial setting**

The URR has a population of approximately 200,000, with only one major town, Basse; it is typical of rural sub-Saharan Africa. Moderate-severe anaemia in children is common in these communities with over 30% of children under 5 years with haemoglobin below 9.9 g/dl(24). Diarrhoeal diseases are also common (25)^,^(26). The study area included 45 villages in the Wuli and Sandu districts, situated approximately 400 km east of the capital Banjul, on the north bank of the river Gambia. All villages had access to borehole tap water at central places.

**Screening and enrolment of participants**

Prospective participants (children 6-35 months of age) were identified through data collected by the study field team in the 45 study villages. The field team visited the parents of all young children identified as prospective participants to explain the study and answer any questions they may have. Those interested in taking part were invited to attend a screening visit at one of five study health facilities.

At screening, each child was examined by a nurse or clinician. To be eligible for the study, participants had to meet all the following inclusion criteria and none of the exclusion criteria.

**Procedures**

Children were allocated to one of the study health facilities according to the child’s home proximity to each of the 5 study health facilities (target max. 60 children seen at each health facility) and study samples were collected at one of these clinics: Yorrobawol health center, Darsilami community health post, Konkuba community health post, Taibatu health post and Chamoi Health Center (Supplementary Figure 1) and transported in cold boxes to a laboratory in Basse for sample processing and analysis. For logistical reasons, children allocated to one health facility had all their study visits (including weekly check-ups) on the same day of the week (for example, Yorrobawol health center had all study visits on Mondays).

The recruitment period for each of the three cohorts was planned to be approximately 1 month prior to enrolment into each of the sequential cohorts, with expectations to screen 50 children/day for 2 weeks, and the following 2 weeks to get all screening results back from the lab. Eligible children were invited for a pre-enrolment day back at the clinic (Day 0), for a finger prick to confirm absence of malaria and that haemoglobin was still within the inclusion range. Those confirmed eligible were then randomised and enrolled in the study.

The week after randomisation, children were taken back to the clinic again on the same day of the week as their Day 0 and this was their study Day 1. On study Day 1, we took a photo of the child (with consent) and printed and laminated a study ID card that we asked the guardian to keep safe during the study. This ID card contained the photo of the child and their randomisation/study ID number. A wrist band was also provided, showing the same study ID number, so that there was no confusion regarding the identity of the study participants. Then, we collected venous blood (total of 5 ml divided into 1 ml EDTA, 1 ml LH and 3 ml serum collection tubes) and stool samples from the children (baseline samples). Blood sample collection was done either before the first meal of the day or, in cases where this was not possible, at least 1 hour after the last meal (to avoid more dynamic iron parameters, such as hepcidin and serum iron, influencing the iron absorption from the previous meal). When it was not possible to collect stool samples at the clinic, they could be collected by the mother at home on the morning of the study visit, with a 7 days possible window for sample collection, using toilet pots and disposable liners supplied by the study team for each child. In that case, the field worker then aliquoted the stool sample into the appropriate stool sample tubes (samples for microbiome profiling were stabilised using a DNA preservative) and transported them to the laboratory for processing as feasible after collection of the sample.

On Day 1, demographic and immunisation data were collected and the morbidity questionnaire was completed.

At the end of this visit, the mother was encouraged to feed the child and once the child was settled, they were given the iron supplementation or placebo.

Each arm included an intervention period (follow-up) of 85 days plus an additional active follow-up period of 4 weeks post intervention (until Day 113). Highly trained and experienced field workers visited all children every day during the 85 days supplementation period in order to administer the iron supplements or placebo. On these occasions they also checked on the children’s general health and actively look for signs of malaria and co-infections. Children showing signs of these infections were referred to the study nurse and, if required, to the nearest health center. Three times per week, morbidity data (including questions regarding fever, diarrhoea, vomiting, cough, malaria symptoms, any other illness, appetite and any mediation taken and assessment of body temperature) were recorded (see questionnaire, Supplementary Data 4). Every week, at the study health facilities children wellbeing was checked by the study nurses and they had a finger prick capillary blood collection to determine their malaria and Hb status. Children found with a positive RDT during the study were further tested with a blood film and those confirmed as malaria positive were treated according to national guidelines. These check-ups continued 4 weeks post intervention to follow-up on adverse events (AEs) and serious adverse events (SAEs).

***Preparation and administration of investigational products***

Iron preparations and placebo comparator were supplied as powders of identical colour with each daily dose contained in a hard HPMC powder-filled easy-open capsules.

Each daily dose capsule contained the single active IMP compound (IHAT or ferrous sulphate) or the placebo compound blended with a small amount of a food grade colouring agent under cGMP prior to capsule filling in order to colour-match the IMP powders.

On the day of administration, the field assistant opened the capsule and added the entire powder content to 10 ml of Yandi orange flavour drink (a local soft drink) contained in a disposable plastic cup. This drink was selected for its acceptability by local children as well as its low levels of natural chelators (e.g. citric acid) and absence of ascorbic acid, which meant that IHAT was substantially maintained in its natural nano-disperse phase (data not shown). The dose was administered directly into the child’s mouth using a disposable Pasteur pipette (in the younger children), a spoon, or drunk directly from the plastic cup (in the older children). Whenever possible, each dose was ingested after a feed or within 1 hour of the last meal. If the child had not been fed before supplementation, the mother was encouraged to feed the child immediately after supplementation.

**Concomitant medications/treatments**

All medications were permissible, except iron supplementation other than provided by the study, including multi-micronutrient powders containing iron.

**Definitions**

**Diarrhoea**

Diarrhoea was defined as 3 or more loose or watery stools per day and moderate-severe diarrhoea refers to those diarrhoea episodes where (i) the child passes more than 5 loose or watery stools per day, or (ii) there is blood in the stool (dysentery), or (iii) the child shows signs of clinical dehydration (assessed by the study nurse based on physical signs such as little or no urination, sunken eyes, and skin that lacks its normal elasticity).

**Inflammation adjusted ferritin**

Ferritin was adjusted for inflammation using the regression model defined by the BRINDA group (Eq. 1 and 2)^7^. For children with normal CRP and AGP levels, no adjustment to the observed ferritin was made.

| Eq. 1 | ln*Ferritin_adjusted_* = ln*Ferritin_observed_* − 0.19(ln*CRP_observed_* + 2.26) − 0.74(ln*AGP_observed_* + 0.52)  where, ln*CRP_reference_* = -2.26 and ln*AGP_reference_* = -0.52 |
| --- | --- |
| Eq. 2 | *Ferritin_adjusted_* = exp ^ (ln*Ferritin_observed_*) |
