# Supplementary material for: A novel nano-iron supplement versus standard treatment for iron deficiency anaemia in children 6–35 months (IHAT-GUT trial): a double-blind, randomised, placebo-controlled non-inferiority phase II trial in The Gambia
Source: eClinicalMedicine. 2023 Feb 9;56:101853. doi: 10.1016/j.eclinm.2023.101853 (PMC9985047; doi:10.1016/j.eclinm.2023.101853)
Supplement: Supplementary Data S3 [file mmc3.pdf]

STATISTICAL ANALYSIS PLAN (SAP)

Version 1.0 – 08 Mar 2019

A novel nano-iron supplement (IHAT) to safely combat iron deficiency and anaemia (IDA) in young children: a double-blind randomised controlled trial

**Version:**	1.0
**Date**	08 Mar 2019
**Protocol number**	1489, V5.0, 21 May 2018
**SCC No**	1489
**Registration number**	NCT02941081
**Prepared by**	Nuredin Ibrahim Mohammed, Trial Statistician, MRC Unit The Gambia at The LSHTM
**Signature/date**	
**Reviewed by**	David Jeffries, Head, Statistics and Bioinformatics, MRC Unit The Gambia at The LSHTM
**Signature/date**	
**Signature/date**	Dora Pereira, University of Cambridge, UK
	James Wason, TSC Statistician, MRC Biostatistics Unit, Cambridge, UK
**Signature/date**	

Table of contents

Page

1 Introduction 5
2 Study objectives 6
2.1 Study design ... 6
2.2 Study outcomes ... 8
2.2.1 Primary ... 9
2.2.2 Secondary ... 10
2.3 Study evaluations ... 11
3 Statistical considerations 14
3.1 Study population ... 14
3.2 Definitions and derived variables ... 15
3.2.1 Demographic/baseline characteristics ... 15
3.2.2 Iron deficiency ... 15
3.2.3 Haemoglobin improvement ... 16
3.2.4 Diarrhoea ... 16
3.2.5 Treatment failures ... 17
3.2.6 Malaria ... 17
3.3 Primary Efficacy Endpoint ... 17
3.3.1 Efficacy for IDA correction ... 17
3.4 Primary Safety Endpoints ... 18
3.4.1 'Incidence density' of moderate-severe diarrhoea ... 18
3.4.2 'Period prevalence' of moderate-severe diarrhoea ... 18
3.5 Secondary Efficacy Endpoints ... 18
3.5.1 Treatment failures ... 18
3.5.2 Iron status ... 18
3.6 Secondary Safety Endpoints ... 19
3.6.1 'Longitudinal prevalence' of diarrhoea ... 19
3.6.2 'Longitudinal prevalence' of moderate-severe diarrhoea ... 19
3.6.3 'Incidence density' of bloody diarrhoea ... 19
3.6.4 Malaria infection ... 19
3.6.5 Acute respiratory infection (ARI) ... 19
3.7 Handling of missing data ... 19
3.8 Site/Village/Region specific analysis ... 20
3.9 Safety parameters ... 20
3.10 Sample size determination ... 21
3.11 Statistical analysis ... 22
3.11.1 Descriptive analysis ... 24
3.11.1.1 Subject Disposition ... 24
3.11.1.2 Demographics and baseline characteristics ... 27
3.11.1.3 Primary efficacy summary ... 32
3.11.1.4 Primary safety summary ... 32
3.11.1.5 Other safety measures ... 33
3.11.1.6 Other secondary outcomes ... 38

3.11.2 Comparative analysis 39
3.11.2.1 Primary objective 1 39
3.11.2.2 Primary objective 2 40
3.11.2.3 Primary objective 3 40
3.11.2.4 Primary objective 4 41
3.11.2.5 Secondary analysis 42
3.11.2.6 Exploratory analysis 45
4 References 46

List of abbreviations

AE	Adverse Event
AGP	Alpha-1 acid Glycoprotein
CRF	Case Report Form
CRP	C-reactive protein
CI	Confidence interval
$FeSO_4$	Ferrous Sulphate
Hb	Haemoglobin
ID	Iron Deficiency
IDA	Iron Deficiency Anaemia
IHAT	Iron Hydroxide Adipate Tartrate
MRC	Medical Research Council
MRCG	Medical Research Council Unit, The Gambia
NTBI	Non-transferrin bound iron
OR	Odds ratio
PI	Principal Investigator
RCT	Randomised Controlled Trial
RDT	Rapid Diagnostic Test
RR	Rate ratio
SAE	Serious Adverse Event
SD	Standard Deviation
sTfR	Soluble transferrin receptor
STH	Soil-Transmitted Helminths
TSAT	Transferrin saturation
TSC	Trial Steering Committee
WHO	World Health Organization

1 Introduction

Iron deficiency (ID) and its associated anaemia (IDA) remain the most common forms of micronutrient malnutrition in the world today. Globally, IDA is estimated to affect 1.24 billion people, the majority of whom are children and women from resource-poor countries, and is responsible for an estimated loss of 35 million DALYs (1.5% of total disability-adjusted life years) [1-3]. IDA is estimated to cause more years lived with disability (YLD) than all other micronutrient deficiencies, haemoglobinopathies and haemolytic anaemias combined, and is the leading contributor to YLD in most low-income countries [1]. Most sub-Saharan Africa countries have an anaemia prevalence above 40% in young children and pregnant women, a severe public health problem according to the World Health Organization (WHO) [4]. Iron deficiency is frequently exacerbated by concomitant parasitic and bacterial enteric infections and, together, these account for the majority of anaemia cases in developing countries [5-7].

Iron supplementation with simple ferrous salts is cheap and widely available, but constitutes a non-physiological approach to providing iron that has been associated with significant side-effects and adverse events [8-14]. Data and meta-analysis from trials involving nearly ten thousand young children, mainly from resource-poor countries, have consistently shown that conventional oral iron supplements used to treat IDA are associated with increased infection, including bloody diarrhoea [9, 12, 15, 16], detrimental changes to the gut microbiome and gut inflammation [16-19]. Therefore, in countries with poor infection control, iron supplementation in young children could further increase the burden from enteric infection and environmental enteropathy (i.e. persistent gut damage and inflammation that leads to malabsorption), which is a major cause of growth failure in children in resource-poor environments and may later exacerbate the risk of IDA [20, 21].

Since 2005, we have been developing an engineered analogue of natural food iron as an alternative iron supplement. The novelty of IHAT, is that it is not a soluble compound nor does it require solubilisation in the stomach prior to uptake by the duodenal enterocytes since it is taken up as whole nanoparticles [22, 23], similarly to what has been proposed for dietary plant ferritin [24-26]. This means that the unabsorbed fraction of the compound that transits to the lower gut, which is usually at least 70% of all ingested oral iron, irrespective of the form, will remain nanoparticulate and, therefore not soluble, and as such should not be available to promote significant pathogen growth and tissue inflammation [27, 28].

Our pre-clinical and early-clinical data indicates that IHAT is effectively absorbed in humans, corrects IDA in animal models, is not redox reactive and does not have a detrimental impact on the gut microbiome [22, 27-30].

Thus the main purpose of the IHAT-GUT study is to determine whether supplementation with IHAT safely corrects IDA in young children compared to standard-of-care.

2 Study objectives

The main purpose of the IHAT-GUT study is to determine whether supplementation with IHAT safely corrects IDA in young children compared to the present standard of care. We hypothesise that 12-weeks supplementation with IHAT will correct iron deficiency and improve haemoglobin levels in young children without causing diarrhoea or inducing intestinal inflammation and detrimental changes in the gut microbiome.

The IHAT-GUT study has four primary combined objectives to test efficacy and safety of IHAT:

1. The primary objectives in terms of efficacy for this trial are to test non-inferiority of IHAT in relation to ferrous sulphate at correcting ID and improving Hb levels after 12 weeks of supplementation.
2. The primary objectives for safety are to test superiority of IHAT in relation to ferrous sulphate and non-inferiority in relation to placebo based on moderate-severe diarrhoea incidence and prevalence.

Secondary objectives of the IHAT-GUT trial are to test whether IHAT is non-detrimental with respect to enteric pathogen burden, does not increase morbidity, does not decrease abundances of *Lactobacillaceae* and *Bifidobacteriaceae* relative to *Enterobacteriaceae*, and does not cause intestinal inflammation.

2.1 Study design

The IHAT-GUT trial is a three-arm, parallel, individually randomised, placebo-controlled, double-blind study with iron supplementation in young children with mild to moderate iron deficient anaemia. Participants are children 6-35 months of age, considered generally healthy and with IDA defined as 7≤ Hb <11 g/dl and ferritin <30 µg/L [31]. Ferrous sulphate ($FeSO_4$), the current standard of care for iron supplementation, is used as the active comparator at the conventional daily dose of 12.5mg iron.

In total, approximately 600 children are randomised (1:1:1) to IHAT, $FeSO_4$ or placebo, and each arm (details in the protocol p33-35) includes an intervention period of 12 weeks starting 1 week after randomisation. The daily iron dose for IHAT is the bioequivalent to 12.5 mg elemental iron.

Randomisation is based on a stratified block design to achieve group balance in terms of age and baseline haemoglobin concentration (details in the protocol p32-34). Based on the assessments at the pre-enrolment day (Day 0), each child is categorised into two Hb classes:below or equal to/above the median Hb for the recruited cohort (as the trial recruited subjects in 3 rounds, the baseline Hb is classified as low/high based on the

median for that cohort/round) and also according to age into 3 classes; >=6 and <12 months (referred to as 6-11 months), >=12 and <24 months (referred to as 12-23 months and >=24 and <36 months (referred to as 24-35 months). This divides children into 6 different strata and in each strata the children are randomly assigned to one of the three study treatment arms (1:1:1 ratio) using a computer program and a block randomisation approach with fixed block size by age and Hb levels (a block size of six was used).

Each child has a unique intervention code which is also their study ID/randomisation number, this means that the study team is unaware of which children belong to the same treatment arm. The child study ID is the same code that is used to label the capsules for the study treatment they receive.

A flow chart of the study participant timeline is presented in Figure 2 (see Figure 5 in the protocol for more details).

On study Day 1, i.e. the first day of supplementation, each child is invited back to the study clinic and a photo is taken (with consent) for a study ID card that also contains the child's randomisation/study ID number. At this visit, demographic and immunisation data are collected and the study morbidity questionnaire is completed. A venous blood sample and a stool sample are also collected (study baseline samples). After blood collection, the field staff give the child their allocated study arm supplement.

Every day, over the following 12 weeks, the field staff visits the child at home in order to administer the allocated study supplement and to check on the child's health status. Three times per week, they complete the study morbidity questionnaire, which includes questions regarding fever, diarrhoea, vomiting, malaria symptoms, other illness, hospitalisation, appetite and medication. If a child is found unwell they are referred to the study nurse or clinician for evaluation and treatment. These check-ups continue 4 weeks post-supplementation to follow-up on adverse events.

During the study supplementation period, each week children are invited back to the study clinic for a check-up by the study nurse and a finger prick to determine their malaria and Hb status. Children found with a positive RDT at any time during the supplementation period are further tested with a blood film and treated according to The Gambian national guidelines if malaria is confirmed. Any child where Hb falls below 7 g/dL during the supplementation period discontinues the study supplementation and is offered standard-of-care oral iron treatment according to the national guidelines. These children continue to be followed up by the clinical team at the weekly clinics and will not be excluded from the data analysis but considered as treatment failures.

On study days 15 and 85, the child visits the study clinic and stool and venous blood samples are collected. Immunisation data is also recorded on these occasions. Height and weight is re-measured on study Day 85.

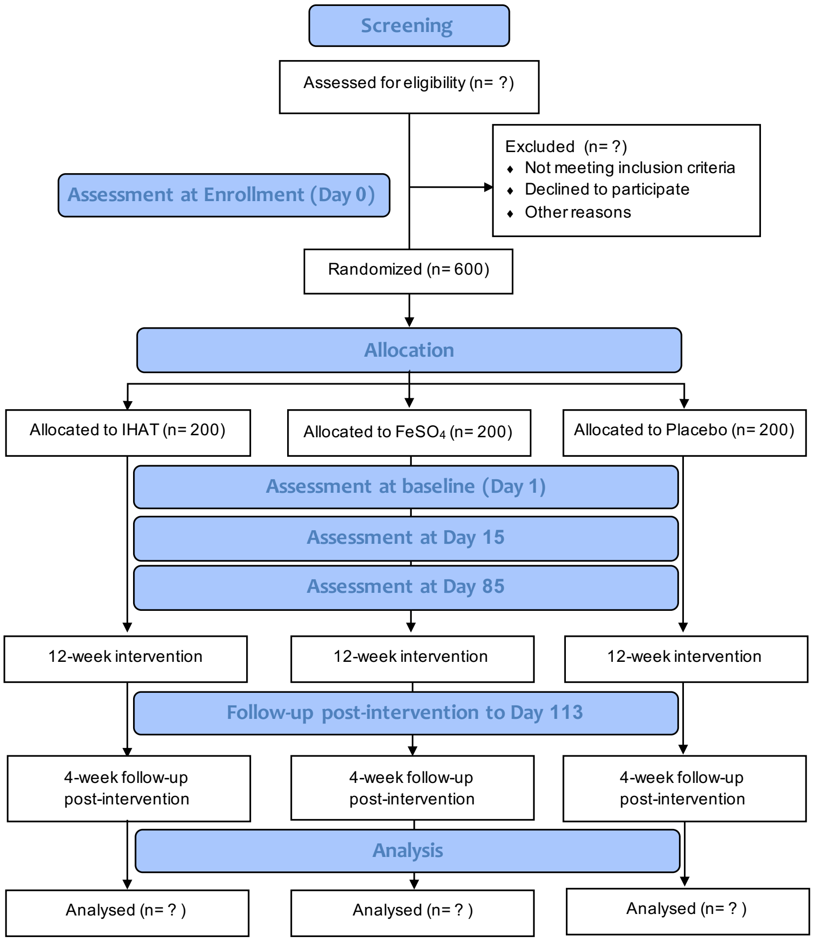

Figure 1. IHAT-GUT study flow diagram. For further details please refer to study protocol [32].

2.2 Study outcomes

All participants are assessed at three different time-points: at baseline (Day 1 of supplementation), and at 15 and 85 days after start of supplementation. The Day 15 time-point provides an indication of acute compound-related effects and the Day 85

time-point is indicative of chronic (i.e. longer term) compound-related effects, for all the 'safety' outcomes.

2.2.1 Primary

The primary **efficacy** outcome of the trial is the proportion of children in each arm who resolve iron deficiency and either achieve a normal Hb at Day 85 or show an increase of at least 1 g/dL between Day 1 and Day 85. The primary **safety** outcome is the frequency of moderate-severe diarrhoea.

Therefore, there are four primary endpoints of the trial:

(1) iron deficiency at Day 85;

(2) haemoglobin levels at Day 85;

(3) 'incidence density' of moderate-severe diarrhoea between Day 1 and Day 85;

(4) 'period prevalence' of moderate-severe diarrhoea between Day 1 and Day 85 supplementation period (i.e. the proportion of children with at least one episode of moderate-severe diarrhoea in this period).

To assess **iron deficiency**, we will use regression-adjusted ferritin concentration below 12 μg/L, where ferritin values are adjusted for inflammation using the regression model recommended by the Biomarkers Reflecting Inflammation and Nutritional Determinants of Anemia (BRINDA) group [6].

Iron deficiency and Hb levels at Day 85 will be used to assess non-inferiority of IHAT relative to $FeSO_4$ in terms of efficacy at treating IDA. We will determine the **proportion of children in each arm who resolve iron deficiency and either achieve a normal Hb (≥ 11 g/dL) or an increase in Hb of at least 1 g/dL after 12 weeks of iron supplementation.**

'Incidence density' and 'period prevalence' of moderate-severe diarrhoea will both be used to assess superiority of IHAT relative to $FeSO_4$ and non-inferiority relative to placebo for the diarrhoea outcome (i.e. safety or tolerability).

Moderate-severe diarrhoea is defined as those diarrhoea episodes where: (i) the child passes more than 5 loose or liquid stools per day, (ii) there is blood or mucus in the stool (dysentery), or (iii) the child shows signs of clinical dehydration (assessed by the study nurse based on physical signs such as little or no urination, sunken eyes, and skin that lacks its normal elasticity). **Incidence density** of moderate-severe diarrhoea is

defined as the number of new moderate-severe diarrhoea episodes per child over the 12 weeks supplementation period. **Period prevalence** of moderate-severe diarrhoea is defined as the proportion of children with at least one episode of moderate-severe diarrhoea in this period.

2.2.2 Secondary

Secondary endpoints are:

a. faecal microbiome diversity and profile (particularly in terms of abundance of *Enterobacteriaceae*, *Bifidobacteriaceae, Lactobacillaceae*) (data collected at Day 1, 15 and 85)
b. abundance of enteric pathogens (data collected at Day 1, 15, 85)
c. faecal calprotectin (marker of gut inflammation, data collected at Day 1, 15 and 85)
d. hospitalisation and morbidity (adverse event data collected throughout the study period)
e. malaria infection (data collected every week with RDT)
f. treatment failures (i.e. the number of children who have to discontinue study supplementation because their Hb falls below 7 g/dL during the intervention period)
g. 'longitudinal prevalence' of diarrhoea, i.e. the proportion of days a child has diarrhoea over the intervention period (data collected three times per week using the study questionnaire)
h. 'longitudinal prevalence' of moderate-severe diarrhoea, i.e. the proportion of days a child has moderate-severe diarrhoea over the intervention period (data collected three times per week using the study questionnaire)

i. 'incidence density' of bloody diarrhoea (i.e. the number of bloody diarrhoea episodes per child-month of observation) (data collected three times per week using the study questionnaire)
j. systemic inflammation (serum C-reactive protein (CRP) and alpha 1-acid glycoprotein (AGP)) (data collected at Day 1, 15, 85)
k. iron status and handling biomarkers (hepcidin, soluble transferrin receptor (sTfR), transferrin saturation and circulating non-transferrin bound iron - NTBI) (data collected at Day 1, 15, 85).

2.3 Study evaluations

All field data is captured in electronic case report forms (eCRF) and entered directly into handheld tablets using the Android 7.0 software and the REDCap mobile app. Data is synced daily into the database via secure Wi-Fi network.

The following data is recorded: date of informed consent, personal data (ID, initials, date of birth), socioeconomic data, height and weight, anthropometry z-scores, information on health status and regarding participation in other studies, date and time of all venous and finger prick blood collections, date and time of all faecal sample collections, date and time of supplement administration, data on morbidity, adverse events, lab results.

Details of the schedule of assessments recorded on the eCRF at each visit to the clinics are provided in the Table below.

Clinic visit	Assessments
Screening	Review of eligibility against inclusion and exclusion criteria<br>Informed consent<br>Immunisation<br>Demographics (dob, age, gender, district, village, compound, clinic/health post)<br>Height/Length<br>Weight<br>Anthropometry z-scores (WAZ, HAZ, WHZ)<br>Field Hb<br>Field malaria (RDT)<br>Lab Hb<br>Ferritin
Day 0	Field Hb<br>Field malaria (RDT)
Day 1	Field Hb<br>Field malaria (RDT)<br>Full Blood Count parameters (Medonic):<br>[Lab Hb, RBC, MCV, RDW%, RDWa, HCT, PLT, MPV, PDW, PCT, LPCR, WBC, MCH, MCHC, LYM, GRAN, MID, LYM %, GRAN %, MID %]<br>Cobas analyser parameters:<br>[AGP, CRP, Ferritin, Serum Fe, sTfR, Transferrin, UIBC, TSAT%]<br>Microbiome<br>Calprotectin<br>NTBI<br>Hepcidin(1)<br>Kato Katz parasite morphology

	Kato Katz total parasite egg count<br>Morbidity questionnaire<br>Adverse Events
Day 8	Field Hb<br>Field malaria (RDT)<br>Morbidity questionnaire<br>Adverse Events
Day 15	Field Hb<br>Field malaria (RDT)<br>Full Blood Count parameters (Medonic):<br>[Lab Hb, RBC, MCV, RDW%, RDWa, HCT, PLT, MPV, PDW, PCT, LPCR, WBC, MCH, MCHC, LYM, GRAN, MID, LYM %, GRAN %, MID %]<br>Cobas analyser parameters:<br>[AGP, CRP, Ferritin, Serum Fe, sTfR, Transferrin, UIBC, TSAT%]<br>Microbiome<br>Calprotectin<br>NTBI<br>Hepcidin(1)<br>Kato Katz parasite morphology<br>Kato Katz total parasite egg count<br>Morbidity questionnaire<br>Adverse Events
Day 22	Field Hb<br>Field malaria (RDT)<br>Morbidity questionnaire<br>Adverse Events
Day 29	Field Hb<br>Field malaria (RDT)<br>Morbidity questionnaire<br>Adverse Events
Day 36	Field Hb<br>Field malaria (RDT)<br>Morbidity questionnaire<br>Adverse Events
Day 43	Field Hb<br>Field malaria (RDT)<br>Morbidity questionnaire<br>Adverse Events
Day 50	Field Hb<br>Field malaria (RDT)<br>Morbidity questionnaire<br>Adverse Events
Day 57	Field Hb<br>Field malaria (RDT)<br>Morbidity questionnaire

	Adverse Events
Day 64	Field Hb<br>Field malaria (RDT)<br>Morbidity questionnaire<br>Adverse Events
Day 71	Field Hb<br>Field malaria (RDT)<br>Morbidity questionnaire<br>Adverse Events
Day 78	Field Hb<br>Field malaria (RDT)<br>Morbidity questionnaire<br>Adverse Events
Day 85	Field Hb<br>Field malaria (RDT)<br>Full Blood Count parameters (Medonic):<br>[Lab Hb, RBC, MCV, RDW%, RDWa, HCT, PLT, MPV, PDW, PCT, LPCR, WBC, MCH, MCHC, LYM, GRAN, MID, LYM %, GRAN %, MID %]<br>Cobas analyser parameters:<br>[AGP, CRP, Ferritin, Serum Fe, sTfR, Transferrin, UIBC, TSAT%]<br>Microbiome<br>Calprotectin<br>NTBI<br>Hepcidin(1)<br>Kato Katz parasite morphology<br>Kato Katz total parasite egg count<br>Morbidity questionnaire<br>Adverse Events
Day 113	Field Hb<br>Field malaria (RDT)<br>Morbidity questionnaire<br>Adverse Events
**Field data**	
3x per week	Morbidity questionnaire: diarrhoea, dehydration, temperature, history of fever, cough, breathing, convulsion, vomiting, appetite, illness in the last few days, concomitant medication
Every day	Supplement administration<br>Adverse events

(1) *Analysis of the hepcidin outcome is postponed until further notice due to funding constraints.*

3 Statistical considerations

3.1 Study population

Two main study populations will be considered in preparing descriptive summaries and in subsequent analyses. Subjects who have satisfied the population criteria will be classified in the designated population and will only be included in analyses for which they have available data.

Intention-to-Treat (ITT) Population

The ITT population is defined as all randomised subjects, regardless of when they withdrew from the study, or whether they fully adhered to the treatment or switched to an alternative treatment. The ITT population will be used to present all the efficacy and safety data (including the primary non-inferiority and superiority comparisons) by randomised treatment group but this will be a sensitivity analysis for non-inferiority comparisons. Subjects will be summarised according to the treatment to which they were randomised, regardless of which treatment they actually received.

The ITT population will also be used to present the exploratory treatment effect sizes for all study outcomes by randomised treatment group.

Per-protocol (PP) population

The PP population is defined as all subjects randomised to one of the three arms of the trial and who actually completed the study protocol without any major protocol deviations, having received their assigned treatment for at least 80% of the study period. This PP population only includes the subset of the ITT population who have (1) not missed more than 20% of the assigned study supplement daily doses from Day 1 to Day 85 (i.e. not missed more than 17 days of supplementation), (2) have provided study samples for the relevant study timepoints and (3) have not had any major protocol deviations (i.e. children randomised outside eligibility criteria and those protocol deviations which could have impacted on participant safety or on the scientific credibility of the trial).

The PP population will be used to present the efficacy and safety data for the primary non-inferiority comparisons by actual treatment received as assigned. As recommended by the trial DSMB, we will conduct both ITT and PP analyses for the non-inferiority hypothesis, but our primary comparisons will be conducted using the PP population.

The PP population will also be used to present the exploratory treatment effect sizes for all trial outcomes by actual treatment received as assigned.

3.2 Definitions and derived variables

3.2.1 Demographic/baseline characteristics

Age: will be calculated using the Date of Birth (DOB) and the date of recruitment/enrollment (Day 0), and presented as age at last birthday as an integer.

Age = Integer part of [(Date of Baseline visit – Date of Birth) / 30.4]

Age group: will be defined in three groups - Young, Middle, Old (6-11 months, 12-23 months and 24-35 months respectively)

Gender: will be male or female.

Anthropometry: will be defined as z-scores assessed at the screening visit - WAZ (weight-for-age), HAZ (height-for-age), and WHZ (weight-for-height)

Hb level: will be two groups - Low (below median Hb), High (equal to/above the median Hb) for each recruited cohort.

Hb x Age group strata: this will be created by all possible combinations (six) of Hb and age groups above as

a. Low Hb, young age group
b. Low Hb, middle age group
c. Low Hb, old age group
d. High Hb, young age group
e. High Hb, middle age group
f. High Hb, old age group

3.2.2 Iron deficiency

Ferritin values will be adjusted for inflammation using the regression model recommended by the Biomarkers Reflecting Inflammation and Nutritional Determinants of Anemia (BRINDA) group [6].

An adjustment to the measured ferritin value (Eq.1 and 2 below) will be made for all children with elevated C-reactive protein (CRP) and/or elevated α-1-acid-glycoprotein (AGP), i.e. for all children with indication of inflammation. Elevated CRP shall mean all cases where CRP > 0.1 mg/L and elevated AGP shall mean all cases where AGP > 0.59 g/L [6].

For children with normal CRP and AGP levels, no adjustment to the observed ferritin will be made and $Ferritin_{adjusted} = Ferritin_{observed}$.

Eq. 1 $$\ln Ferritin_{adjusted} = \ln Ferritin_{observed} - 0.19(\ln CRP_{observed} + 2.26) - 0.74(\ln AGP_{observed} + 0.52)$$

where, $\ln CRP_{reference} = -2.26$ and $\ln AGP_{reference} = -0.52$

Eq. 2 $$Ferritin_{adjusted} = \exp \hat{} (\ln Ferritin_{observed})$$

To assess **iron deficiency (ID)**, we will use this regression-adjusted ferritin concentration ($Ferritin_{adjusted}$) and consider 'ID not corrected' if $Ferritin_{adjusted} < 12$ μg/L and 'ID corrected' when $Ferritin_{adjusted} \geq 12$ μg/L.

Iron deficiency will be a binary variable (ID =1 corrected ID, ID=0 not corrected ID).

3.2.3 Haemoglobin improvement

The haemoglobin change after 85 days of study treatment will be a continuous variable calculated as the difference in the laboratory haemoglobin measurement between study Day 85 and study Day 1:

$Hb_{increase}$ = (Lab $Hb_{Day\ 85}$) – (Lab $Hb_{Day\ 1}$)

Normal haemoglobin values shall mean all Lab $Hb_{Day\ 85} \geq 11$ g/dl.

Haemoglobin improvement is a binary variable defined as = 1, IF [($Hb_{increase} \geq 1$ g/dl) OR (Lab $Hb_{Day\ 85} \geq 11$ g/dl)] and = 0, IF [($Hb_{increase} < 1$ g/dl) AND (Lab $Hb_{Day\ 85} < 11$ g/dl)].

3.2.4 Diarrhoea

Diarrhoea is defined as 3 or more loose or watery stools per day.

Dysentery or bloody diarrhoea is defined as diarrhoea where there is blood in the stools.

Mild diarrhoea is defined as diarrhoea with 3 or 4 loose or watery stools per day, no signs of dehydration, no dysentery and no mucus in stool.

Moderate diarrhoea is defined as diarrhoea and 5 or more loose or watery stools per day, or diarrhoea with some signs of dehydration, in both cases without dysentery or mucus in stool.

Severe diarrhoea is defined as diarrhoea with severe dehydration, or dysentery or mucus in stool.

Infectious diarrhoea is defined as cases of diarrhoea with dysentery or mucus in stool and where the child has fever.

3.2.5 Treatment failures

Treatment failures are defined as those children who are discontinued from the study supplementation because their Hb falls below 7 g/dl (severe anaemia) at any point during the study supplementation period with assessments conducted between Day 8 and Day 85. These children won't be included in the PP analysis if they received less than 80% of their assigned treatment at the time of discontinuing the study supplementation. Treatment failure is also a separate outcome variable.

3.2.6 Malaria

Malaria infection cases are defined as children with a positive field RDT and laboratory confirmation of presence of parasites with duplicate blood films.

3.3 Primary Efficacy Endpoint

3.3.1 Efficacy for IDA correction

We define 'response' for IDA as correction of iron deficiency and either achieving a normal Hb or an increase of at least 1 g/dL after 12 weeks of iron supplementation.

Therefore, to determine the efficacy primary endpoint for IDA correction we will determine the proportion of children who at the Day 85 study timepoint achieve:

(ID corrected, *as defined in 3.2.2 above*) AND (Haemoglobin improvement, *as defined in 3.2.3*)

3.4 Primary Safety Endpoints

3.4.1 'Incidence density' of moderate-severe diarrhoea

'*Incidence density'* of moderate-severe diarrhoea is defined as the number of new moderate or severe diarrhoea episodes per child over the 85 days intervention.

3.4.2 'Period prevalence' of moderate-severe diarrhoea

'*Period prevalence'* of moderate-severe diarrhoea is defined as the proportion of children with at least one episode of moderate or severe diarrhoea over the 85 days intervention.

3.5 Secondary Efficacy Endpoints

3.5.1 Treatment failures

We will determine the proportion of children in each arm defined as treatment failures as per section 3.2.5.

3.5.2 Iron status

We will calculate the sTfR/$\log_{10}$ferritin (sTfR-F) index and determine the proportion of children in each arm with a normal iron status defined as sTfR-F ≤ 2 [31] at the study Day 85.

We will also determine the proportion of children in each arm with normal alternative biomarkers of iron status at study Day 85 based on the secondary endpoints for other single iron handling indicators:

Soluble transferrin receptor:	sTfR > 8.3 mg/L [6]
Mean corpuscular volume:	MCV< 75 fl
Mean corpuscular haemoglobin:	MCH < 25 pg

We will determine the proportion of children in each arm who remain IDA at study Day 85 based on the current WHO recommendations of Hb < 11 g/dl and $\text{Ferritin}_{unadjusted} < 30$ μg/L, for populations with high burden of infection and inflammation [33].

3.6 Secondary Safety Endpoints

3.6.1 'Longitudinal prevalence' of diarrhoea

'*Longitudinal* prevalence' of diarrhoea of any kind is defined as the proportion of days a child has diarrhoea over the total duration of the 85 days intervention period.

3.6.2 'Longitudinal prevalence' of moderate-severe diarrhoea

'*Longitudinal* prevalence' of moderate-severe diarrhoea is defined as the proportion of days a child has moderate or severe diarrhoea over the total duration of the 85 days intervention period.

3.6.3 'Incidence density' of bloody diarrhoea

'*Incidence density'* of bloody diarrhoea is defined as the number of new dysentery <u>or</u> mucus diarrhoea episodes per child-month of follow-up.

3.6.4 Malaria infection

We will determine the proportion of children in each arm with confirmed malaria as per 3.2.6.

3.6.5 Acute respiratory infection (ARI)

We will determine the proportion of children in each arm with ARI reported as an adverse event.

3.7 Handling of missing data

The extent of missing data will be assessed by treatment group and covariates including age group and Hb level. By design, the burden of missing data in this trial will be more on the outcome rather than baseline covariates. Thus, we will perform sensitivity analyses relying on analytical techniques irrespective of the extent of missing data. The main analytical technique that will be considered is adjusting the models for covariates for which balance could be affected by missing outcome data. Multiple imputation will be used to account for missing data if there are missing covariates in more than 5% of participants.

3.8 Site/Village/Region specific analysis

No site specific analysis will be performed.

3.9 Safety parameters

Adverse events will be summarised per treatment group:

- Number of all adverse events (AEs)
- Number of all serious adverse events (SAEs)
- Number of events per main diagnosis category (ARI, Asthma, Acute Ear Infection, Chronic Ear Infection, Skin Lesions, Infected Skin Lesions, Burns, Infected Burns, Abscesses/Soft Tissue Infections, Sepsis, Fever, Vomiting, Poor Appetite, Diarrhoea with No dehydration, Diarrhoea with Some Dehydration, Diarrhoea with Severe Dehydration, Persistent Diarrhoea, Dysentery, Oral Sores/Gingivitis, Abdominal pain, Measles, Chicken pox, Conjunctivitis, Trauma, Malaria, UTI, Worms, Other)
- Duration of the AE/SAE in days (median, range)
- Number of subjects with AEs
- Maximum number of AE per subject
- Number of subjects with SAEs
- Type of SAE (death, hospitalisation)
- Number of AEs by severity (mild, moderate, severe, life-threatening)
- Number of AEs by relatedness to study treatment (definitely unrelated, unlikely, possible, probable, definitely related)
- Subjects with AEs by relatedness to study treatment (definitely unrelated, unlikely, possible, probable, definitely related)
- Events leading to discontinuation of study treatment

3.10 Sample size determination

The hypotheses being tested in this study are:

1) non-inferiority of IHAT compared to ferrous sulphate for correction of iron deficiency and either achieving a normal Hb or an increase of at least 1 g/dL after 12 weeks of iron supplementation. A non-inferiority margin of 0.1 is used. The null hypothesis is therefore the probability in the IHAT arm minus the response probability in the ferrous sulphate arm is less than or equal to – 0.1.

2) superiority of IHAT compared to ferrous sulphate in terms of incidence density of moderate-severe diarrhoea. Here the null hypothesis is that the mean number of new episodes in the IHAT arm is greater than or equal to the mean number in the ferrous sulphate arm.

3) superiority of IHAT compared to ferrous sulphate in terms of prevalence of moderate-severe diarrhoea. Here the null hypothesis is that the prevalence of diarrhoea in the IHAT arm is greater than or equal to the prevalence in the ferrous sulphate arm.

4) non-inferiority of IHAT compared to placebo in terms of prevalence of moderate-severe diarrhoea. Here the null hypothesis is that the prevalence of diarrhoea in the placebo arm minus the prevalence in the IHAT arm is less than or equal to – 0.1.

Because this is a pilot study, with any significant results being retested in a subsequent pivotal study, we do not adjust for multiple testing. Each hypothesis is to be tested at a 10% one-sided type I error rate.

The sample size (600 in total, equally randomised between the three arms) provides high power for each of the four hypotheses:

1) 89% power to test non-inferiority on the IDA response probability, with a non-inferiority margin of 0.1 (0.583 on the odds ratio scale) assuming that the true response probability is 0.3 on IHAT and ferrous sulphate [9, 12, 40] arms;
2) 90% power to test superiority for the incidence density of moderate-severe diarrhoea outcome assuming IHAT provides a 20% reduction in the mean for ferrous sulphate (i.e. from 1.28 episodes per child over the 12 weeks supplementation period to 1.02; calculations based on unpublished data for studies with iron supplements in rural Gambia);
3) 90% power to test reduction in prevalence of moderate-severe diarrhoea from 25% in the ferrous sulphate arm to 15% in the IHAT arm;

4) 93% power to test non-inferiority (0.1 non-inferiority margin, 0.583 on the odds ratio scale) of IHAT against placebo for prevalence of moderate-severe diarrhoea when both have 15% prevalence. For these calculations, we used published diarrhoea period prevalence data from studies with iron supplementation [12, 16].

For the secondary outcomes, the trial (n=200 per arm) will have over 85% power to detect significant differences between all the arms in terms of enterobacteria [16], NTBI [38] and calprotectin [16].

To account for a non-completion rate of 15%, the total number of children we plan to enrol in the study is n=705.

3.11 Statistical analysis

In this section, the general approach for the statistical analyses is briefly outlined. More specific analysis methods for each endpoint and related tables for results is presented in subsequent sections.

Summary statistics will be produced for all by treatment group subjects are randomised to and overall for all study participants. For safety indicators, the summary will be produced by actual treatment received. Summary of data will also be presented by visit day where appropriate in addition to treatment group.

For continuous variables, the mean (arithmetic), standard deviation (SD), median, the 25th and 75th percentiles, the minimum and maximum statistics will be presented.

For categorical variables, counts and percentages will be used. Unless otherwise specified, the denominator for each percentage will be the number of subjects overall or within the population treatment group.

All comparative analyses for the primary, secondary and exploratory outcomes will in general be based on the generalized linear models. That is, logistic, Poisson and linear regressions will be used for binary, count and continuous outcomes respectively. Table 1 shows a brief summary of the type of comparison along with the analytical method used for the primary objectives.

Table 1: Summary of the four primary comparisons the trial.

Groups compared	Outcome	Comparison	Population	Method	Decision
IHAT Vs FeSO4	IDA correction/ response probability	Non-inferiority	ITT, PP	Logistic regression	Declare non-inferiority if lower limit of 90% one-sided CI for OR >0.583.
IHAT Vs FeSO4	Incidence density of diarrhoea	Superiority	ITT	Poisson regression	Declare superiority if the one-sided p-value for the Wald test of the effect of IHAT is less than 0.1.
IHAT Vs FeSO4	Prevalence of diarrhoea	Superiority	ITT	Logistic regression	Declare superiority if the one-sided p-value for the Wald test of the effect of IHAT is less than 0.1.
Placebo Vs IHAT	Prevalence of diarrhoea	Non-inferiority	ITT, PP	Logistic regression	Declare non-inferiority if lower limit of 90% one-sided CI for OR >0.583.

The odds ratio (OR), rate ratio (RR) and mean difference estimates between treatment arms, CIs and p-values will be presented.

All hypotheses testing for non-inferiority and superiority will be carried out at 10% (one-sided) significance level unless otherwise specified. P-values will be rounded to three decimal places. P-values less than 0.001 will be reported as <0.001 in tables. P-values greater than 0.999 will be reported as >0.999. The data listings will be sorted by treatment group and subject number.

After fitting each proposed model, we will check adequacy of each model using regression diagnostics. For linear regression models, we will check normality of residuals and use transformation if they show gross departure from normality. If count data show

over-dispersion, we will fit quasi-Poisson or negative binomial models instead of Poisson regression and Bayesian information criteria (BIC) will be used to decide the best fitting model.

In addition to the primary analyses in the trial protocol, further exploratory analyses including but not limited to those listed in section 3.11.2.6 may be conducted later. All statistical analysis will be performed using R and STATA® Packages.

3.11.1Descriptive analysis

3.11.1.1 Subject Disposition

For each of the three cohorts, subjects' enrolment history will be summarised using a CONSORT diagram. This will show the number of subjects screened, subjects consented, subjects called for day 0, subjects enrolled and randomised, subject numbers at each of the key follow up dates (day 1, day 15, day 85 and day 113), and number of subjects analysed for the ITT, PP and AT populations. Information on the reasons for not completing the study and exclusion/screen failures in will also be presented.

NEED BASELINE CHARACTERISTICS TABLE BEFORE GOING ON TO RETENTION

Expected and actual enrolment numbers as well as consent rates per week of screening for each of the three cohorts will be presented using plots.

Overall subject status summary will be tabulated providing numbers of subjects enrolled and randomised, attended visits on days 1, 15, 85 and 113, completed the study, (113 days follow-up), voluntary withdrawal, any deaths, lost to follow up (Day 85) and discontinued supplementation due to Hb < 7 g/dL.

Table 2. Study retention

	N (%)
Day 0 (enrolment visit)	
Day 1	
Day 85	
Day 113	
Lost to follow up	

Deaths	
Withdrawals	

Table 3. Study retention by study arm

	**IHAT N (%)**	**FeSO4 N (%)**	**Placebo N (%)**
Day 0 (enrolment visit)			
Day 1			
Day 85			
Day 113			
Lost to follow up			
Deaths			
Withdrawals			

Table 4. Study retention by study arm, Hb level (low/high) and age group (young/middle/old)

	**IHAT N (%)**						**FeSO4 N (%)**						**Placebo N (%)**					
	G1	G2	G3	G4	G5	G6	G1	G2	G3	G4	G5	G6	G1	G2	G3	G4	G5	G6
Day 0																		
Day 1																		
Day 85																		
Day 113																		
Lost to follow up																		
Deaths																		
Withdrawal																		

G1(Low, Young),G2(Low, Middle),G3(Low, Old),G4(High, Young),G5(High, Middle),G6(High, Old)

Table 5. List of study withdrawals

**Participant ID**	**Time of withdrawal (Study Day)**	**Reason for withdrawal**

Table 6. List of study withdrawals by study arm

Participant ID	Time of withdrawal (Study Day)	Reason for withdrawal
**IHAT**		
**FeSO4**		
**Placebo**		

Table 7. Number of subjects complying with daily doses*

Compliance with daily doses over time (days)	Randomised			Overall
	IHAT N (%)	$FeSO_4$ N (%)	Placebo N (%)	
28				
49				
85				

* the number and % of subjects in each group who have taken 1 month (28 days), 2 month (49 days) and 3 months (85 days) worth of daily doses.

Table 8. Summary of daily doses taken and consecutive daily doses missed by subjects in each arm

Doses	IHAT			$FeSO_4$			Placebo		
	N (%)	Median (IQR)	Range (Min, Max)	N (%)	Median (IQR)	Range (Min, Max)	N (%)	Median (IQR)	Range (Min, Max)
Taken									
Missed									

3.11.1.2 Demographics and baseline characteristics

Demographic and baseline characteristics of all children enrolled in the study will be tabulated as shown in the tables 9 and 10 below.

Table 9. Characteristics of the study participants at baseline

**Characteristic**	**N (%)**	**Mean (SD)**	**Median (IQR)**	**Range (Min, Max)**
Age (months)				
Age group				
6-11mo				
12-23mo				
24-35mo				
Gender				
Female				
Male				
Weight (kg)				
Height (cm)				
HAZ				
WAZ				
WHZ				
Hb (g/dl)				
Hb group				
Low				
High				
HbXAge group strata				

Low, Young				
Low, Middle				
Low, Old				
High, Young				
High, Middle				
High, Old				
High, Old				
Ferritin (µg/L)				
Health facility cluster				
1 Yorrobawol				
2 Taibatu				
3 Darsilami				
4 Kuwonkuba				
5 Chamoi Bunda				

Table 10. Characteristics of the study participants at baseline, by treatment group

Characteristic	IHAT				$FeSO_4$				Placebo			
	N(%)	Mean (SD)	Median (IQR)	Range (Min, Max)	N (%)	Mean (SD)	Median (IQR)	Range (Min, Max)	N (%)	Mean (SD)	Median (IQR)	Range (Min, Max)
Age (months)												
Age group												
6-11mo												
12-23mo												
24-35mo												
Gender												
Female												
Male												
Weight (kg)												
Height (cm)												
HAZ												
WAZ												
WHZ												
Hb (g/dl)												
Hb group												
Low												
High												
HbXAge group strata												
Low, Young												
Low, Middle												
Low, Old												

High, Young											
High, Middle											
High, Old											
High, Old											
Ferritin (µg/L)											
Health facility cluster											
1 Yorrobawol											
2 Taibatu											
3 Darsilami											
4 Kuwonkuba											
5 ChamoiBunda											

3.11.1.3 Primary efficacy summary

Table 11. Baseline and final Hb and ferritin by randomization arm

	Group		
**Baseline-Day 1**	IHAT	$FeSO_4$	Placebo
Iron deficiency anaemia (IDA)[1]			
Iron deficiency[2]			
Mean Hb (95% CI)			
Mean Ferritin (95% CI)			
Mean Ferritin$_{adjusted}$[2] (95% CI)			
**Final-Day 85**			
Iron deficiency anaemia (IDA)[1]			
Iron deficiency[2]			
Mean Hb (95% CI)			
Mean Ferritin (95% CI)			
Mean Ferritin$_{adjusted}$[2] (95% CI)			

1. *As defined in 3.5.2 above (WHO recommendation)*
2. *As defined in 3.2.2 above (using the BRINDA recommendation for inflammation adjusted ferritin)*

3.11.1.4 Primary safety summary

Table 12. Frequency of moderate and severe diarrhoea by treatment

**Diarrhoea**	**Randomised**			**Overall**
	IHAT	$FeSO_4$	Placebo	
Moderate, N (%)				
Severe, N (%)				
Duration in days, Median (Minimum-Maximum)				
Proportion of days with Diarrhoea, %				

3.11.1.5 Other safety measures

Safety will additionally be assessed through summary of adverse events and compliance with study treatment. Safety data will be summarised for all subjects and evaluated based data on adverse events, serious adverse events, the relatedness of events to study treatment, events leading to discontinuation of treatment, malaria, treatment failures, any deaths and compliance with study treatment. These parameters will be summarised appropriately by study arm. All protocol deviations, violations and non-compliance will also reported.

Table 13. Summary of all adverse events for subjects by treatment arm

Topics	Randomised			Overall
	IHAT	$FeSO_4$	Placebo	
**Number of AEs reported**				
**Number of Subjects with AEs [1]**				
**Duration of AEs in days (Median, Min-Max)**				
**Maximum AEs number per subject**				
**Number of SAEs reported**				
**Number of Subjects with SAEs [1]**				
**Number of AEs by Severity*, N (%)**				
Mild				
Moderate				
Severe				
Life-threatening				
**Subjects with AEs by Severity [2]****				

Mild				
Moderate				
Severe				
Life-threatening				
**CAUSALITY**				
**Number of AEs by Relatedness to Treatment*, N (%)**				
Definitely unrelated				
Unlikely				
Possible				
Probable				
Definitely related				
**Subjects with AEs by Relatedness to Treatment [2]****				
Definitely unrelated				
Unlikely				
Possible				
Probable				
Definitely related				

[1] Subjects who experience one or more AEs or SAEs are counted only once.
[2] Subjects are counted only once within a particular severity grade or relatedness category.
*Percentages are based on total number of AEs reported.
**Percentages are based on total number of subjects followed-up.

Table 14. Summary of adverse events (main diagnosis) by treatment arm

**AE diagnosis**	**Randomized**			**Overall**
	**IHAT N (%)**	**$FeSO_4$ N (%)**	**Placebo N (%)**	

ARI				
Acute Ear Infection				
Chronic Ear Infection				
Skin Lesions				
Infected Skin Lesions				
Burns				
Infected Burns				
Abscesses/Soft Tissue Infections				
Fever				
Vomiting				
Poor Appetite				
Diarrhoea with No dehydration				
Diarrhoea with Some Dehydration				
Diarrhoea with Severe Dehydration				
Persistent Diarrhoea				
Dysentery				
Oral Sores/Gingivitis				
Abdominal pain				
Measles				
Chicken pox				
Conjunctivitis				
Trauma				
Malaria				
UTI				
Worms				
Other				

Table 15. Summary of subjects and severity of adverse events by main diagnosis

AE diagnosis	Total N	Total N=Mild (%)*	Total N=Moderate (%)*	Total N=Severe (%)*	Total Subjects (%)**
ARI					
Acute Ear Infection					
Chronic Ear Infection					
Skin Lesions					

Infected Skin Lesions					
Burns					
Infected Burns					
Abscesses/Soft Tissue Infections					
Fever					
Vomiting					
Poor Appetite					
Diarrhoea with No dehydration					
Diarrhoea with Some Dehydration					
Diarrhoea with Severe Dehydration					
Persistent Diarrhoea					
Dysentery					
Oral Sores/Gingivitis					
Abdominal pain					
Measles					
Chicken pox					
Conjunctivitis					
Trauma					
Malaria					
UTI					
Worms					
Other					

*Percentage in relation to total number of adverse events of that particular diagnostics.
**Number of individual participants experiencing a certain type of adverse event where each participant is counted only within a particular diagnosis.

Table 16. Summary of all serious adverse events for subjects

Subject ID	Treatment	Age	Onset Date	Stop Date	Study Day	Relationship to Intervention*	Outcome**	Description of SAE

*Definitely related, Probable, Possible, Unlikely, Definitely unrelated
**(Recovered, without treatment; Recovered, with treatment; Still Present, no treatment; Still Present, being treated; Residual effect(s) present – no treatment; Residual effect(s) present- being treated; Subject died

Table 17. Protocol deviations in the study

Protocol Deviation	Major/Minor*	Total (unique IDs)	% of total events**
**Total # of Deviations**			
**Participants Enrolled**			
**Participants with deviations**			

*A deviation is major if it has an impact on the conduct of the trial, credibility of the data or safety of participants. All other deviations are considered as minor.
**Percentage relative to total number of events of the specified type for all enrolled subjects.

3.11.1.6 Other secondary outcomes

Table 18. Summary of other secondary outcomes

**Outcome**	**Group**		
	IHAT	$FeSO_4$	Placebo
Continuous, Median (IQR)			
**Microbiome**			
Bifidobacteriaceae			
Lactobacillaceae			
Enterobacteriaceae			
(Bifidobacteriaceae + Lactobacillaceae)/ Enterobacteriaceae			
**Iron Status**			
sTfR/log10ferritin ≤ 2			
IDA WHO (Hb<11 g/dl and $Ferritin_{unadjusted}$ < 30 μg/l)			
MCV < 75 fl			
MCH < 25 pg			
sTfR > 8.3 mg/L			
Anaemia (Hb<11 g/dl)			
Iron deficiency WHO ($Ferritin_{unadjusted}$ < 30 μg/l)			
Iron deficiency inflammation-adjusted ($Ferritin_{adjusted}$ < 12 μg/l)			
Circulating non-transferrin bound iron - NTBI			
**Inflammation**			
Serum C-reactive protein (CRP)			
Alpha 1-acid glycoprotein (AGP)			
Faecal calprotectin (marker of gut inflammation)			
Categorical, N (%)			
The proportion of days a child has diarrhoea over the intervention period ('longitudinal prevalence' of diarrhoea)			
The proportion of days a child has moderate-severe diarrhoea over the intervention period ('longitudinal prevalence' of moderate-severe diarrhoea)			

'Incidence density' of bloody diarrhoea (i.e. The number of bloody diarrhoea episodes per child-month of observation)			
Treatment failures (i.e. The number of children who have to discontinue study supplementation because their hb falls below 7 g/dl)			
Hospitalisation			
Malaria infection			
ARI			

3.11.2 Comparative analysis

3.11.2.1 Primary objective 1

The first primary outcome, IDA correction/response probability, is compared for non-inferiority for IHAT Vs $FeSO_4$ supplementation using a logistic regression model. The number and proportion of subjects who achieved IDA correction after 12 weeks will be summarised by treatment group.
Both crude and adjusted OR for achieving IDA correction after 12 weeks of IHAT Vs FeSO4 supplementation will be reported with the 90% one-sided confidence interval. The models for calculating the crude and adjusted ORs are specified respectively as

IDA correction = Treatment arm
IDA correction = Treatment arm + Hb(low/high)XAge(young/middle/old) strata
where IDA correction (Yes/No) is the response as defined in section 3.2.2. Non-inferiority will be declared if lower limit of 90% one-sided CI for the OR from the adjusted model is greater than 0.583.

Table 19. IDA correction/response probability comparing IHAT Vs $FeSO_4$-primary endpoint

	Group

	IHAT	$FeSO_4$	P-value
IDA correction/response probability (%)			
OR (90% CI)- Crude		Reference	
OR (90% CI)- Adjusted*		Reference	

*Adjusted for age and Hb groups

3.11.2.2 Primary objective 2

The second primary outcome, incidence density of moderate-severe diarrhoea, is compared for superiority of IHAT Vs FeSO4 using a Poisson regression model. The incidence density over the 12 weeks intervention will be summarised by treatment group. Crude and adjusted RRs comparing IHAT Vs FeSO4 supplementation will be reported along with the 90% confidence interval. The models for calculating the crude and adjusted RRs are specified respectively as

Incidence = Treatment arm
Incidence = Treatment arm + Hb(low/high)XAge(young/middle/old) strata
where incidence is defined as the number of new moderate-severe diarrhoea episodes per child over the 12 weeks intervention as defined in section 3.4.1. Superiority will be declared if the one-sided p-value for the Wald test of the effect of IHAT from the adjusted model is less than 0.1. If the count data appear to be over-dispersed than implied by the Poisson distribution, quasi-Poisson or negative binomial models will be considered.

Table 20. Incidence density of moderate-severe diarrhoea comparing IHAT Vs $FeSO_4$-primary endpoint

	Group		
	IHAT	$FeSO_4$	P-value
Incidence density of moderate-severe diarrhoea			
RR (90% CI)- Crude		Reference	
RR (90% CI)- Adjusted		Reference	

3.11.2.3 Primary objective 3

The third primary outcome, period prevalence of moderate-severe diarrhoea, is compared for superiority of IHAT Vs FeSO4 using a logistic regression model. The number and proportion of subjects with at least one episode of moderate-severe diarrhoea over the 12 weeks of intervention will be summarised by treatment group.

Crude and adjusted ORs comparing IHAT Vs FeSO4 supplementation will be reported along with the 90% confidence interval. The models for calculating the crude and adjusted ORs are specified respectively as

Diarrhoea = Treatment arm
Diarrhoea = Treatment arm + Hb(low/high)XAge (young/middle/old) strata
where Diarrhoea (Yes/No) is the response as defined in section 3.4.2. Superiority will be declared if the one-sided p-value for the Wald test of the effect of IHAT from the adjusted model is less than 0.1.

Table 21. Prevalence of moderate-severe diarrhoea comparing IHAT Vs $FeSO_4$ -primary endpoint

	Group		
	IHAT	$FeSO_4$	P-value
Prevalence of diarrhoea (%)			
OR (90% CI)- Crude		Reference	
OR (90% CI)- Adjusted		Reference	

3.11.2.4 Primary objective 4

The outcome is the same as the third primary outcome above, period prevalence of diarrhoea. However, the comparison here is non-inferiority of IHAT Vs Placebo and this will be examined using a logistic regression model. Summary will be produced by treatment group for the number and proportion of subjects with at least one episode of moderate-severe diarrhoea over the 12 weeks of intervention. The same model specification as in the previous section will be used to calculate the crude and adjusted ORs along with the 90% confidence interval. Non-inferiority will be declared if lower limit of 90% one-sided CI for the OR from the adjusted model is greater than 0.583.

Table 22. Prevalence of moderate-severe diarrhoea comparing IHAT Vs Placebo-primary endpoint

	Group		
	IHAT	Placebo	P-value
Prevalence of diarrhoea (%)			
OR (90% CI)- Crude		Reference	
OR (90% CI)- Adjusted		Reference	

3.11.2.5 Secondary analysis

The main secondary analyses from this trial are summarised in table 23 below. For all the outcomes listed, IHAT Vs $FeSO_4$ and/or IHAT Vs placebo and/or $FeSO_4$ Vs placebo will be compared using the appropriate regression model with the treatment arm and age and Hb strata as covariates. The relevant summary statistics for all the outcomes will be produced by treatment group. Both the crude and adjusted mean differences, RRs or ORs between the treatments groups compared will be presented along with the 90% CI.

Table 23: Summary of comparisons for the secondary outcomes

Outcome	Groups compared	Population	Method
Continuous			
(Bifidobacteriaceae + Lactobacillaceae)/ Enterobacteriaceae	H_0: IHAT= Placebo<br>H_A: IHAT≠ Placebo<br><br>H_0: IHAT=$FeSO_4$<br>H_A: IHAT> $FeSO_4$	ITT, PP	Linear regression
Faecal calprotectin (marker of gut inflammation)	H_0: IHAT= Placebo<br>H_A: IHAT≠ Placebo<br><br>H_0: IHAT= $FeSO_4$<br>H_A: IHAT< $FeSO_4$	ITT, PP	Linear regression
The proportion of days a child has diarrhoea over the intervention period (‘longitudinal prevalence’ of diarrhoea)	H_0: IHAT= Placebo<br>H_A: IHAT≠ Placebo<br><br>H_0: IHAT= $FeSO_4$<br>H_A: IHAT< $FeSO_4$	ITT, PP	Linear regression
The proportion of days a child has moderate-severe diarrhoea over the intervention period (‘longitudinal prevalence’ of moderate-severe diarrhoea)	H_0: IHAT=Placebo<br>H_A: IHAT≠ Placebo<br><br>H_0: IHAT= $FeSO_4$<br>H_A: IHAT< $FeSO_4$	ITT, PP	Linear regression
Serum C-reactive protein (CRP)	H_0: IHAT= Placebo<br>H_A: IHAT≠ Placebo<br><br>H_0: IHAT= $FeSO_4$<br>H_A: IHAT< $FeSO_4$	ITT, PP	Linear regression
Alpha 1-acid glycoprotein (AGP)	H_0: IHAT= Placebo<br>H_A: IHAT≠ Placebo	ITT, PP	Linear regression

	H_0: IHAT= $FeSO_4$<br>H_A: IHAT< $FeSO_4$		
Circulating non-transferrin bound iron (NTBI)	H_0: IHAT= Placebo<br>H_A: IHAT≠ Placebo<br><br>H_0: IHAT= $FeSO_4$<br>H_A: IHAT< $FeSO_4$	ITT, PP	Linear regression
Count/Binary			
'Incidence density' of bloody diarrhoea (i.e. The number of bloody diarrhoea episodes per child-month of observation)	H_0: IHAT= Placebo<br>H_A: IHAT≠ Placebo<br><br>H_0: IHAT= $FeSO_4$<br>H_A: IHAT< $FeSO_4$	ITT, PP	Poisson regression
Hospitalisation/morbidity<br>• Hospitalisation<br>• ARI<br>• Other infections<br>• Diarrhoea<br>• Fever	H_0: IHAT= Placebo<br>H_A: IHAT≠ Placebo<br><br>H_0: IHAT= $FeSO_4$<br>H_A: IHAT< $FeSO_4$	ITT, PP	Poisson regression
Malaria infection	H_0: IHAT= Placebo<br>H_A: IHAT≠ Placebo<br><br>H_0: IHAT= $FeSO_4$<br>H_A: IHAT≠ $FeSO_4$	ITT, PP	Poisson regression
Treatment failures (i.e. The number of children who have to discontinue study supplementation because their hb falls below 7 g/dl)	H_0: IHAT=Placebo<br>H_A: IHAT<Placebo<br><br>H_0: $FeSO_4$= Placebo<br>H_A: $FeSO_4$<Placebo<br><br>H_0: IHAT= $FeSO_4$<br>H_A: IHAT< $FeSO_4$	ITT, PP	Logistic regression

Table 24: Comparisons by treatment arms for the secondary outcomes

Outcome	Hypotheses	Comparison	Effect[a] (90%CI)		P-value (adjusted)
			Crude	Adjusted	
Continuous					
(Bifidobacteriaceae + Lactobacillaceae)/ Enterobacteriaceae	H_0: IHAT= Placebo<br>H_A: IHAT≠ Placebo	IHAT Vs placebo			
	H_0: IHAT=$FeSO_4$<br>H_A: IHAT> $FeSO_4$	IHAT Vs $FeSO_4$			
Faecal calprotectin	H_0: IHAT= Placebo<br>H_A: IHAT≠ Placebo	IHAT Vs placebo			
	H_0: IHAT= $FeSO_4$<br>H_A: IHAT< $FeSO_4$	IHAT Vs $FeSO_4$			
The proportion of days a child has diarrhoea	H_0: IHAT= Placebo<br>H_A: IHAT≠ Placebo	IHAT Vs placebo			
	H_0: IHAT= $FeSO_4$<br>H_A: IHAT< $FeSO_4$	IHAT Vs $FeSO_4$			
The proportion of days a child has moderate-severe diarrhoea	H_0: IHAT=Placebo<br>H_A: IHAT≠ Placebo	IHAT Vs placebo			
	H_0: IHAT= $FeSO_4$<br>H_A: IHAT< $FeSO_4$	IHAT Vs $FeSO_4$			
Serum C-reactive protein (CRP)	H_0: IHAT= Placebo<br>H_A: IHAT≠ Placebo	IHAT Vs placebo			
	H_0: IHAT= $FeSO_4$<br>H_A: IHAT< $FeSO_4$	IHAT Vs $FeSO_4$			
Alpha 1-acid glycoprotein (AGP)	H_0: IHAT= Placebo<br>H_A: IHAT≠ Placebo	IHAT Vs placebo			
	H_0: IHAT= $FeSO_4$<br>H_A: IHAT< $FeSO_4$	IHAT Vs $FeSO_4$			
Circulating non-transferrin bound iron (NTBI)	H_0: IHAT= Placebo<br>H_A: IHAT≠ Placebo	IHAT Vs placebo			
	H_0: IHAT= $FeSO_4$<br>H_A: IHAT< $FeSO_4$	IHAT Vs $FeSO_4$			
Count					
'Incidence density' of bloody diarrhoea*	H_0: IHAT= Placebo<br>H_A: IHAT≠ Placebo	IHAT Vs placebo			
	H_0: IHAT= $FeSO_4$<br>H_A: IHAT< $FeSO_4$	IHAT Vs $FeSO_4$			
Binary					
Hospitalisation**	H_0: IHAT= Placebo<br>H_A: IHAT≠ Placebo	IHAT Vs placebo			
	H_0: IHAT= $FeSO_4$<br>H_A: IHAT< $FeSO_4$	IHAT Vs $FeSO_4$			
ARI**	H_0: IHAT= Placebo<br>H_A: IHAT≠ Placebo	IHAT Vs placebo			

	H_0: IHAT= $FeSO_4$<br>H_A: IHAT< $FeSO_4$	IHAT Vs $FeSO_4$			
Other infections**	H_0: IHAT= Placebo<br>H_A: IHAT≠ Placebo	IHAT Vs placebo			
	H_0: IHAT= $FeSO_4$<br>H_A: IHAT< $FeSO_4$	IHAT Vs $FeSO_4$			
Diarrhoea**	H_0: IHAT= Placebo<br>H_A: IHAT≠ Placebo	IHAT Vs placebo			
	H_0: IHAT= $FeSO_4$<br>H_A: IHAT< $FeSO_4$	IHAT Vs $FeSO_4$			
Fever**	H_0: IHAT= Placebo<br>H_A: IHAT≠ Placebo	IHAT Vs placebo			
	H_0: IHAT= $FeSO_4$<br>H_A: IHAT< $FeSO_4$	IHAT Vs $FeSO_4$			
Malaria infection**	H_0: IHAT= Placebo<br>H_A: IHAT≠ Placebo	IHAT Vs placebo			
	H_0: IHAT= $FeSO_4$<br>H_A: IHAT≠ $FeSO_4$	IHAT Vs $FeSO_4$			
Treatment failures**	H_0: IHAT=Placebo<br>H_A: IHAT<Placebo	IHAT Vs placebo			
	H_0: $FeSO_4$= Placebo<br>H_A: $FeSO_4$<Placebo	IHAT Vs $FeSO_4$			
	H_0: IHAT= $FeSO_4$<br>H_A: IHAT< $FeSO_4$	$FeSO_4$ Vs placebo			

[a]Effect is mean difference, RR (marked *) or OR (marked **).

3.11.2.6 Exploratory analysis

We will examine the effect of iron supplementation on soil-transmitted helminths (STH) infection and the association of specific bacteria groups of the gut microbiome (e.g. enterobacteria) with STH. In relation to the microbiome data analysis, we will work together with our collaborators in the Sanger Institute and use the most up to date bioinformatics methodology available at the time of data analysis to investigate the impact of treatment arm and time in the composition of the gut microbiome. Again, a separate analysis plan will be prepared once the main analyses for this trial have been completed.

4 References

1. GBD 2016 Disease and Injury Incidence and Prevalence Collaborators, *Global, regional, and national incidence, prevalence, and years lived with disability for 328 diseases and injuries for 195 countries, 1990-2016: a systematic analysis for the Global Burden of Disease Study 2016.* Lancet, 2017. **390**(10100): p. 1211-1259.
2. Global Burden of Disease Study, *Global Burden of Disease Study 2016 (GBD 2016) Results*. 2016, Institute for Health Metrics and Evaluation (IHME): Seattle, United States.
3. Stevens, G.A., et al., *Global, regional, and national trends in haemoglobin concentration and prevalence of total and severe anaemia in children and pregnant and non-pregnant women for 1995–2011: a systematic analysis of population-representative data.* The Lancet Global Health, 2013. **1**(1): p. e16-e25.
4. WHO, *The global prevalence of anaemia in 2011.* 2015, World Health Organization: Geneva.
5. Wirth, J.P., et al., *Predictors of anemia in women of reproductive age: Biomarkers Reflecting Inflammation and Nutritional Determinants of Anemia (BRINDA) project.* Am J Clin Nutr, 2017. **106**(Suppl 1): p. 416S-427S.
6. Engle-Stone, R., et al., *Predictors of anemia in preschool children: Biomarkers Reflecting Inflammation and Nutritional Determinants of Anemia (BRINDA) project.* Am J Clin Nutr, 2017. **106**(Suppl 1): p. 402S-415S.
7. Kassebaum, N.J., et al., *A systematic analysis of global anemia burden from 1990 to 2010.* Blood, 2014. **123**(5): p. 615-24.
8. Sazawal, S., et al., *Effects of routine prophylactic supplementation with iron and folic acid on admission to hospital and mortality in preschool children in a high malaria transmission setting: community-based, randomised, placebo-controlled trial.* Lancet, 2006. **367**(9505): p. 133-43.
9. Soofi, S., et al., *Effect of provision of daily zinc and iron with several micronutrients on growth and morbidity among young children in Pakistan: a cluster-randomised trial.* Lancet, 2013. **382**(9886): p. 29-40.
10. Prentice, A.M., H. Verhoef, and C. Cerami, *Iron fortification and malaria risk in children.* Jama, 2013. **310**(9): p. 914-5.
11. Prentice, A.M., *Iron metabolism, malaria, and other infections: what is all the fuss about?* J Nutr, 2008. **138**(12): p. 2537-41.
12. Zlotkin, S., et al., *Effect of iron fortification on malaria incidence in infants and young children in ghana: A randomized trial.* Jama, 2013. **310**(9): p. 938-947.
13. Prentice, A.M., et al., *Dietary strategies for improving iron status: balancing safety and efficacy.* Nutr Rev, 2017. **75**(1): p. 49-60.
14. Tolkien, Z., et al., *Ferrous sulfate supplementation causes significant gastrointestinal side-effects in adults: a systematic review and meta-analysis.* PLoS One, 2015. **10**(2): p. e0117383.
15. Mayo-Wilson, E., et al., *Preventive zinc supplementation for children, and the effect of additional iron: a systematic review and meta-analysis.* BMJ Open, 2014. **4**(6): p. e004647.

16. Jaeggi, T., et al., *Iron fortification adversely affects the gut microbiome, increases pathogen abundance and induces intestinal inflammation in Kenyan infants.* Gut, 2014.
17. Zimmermann, M.B., et al., *The effects of iron fortification on the gut microbiota in African children: a randomized controlled trial in Cote d'Ivoire.* Am J Clin Nutr, 2010. **92**(6): p. 1406-15.
18. Tang, M., et al., *Effect of Vitamin E With Therapeutic Iron Supplementation on Iron Repletion and Gut Microbiome in US Iron Deficient Infants and Toddlers.* J Pediatr Gastroenterol Nutr, 2016. **63**(3): p. 379-85.
19. Paganini, D., M.A. Uyoga, and M.B. Zimmermann, *Iron Fortification of Foods for Infants and Children in Low-Income Countries: Effects on the Gut Microbiome, Gut Inflammation, and Diarrhea.* Nutrients, 2016. **8**(8).
20. Naylor, C., et al., *Environmental Enteropathy, Oral Vaccine Failure and Growth Faltering in Infants in Bangladesh.* EBioMedicine, 2015. **2**(11): p. 1759-66.
21. Lin, A., et al., *Household environmental conditions are associated with enteropathy and impaired growth in rural Bangladesh.* Am J Trop Med Hyg, 2013. **89**(1): p. 130-7.
22. Powell, J.J., et al., *A nano-disperse ferritin-core mimetic that efficiently corrects anemia without luminal iron redox activity.* Nanomedicine: Nanotechnology, Biology and Medicine, 2014. **10**(7): p. 1529-1538.
23. Pereira, D.I., et al., *Caco-2 Cell Acquisition of Dietary Iron(III) Invokes a Nanoparticulate Endocytic Pathway.* PLoS One, 2013. **8**(11): p. e81250.
24. Theil, E.C., et al., *Absorption of iron from ferritin is independent of heme iron and ferrous salts in women and rat intestinal segments.* J Nutr, 2012. **142**(3): p. 478-83.
25. Kalgaonkar, S. and B. Lonnerdal, *Receptor-mediated uptake of ferritin-bound iron by human intestinal Caco-2 cells.* J Nutr Biochem, 2009. **20**(4): p. 304-11.
26. San Martin, C.D., et al., *Caco-2 intestinal epithelial cells absorb soybean ferritin by mu2 (AP2)-dependent endocytosis.* J Nutr, 2008. **138**(4): p. 659-66.
27. Pereira, D.I.A., et al., *Dietary iron depletion at weaning imprints low microbiome diversity and this is not recovered with oral nano Fe(III).* MicrobiologyOpen, 2015. **4**(1): p. 12-27.
28. Pereira, D.I.A., et al., *Nanoparticulate iron(III) oxo-hydroxide delivers safe iron that is well absorbed and utilised in humans.* Nanomedicine: Nanotechnology, Biology and Medicine, 2014. **10**(8): p. 1877-1886.
29. Latunde-Dada, G.O., et al., *A Nanoparticulate Ferritin-Core Mimetic Is Well Taken Up by HuTu 80 Duodenal Cells and Its Absorption in Mice Is Regulated by Body Iron.* The Journal of Nutrition, 2014. **144**(12): p. 1896-1902.
30. Aslam, M.F., et al., *Ferroportin mediates the intestinal absorption of iron from a nanoparticulate ferritin core mimetic in mice.* Faseb Journal, 2014. **28**(8): p. 3671-8.
31. Pasricha, S.R., et al., *Expression of the iron hormone hepcidin distinguishes different types of anemia in African children.* Sci Transl Med, 2014. **6**(235): p. 235re3.
32. Pereira, D., et al., *A novel nano-iron supplement to safely combat iron deficiency and anaemia in young children: The IHAT-GUT double-blind,*

randomised, placebo-controlled trial protocol [version 2; referees: 2 approved]. Gates Open Research, 2018. **2**(48).

33. WHO. *Iron Deficiency Anaemia: Assessment, Prevention and Control*. 2001 25/09/2015]; 1-132]. Available from: http://www.who.int/nutrition/publications/en/ida_assessment_prevention_control.pdf.
34. De-Regil, L.M., et al., *Home fortification of foods with multiple micronutrient powders for health and nutrition in children under two years of age.* Cochrane Database Syst Rev, 2011(9): p. CD008959.
35. Ma, Y., et al., *A novel method for non-transferrin-bound iron quantification by chelatable fluorescent beads based on flow cytometry.* Biochem J, 2014. **463**(3): p. 351-62.
