# Supplementary material for: A novel nano-iron supplement versus standard treatment for iron deficiency anaemia in children 6–35 months (IHAT-GUT trial): a double-blind, randomised, placebo-controlled non-inferiority phase II trial in The Gambia
Source: eClinicalMedicine. 2023 Feb 9;56:101853. doi: 10.1016/j.eclinm.2023.101853 (PMC9985047; doi:10.1016/j.eclinm.2023.101853)
Supplement: Supplementary Data S4 [file mmc4.pdf]

Morbidity Questionnaire

1. Randomisation No.	__________________________________
2. Questionnaire administered?	◯ Yes<br>◯ No
3. Administered by	__________________________________
4. Reason for not administering	◯ Caregiver/Mother travelled<br>◯ Caregiver/Mother refused<br>◯ Caregiver/Mother unwell<br>◯ Caregiver/Mother moved away<br>◯ Other
4a. Other reason	__________________________________
5. Any diarrhoea	◯ Yes<br>◯ No
6. If yes, duration of diarrhoea	__________________________________<br>(in days)
7. If yes, number of stools a day	__________________________________
8. If Yes, is there blood in stool?	◯ Yes<br>◯ No
9. Is there mucus in stool?	◯ Yes<br>◯ No
10. Is the child urinating less?	◯ Yes<br>◯ No
11. Is the child lethargic or unconscious?	◯ Yes<br>◯ No
12. Is the child restless and irritable	◯ Yes<br>◯ No

13. Does the child have sunken eyes?

◯ Yes
◯ No

14. Is the child drinking poorly?

◯ Yes
◯ No

15. Is the child thirsty, drinking eagerly

◯ Yes
◯ No

16. Does the skin pinch go back slowly?

◯ Yes
◯ No

MODERATE TO SEVERE DIARRHOEA/ADVERSE EVENT.

17. Auxillary temperature (degrees celsius) | ________________ (Must be to 1 decimal place e.g. 39.5)

18. History of fever | ◯ Yes ◯ No

19. If Yes, number of days | ________________

20. Cough | ◯ Yes ◯ No

21. If yes, duration of cough | ________________ (in days)

22. Difficulty breathing? | ◯ Yes ◯ No

23. Convulsion? | ◯ Yes ◯ No

24. Has the child been vomiting since our last visit? | ◯ Yes ◯ No

25. If Yes, number of days | ________________

26. How is the child's appetite? | ◯ Normal ◯ Decreased

27. If decreased, number of days | ________________

28. If decreased describe | ________________

29. Has the child had any other illness since our last visit? | ◯ Yes ◯ No

ADVERSE EVENT

30. If yes, number of days | ________________

31. If yes, describe | ________________

32. Has the child been or is on any medication since our last visit? ◯ Yes ◯ No

33. If yes, please state the medication ______________________________________

Protocol Deviation Section

34. Protocol deviation ◯ yes ◯ NO
