# Supplementary material for: A novel nano-iron supplement versus standard treatment for iron deficiency anaemia in children 6–35 months (IHAT-GUT trial): a double-blind, randomised, placebo-controlled non-inferiority phase II trial in The Gambia
Source: eClinicalMedicine. 2023 Feb 9;56:101853. doi: 10.1016/j.eclinm.2023.101853 (PMC9985047; doi:10.1016/j.eclinm.2023.101853)
Supplement: Supplementary Data S5 [file mmc5.docx]

A novel nano-iron supplement versus standard treatment for iron deficiency anaemia in children 6-35 months (IHAT-GUT trial): a double-blind, randomised, placebo-controlled non-inferiority trial in The Gambia

Authors:

Nuredin I. Mohammed^1#^, James Wason^2,3#^, Thomas Mendy^1^, Stefan Akio Naß^1,4^, Ogochukwu Ofordile^1^, Famalang Camara^1^, Bakary Baldeh^1^, Chilel Sanyang^1^, Amadou T. Jallow^1^, Ilias Hossain^1^, Nuno Faria^5^, Jonathan J. Powell^5^, Andrew M. Prentice^1^, and Dora I.A. Pereira^1,6^*

**Supplementary Tables**

**Table S1. Number of subjects adhering to daily supplementation doses.** Adherence per study group was defined as the proportion of enrolled subjects in each group who have taken 1-month (28 days), 2 months (49 days) and 3 months (85 days) worth of daily doses. Maximum adherence in each group would be 33.3% if all subjects in that group had not missed a single day of supplementation.

| **Adherence to daily doses over time (days)** | **Study group** | | | **Overall**  **N (%)** |
| --- | --- | --- | --- | --- |
|  | **IHAT**  **n (%)** | **FeSO_4_**  **n (%)** | **Placebo**  **n (%)** |  |
| **28** | 195(30.4) | 201(31.3) | 198(30.8) | 594(92.5) |
| **49** | 190(29.6) | 200(31.2) | 198(30.8) | 588(91.6) |
| **85** | 189(29.4) | 196(30.5) | 195(30.4) | 580(90.3) |

**Table S2. Number of participants with missing follow-up data on Day 85 and Complete Case (CC) numbers for the primary outcomes in the ITT and PP analysis populations.**

|  | **ITT population** | | | **PP population** | | | |
| --- | --- | --- | --- | --- | --- | --- | --- |
|  | **Total** | **Case complete** | **Missing** | **Total** | **Case complete** | **Missing** |  |
| **Total** | 642 | 563 IDA assessment  584 Diarrhoea assessment | 79  58 | 582 | 553 IDA assessment  582 Diarrhoea assessment | 29  nil |  |
| **IHAT** | 214 | 180 IDA assessment  190 Diarrhoea assessment | 34  24 | 189 | 177 IDA assessment  189 Diarrhoea assessment | 12  nil |  |
| **FeSO_4_** | 214 | 192 IDA assessment  196 Diarrhoea assessment | 22  18 | 198 | 190 IDA assessment  198 Diarrhoea assessment | 8  nil |  |
| **Placebo** | 214 | 191 IDA assessment  198 Diarrhoea assessment | 23  16 | 195 | 186 IDA assessment  195 Diarrhoea assessment | 9  nil |  |

ITT, intention-to-treat; PP, per-protocol

**Table S3 – Summary of primary outcomes.** PP – per-protocol, ITT – intention-to-treat; MI - multiple imputation (using chained equations); NI – non-inferiority; NR – not reported as not pre-specified analysis; OR – odds-ratio; RR – rate ratio

|  |  |  | Group | | |
| --- | --- | --- | --- | --- | --- |
| Outcome | Population | Parameter | IHAT | FeSO_4_ | Placebo |
| IDA correction/response probability | | |  |  |  |
|  | MI_ITT | OR (80% CI)- Crude | 1.46 (1.08, 1.98) | Reference | NR |
|  | N=642 | OR (80% CI)- Adjusted* | 1.49 (1.09, 2.02) | Reference | NR |
| Incidence density of moderate-severe diarrhoea | | |  |  |  |
|  | PP | Episodes/child | 2.74 | 3.50 | 2.96 |
|  | N=582 | RR (80% CI)- Crude | 0.78 (0.61, 1.01) | Reference | NR |
|  |  | RR (80% CI)- Adjusted* | 0.77 (0.6, 1.0) | Reference | NR |
|  |  | P-value for superiority | 0.0981 |  |  |
|  |  |  |  |  |  |
|  | MI_ITT | RR (80% CI)- Crude | 0.81 (0.63, 1.04) | Reference | NR |
|  | N=642 | RR (80% CI)- Adjusted* | 0.8 (0.63, 1.03) | Reference | NR |
|  |  | P-value for superiority | 0.133 |  |  |
| Prevalence of moderate-severe diarrhoea | | |  |  |  |
|  | MI_ITT | OR (80% CI)- Crude | Reference | 1.06 (0.79, 1.44) | 0.94 (0.69, 1.27) |
|  | N=642 | OR (80% CI)- Adjusted* | Reference | 1.07 (0.79, 1.45) | 0.94 (0.69, 1.28) |
|  |  | P-value for superiority |  | 0.3912 | NR |

*Adjusted for age and Hb groups; P-values for superiority are one-sided for the primary endpoints. NI p-values are not reported at the request of the reviewer, interpretation is in the context of the 80% CI.

**Table S4. Iron status and inflammation biomarkers at study Day 1 (baseline) and Day 85.**

| Mean (SD) | Day 1 | | | Day 85 | | |
| --- | --- | --- | --- | --- | --- | --- |
|  | IHAT | FeSO_4_ | Placebo | IHAT | FeSO_4_ | Placebo |
| AGP (g/l) | 1.07 (0.41) | 1.1 (0.41) | 1.1 (0.4) | 1.19 (0.42) | 1.15 (0.42) | 1.12 (0.39) |
| Calprotectin* (μg/g) | 231.41 (71.2-445.78) | 180.41 (80.66-397.72) | 212.77 (87.12-535.87) | 211.98 (102.88-600) | 207.16 (113.25-600) | 198.73 (67.46-447.58) |
| CRP (mg/l) | 3.24 (8.5) | 3.94 (11.12) | 3.9 (10.16) | 4.93 (8.63) | 4.13 (8.44) | 4.02 (9.26) |
| Ferritin_adjusted_ (μg/l) | 5.46 (4.93) | 5.01 (4.95) | 5.08 (5.29) | 13.01 (12) | 11.76 (9.06) | 5.67 (5.02) |
| Ferritin_unadjusted_ (μg/l) | 12.95 (11.49) | 13 (16.13) | 14.19 (23.11) | 35.74 (30.12) | 32.01 (32.67) | 14.45 (14.19) |
| Hb (g/dl) | 9.14 (0.98) | 9.22 (1.02) | 9.16 (0.95) | 10.42 (0.91) | 10.33 (0.94) | 9.26 (0.99) |
| MCH (pg) | 20.73 (2.63) | 20.7 (2.51) | 20.94 (2.64) | 22.93 (2.2) | 22.81 (2.15) | 20.98 (2.67) |
| MCV (fl) | 61.33 (6.52) | 61.16 (6.3) | 61.82 (6.9) | 67.11 (5.61) | 66.83 (5.34) | 62.38 (6.92) |
| sTfR (mg/l) | 8.73 (3.71) | 8.65 (3.32) | 8.62 (3.51) | 6.03 (1.9) | 6.3 (2) | 8.7 (3.15) |
| sTfR/log_10_Ferritin index* | 7.73 (5.2-12.16) | 8.92 (5.3-13.46) | 7.87 (5.52-13.78) | 3.85 (3-5.34) | 4.39 (3.32-5.97) | 7.56 (4.99-12.68) |

| *Median (IQR) |
| --- |

**Table S5. Summary of all serious adverse events**

| **Subject ID** | **Study group** | **Age**  **(months)** | **Onset Date** | **Stop Date** | **Study Day** | **Relationship to Intervention*** | **Discontinued study treatment (Y/N)** | **Outcome**** | **Description of SAE** |
| --- | --- | --- | --- | --- | --- | --- | --- | --- | --- |
| **186B** | Placebo | 14 | 26/04/2018 | 29/04/2018 | 109 | Unlikely | N | Recovered, with treatment | Hospitalisation for treatment of acute respiratory infection (ARI) (history of bronchiolitis) |
| **244B** | IHAT | 9 | 18/10/2018 | 22/10/2018 | 65 | Possible | N | Recovered, with treatment | Hospitalisation for treatment of acute respiratory infection (ARI) |
| **268H** | Placebo | 14 | 30/08/2018 | 01/09/2018 | 16 | Possible | Y  Day 12 | Recovered, with treatment | Hospitalisation for treatment of acute respiratory infection (ARI) |
| **618H** | FeSO_4_ | 14 | 20/07/2018 | 20/07/2018 | 87 | Definitely unrelated | N | Subject died | Upper airway obstruction secondary to peanut powder inhalation, no anaphylaxis |
| **643K** | FeSO_4_ | 23 | 31/03/2018 | 23/04/2018 | 81 | Possible | Y  Day 80 | Recovered, with treatment | Hospitalisation for treatment of haemolytic uremic syndrome |

*** Definitely related, Probable, Possible, Unlikely, Definitely unrelated**

**** Recovered, without treatment; Recovered, with treatment; Still Present, no treatment; Still Present, being treated; Residual effect(s) present – no treatment; Residual effect(s) present- being treated; Subject died**

**Table S6. Summary of all adverse events for subjects by treatment arm in all randomised children.** The pre-specified safety population in this study included all children that were randomised; only 3 children randomised to the IHAT group and 2 children randomised to the FeSO_4_ group did not receive at least one dose of their allocated treatment; none of these 5 children had adverse events reported during the study.

| **Adverse Events** | **All randomised** | | | **Overall**  **(N=642)** |
| --- | --- | --- | --- | --- |
|  | IHAT  (n=214) | FeSO_4_  (n=214) | Placebo  (n=214) |  |
| **Number of AEs reported** | 225 | 251 | 246 | 722 |
| **Number of subjects with AEs, N(%) [1]** | 143(66.8) | 146(68.2) | 143(66.8) | 432(67.3) |
| **Duration of AEs in days (Median, Min-Max)** | 5(1-12) | 5(1-24) | 5(1-16) | 5(1-24) |
| **Maximum AEs number per subject** | 5 | 6 | 5 | 6 |
| **Number of SAEs reported** | 1 | 2 | 2 | 5 |
| **SAEs leading to treatment discontinuation** | 0 | 1 | 1 | 2 |
| **Number of subjects with SAEs, N(%) [1]** | 1(0.5) | 2(0.9) | 2(0.9) | 5(0.8) |
| **Deaths, N(%)** | 0 | 1(0.5) | 0 | 1 |
| **Number of AEs by severity*, N (%)** |  |  |  |  |
| Mild | 151(67.1) | 163(64.9) | 155(63.0) | 469(65.0) |
| Moderate | 66(29.3) | 79(31.5) | 83(33.7) | 228(31.6) |
| Severe | 8(3.6) | 9(3.6) | 8(3.3) | 25(3.5) |
| Life-threatening | 0 | 0 | 0 | 0 |
| **Subjects with AEs by severity [2]**** |  |  |  |  |
| Mild | 84(58.7) | 79(54.1) | 78(54.5) | 241(55.8) |
| Moderate | 52(36.4) | 59(40.4) | 57(39.9) | 168(38.9) |
| Severe | 7(4.9) | 8(5.5) | 8(5.6) | 23(5.3) |
| Life-threatening | 0 | 0 | 0 | 0 |
| **CAUSALITY** | | | | |
| **Number of AEs by possible relatedness to treatment*, N (%)** |  |  |  |  |
| Definitely unrelated | 11(4.9) | 22(8.8) | 15(6.1) | 48(6.6) |
| Unlikely | 61(27.1) | 78(31.1) | 60(24.4) | 199(27.6) |
| Possible | 153(68.0) | 151(60.2) | 171(69.5) | 475(65.8) |
| Probable | 0 | 0 | 0 | 0 |
| Definitely related | 0 | 0 | 0 | 0 |
| **Subjects with AEs by possible relatedness to treatment [2]**** |  |  |  |  |
| Definitely unrelated | 7(4.9) | 9(6.2) | 6(4.2) | 22(5.1) |
| Unlikely | 27(18.9) | 32(21.9) | 23(16.1) | 82(19) |
| Possible | 109(76.2) | 105(71.9) | 114(79.7) | 328(75.9) |
| Probable | 0 | 0 | 0 | 0 |
| Definitely related | 0 | 0 | 0 | 0 |

[1] Subjects who experience one or more AEs or SAEs are counted only once.

[2] Subjects are counted only once within a particular severity grade or relatedness category.

*Percentages are based on total number of AEs reported in each group.

**Percentages are based on total number of subjects with reported AEs in each group.
