# Supplementary material for: A novel nano-iron supplement versus standard treatment for iron deficiency anaemia in children 6–35 months (IHAT-GUT trial): a double-blind, randomised, placebo-controlled non-inferiority phase II trial in The Gambia
Source: eClinicalMedicine. 2023 Feb 9;56:101853. doi: 10.1016/j.eclinm.2023.101853 (PMC9985047; doi:10.1016/j.eclinm.2023.101853)
Supplement: Supplementary Data S6 [file mmc6.docx]

A novel nano-iron supplement versus standard treatment for iron deficiency anaemia in children 6-35 months (IHAT-GUT trial): A double-blind, randomised, placebo-controlled non-inferiority trial in The Gambia

Authors:

Nuredin I. Mohammed^1#^, James Wason^2,3#^, Thomas Mendy^1^, Stefan Akio Naß^1,4^, Ogochukwu Ofordile^1^, Famalang Camara^1^, Bakary Baldeh^1^, Chilel Sanyang^1^, Amadou T. Jallow^1^, Ilias Hossain^1^, Nuno Faria^5^, Jonathan J. Powell^5^, Andrew M. Prentice^1^, and Dora I.A. Pereira^1,6^*


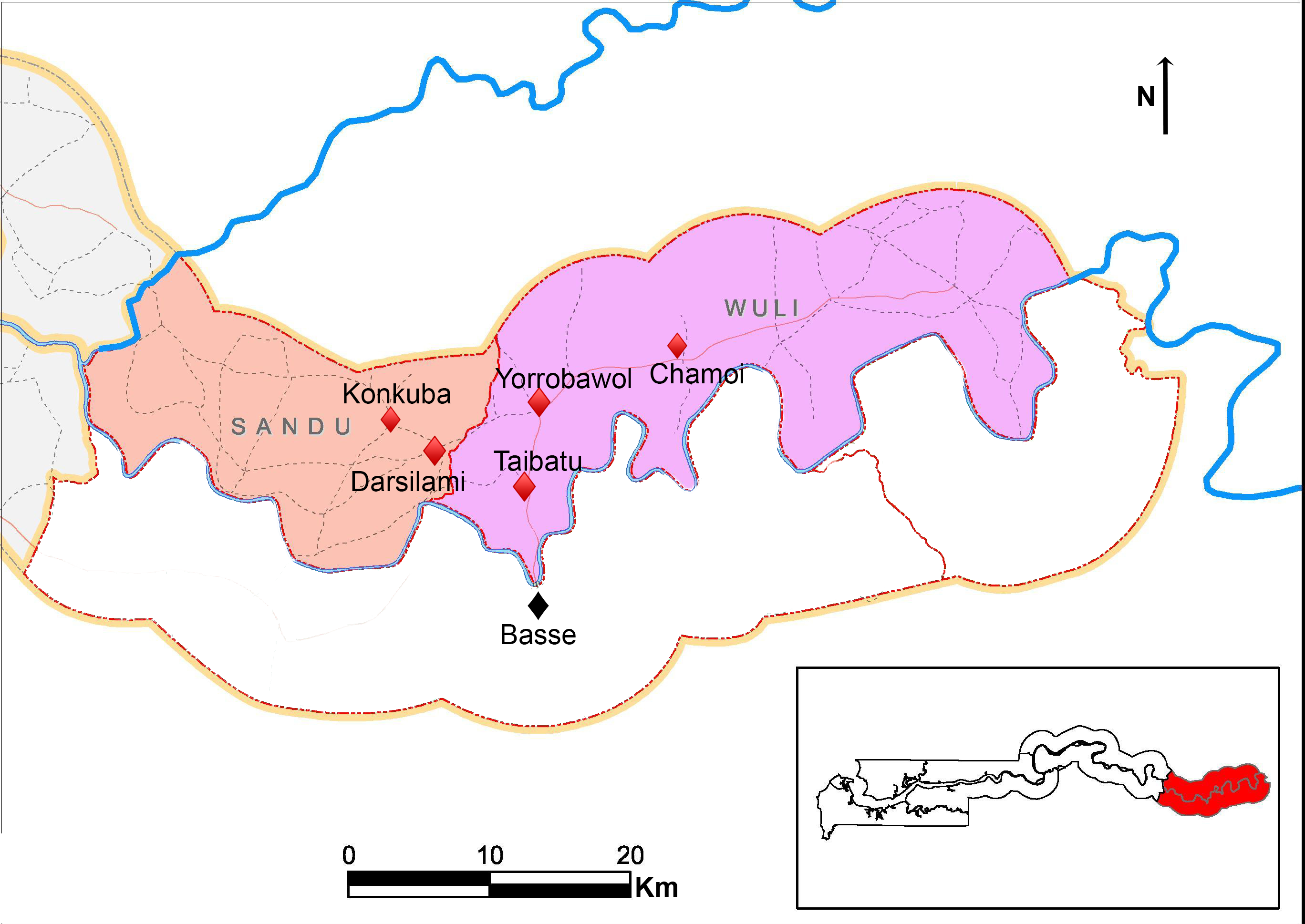


**Supplementary Figure 1.** Map of Upper River Region in The Gambia, with location of study clinical facilities. Study samples are collected at one of the clinical facilities, Yorrobawol health center, Darsilami community health post, Konkuba community health post, Taibatu health post and Chamoi Health Center, and transported to the study laboratory in Basse for sample processing and analysis, and from there to other laboratories for further analysis. Image reproduced from © 2018 Pereira DIA, Mohammed NI, Ofordile O *et al.* A novel nano-iron supplement to safely combat iron deficiency and anaemia in young children: The IHAT-GUT double-blind, randomised, placebo-controlled trial protocol [version 2; peer review: 2 approved]. *Gates Open Res* 2018, 2:48 (<https://doi.org/10.12688/gatesopenres.12866.2>)
