# Supplementary material for: A novel nano-iron supplement versus standard treatment for iron deficiency anaemia in children 6–35 months (IHAT-GUT trial): a double-blind, randomised, placebo-controlled non-inferiority phase II trial in The Gambia
Source: eClinicalMedicine. 2023 Feb 9;56:101853. doi: 10.1016/j.eclinm.2023.101853 (PMC9985047; doi:10.1016/j.eclinm.2023.101853)
Supplement: Supplementary Data S7 [file mmc7.pdf]

INDEPENDENT DATA AND SAFETY MONITORING BOARD (DSMB) CHARTER

A novel nano-iron supplement (IHAT) to safely combat iron deficiency and anaemia (IDA) in young children: a double-blind randomised controlled trial

IHAT-Gut trial

Protocol No: SCC1489

Sponsor: Medical Research Council Unit The Gambia at the London School of Hygiene and Tropical Medicine (MRC Unit The Gambia at LSHTM)

Clinicaltrials.org: NCT02941081

Chief Investigator: Andrew Prentice (MRC Unit The Gambia at LSHTM)

Principal Investigators: Dora Pereira (PI, University of Cambridge) and Mohammad Ilias Hossain (local PI and Clinical Trial Coordinator, MRC Unit The Gambia at LSHTM)

DSMB Charter Version – Date V2.0, 09th April 2018

Table of contents

Signature page 2
1 Introduction 5
1.1 Trial name and identification 5
1.1.1 Full title 5
1.1.2 Acronym 5
1.1.3 Sponsors identification 5
1.1.4 Trial registration 5
1.2 Trial objectives 5
1.2.1 Interventions 5
1.2.2 Primary 5
1.2.3 Secondary 6
1.2.4 Exploratory 6
1.3 Overview of the trial design 6
1.4 Scope of the charter 9
2 Roles and Responsibilities 9
2.1 Overview 9
2.2 Terms of reference 9
2.3 Specific roles of the DSMB 9
2.4 Trial pause rules and DSMB review 10
2.5 Deaths of trial participants 11
3 Before or early in the trial 12
4 Composition 12
4.1 Membership 12
4.2 Definition of quorate 13
4.3 Independence of the DSMB members 14
4.4 Roles 14
4.4.1 Chair (or Deputy Chair) 14
4.4.2 DSMB statistician 14
4.4.3 Trial statistician 15
4.4.4 Principal investigators and other trial staff 15
4.4.5 Sponsor representative 15
5 Relationships 15
6 Organisation of the DSMB meetings 16
6.1 First meeting 16
6.2 Follow-up meetings 16
6.3 Final meeting 17
6.4 Additional meetings 17
7 Trial documentation and procedures to ensure confidentiality and proper communication 17
7.1 Provision of documents 17
7.2 Scientific update 17
7.3 Open sessions 17

7.4 Closed sessions 18
7.5 Communication 19
8 Decision making 19
9 Reporting 20
10 After the trial 21
11 AppendiX – DSMB SiGnature page 22

1 Introduction

1.1 Trial name and identification

1.1.1 Full title

A novel nano-iron supplement (IHAT) to safely combat iron deficiency and anaemia (IDA) in young children: a double-blind randomised controlled trial.

1.1.2 Acronym

IHAT-Gut

1.1.3 Sponsors identification

SCC1489

1.1.4 Trial registration

NCT02941081

1.2 Trial objectives

1.2.1 Interventions

- Daily supplementation with the clinical standard for iron supplementation (ferrous sulphate, $FeSO_4$ powder) equivalent to 12.5 mg Fe

- Daily supplementation with iron hydroxide adipate tartrate (IHAT, a new iron formulation developed by the MRC) powder bioequivalent to 12.5 mg Fe (i.e. 20 mg Fe taking into account IHAT's relative bioavailability to $FeSO_4$)

- Daily dose of a placebo powder (pure saccharose EP/USP-NF)

1.2.2 Primary

- Show non-inferiority of IHAT compared to ferrous sulphate for efficacy (in terms of Hb and iron deficiency correction: i.e. IDA) response probability (or prevalence).

- Show superiority of IHAT compared to ferrous sulphate in terms of incidence density of moderate-severe diarrhoea.

- Show superiority of IHAT compared to ferrous sulphate in terms of prevalence of moderate-severe diarrhoea.

- Show non-inferiority of IHAT compared to placebo in terms of prevalence of moderate-severe diarrhoea.

1.2.3 Secondary

- Show that IHAT supplementation does not increase enteric pathogen burden.

- Show that IHAT supplementation is non-detrimental to the gut microbiome (for example in terms of enterobacteria abundance and the ratio bifidobacteria:enterobacteria)

- Show that IHAT supplementation does not cause additional intestinal inflammation.

- To show that IHAT supplementation does not cause more treatment failures than ferrous sulphate supplementation.

- To describe the effect of IHAT supplementation on malaria infection risk.

- To describe the impact of IHAT supplementation on hospitalisation and morbidity, particulary in relation to malaria and other infectious diseases.

- To show that IHAT supplementation does not increase the longitudinal prevalence of moderate-severe diarrhoea.

- To show that IHAT supplementation does not increase the incidence density of bloody diarrhoea.

- To determine the effect of IHAT supplementation on systemic inflammation.

- To determine the effect of IHAT supplementation on systemic markers of iron handling (for example non-transferrin bound iron).

1.2.4 Exploratory

- To investigate the effect of iron supplementation on soil-transmitted helminths (STH) infection.

- To investigate the association of specific bacteria groups of the gut microbiome (e.g. enterobacteria) with STH.

1.3 Overview of the trial design

For detailed information about the trial design and conduct please refer to the IHAT-Gut clinical trial protocol.

To investigate the primary and secondary objectives we will conduct a 3-arm, parallel, randomised, double-blind, placebo-controlled, phase 2, clinical trial.

Participants will be iron deficient anaemic young children living in rural communities in the North Bank of the Upper River Division in The Gambia. Inclusion criteria will be: apparently healthy as judged by a study nurse on the day of recruitment, age 6-35 months, no malaria (RDT negative), not severely

malnourished (HAZ, WAZ, WHZ >-3 SD), and IDA defined as 7≤Hb<11 g/dl (for recruitment we will use Medonic Hb values rather than HemoCue) with serum ferritin<30 μg/L.

The communities and health centres within the study catchment area (Wuli and Sandu districts) will be sensitised to the study. Young children will be identified using the enumeration/census data collected by the field team within the study catchment area. At screening, once mothers/guardians of the child have signed the informed consent form, the child will be physically examined by a study nurse and, if the child is considered as generally healthy, their height and weight will be measured and a finger prick blood sample will be collected for Hb and RDT testing. If z-scores are >-3, 7 ≤Hb< 11 g/dL and the RDT is negative, a small venous blood sample will be collected to confirm the Hb levels and determine serum ferritin. A total of 705 eligible children will be randomised into the 3 study arms (n=235 per arm).

In each study arm, the children will be supplemented daily for 12 weeks with either placebo, ferrous sulphate or IHAT. Blood and stool samples will be collected at baseline (Day 1) and at day 15 and day 85 during the intervention period. Following the 12 weeks of intervention there will be an additional active follow-up period of 4 weeks without intervention.

Highly trained and experienced field workers will be visiting all children every day during the 12 weeks supplementation period in order to administer the iron supplements or placebo and on these occasions they will check on the children's general health and actively look for signs of malaria and co-infections. If a child shows signs of these infections, the field worker will refer to the study nurse who will perform adequate tests and the child will be offered the appropriate treatment/referral to the next health center. Three times per week, morbidity data (including questions regarding fever, diarrhoea, vomiting, cough, any other illness, appetite and any mediation taken and assessment of body temperature) will be captured. Every week children will visit one of the study health clinics and be screened using a finger prick blood sample to determine their malaria and Hb status and children found with a positive RDT during the study will be further tested with a blood film and treated according to national guidelines. These visits will continue 4 weeks post intervention to follow-up on AE/SAEs. Any child where Hb falls below 7 g/dL during the follow-up study period will stop the study and will be referred to the next health centre for management and will be provided with iron supplements for 3 consecutive months according to national and WHO guidelines. These children will be treated in the data analysis as treatment failures.

At the end of the follow-up period (12+4 weeks), the children in any arm who still have anaemia (Hb<11 g/dL) will be provided with iron supplementation for 3 consecutive months as per national and WHO guidelines.

The flow chart of the study showing the timings of the interventions and laboratory assessments is shown in Figure 1.

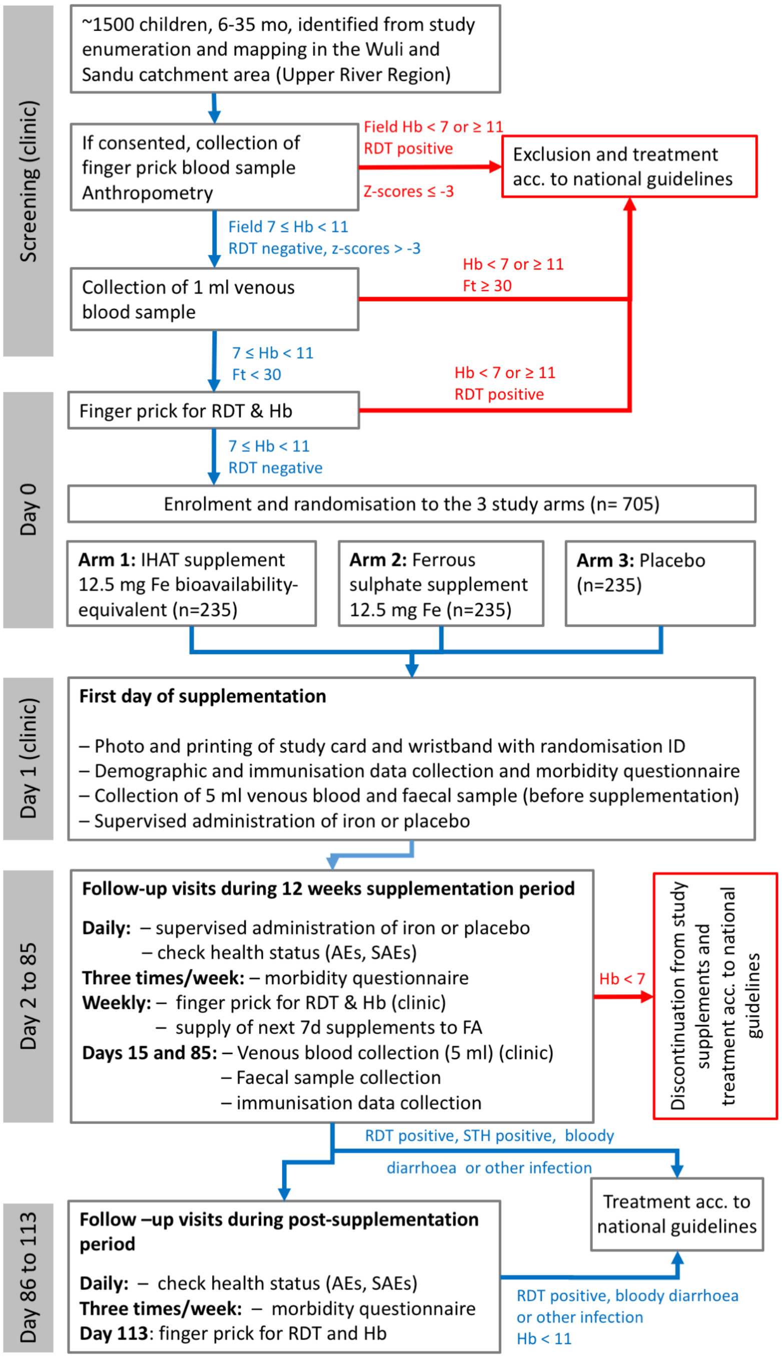

Figure 1. Study Flow Chart.

Abbreviations: Hb, haemoglobin; Ft, ferritin; RDT, rapid diagnostics test; STH, soil-transmitted helminths; AE, adverse events; SAEs, serious adverse events; FA, field assistant; Fe, iron.

1.4 Scope of the charter

The purpose of the DSMB charter is to describe the roles and responsibilities of the independent DSMB for the IHAT-Gut trial, including the membership of the DSMB, the timing and organisation of the meetings, methods of providing information to and from the DSMB, frequency and format of the meetings, statistical issues and relationships with the sponsor, the principal investigators and the trial steering committee (TSC).

2 Roles and Responsibilities

2.1 Overview

The role of the DSMB is to help safeguard the health and interests of children enrolled in the IHAT-Gut trial, through periodic review of the safety data generated, and to monitor the overall conduct of the trial in order to protect the validity and credibility of the trial. The DSMB will not review the efficacy or other non-safety endpoints during the trial.

2.2 Terms of reference

The DSMB should receive and review the safety and the recruitment and retention data generated during the trial, along with a summary of monitoring findings, and advise the sponsor and the trial steering committee (TSC) if, in their view:

- There is a level of concern about the safety of the young children in any treatment arm such that future recruitment to that arm is no longer judged to be appropriate.

- The quality of the safety data generated is of such concern (in terms of completeness and accuracy) that an assessment of the children's safety is not considered to be possible.

- The quality of the safety and other data generated is of such significant concern (in terms of completeness or accuracy) that the results of the trial are likely to be called into question irrespective of outcome.

- The rate of recruitment is such that it is considered to be unlikely that the trial will be completed successfully.

- They become aware of other data addressing the same or related questions to those in the IHAT-Gut trial such that the trial is no longer considered to be justified in its current form.

2.3 Specific roles of the DSMB

- To evaluate, on an ongoing basis, the accumulating solicited and unsolicited safety data from the children enrolled in the trial and to advise on trial continuation on this basis. Safety data will be reviewed by the DSMB as overall pulled data (open session) and tabulated by coded treatment arm (closed

session). Unblinding of the treatment arm codes will only be requested by the DSMB if there are statistical differences ($p<0.05$) between treatment arms that warrant important concerns in terms of the overall safety of trial participants or in individual cases if there is a pattern of SAEs which may be related to study treatment where the DSMB feels there is ‘potential for harm’.

- To monitor the rate of recruitment and level of retention of children and to examine any trends apparent related to non-retention (e.g. consent withdrawal, loss to follow-up etc) and to provide advice accordingly.

- To monitor evidence for treatment harm (e.g. AEs, SAEs, deaths)

- To assess data quality and completeness with the aim of ensuring the subsequent validity and credibility of the data generated.

- To monitor protocol compliance by investigators and participants.

- To advise on protocol amendments.

- To consider factors external to the trial when relevant information becomes available, such as scientific or therapeutic developments that may have an impact on the safety of the participants or the ethics of the trial.

- To ensure at all times the confidentiality of the trial data and the DSMB discussions.

- To consider the ethical implications of any recommendations made.

- To report conflicts of interest guidelines as detailed in this charter (see DSMB Membership).

- To document the outcome of all DSMB reviews and to provide the necessary information to the sponsor via the trial steering committee (TSC).

- To decide whether to recommend that the trial continues to recruit participants or whether recruitment should be stopped either for everyone or for some treatment groups and/or some participant subgroups.

- To suggest additional data analyses.

- The DSMB will not evaluate the efficacy or other non-safety endpoints during the trial although they may be asked to comment on these outcomes following trial completion. The majority of data related to these endpoints will only become available at this point.

2.4 Trial pause rules and DSMB review

Recruitment to the trial will be paused with immediate effect if any child has a serious adverse reaction (SAR), whether this is a suspected serious adverse reaction (SSAR) or suspected unexpected serious adverse reaction (SUSAR), to any of the iron supplements (or placebo) administered during the trial.

SAR is defined in the study protocol and constitutes any SAE where a causal relationship between the study supplements (or placebo) and the SAE is at least a reasonable probability, i.e. 'likely to be caused by' (probably related or definitely related). Follow-up of the children who have already been recruited will continue as planned pending the review of the DSMB.

Causality will be determined initially by the principal investigators in discussion with the study clinician, the clinical team, and with input from the local safety monitor, considering the chronology of events and evidence for other causes – either from medical history, examination or further investigations. Such assessment, particular in relation to 'expectedness', will take into consideration the safety profile of iron supplements and the information included in the 'Reference Safety Information' section of the IHAT Investigator Brochure; a conservative approach will be taken when there is doubt.

All SAR will be reported to the Sponsor and the local safety monitor (LSM) by email using the standard SAE report for the trial within 24 hours of the investigators becoming aware.

The complete report of an SAR will be send to the DSMB members as soon as the detailed report of the event is available (usually within 7 days of the investigator becoming aware). The DSMB chair or his deputy will acknowledge receipt of the report and any member of the DSMB may seek further information if judged to be required.

The DSMB will be asked to review all available data related to the SAR and advice on trial continuation. The DSMB will endeavour to respond within 7 days, initially by email to the TSC. Additional meetings may be necessary to make a full assessment of the reaction and its outcome, and these may be chaired by another member of the DSMB if necessary.

2.5 Deaths of trial participants

Any death considered to represent an SAR will prompt a trial pause as outlined above (2.4).

In addition, all deaths not considered to be related to the study interventions will also be reported by email to the Sponsor and the LSM within 24 hours. Reporting to the DSMB members will be made as soon as a full report related with the event is available. All other SAE which do not represent death, i.e. those medical events that are life threatening, require hospitalisation, or result in persistent or significant disability, will be reported by email to the Sponsor and the LSM within 7 days of the investigators becoming aware. Reporting to the DSMB members will be made as soon as a full report related with the event is available.

The DSMB chair or his deputy will acknowledge receipt of the report and any member of the DSMB may seek further information if required. A DSMB meeting could be setup under these circumstances if there are any concerns for the absence of an apparent causal association. All deaths and other SAE will be reviewed at the scheduled DSMB meetings.

3 Before or early in the trial

All members of the DSMB should review the protocol and should be supportive of the trial aims and methods while being in a position to maintain the independence and the critical and constructive review required of DSMB members. Any individual invited to take part of the DSMB who does not consider themselves in this position should decline the role – where possible with due explanation.

The DSMB members are not expected to have direct input into the clinical trial protocol unless, on review, any aspect of the protocol is considered to be contrary to the DSMB aims such that a protocol amendment is deemed to be required.

All DSMB members should review the DSMB Charter and may provide advice or recommend modifications to the charter prior to or at the initial DSMB meeting.

The DSMB should be provided with a 'dummy' report, exemplifying the format of the data to be provided, prior to the first meeting where 'real' trial data will be reviewed. This will help the DSMB members to familiarise themselves with the structure of the report but will also provide them the opportunity to request modifications to the report for ease of review.

The DSMB should hold their first meeting before the trial starts or during the initial month of recruitment for the trial, to discuss the protocol, the trial, future meetings, and to have an opportunity to clarify any aspects with the principal investigators.

4 Composition

Prior to the first DSMB meeting, all DSMB members will be required to sign a statement confirming their independence, declaring any real or potential conflict of interest and confirming that they are prepared to maintain the confidentiality of the trial data throughout the trial.

4.1 Membership

The details of the members of the DSMB for the IHAT-Gut trial are provided below.

Name and Position	Role	Voting member	Fields of expertise
**Professor James Jay Berkley**<br><br>**Professor of Paediatric Infectious Diseases, KEMRI-Wellcome (Kilifi, Kenya)**	Chair	Yes	Consultant paediatrician, paediatric infectious diseases, infection and inflammation in childhood malnutrition, perinatal health.

**Dr Sant-Rayn Pasricha**<br><br>**Researcher in iron biology, MRC Human Immunology Unit, University of Oxford (U.K.)**	Member	Yes	MD (haematology), iron nutrition and iron metabolism, public health nutrition, haemoglobinopathies, anaemia
**Dr Stefan Unger**<br><br>**Paediatric Respiratory Consultant and Honorary Clinical Fellow, University of Edinburg (U.K.)**	Deputy Chair	Yes	Consultant paediatrician, infectious diseases, childhood malnutrition, trial management in LMIC
**Dr Ronald Kiguba**<br><br>**Lecturer, Department of Pharmacology & Therapeutics, College of Health Sciences, Makerere University (Uganda)**	Member	Yes	Clinical epidemiology & biostatistics, Pharmacoepidemiology
**Dr Munya Dimairo**<br><br>**Medical statistician, Clinical Trials Research Unit, University of Sheffield (U.K.)**	Member	Yes	DSMB biostatistician

The Curriculum Vitae of the DSMB members are included in Appendix.

4.2 Definition of quorate

Every effort should be made to set the date of the DSMB meetings to allow all members to attend.

At least three members of the DSMB including both the Chair and the DSMB statistician must be present for the DSMB to be considered quorate. If the Chair cannot attend the meeting, he will delegate Chair responsibilities to the Deputy Chair, but these situations should be an exception. Additional reports should be actively pursued if any DSMB member is unable to attend.

The chair should confirm whether a particular individual wishes to remain as part of the DSMB if they are not able to attend two meetings in a row.

4.3 Independence of the DSMB members

It is essential that the judgment of members of the DSMB is not influenced by factors other than those necessary to maintain the IHAT-Gut trial participant safety, and to preserve the integrity of the study. Independence is essential to ensure that DSMB members are objective and capable of an unbiased assessment of the trial safety data and data quality. The following will ensure the independence of the DSMB:

- Members of the DSMB will not participate in any aspect of the investigations to be undertaken within this trial. They will not have any direct involvement in organising or running the trial or in the final publication of trial results.

- Members of the DSMB must not have a direct interest in knowing or influencing the trial outcome or have a financial or intellectual interest in a specific trial outcome. Such an interest might include a strongly held prior belief in the value or otherwise of the study interventions.

- DSMB members must disclose all pharmaceutical companies, biotechnology companies, and CROs in which they hold financial interest. Members must disclose all consultancies with pharmaceutical companies, biotechnology companies, and CROs which could be considered as relevant to the trial.

- By agreeing to be a DSMB member, the member is stating that there are no conflicts of interest preventing their independent and unbiased review of the IHAT-Gut trial.

4.4 Roles

4.4.1 Chair (or Deputy Chair)

The chair of the DSMB will chair the DSMB meetings (open and closed sessions) with the aim of ensuring that the views of all members of the DSMB are heard and that, when possible, a consensus decision is reached on any question. The chair will aim to avoid being overly directive during the DSMB meetings, rather should facilitate the free expression of views by all members.

4.4.2 DSMB statistician

The DSMB statistician provides advice to the DSMB regarding any statistical analysis which has been undertaken on the data to be examined and may, if necessary, request that additional analysis be performed if judged to be necessary as part of the DSMB decision making process. When possible such requests should be made in advance of the DSMB meeting itself.

The DSMB statistician will undertake the analysis of the safety data tabulated by treatment arm for the report to present during the closed session of the DSMB meetings.

The DSMB statistician will have the responsibility to take minutes of the closed sessions of the meetings and write the closed session reports.

4.4.3 Trial statistician

The trial statistician (Dr Nuredin Mohammed) will provide the necessary summary statistics and will undertake any additional statistical analysis required in generating the necessary reports for the open sessions of the DSMB meetings, but will remain blinded to the treatment arms. The trial statistician will provide the DSMB statistician with the necessary information to conduct the tabulation by treatment arm for the closed session of the meetings but will remain blinded to the treatment arms and will not conduct any data analysis based on grouping of study participants per treatment arm. He may also be asked to provide additional statistical analysis in discussion with the DSMB statistician.

The trial statistician does not play a role in the decision making process of the DSMB.

4.4.4 Principal investigators and other trial staff

The principal investigator, through the TMG, will provide the necessary details regarding specific safety events (e.g. serious adverse events) in the report to the DSMB while remaining blinded to study group. The principal investigator or their representative will be present during the open session of the DSMB meetings to provide any further details of safety events or the processes for safety reporting as requested by the DSMB. The principal investigator will take the minutes of the open session of the DSMB meetings.

The principal investigator do not play any role in the decision making process of the DSMB.

4.4.5 Sponsor representative

A representative of the sponsor may be invited to the open sessions of the DSMB meetings and may be asked to answer any questions regarding the ongoing oversight of the trial but will not play any role in the decision making process of the DSMB.

5 Relationships

The DSMB makes recommendations to the sponsor, through the Trial Steering Committee (TSC), regarding the outcome of the DSMB meetings and whether continued recruitment is warranted. The DSMB also makes recommendations for any additional safety or other follow-up measures. The sponsor will delegate decisions regarding the trial proceeding to the TSC. However, only under exceptional circumstances, and following discussions between the DSMB chair, the TSC and the sponsor, would the TSC be expected to act contrary to the recommendation of the DSMB. The Trial Management Group (TMG) will act upon the decisions of the TSC.

6 Organisation of the DSMB meetings

For practical reasons the DSMB meetings will routinely take place by teleconference or videoconference rather than face-to-face.

Each DSMB meeting will include an open session and a closed session.

At the open sessions, the principal investigator(s) and other senior members of the site investigator team, and members of the DSMB will be present along with the trial statistician. A sponsors representative may also be present and may be requested by the DSMB members. During the open session, summary statistics for all children enrolled in the trial (all groups combined) will be presented. This will allow any specific queries related to trial conduct, reporting, or to specific safety events, to be raised and addressed by the investigator team who will be best placed to do so. Information on recruitment rates, data quality and adherence to protocol will also be presented. These data will be presented by the principal investigator(s) or a representative alongside the trial statistician, as appropriate.

The closed session will involve only the DSMB voting members. During the closed session, the same summary statistics divided according to treatment arm, as prepared by the DSMB statistician, will be reviewed by the DSMB.

In general, the DSMB reports/data presentation will be descriptive in nature to allow the DSMB to determine the clinical significance of any reported differences between groups and to limit the risk of drawing inaccurate conclusions based on small numbers of events and chance findings at an early stage. Specific analysis may be requested in advance of a given DSMB meeting if required.

6.1 First meeting

At the first meeting, the draft DSMB charter will be reviewed and finalised prior to final sign-off by the sponsor, investigator and DSMB members. Any DSMB members unable to attend must confirm their agreement with the contents before the final version of the charter is signed. The first meeting should occur preferably before recruitment is started or at least during the first month of recruitment.

At the opening meeting the structure of the open and closed meeting reports must also be agreed.

6.2 Follow-up meetings

The first follow-up meeting should be planned to occur once the first 235 children (i.e. the first cohort) have been randomised and completed at least 1 month of the intervention but before they completed the 3 months of the intervention. A second follow-up meeting should occur after the second cohort of 235 children completed the 3 months of the intervention. A third follow-up meeting should occur when tria data collection is completed (i.e. when the last child in the third cohort completes the study).

This schedule may be modified at any stage based on recommendations made by the DSMB.

6.3 Final meeting

A final DSMB meeting will be scheduled once data for the primary endpoints is available. At this final meeting the DSMB will review the data analysis plan and may additionally comment on the interpretation of the results.

6.4 Additional meetings

Additional meetings of the DSMB will be required in the event that a pause rule is met (please refer to Section 2.4).

7 Trial documentation and procedures to ensure confidentiality and proper communication

7.1 Provision of documents

The documents for review should generally be provided to DSMB members at least 7 days before the scheduled meeting. The data for review by the DSMB statistician should also be provided at least 7 days before the scheduled meerting. The only exception to this would be for additional meetings called urgently when the documents may be presented to the DSMB on the day of the meeting, although, every effort will be made to provide the documents in advance of the meeting. Summarised scientific update information (as section 7.2) will be presented on the day of the meeting, but any available published literature (where relevant) will be provided to the DSMB in advance.

7.2 Scientific update

The Principal Investigator, following prior discussion with the Trial Steering Committee, will initially present any updated external information which is considered of relevance to the ongoing IHAT-Gut trial. In particular, this would include any new published data or new systematic reviews which are considered to be relevant to evaluate the scientific value or risk-benefit of the IHAT-Gut trial.

7.3 Open sessions

The following pooled data will be available to the open session of the DSMB:

a. Recruitment and follow-up numbers (children consented; children randomised and enrolled into the interventions; withdrawals and loss to follow-up)

b. Safety data:

- Adverse events (AEs)

- Serious adverse events (SAEs)
- Adverse reactions (AR)
- Serious adverse reactions (SAR)

Primary endpoints:

- Proportion of children with at least one episode of moderate-severe diarrhoea over the intervention period (period prevalence)
- Total number of events for moderate-severe diarrhoea per child over the intervention period (incidence density)

Secondary endpoints:

- The proportion of days a child has moderate-severe diarrhoea over the intervention period (longitudinal prevalence)
- The number of bloody diarrhoea episodes per child-month of observation (incidence density of bloody diarrhoea)
- Malaria infections
- Acute upper and lower respiratory tract infections
- Hospitalisation
- Discontinuation of supplementation due to Hb<7 g/dl

c. Protocol deviations (missed daily supplement, missed samples etc)

d. Protocol violations

e. Non-compliance

f. Data entry timelines, data completeness, data queries and data quality

7.4 Closed sessions

In addition to all the material already reviewed during the open session, the closed session material will include the breakdown of the safety data (point 'b.' above) by coded treatment arm (A,B or C). The DSMB will remain blinded to the treatment code group. The DSMB may request unblinding of the

treatment codes if significant statistical differences (p<0.05) are observed between the study arms which are deemed by the DSMB to pose important safety concerns for the study participants or in individual cases if there is a pattern of SAEs which may be related to study treatment where the DSMB feels there is 'potential for harm'. If this is the case, an independent MRC statistician will provide the DSMB statistician with the code key. The Sponsor representatives and the study team will remain blinded.

7.5 Communication

The DSMB chair or his delegate should generally aim to provide feedback to the investigator team immediately on completion of the closed session unless there are additional questions which require clarification.

A brief report summarising the recommendation should be provided to the sponsor and principal investigators, through the TSC, within 7 days of the meeting. Such a report requires only the outcome of the meeting to be provided. A summary of any discussions should not be provided as this would risk unblinding the sponsor and investigators.

Contact of TSC Chair: Dr Margaret Pinder mpinder@mrc.gm (copy to the PI: diap2@cam.ac.uk)

8 Decision making

The DSMB may recommend:

a. No action needed, trial continues as planned.

b. Stopping recruitment within a subgroup or the entire trial but allowing all recruited children to continue follow-up as planned.

c. Stopping the trial and providing all children with the standard-of-care iron supplement for treatment of iron deficiency anaemia according to the national guidelines.

d. Stopping a single arm of the trial and provide all children in that arm only with the standard-of-care iron supplement for treatment of iron deficiency anaemia according to the national guidelines. Recruitment and follow-up to the other arms to continue as planned.

e. Sanctioning or proposing protocol changes.

f. Other as judged necessary by the DSMB.

Irrespective of these recommendations, safety follow-up for an additional 1 month period should continue as planned in all children.

The DSMB may also recommend additional safety or other follow-up which would be able to be initiated in an expedited fashion if judged to be warranted. Such a change would generally involve a protocol amendment.

In the absence of relevant baseline data regarding the incidence of adverse events and serious adverse events, particularly those related with diarrhoea, in children between 6-35 months living in the Upper River Division of The Gambia, decisions related to the continuation of the trial should be made with reference to data in the placebo group. The DSMB may request unblinding of the treatment codes if significant statistical differences ($p<0.05$) are observed between the study arms which are deemed by the DSMB to pose important safety concerns for the study participants or in individual cases if there is a pattern of SAEs which may be related to study treatment where the DSMB feels there is 'potential for harm'.

No formal statistical rules will be set to determine the outcome of the DSMB review which should instead be based on the number of expected and unexpected adverse events in each group and their significance as judged by DSMB members.

The aim of the DSMB meeting should be to achieve a consensus decision regarding the recommendation made and resorting to a majority vote should be avoided whenever possible.

On occasions, it may be deemed necessary by the chair of the DSMB to seek the opinion of any members of the DSMB who are not in attendance prior to the final decision being made. A majority decision based on a vote by DSMB members may be required on occasions. The decision making process should not be revealed to those outside the DSMB.

9 Reporting

The outcome of the DSMB meeting should be reported by the chair of the DSMB, or his delegate, to the sponsor via the Trial Steering Committee (TSC, contact in 7.5).

The letter should confirm whether the trial can continue as planned or whether any modifications are recommended (as Section 8). If the DSMB recommend continuation, a simple statement to this effect is sufficient unless the chair feels additional details are required. A more detailed explanation is required if modifications are required, in which case, further discussion between the DSMB chair, Trial Steering Committee, principal investigators and Sponsor are likely to be required.

In the event that the DSMB, the Trial Steering Committee and Sponsor are not in agreement regarding the current recommendation, additional external expertise will be required to reach a conclusion and will be obtained at the time. The expertise required is likely to depend on the nature of any disagreement. Unblinding particular members of the investigator team might on occasions be required under such circumstances although should be avoided whenever possible.

All data provided to the DSMB and all deliberations of the DSMB will be privileged and confidential. The DSMB will agree to use this information to accomplish the responsibilities of the DSMB, as outlined in this DSMB Charter, and will not use it for other purposes without written consent from the sponsor. Individual DSMB members must not have direct communication regarding the study outside the DSMB (including, but not limited to communication with the investigators, the Gambia Government/MRC Joint Ethics Committee, the Medicines Control Agency of The Gambia, or sponsor) except as authorised by the DSMB.

10 After the trial

A final meeting will take place at the end of the trial (please refer to Section 6.3).

The members of the DSMB will be acknowledged in the main publication resulting from the trial. DSMB members should maintain confidentiality related to specific DSMB activities undertaken during the trial and the data reviewed indefinitely.

11 APPENDIX – DSMB SIGNATURE PAGE

A novel nano-iron supplement (IHAT) to safely combat iron deficiency and anaemia (IDA) in young children: a double-blind randomised controlled trial

The IHAT-Gut Trial

Protocol number: SCC1489

I confirm that I have read, understood and will work according this DSMB charter (Version 1.0, 15th May 2017). I will also work consistently according to the ethical principles that have their origin in the Declaration of Helsinki and that are consistent with the principles of Good Clinical Practice and applicable laws and regulations.

I confirm that all information provided regarding the trial and all matters discussed by the DSMB will be treated as confidential.

I confirm that I have no significant conflicts of interest which prevent me from sitting on the DSMB according to the definitions set out in this charter. Should this situation change at any time during the trial I will declare this and realise that I may not, under such circumstances, be able to continue to be a member of the DSMB.

Any possible conflicts of interest are listed below:

____

____

____

____

____

[Indicate 'No conflicts of interest' if this is the case]

____________________ ____________________ ____________________

Print Name Signature Date
